# Single atoms of indium on hafnia enable superior CO_2_-based methanol synthesis

**DOI:** 10.1038/s41565-026-02135-y

**Published:** 2026-03-02

**Authors:** Yung-Tai Chiang, Milica Ritopecki, Patrik O. Willi, Katja Raue, Jordi Morales-Vidal, Tangsheng Zou, Mikhail Agrachev, Henrik Eliasson, Jianyang Wang, Rolf Erni, Wendelin J. Stark, Gunnar Jeschke, Robert N. Grass, Núria López, Sharon Mitchell, Javier Pérez-Ramírez

**Affiliations:** 1 Institute for Chemical and Bioengineering, Department of Chemistry and Applied Biosciences, ETH Zurich, Zurich, Switzerland; 2 NCCR Catalysis, Zurich, Switzerland; 3 Institute of Chemical Research of Catalonia (ICIQ-CERCA), The Barcelona Institute of Science and Technology, Tarragona, Spain; 4 Universitat Rovira i Virgili, Tarragona, Spain; 5 Institute of Molecular Physical Science, Department of Chemistry and Applied Biosciences, ETH Zurich, Zurich, Switzerland; 6 Electron Microscopy Center, Empa—Swiss Federal Laboratories for Materials Science and Technology, Dubendorf, Switzerland

**Keywords:** Heterogeneous catalysis, Materials for energy and catalysis, Nanoscale materials, Catalytic mechanisms, Structural properties

## Abstract

Indium–zirconium oxides rank among the most selective and stable catalysts for CO_2_ hydrogenation to methanol. Yet, despite extensive research, the mechanistic origin of the exceptional role of monoclinic zirconia remains unresolved and continues to set the benchmark in the field. Here we show that monoclinic hafnia, a wide-bandgap oxide rarely explored in catalysis, can outperform this benchmark. Nanostructured indium–hafnium oxides synthesized via flame spray pyrolysis achieve up to 70% higher indium-specific methanol productivity than indium–zirconium oxides, with the largest gains observed for single atoms of indium. Experimental and theoretical analyses reveal that a combination of stable monoclinic support surfaces, flexible chemical potential of indium single atoms and the presence of a cooperative hydride–proton reservoir collectively enhance CO_2_ activation and intermediate hydrogenation. Crucially, the precise control of surface hydroxylation is required. These findings establish a new benchmark for green methanol synthesis and provide generalizable design principles for next-generation oxide supports in single-atom catalysis.

## Abstract (web summary)

Monoclinic HfO_2_ stabilizes hydroxylated indium atoms that drive efficient CO_2_ hydrogenation to methanol, offering a general strategy for coupling single-atom catalysis with reducible metal oxides and wide-bandgap dielectric supports.

## Main

Green methanol synthesis via CO_2_ hydrogenation is a key technological pillar towards net-zero chemical and fuel production^1,2^. Copper-based syngas-to-methanol catalysts exhibit high activity, but suffer from modest methanol selectivity and irreversible deactivation by water^3,4^. By contrast, indium oxide (In_2_O_3_)-based systems have emerged as selective and stable alternatives^4–6^, with activity linked to anisotropic oxygen-vacancy formation in partially reduced InO_*x*_ clusters^7–10^. Supporting In_2_O_3_ on oxides improves stability by preventing over-reduction and sintering^4,9–12^. In particular, impregnating In_2_O_3_ (~5 wt%) on monoclinic zirconia (m-ZrO_2_) increases indium-specific methanol space–time yields (STY) 17-fold versus bulk In_2_O_3_, whereas tetragonal zirconia (t-ZrO_2_) achieves only a 9-fold boost^4,13–15^. Proposed explanations include enhanced oxygen vacancy generation^11,13–15^, interfacial lattice strain^13,16^ and electronic interactions^11,14,17^. Theory also suggests that cooperative hydride–proton stabilization, akin to frustrated Lewis pairs^18,19^, is required for methanol formation. Yet, the mechanistic origin of the unique promotional effect of m-ZrO_2_ remains unresolved, and no alternative support has matched its performance in over a decade^4^.

Guided by this, we hypothesized that effective supports should combine (1) a stable monoclinic lattice, (2) a wide bandgap to stabilize the charged hydrogen species and (3) the ability to stabilize low-valence In species associated with oxygen vacancies. A survey of the Materials Project database identified hafnium oxide (HfO_2_) as the only practical, non-toxic support satisfying these criteria (Supplementary Table 1)^20^. HfO_2_ typically crystallizes in the same monoclinic baddeleyite structure as ZrO_2_ due to closely matching ionic radii (0.76 Å versus 0.78 Å), valency (6*s*^2^5*d*^2^ versus 5*s*^2^4*d*^2^), and Pauling electronegativity (1.30 versus 1.33) of Hf and Zr (refs. ^21,22^). Its wide bandgap and high dielectric constant make it a leading high-*κ* dielectric in semiconductor and ferroelectric devices^23,24^, but these same properties have contributed to the perception of catalytic inertness, with prior studies limited to acid–base chemistry^25,26^. To evaluate its potential, we prepared indium–hafnium oxides (InHfO_*x*_) with distinct nanostructures by varying the In_2_O_3_ content, harnessing flame spray pyrolysis (FSP) to enhance the dispersion of supported metal species and oxygen-vacancy formation^15,27,28^. Strikingly, InHfO_*x*_ outperforms indium–zirconium oxides (InZrO_*x*_) across all nanostructures, enabled by the structural and electronic stability of HfO_2_. Our findings show that stabilizing indium single atoms on HfO_2_ is crucial to unlocking its exceptional promotional effect, validated through experimental and theoretical analyses.

## Nanostructured InHfO_*x*_ unlocks superior CO_2_ hydrogenation performance

**Fig. 1:**
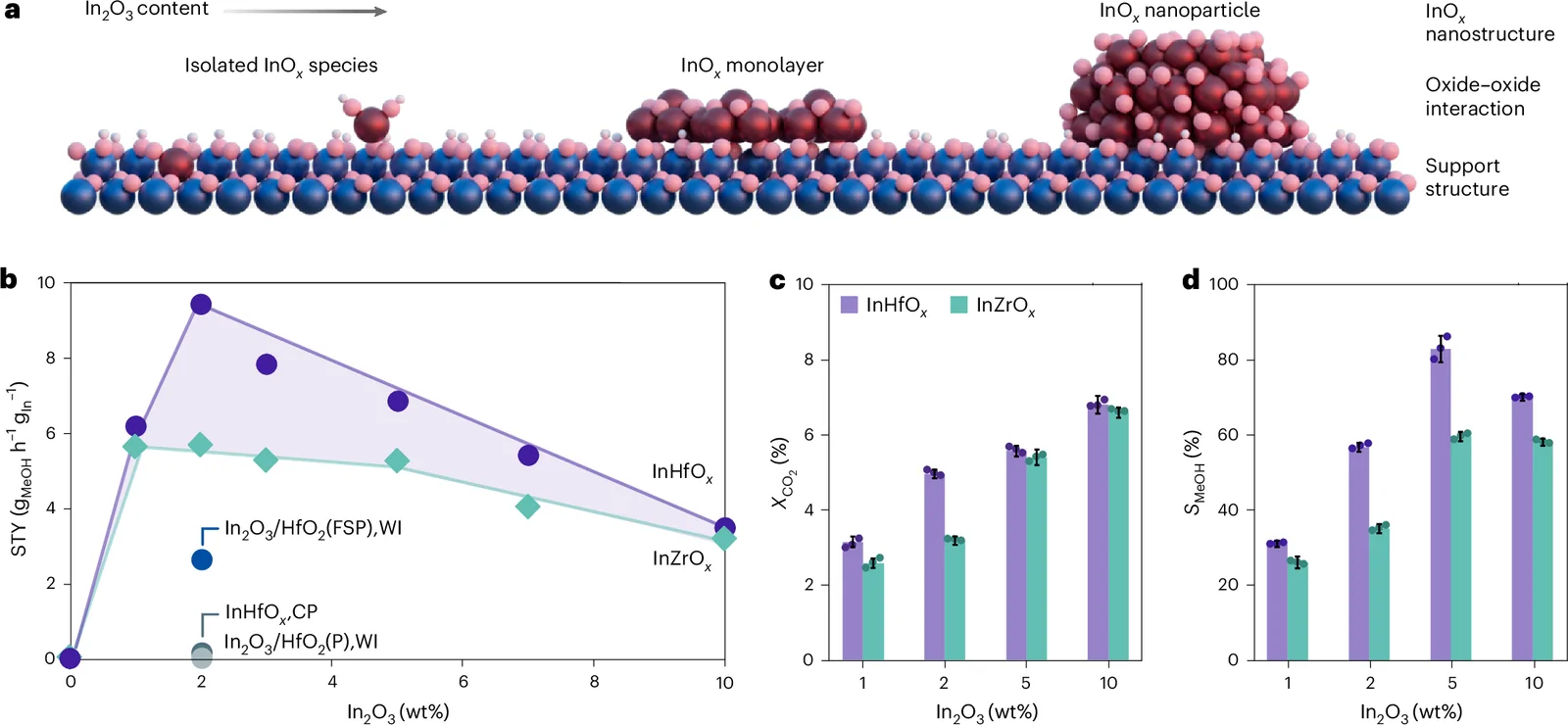
CO_2_ hydrogenation performance of InHfO_*x*_ and InZrO_*x*_ catalysts. **a**, Approach to elucidate structure–performance relations in InHfO_*x*_ catalysts by varying In_2_O_3_ content via FSP, focusing on InO_*x*_ and support structures, and their interfacial interaction. **b**–**d**, Methanol STY (**b**), CO_2_ conversion *X*_CO2_ (**c**) and methanol selectivity *S*_MeOH_ (**d**) over InHfO_*x*_ and InZrO_*x*_ catalysts with varying In_2_O_3_ content. Reaction conditions: *T* = 553 K, *P* = 50 bar, H_2_/CO_2_ = 4, GHSV = 24,000 cm^3 ^h^−1^ g_cat_^−1^, tos = 20 h. The reference catalysts in **b** include In_2_O_3_/HfO_2_,WI and InHfO_*x*_,CP, prepared by wet impregnation of HfO_2_(P) and coprecipitation, respectively, and In_2_O_3_/HfO_2_(FSP),WI, obtained by wet impregnation of HfO_2_(FSP). The highlighted area shows the difference in promotional effect between the InHfO_*x*_ and InZrO_*x*_ systems. In **d**, GHSV was varied between 24,000 and 48,000 cm^3 ^h^−1^ g_cat_^−1^ to achieve a constant CO_2_ conversion. **c**,**d**, Bars show mean values from repeated measurements (*n* = 3); error bars indicate the standard deviation. Individual data points are overlaid as dots. Source data

We prepared a series of nanostructured InHfO_*x*_ catalysts (1–10 wt% of In_2_O_3_, indicated as a prefix where relevant) via FSP. This compositional range enabled the evaluation of interactions between distinct InO_*x*_ nanostructures and the HfO_2_ support, and the impact on CO_2_ hydrogenation performance (Fig. 1a). Inductively coupled plasma optical emission spectroscopy confirms that the measured compositions closely match the nominal values (Supplementary Table 2). Under CO_2_ hydrogenation conditions (*T* = 553 K, *P* = 5 MPa, GHSV = 24,000 cm^3 ^h^−1^ g_cat_^−1^, H_2_/CO_2_ = 4), InHfO_*x*_ catalysts consistently outperform benchmark InZrO_*x*_ across all In_2_O_3_ contents (Fig. 1b and Supplementary Table 3). The highest indium-specific STY of 9.3 g_MeOH_ h^−1^ g_In_^−1^ occurs at 2 wt% of In_2_O_3_, representing a 70% improvement over InZrO_*x*_, and exceeding all previously reported supported In_2_O_3_ catalysts (Supplementary Fig. 1 and Supplementary Table 4)^15,29^. This enhancement reaches up to 66% per gram of catalyst, and ~2–3 times per unit surface area, highlighting the superior intrinsic reactivity of the In−O−Hf interface (Supplementary Fig. 2). The optimal catalyst demonstrates stable performance over 150 h (Supplementary Fig. 3), and resists CO poisoning under hybrid CO/CO_2_ feeds (up to 1:1; Supplementary Fig. 4).

Unlike InZrO_*x*_, which shows limited sensitivity to In_2_O_3_ content (1–5 wt%), InHfO_*x*_ exhibits strong compositional dependence (Fig. 1c,d). At low In_2_O_3_ content (2 wt%), the improved STY of InHfO_*x*_ arises from both higher CO_2_ conversion (~5% versus 3%) and methanol selectivity (57% versus 35%); however, at higher In_2_O_3_ contents (5–10 wt%), improvements are driven primarily by higher methanol selectivity (80% versus 60%). At 10 wt% of In_2_O_3_, performance converges with InZrO_*x*_, indicating a shift towards the dominance of bulk In−O−In motifs.

Kinetic analyses show lower apparent activation energies (*E*_a,app_) for methanol formation versus CO formation over InHfO_*x*_ catalysts, highlighting preferential methanol synthesis pathways (Supplementary Fig. 5). By contrast, 2InZrO_*x*_ favours CO formation via the reverse water–gas shift (RWGS), consistent with prior reports^30,31^. These findings suggest that the In−O−Hf interface steers reaction energetics, prompting structural and mechanistic investigations into unveiling the architecture governing InHfO_*x*_ performance.

## Interfacial architecture and support structure of InHfO_*x*_

**Fig. 2:**
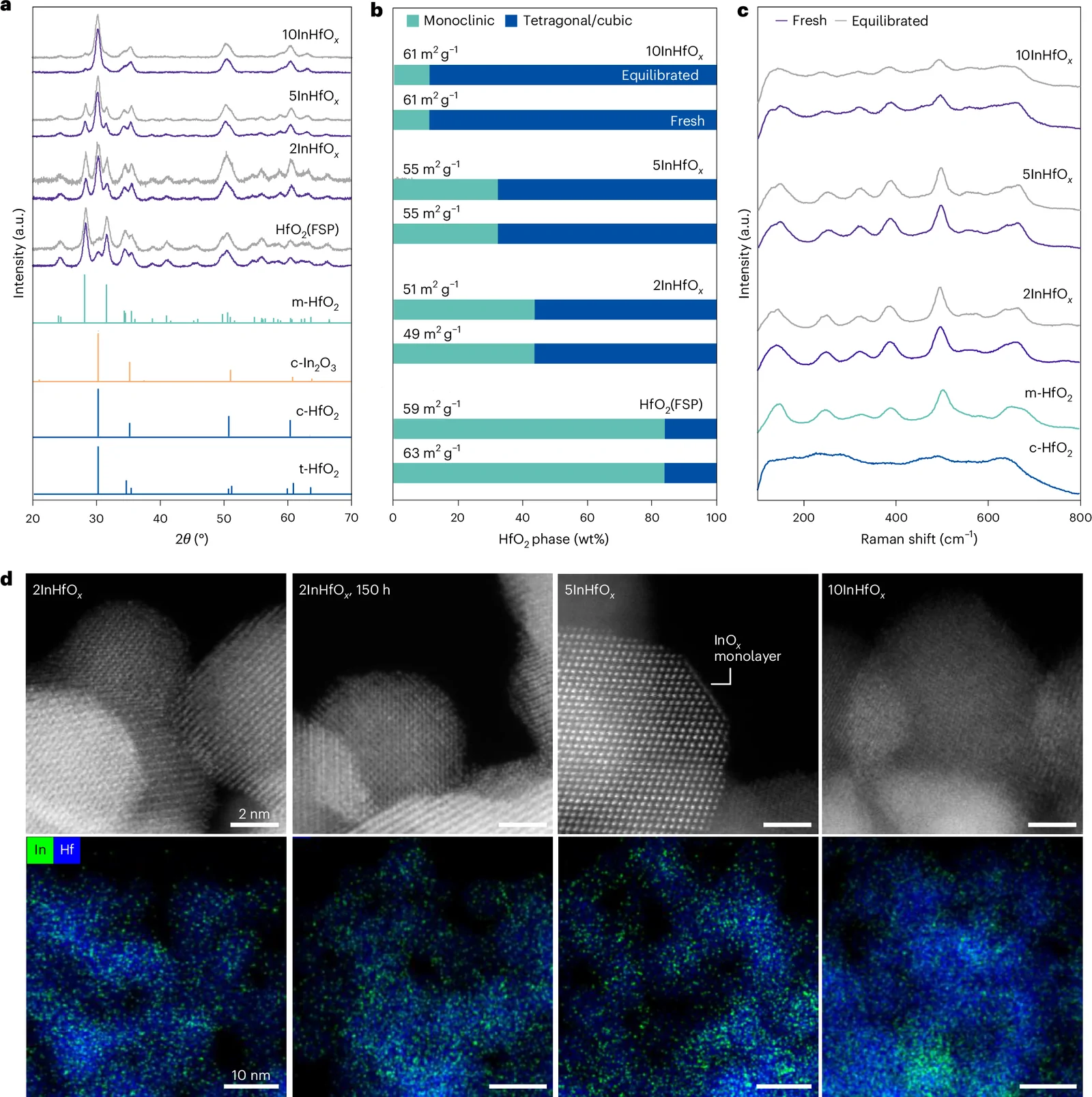
Bulk and surface structures of InHfO_*x*_ catalysts. **a**–**c**, XRD patterns (**a**), derived phase composition and specific surface areas (**b**) and Raman spectra (**c**) of selected fresh (purple lines) and equilibrated (after 20 h CO_2_ hydrogenation, grey lines) catalysts, identifying the presence of monoclinic and tetragonal or cubic HfO_2_ as bulk crystalline phases, whereas exclusively, the monoclinic lattice is observed at the surface except at high In_2_O_3_ content. **d**, High-angle annular dark-field STEM images and representative elemental maps of fresh InHfO_*x*_ catalysts and 2InHfO_*x*_ after 150 h of CO_2_ hydrogenation. The indicated scale values apply to all images in the same row. Reaction conditions in **a**–**c** are the same as those in Fig. 1. Source data

To probe changes in support structures and indium speciation with In_2_O_3_ content, all catalysts were characterized in fresh and equilibrated (after 20 h of use in CO_2_ hydrogenation) forms. X-ray diffraction analysis (XRD) reveals that at low In_2_O_3_ content, the support structure is dominated by monoclinic hafnia (m-HfO_2_), with increasing tetragonal (t) or cubic (c) fractions at higher In_2_O_3_ content (Fig. 2a,b). Overlapping reflections hinder the precise quantification of these secondary phases^32,33^. The formation of these polymorphs is probably due to the rapid quenching rate during FSP^15,27^, as they are absent in materials synthesized via other routes (as shown below). Compared with equilibrated InZrO_*x*_ catalysts, InHfO_*x*_ consistently exhibits a higher monoclinic fraction (Supplementary Fig. 6), consistent with density functional theory (DFT) Perdew–Burke–Ernzerhof (PBE) simulations, indicating a higher intrinsic stability of m-HfO_2_ (Δ*H*_f_ = 0.17 eV f.u.^−1^) relative to m-ZrO_2_ (0.11 eV f.u.^−1^; Supplementary Note 1, Supplementary Fig. 7 and Supplementary Table 5). Indium incorporation with simultaneous oxygen-vacancy formation stabilizes the cubic and tetragonal phases of HfO_2_ and ZrO_2_ (Supplementary Fig. 8). The absence of shifts in the measured support reflections indicates a minimal bulk incorporation of indium (Supplementary Fig. 9)^27^, consistent with the DFT predictions that such incorporation into m-HfO_2_ is strongly endothermic (>2 eV), even when oxygen vacancy formation is considered.

Raman spectroscopy (325-nm excitation) confirms dominant m-HfO_2_ surface modes (*A*_1g_ + *B*_1g_ = 136 cm^−1^, *A*_g_ = 502 cm^−1^) in fresh and equilibrated InHfO_*x*_ at low In_2_O_3_ content (Fig. 2c)^34,35^, which weaken at higher contents as broad features characteristic of c-HfO_2_ emerge, consistent with previous reports on heterovalent-doped c-HfO_2_ (ref. ^32^). InZrO_*x*_ systems undergo tetragonal-to-monoclinic surface transformation, leaving a minor residual tetragonal fraction (Supplementary Fig. 10), similar to previous reports^15,31^. Thus, InHfO_*x*_ retains a higher monoclinic character both at the surface and in the bulk, consistent with the higher monoclinic-to-tetragonal transition temperature of HfO_2_ than ZrO_2_ (~500 K)^22,36^.

**Extended Data Fig. 1:**
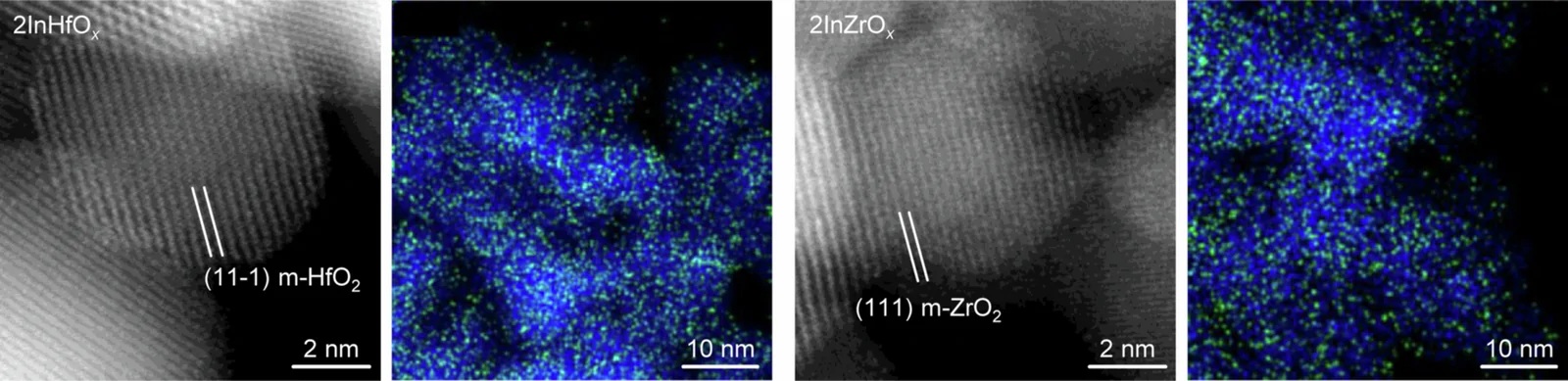
Nanostructures of 2InHfO_*x*_ and 2InHfO_*x*_ catalysts. Representative HAADF-STEM images (left) and corresponding EDS elemental maps (right) of fresh 2InHfO_*x*_ and 2InZrO_*x*_ catalysts, highlighting the observation of the monoclinic lattice and the uniform high dispersion of indium across both catalysts.

Aberration-corrected high-angle annular dark-field scanning transmission electron microscopy (STEM) imaging and energy-dispersive X-ray (EDX) spectroscopy mapping reveal a clear nanoscale resemblance between 2InHfO_*x*_ and 2InZrO_*x*_, exhibiting lattice fringes of the monoclinic supports (Extended Data Fig. 1). For 2InZrO_*x*_, isolated indium atoms are directly visible, whereas the detection of In atoms is more challenging for 2InHfO_*x*_ due to the high atomic number of Hf; however, EDX maps confirm the high dispersion of indium species and absence of sintering after 150 h on stream (Fig. 2d). Increasing the In_2_O_3_ content induces a progression from single atoms to InO_*x*_ patches and ultimately nanoparticles in 5InHfO_*x*_ and 10InHfO_*x*_ catalysts, paralleling the behaviour in InZrO_*x*_ (Supplementary Fig. 11).

This speciation trend reflects strong interfacial interaction between InO_*x*_ and m-HfO_2_, enabling epitaxial growth similar to recent reports on m-ZrO_2_ supports and electronic coupling^11–14,16^, as suggested by X-ray absorption near-edge structure spectra (Supplementary Fig. 12). The oxidic state of indium is maintained over HfO_2_, which promotes the formation of atomically dispersed active sites^10–12^. At low In_2_O_3_ content, the system achieves the highest indium utilization among supported In_2_O_3_ catalysts (Supplementary Fig. 1).

## Theoretical description of single-atom InO_*x*_ sites

**Fig. 3:**
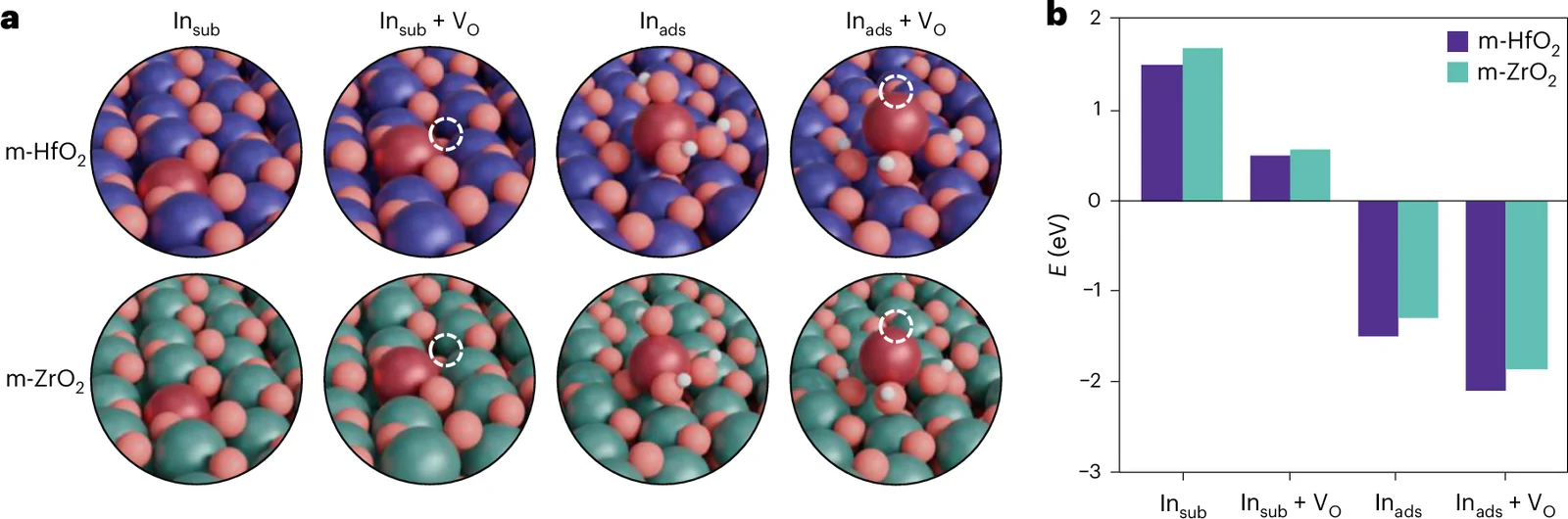
Stabilization of atomically dispersed InO_*x*_ sites on monoclinic supports. **a**, Schematic of DFT-PBE structures of the lowest-energy configurations containing single indium atom species substituted into the support surface (In_sub_) or adsorbed as In(OH)_3_ adatoms (In_ads_) with (In-V_O_) or without (In) an oxygen vacancy (dashed circle) in monoclinic HfO_2_ and ZrO_2_. Colour code of DFT models: Hf, blue; Zr, green; In, red; O, pink. **b**, Corresponding potential energy (*E*) values of the systems shown in **a**. Source data

**Extended Data Fig. 2:**
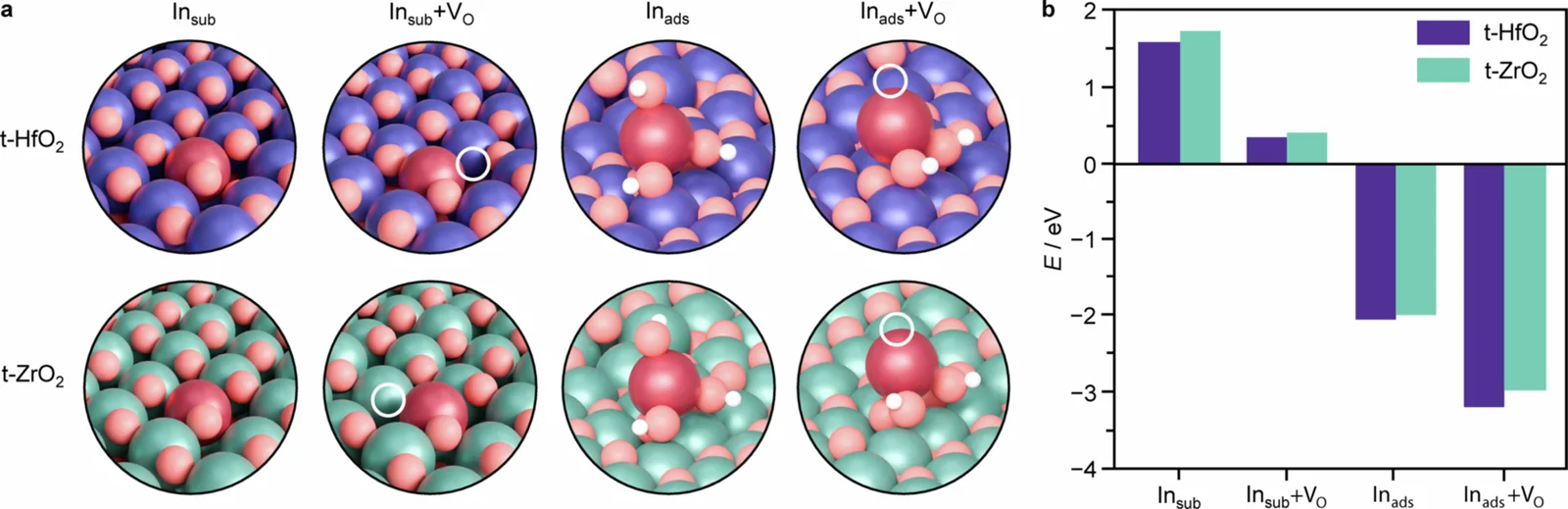
Stabilization of atomically dispersed InO_*x*_ on tetragonal supports. **a**, Schematic DFT-PBE structures of the lowest-energy configurations containing single-atom indium species substituted into the support surface (In_sub_) or adsorbed as In(OH)_3_ adatoms with (In-V_O_) or without (In) an oxygen vacancy (positions indicated by white circle) in tetragonal HfO_2_ and ZrO_2_. Colour code of DFT models: Hf - blue; Zr – green; In - red; O - pink. **b**, Corresponding potential energy (*E*) values of the systems shown in **a**. Source data

Although HfO_2_ and ZrO_2_ share structural similarity, the superior performance of indium single atoms on HfO_2_ underscores the need to resolve the atomic-level interactions in InHfO_*x*_. We, therefore, conducted DFT-PBE simulations to map the energetics governing the formation and associated catalytic behaviour of indium single atoms on HfO_2_ and ZrO_2_ polymorphs, comparing substitutional incorporation and surface anchoring (Supplementary Note 1 and Supplementary Figs. 13–15). Substitution is energetically more favourable when indium replaces Hf or Zr cations, accompanied by the formation of a neighbouring oxygen vacancy (Fig. 3, Extended Data Fig. 2 and Supplementary Figs. 16 and 17), particularly in the tetragonal phase. This agrees with the experimentally observed enrichment of the tetragonal polymorph at higher In_2_O_3_ contents, especially for InZrO_*x*_. Across all configurations, the surface segregation of indium is preferred over bulk incorporation, with a stronger driving force on HfO_2_ than ZrO_2_ (Δ*E*_seg_ = 0.41 eV; Supplementary Fig. 18). This implies fewer inactive bulk-incorporated indium species in HfO_2_, consistent with X-ray photoelectron spectroscopy (XPS) analysis evidencing slightly higher indium surface concentration in 2InHfO_*x*_ compared with 2InZrO_*x*_ (Supplementary Table 6).

Surface-anchored, partially oxyhydroxylated InO_*x*_(OH)_*y*_ adatoms (*x* + *y* < 3) are thermodynamically favoured, with In(OH)_3_ being the most stable configuration (–1.5 eV on m-HfO_2_ versus −1.3 eV on m-ZrO_2_ relative to In, H_2_ and H_2_O) (Fig. 3, Extended Data Fig. 2 and Supplementary Figs. 19 and 20). Aggregation of these species is endothermic by ~2 eV, demonstrating that isolated single atoms are intrinsically favoured (Supplementary Table 7). By contrast, the adsorption of bare indium atoms is endothermic (by 1.2–1.3 eV), underscoring the role of hydroxylation in stabilizing isolated species. Oxygen removal from surface-anchored In(OH)_3_ to form In(OH)_2_ and a hydroxyl on the support surface is energetically accessible (approximately –0.58 eV), indicating flexible indium coordination environments (Supplementary Note 1 and Supplementary Fig. 21). Ab initio thermodynamics shows that In(OH)_3_ species adjacent to a single oxygen vacancy are the most stable under CO_2_ hydrogenation conditions (Supplementary Figs. 22–26). These isolated oxyhydroxylated indium species represent the preferred architecture on both supports, though their stability depends on the support polymorph and oxygen vacancy configuration.

## Synthesis-dependent reducibility and local environments

To assess the influence of the synthesis approach on the exceptional performance of InHfO_*x*_ we prepared 2 wt% of In_2_O_3_ reference catalysts via coprecipitation (InHfO_*x*_,CP) and wet impregnation of HfO_2_(P), synthesized by precipitation (In_2_O_3_/HfO_2_(P),WI; Supplementary Table 2). Both catalysts showed negligible activity (Fig. 1b), hinting that flame-synthesized materials possess distinct surface properties. To determine whether this effect arises from the support or the mixed indium–hafnium oxide, we prepared a third reference via wet impregnation of the indium precursor onto FSP-derived HfO_2_(FSP) (In_2_O_3_/HfO_2_(FSP),WI), which exhibited improved, but still lower, performance (~25% methanol STY of 2InHfO_*x*_). This confirms that the direct flame synthesis of the mixed oxide is required to fully unlock superior performance.

**Extended Data Fig. 3:**
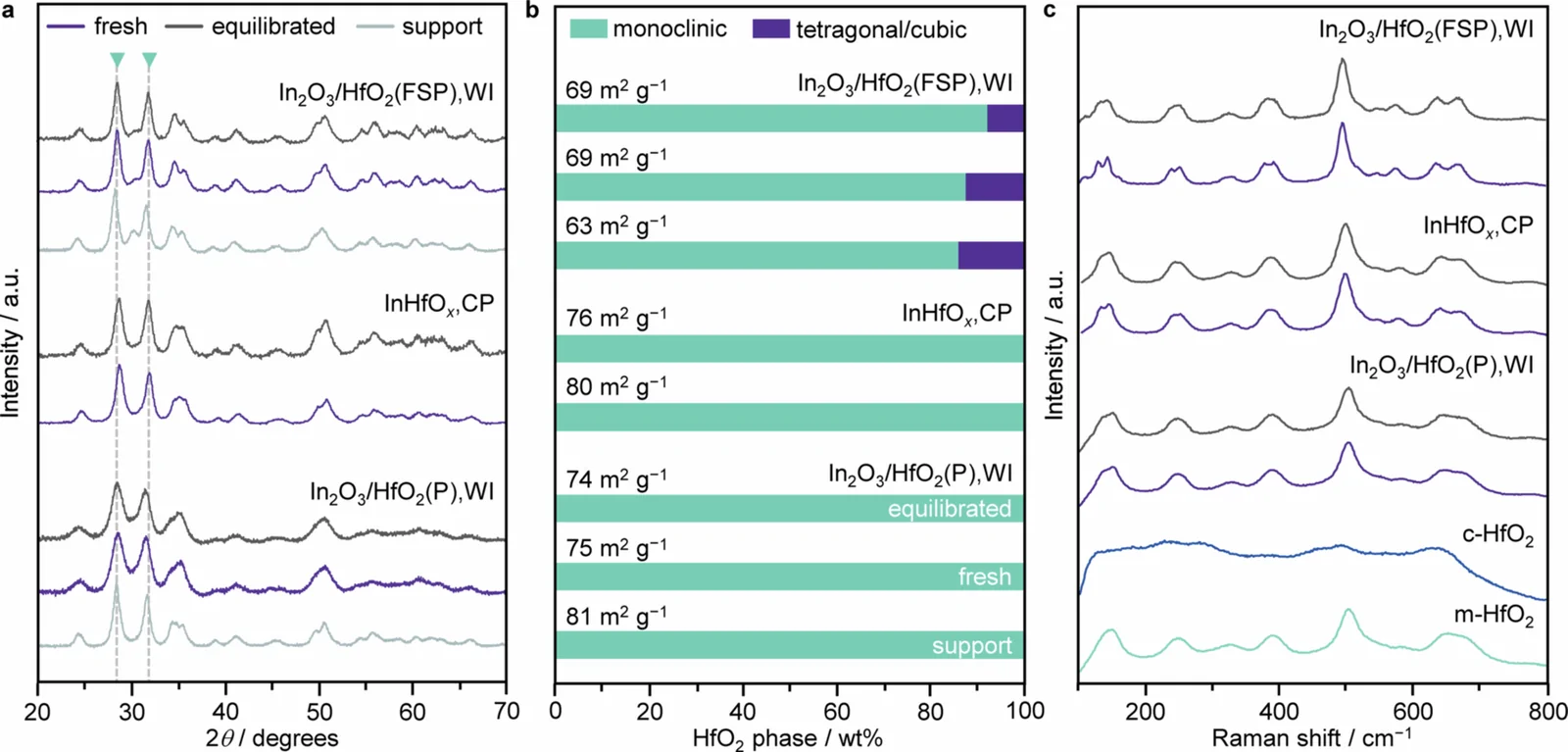
Bulk and surface structure of reference catalysts. **a**, XRD patterns **b**, Derived phase composition and specific surface areas, and **c**, Raman spectra of In_2_O_3_/HfO_2_(P),WI, InHfO_*x*_,CP, and In_2_O_3_/HfO_2_(FSP),WI catalysts in fresh (purple lines) and equilibrated (dark grey lines) forms. In (**a**), the profiles of the respective supports are indicated by light grey lines. The crystalline structures of the catalysts largely resemble those of the pure supports, while the surface only exhibits the monoclinic phase in all cases. Reaction conditions as indicated in Fig. 1. Source data

XRD and Raman measurements show predominantly monoclinic HfO_2_ for all reference catalysts (Extended Data Fig. 3), implying that the monoclinic phase alone is insufficient for high performance. STEM-EDX mapping confirms the presence of atomically dispersed indium species in all catalysts (Supplementary Figs. 27 and 28), and comparable specific surface area and In K-edge X-ray absorption near-edge structure spectra (Supplementary Fig. 29) exclude the aggregation of InO_*x*_ and over-reduction to the metallic phase as the determining factors. Instead, these observations suggest that the local coordination environment of surface InO_*x*_ species is synthesis dependent and controls reactivity.

**Fig. 4:**
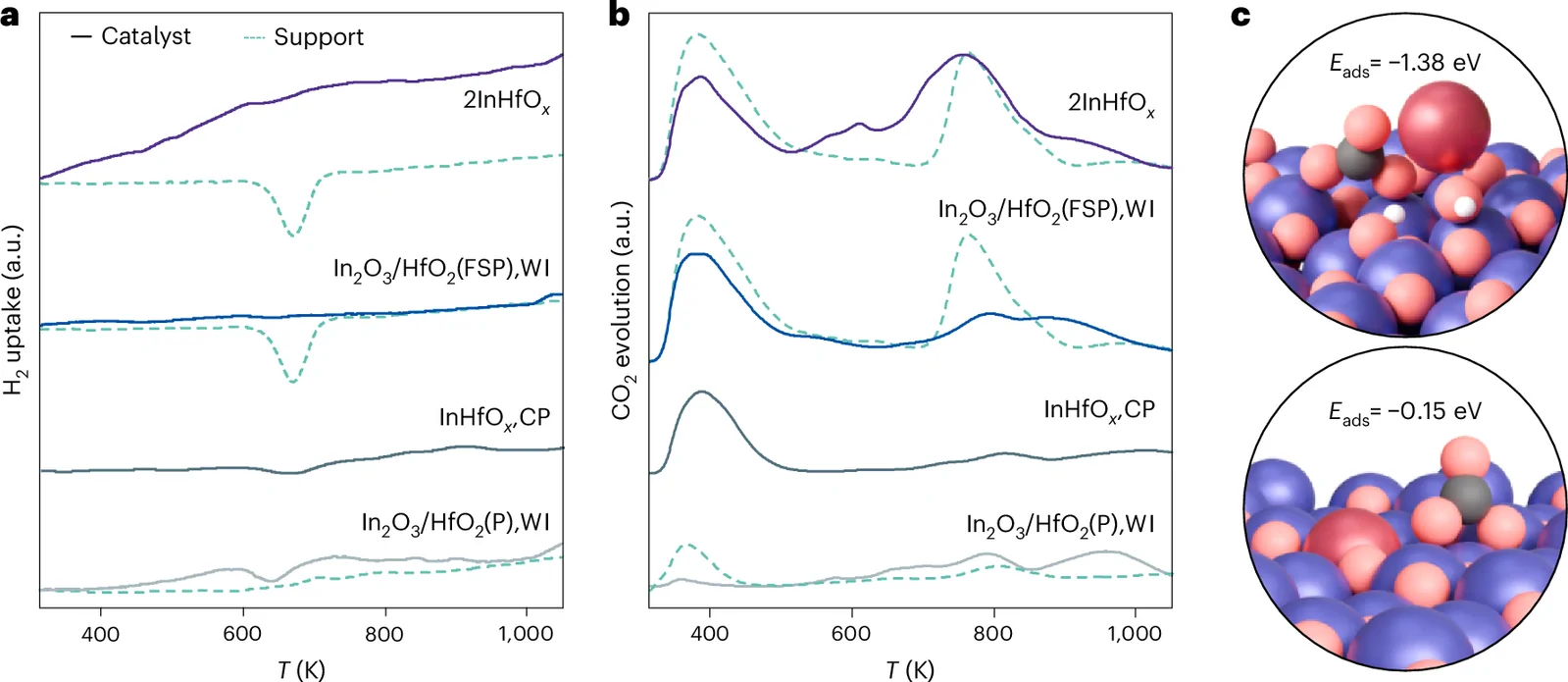
Impact of synthesis approach on CO_2_ adsorption. **a**,**b**, H_2_-TPR profiles (**a**) and CO_2_-TPD profiles (**b**) of 2InHfO_*x*_ and reference catalysts, highlighting the lower extent of chemisorbed CO_2_ on catalysts in which HfO_2_ was prepared by precipitation routes. The grey dashed lines indicate the respective profiles of the HfO_2_ supports. **c**, DFT-PBE-simulated CO_2_ adsorption energies on oxygen-defficient In(OH)_3_-m-HfO_2_ (indium adatoms anchored on the support surface) and In@m-HfO_2_ (indium atoms substituted in the support lattice) models, illustrating how surface hydroxylation impacts the CO_2_ adsorption strength. Source data

**Extended Data Fig. 4:**
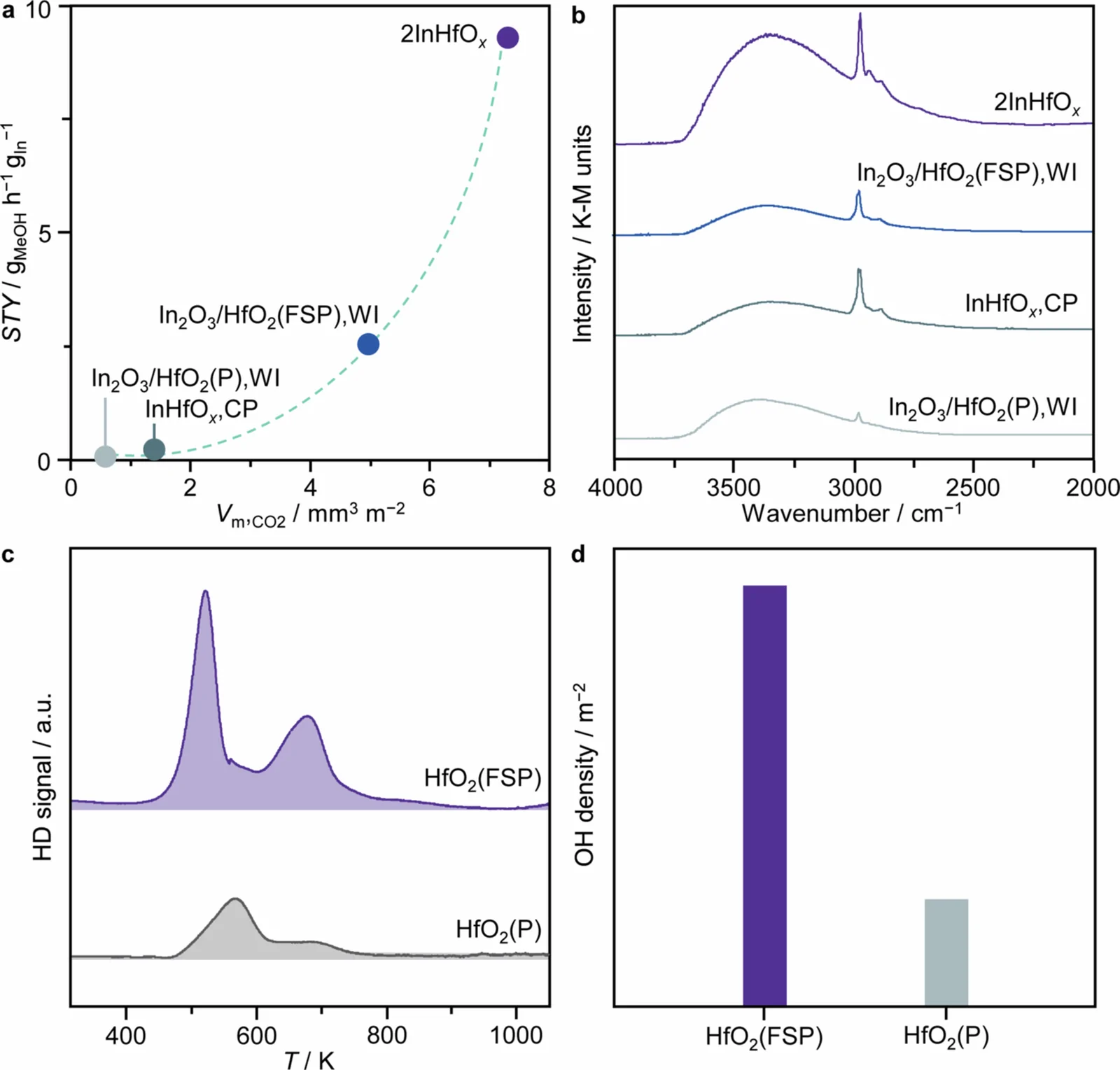
Synthesis-dependent hydroxyl group density in InHfO_*x*_ catalysts. **a**, Indium-specific methanol *STY* as a function of volumetric CO_2_ uptake over 2InHfO_*x*_, In_2_O_3_/HfO_2_(FSP),WI, InHfO_*x*_, CP, In_2_O_3_/HfO_2_,WI, and 2InZrO_*x*_ catalysts. **b**, Corresponding ex-situ DRIFT spectra of the hydroxyl region of the catalysts. **c**, D_2_/OH exchange analysis of HfO_2_ supports prepared by flame spray pyrolysis and precipitation, respectively. **d**, Normalized density of surface OH groups on the supports estimated from the HD peak area in D_2_/OH exchange experiments. Source data

We, therefore, examined CO_2_ and H_2_ activation, as the initial steps of CO_2_ hydrogenation^5,14,31^. All catalysts exhibit comparable H_2_–D_2_ exchange temperatures (*T*_exc_), suggesting similar H_2_ activation capability (Supplementary Fig. 30). By contrast, CO_2_ chemisorption varies substantially: 2InHfO_*x*_ displays strong CO_2_ uptake, whereas In_2_O_3_/HfO_2_(P),WI and InHfO_*x*_,CP show negligible adsorption (Extended Data Fig. 4a). Temperature-programmed reduction of H_2_ (H_2_-TPR) reveals a broad reduction feature in 2InHfO_*x*_ starting at ~400 K (Fig. 4a), characteristic of oxygen vacancies associated with confined InO_*x*_ species^29,30,37^. These signals are weaker or absent in the reference catalysts, indicating lower reducibility.

Temperature-programmed desorption of CO_2_ (CO_2_-TPD) confirms two desorption features (at 380 K and 760 K), assigned to CO_2_ chemisorbed on hydroxyl groups and oxygen vacancies, respectively^4,29,38–40^. Both features are diminished in reference systems (Fig. 4b), suggesting reduced oxygen-vacancy density and surface basicity. In situ diffuse-reflectance infrared Fourier transform spectroscopy (DRIFTS) reveals intense OH-stretching bands (2,500–3,700 cm^−1^) in flame-made catalysts, which are notably weaker in the reference materials (Extended Data Fig. 4b)^41,42^. Given that the CO_2_-TPD profiles of binary oxides resemble those of bare HfO_2_ supports, these observations indicate that surface hydroxylation plays a critical role in CO_2_ activation. Hydrogen–deuterium (H–D) exchange experiments further demonstrate a threefold higher surface OH density in HfO_2_(FSP) versus HfO_2_(P) (Extended Data Fig. 4c,d), corroborated by increased hydroxylated oxygen species observed in the XPS profiles (Supplementary Fig. 31). Finally, NH_3_-TPD analysis reveals a higher density of Lewis acidic sites in reference catalysts (Supplementary Fig. 32), resulting in a competitive inhibition of CO_2_ activation.

DFT simulations corroborate these observations: CO_2_ adsorption is 1.23 eV more exothermic on hydroxylated indium adatoms than on substitutional indium sites (Fig. 4c). Accordingly, hydroxylated indium adatoms are taken to best represent FSP-derived 2InHfO_*x*_, whereas substitutional models better describe InHfO_*x*_,CP and In_2_O_3_/HfO_2_(P),WI. These differences originate from distinct thermal histories. In FSP, the mismatch in melting points between In_2_O_3_ (2,180 K) and HfO_2_ (3,031 K) or ZrO_2_ (2,988 K), combined with high flame temperatures (2,500–3,000 K) and rapid quenching in air, promotes independent nucleation and anchoring of hydroxylated indium species on the support surface. By contrast, the absence of hydroxyl groups on the surface of precipitated HfO_2_ due to the high calcination temperature (973 K) precludes the formation of hydroxylated indium species. To confirm whether surface hydroxylation, and not another aspect of the FSP synthesis, is the key factor, we accessed monoclinic HfO_2_ via controlled hydrothermal treatment followed by calcination at a lower temperature (673 K). The resulting In_2_O_3_/HfO_2_(H),WI catalyst retains surface hydroxyls and matches the methanol productivity of 2InHfO_*x*_ (Supplementary Fig. 33), demonstrating that a hydroxylated surface, not the synthesis method, is decisive for high performance.

## Electronic structure and oxygen-vacancy formation

**Fig. 5:**
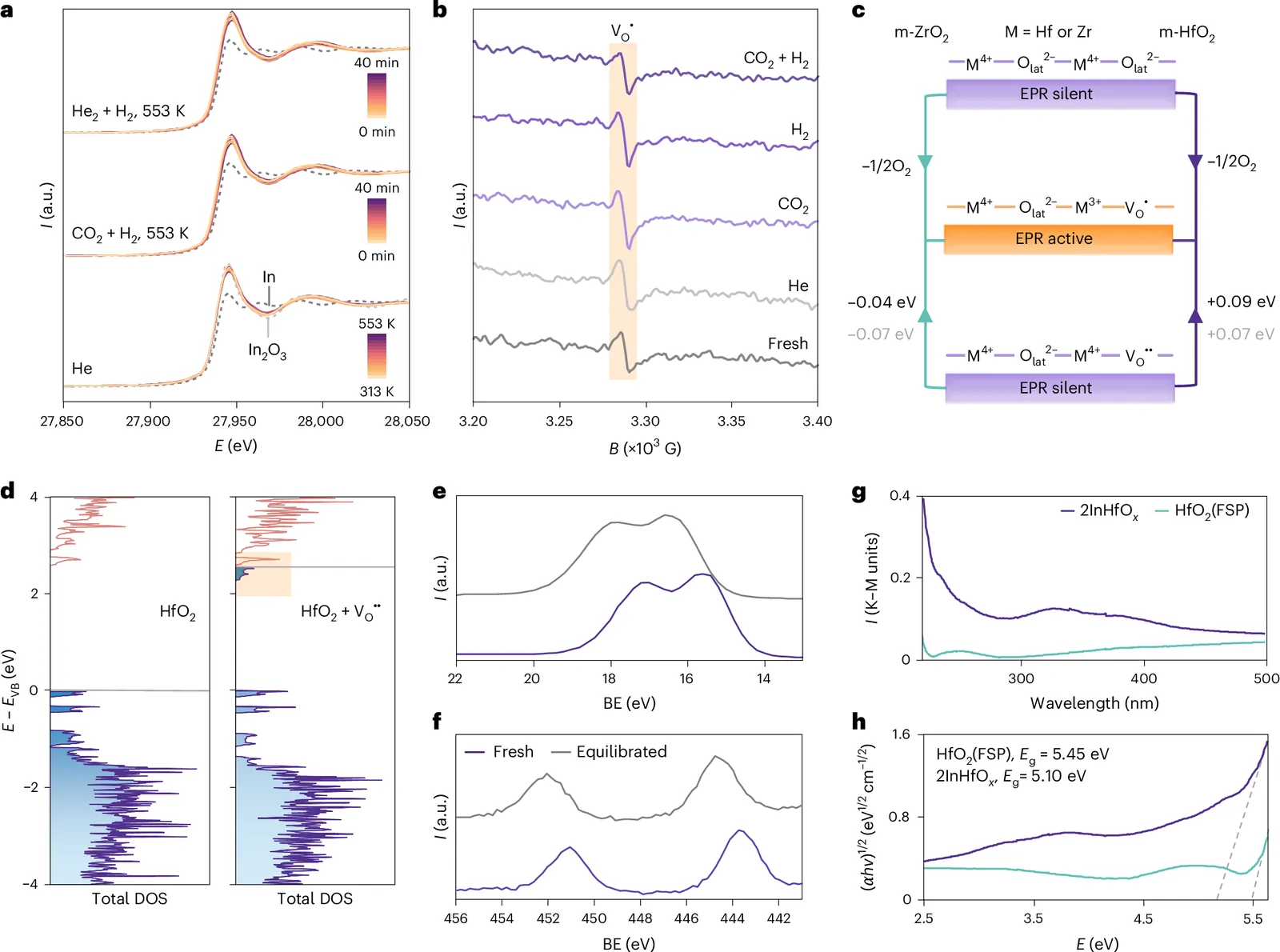
Oxygen-vacancy formation and electronic structure. **a**, Evolution of In K-edge X-ray absorption near-edge structure spectra of 2InHfO_*x*_ under He (553 K, 1.5 MPa), CO_2_ + H_2_ (553 K, 15 bar, H_2_/CO_2_ = 4) and He + H_2_ (553 K, 15 bar, H_2_/He = 4). **b**, In situ EPR spectra of fresh 2InHfO_*x*_ and after treatment in He (553 K, 10 bar), H_2_ (553 K, 10 bar), CO_2_ (553 K, 10 bar) and CO_2_ + H_2_ (553 K, 10 bar, H_2_/CO_2_ = 4). **c**, DFT-derived oxygen-vacancy formation energies on m-HfO_2_ and m-ZrO_2_, comparing the EPR active (M^3+^-V_O_^●^) and EPR silent (M^4+^-V_O_^●●^) ensembles. Values in black and grey were obtained with the HSE-13 and PBE density functionals, respectively. **d**, Total DOS for pristine and oxygen-deficient m-HfO_2_ (−111) surfaces computed at the PBE level. The DOS are aligned with respect to the valence band of the pristine system. **e**,**f**, XPS core-level spectra of Hf4*f* (**e**) and In3*d* (**f**) of 2InHfO_*x*_ catalysts in fresh and equilibrated forms. **g**,**h**, DR UV–vis absorption spectra of 2InHfO_*x*_ and HfO_*x*_-FSP (**g**) and the corresponding Tauc plots used to derive the bandgaps (**h**). Source data

**Extended Data Fig. 5:**
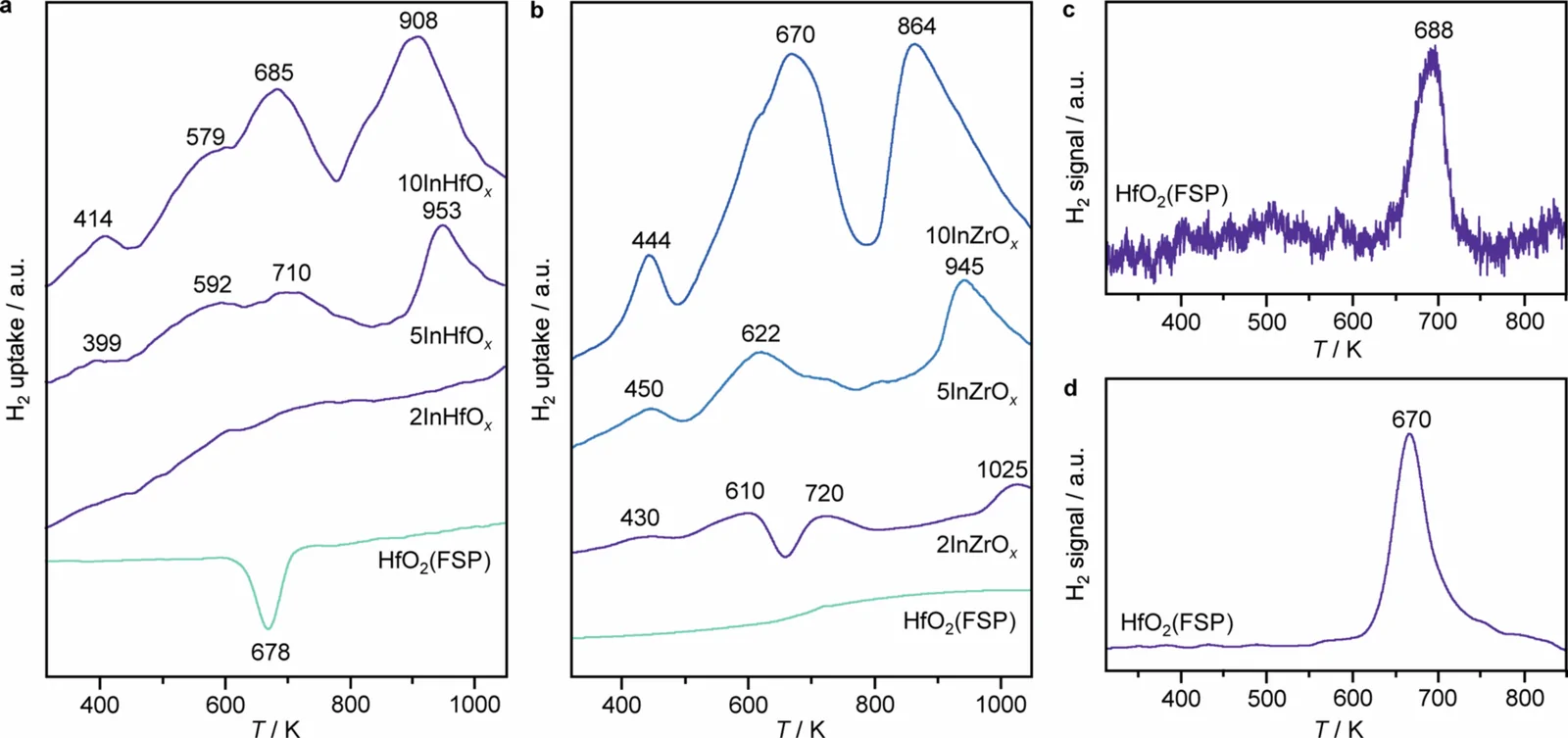
Catalyst reducibility and hydrogen storage ability. **a**,**b**, H_2_-TPR profiles of InHfO_*x*_ (**a**) and InZrO_*x*_ (**b**) catalysts prepared by FSP, with the pure oxide supports serving as references. **c**, H_2_-TPR coupled to mass spectrometry profile and **d**, H_2_-TPD profile of HfO_2_(FSP), confirming its capacity for hydrogen uptake and release. Source data

Although surface hydroxylation explains the synthesis-dependent performance, it does not fully account for the superior performance of 2InHfO_*x*_ over 2InZrO_*x*_. In particular, H_2_-TPR profiles show broader and more complex reduction features for indium single atoms anchored on HfO_2_ (~400–800 K; Extended Data Fig. 5) than on ZrO_2_, suggesting differences in oxygen-vacancy chemistry. These are paralleled by wider CO_2_-TPD peaks at ~760 K. In situ X-ray absorption spectroscopy (XAS) at the In K-edge evidences a decrease in the oxidation state of indium in both systems during thermal treatment in He, attributed to oxygen-vacancy formation (Fig. 5a and Supplementary Fig. 36)^31,43^. On subsequent CO_2_ hydrogenation (CO_2_ + H_2_), 2InHfO_*x*_ maintains a stable oxidation state, whereas 2InZrO_*x*_ undergoes further reduction, indicating a greater redox stability of indium on HfO_2_. Consistently, indium remains highly oxidized in InHfO_*x*_ following sequential treatment under more reducing conditions (H_2_ + He), whereas the white-line intensity of 2InZrO_*x*_ was further reduced. Extended X-ray absorption fine structure spectra confirm indium remains atomically dispersed in both catalysts throughout all treatments (Supplementary Fig. 37).

Electron paramagnetic resonance spectroscopy (EPR) further highlights the difference in oxygen-vacancy chemistry (Supplementary Table 8). Only an isotropic signal from EPR-silent trapped electrons at the colour centres (*g*_iso_ = 2.004, V_O_^●^) is detected in fresh 2InHfO_*x*_, whereas 2InZrO_*x*_ exhibits an additional broad ferromagnetic feature associated with the formation of coupled oxygen vacancies (Fig. 5b and Supplementary Figs. 38 and 39). Under CO_2_, H_2_ and CO_2_ + H_2_ treatments, the spectra of 2InHfO_*x*_ remain largely unchanged, suggesting the limited formation of paramagnetic vacancies and a high stability of indium atoms. Comparatively, 2InZrO_*x*_ undergoes dynamic interconversion between V_O_^●^, Zr^3+^ (*g*_*xx* _= *g*_*yy*_ = 1.98, *g*_*zz*_ = 1.96) and superoxide species (O_2_^●−^, *g*_*zz*_ = 2.035, *g*_*yy*_ = 2.01, *g*_*xx*_ = 2.002), evidencing an increased formation of redox-active coupled vacancies under reducing conditions (Supplementary Table 9 and Supplementary Note 3)^15,31^. This contrast is unexpected, as the broader H_2_-TPR reduction peaks and the lower indium-associated oxygen-vacancy formation energies predicted by DFT suggest that vacancies should also form in 2InHfO_*x*_. The absence of observable paramagnetic signals may indicate a distinct vacancy character, involving diamagnetic defects localized near indium centres, rather than the coupled paramagnetic states in 2InZrO_*x*_. Moreover, the higher atomic number of Hf could facilitate a strong spin–orbit coupling, broadening the magnetic responses of unpaired electrons and rendering such vacancies undetectable by EPR^44^.

DFT (HSE-13) simulations explain this behaviour. In HfO_2_, diamagnetic Hf^4+^-V_O_^●●^ configurations are preferred by 0.09 eV over EPR-active Hf^3+^-V_O_^●^ (0.07 eV with PBE; Fig. 5c,d), whereas in ZrO_2_, the trend is reversed. This suggests that oxygen vacancies in InHfO_*x*_ may localize electrons in a way that maintains indium in higher oxidation states, echoing in situ XAS results. This aligns with XPS analysis, which substantiates positive binding energy shifts on In3*d* and Hf4*f* after a reaction in 2InHfO_*x*_ (Fig. 5e,f), but not in 2InZrO_*x*_ (Supplementary Fig. 40), or in InHfO_*x*_ catalysts with a higher In_2_O_3_ content (Supplementary Fig. 41). Interestingly, when catalysts are exposed to air after in situ EPR experiments, we observe stronger signals associated with O_2_^●−^ radicals for 2InHfO_*x*_ than for 2InZrO_*x*_ (~1.5-fold higher), reflecting higher concentrations of reactive electrons localized in oxygen vacancies (Supplementary Fig. 42).

The difference in vacancy energetics influences the band structure. Diffuse-reflectance ultraviolet–visible (DR UV–vis) absorption analysis reveals shallow defect states near the HfO_2_ conduction band (Fig. 5g,h), aligned with the density of states (DOS) calculations that attribute these features to electrons confined in oxygen vacancies (Fig. 5d). Experimentally, both 2InHfO_*x*_ and HfO_2_(FSP) exhibit an absorption signal near the band edge (~250 nm), echoing the DFT results^45–47^. In particular, introducing indium causes a bathochromic shift in the absorption edge, partially overlapping with the energy level of oxygen vacancies and effectively narrowing the bandgap (Supplementary Fig. 43)^15^. This overlap facilitates electronic transitions and strengthens coupling between vacancies and hybridized atoms. As further evidence, 5InHfO_*x*_, which has a narrower bandgap than 2InHfO_*x*_, shows no EPR signal for isolated electrons, indicating that electrons are largely localized among different orbitals or exist as exchange-coupled magnetic polarons (Supplementary Fig. 44). By contrast, in ZrO_2_-based systems, oxygen-vacancy states lie far away from the conduction band, limiting electronic interactions with nearby atoms (Supplementary Fig. 45).

## Dynamics and stabilization of reaction intermediates

**Fig. 6:**
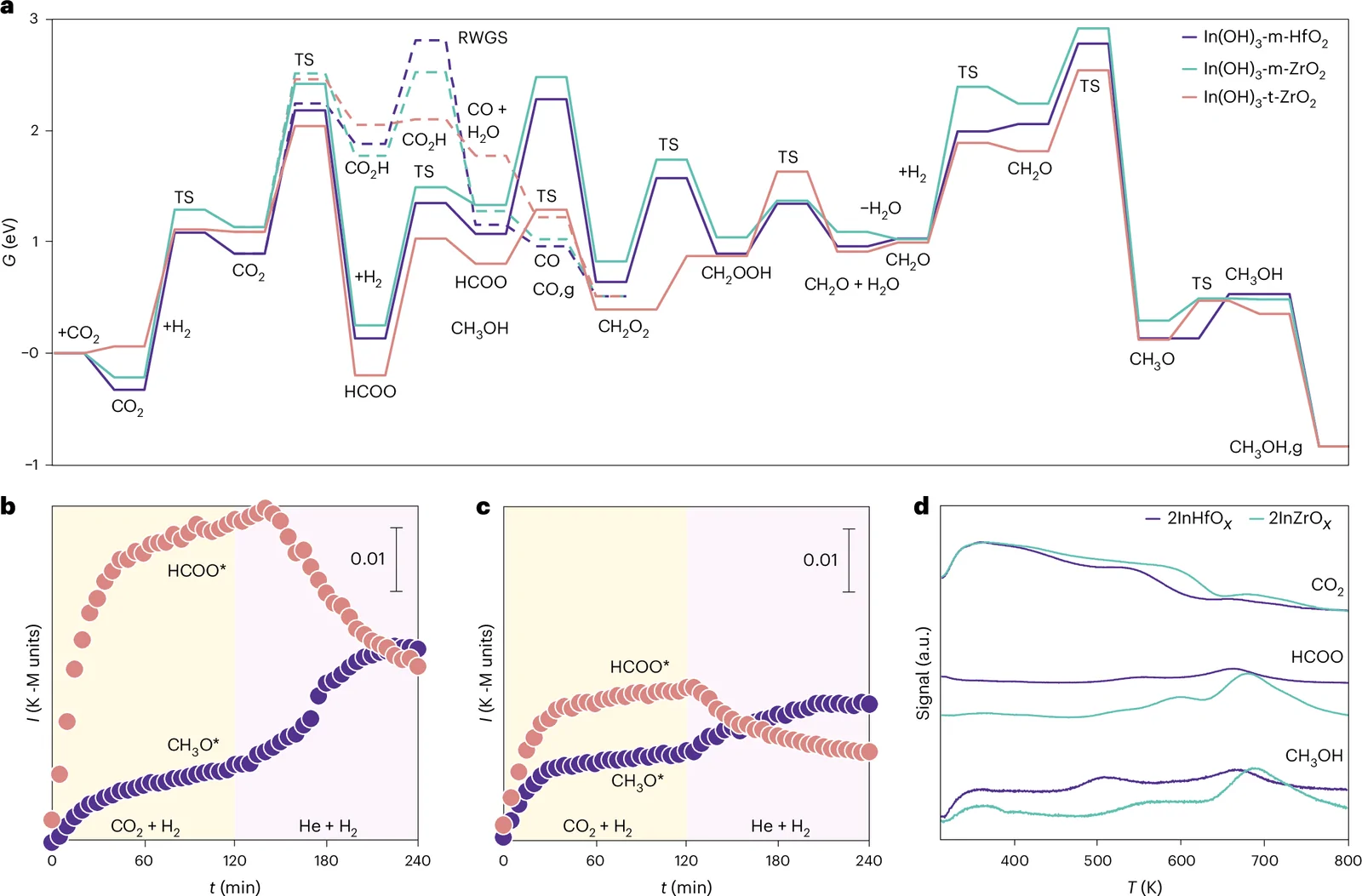
Kinetic and mechanistic analyses. **a**, Gibbs free energy (*G*) profile for reaction paths for CO_2_ hydrogenation to CH_3_OH and RWGS on the In(OH)_3_-m-HfO_2_, In(OH)_3_-m-ZrO_2_ and In(OH)_3_-t-ZrO_2_ model surfaces with one oxygen vacancy computed with PBE. TS, transition state. **b**,**c**, Evolution of the intensities of formate (HCOO*, 2,876 cm^−1^; pink) and methoxy (CH_3_O*, 2,827–2,837 cm^−1^; purple) bands derived from in situ DRIFTS measurements over 2InHfO_*x*_ (**b**) and 2InZrO_*x*_ (**c**) catalysts during CO_2_ hydrogenation (shaded yellow; *F*_T_ = 20 cm^3 ^min^−1^, *T* = 553 K, *P* = 2.0 MPa, H_2_/CO_2_ = 4; dwell time, 120 min) and subsequent H_2_/He flow (shaded purple; same conditions except that CO_2_ is replaced by He). **d**, Temperature-programmed surface reaction profiles showing the evolution of CO_2_, formate and methanol signals over 2InHfO_*x*_ and 2InZrO_*x*_ catalysts. The *y* axis is plotted on a logarithmic scale to highlight the full range of signal intensities. Source data

To investigate the origin of the high reactivity of 2InHfO_*x*_, we computed the reaction energy profiles for CO_2_ hydrogenation to methanol via the formate pathway and the competing RWGS reaction (Fig. 6a, Supplementary Note 3 and Supplementary Figs. 46–49)^7,27,48^. Simulations were performed for hydroxylated indium single atoms anchored on m-HfO_2_ and m-ZrO_2_ (denoted as In(OH)_3_-m-HfO_2_ and In(OH)_3_-m-ZrO_2_, respectively) incorporating an oxygen vacancy in the indium coordination sphere, representing the corresponding active structure of 2InHfO_*x*_ and 2InZrO_*x*_. Additional models included In(OH)_3_-t-ZrO_2_ and lattice-substituted In@HfO_2_ corresponding to reference catalysts (Supplementary Fig. 50).

CO_2_ adsorption is more exothermic on In(OH)_3_-m-HfO_2_ than In(OH)_3_-m-ZrO_2_, consistent with the higher CO_2_ conversion measured for 2InHfO_*x*_ (Fig. 1c). Thermodynamically methanol selectivity is slightly higher over In(OH)_3_-m-HfO_2_ (∆*G*_CO2+H2→HCOO_ = ‒0.75, ∆*G*_CO2+H2→CO2H_ = 0.99) compared with In(OH)_3_-m-ZrO_2_ (∆*G*_CO2+H2→HCOO_ = ‒0.88, ∆*G*_CO2+H2→CO2H_ = 0.64), in line with the experimental results (Fig. 1d). Although activation energies are similar, the In(OH)_3_-t-ZrO_2_ model overstabilizes HCOO, CH_2_O_2_ and CH_3_O intermediates, probably contributing to the lower catalytic performance of 2InZrO_*x*_. Short molecular dynamics (MD) simulations (5 ps) under relevant reaction temperatures (Supplementary Figs. 51–53 and Supplementary Videos 1–3) confirmed that indium atoms remain confined within the first coordination shell of Hf/Zr cations, and their mobility decreases in the presence of reaction intermediates adsorbed on the support surface, indicating high single-atom stability. The In@HfO_2_ model exhibits comparable methanol selectivity to In(OH)_3_-m-HfO_2_, but hindered CO_2_ and H_2_ activation steps, verifying the superior reactivity of surface-anchored, hydroxylated InO_*x*_ species.

**Extended Data Fig. 6:**
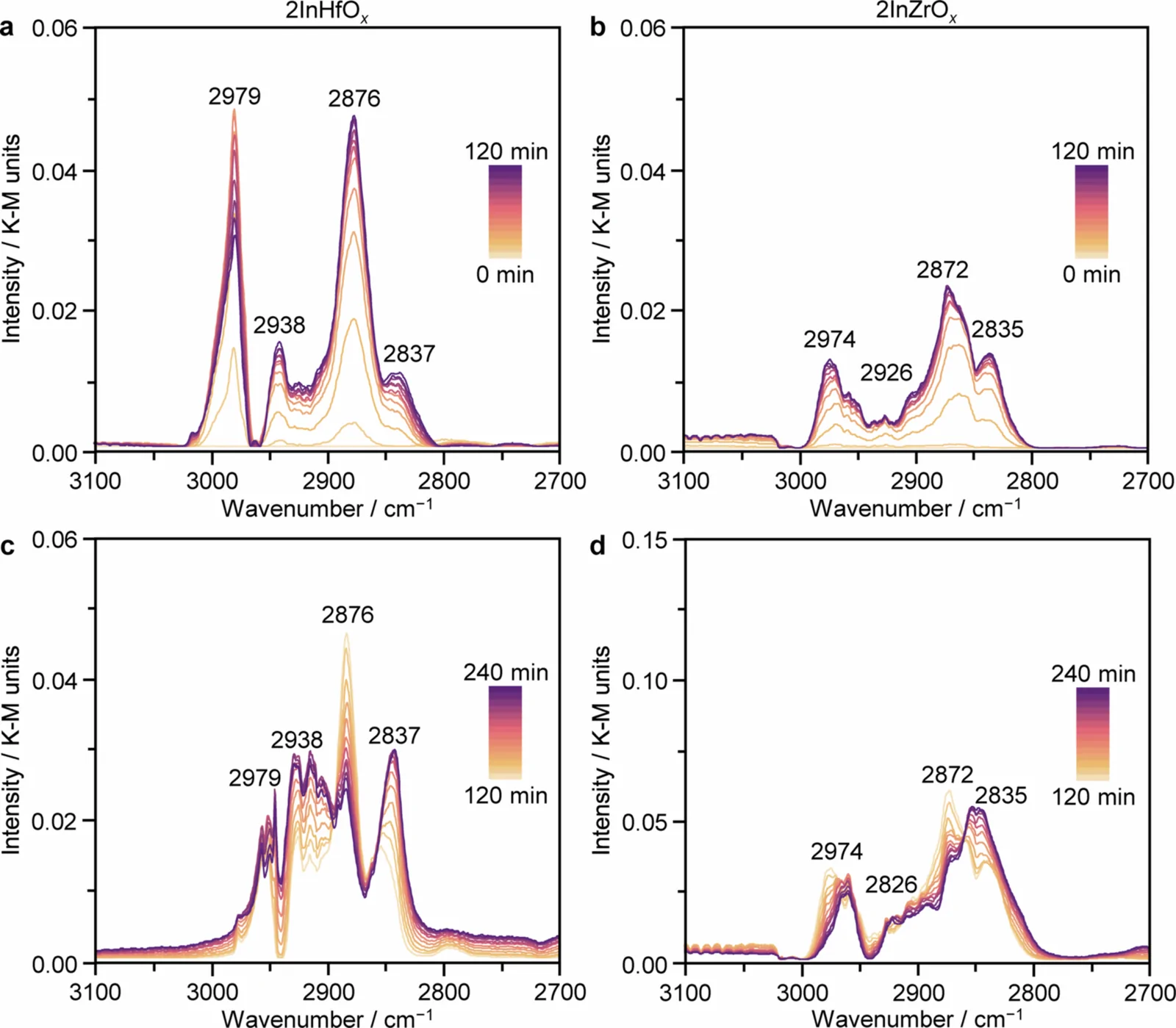
Reaction mechanism and intermediate evolution. In-situ DRIFT spectra of 2InHfO_*x*_ and 2InZrO_*x*_ catalysts **a**,**b**, under reaction conditions (CO_2_ + H_2_) and **c**,**d**, after sequential treatment in H_2_ + He. The bands at 2876-2879 and 2837-2838 cm^−1^ correspond to adsorbed methoxy species, while the 2978 cm^−1^ band observed for 2InZrO*ₓ* indicates formate-derived intermediates. Spectra are shown over time-on-stream indicated by colour gradients. Full spectra are provided in Supplementary Figs. 54-55. Treatment conditions: (*F*_T_ = 20 cm^3 ^min^−1^, *T* = 553 K, *P* = 20 bar, H_2_/CO_2_ and H_2_/He = 4, and dwell time = 120 min). Source data

In situ DRIFTS corroborates the DFT results, showing characteristic bands for formate (HCOO*; 1,385, 1,600, 2,876 and 2,978 cm^−1^) and methoxy (CH_3_O*; 1,054, 1,176, 2,837 and 2,939 cm^−1^) species on both 2InHfO_*x*_ and 2InZrO_*x*_ (Supplementary Figs. 54 and 55)^31^. Carbonate and hydroxyl signals are also observed, but DFT-PBE simulations indicate they exert a minimal influence on the reaction landscape, except in In(OH)_3_-t-ZrO_2_ (Supplementary Note 3, Supplementary Table 10 and Supplementary Figs. 56–58). Time-resolved analyses reveal approximately two times higher formate (2,876 cm^−1^) coverage on 2InHfO_*x*_, substantiating the superior reactant activation (Fig. 6b,c and Extended Data Fig. 6). On switching from CO_2_ + H_2_ to He + H_2_, formate decreases and methoxy increases on both catalysts, but with a more drastic intensity change on 2InHfO_*x*_. Methoxy signals reach maxima at 210 min for 2InHfO_*x*_, suggesting more efficient hydrogenation, whereas its desorption appears kinetically limited on 2InZrO_*x*_ as the signal remains roughly constant.

Temperature-programmed surface reaction of CO_2_ (CO_2_-TPSR) further confirms a slightly weaker CO_2_ desorption signal for 2InHfO_*x*_ during heating (Fig. 6d), with a higher formate signal, linking the enhanced formation of this key intermediate to a reduced activation barrier. Methanol production is also higher for 2InHfO_*x*_, probably aided by weaker methanol binding evidenced by methanol (MeOH) TPD, showing a 200-K lower desorption temperature (Supplementary Fig. 59 and Supplementary Table 11). These experimental results align with the DFT-derived energetics for the In(OH)_3_-m-HfO_2_ model and overstabilized intermediates on In(OH)_3_-t-ZrO_2_.

Another factor that may contribute to the superior hydrogenation performance of 2InHfO_*x*_ is the H_2_-storage capacity of HfO_2_ (refs. ^49–51^). DFT simulations indicate that the In(OH)_3_-m-HfO_2_ model promotes hydrogenation of both formate and formaldehyde (CH_2_O) intermediates. Experimentally, a negative H_2_-TPR signal from HfO_2_(FSP) is observed (confirmed to be H_2_ by mass spectrometry; Extended Data Fig. 5c). H_2_-TPD (Extended Data Fig. 5d) and H_2_ chemisorption (Supplementary Table 12) analyses corroborate the enhanced H_2_ uptake. This property arises from the wide bandgap and high dielectric constant of HfO_2_, which enable neighbouring hydride–proton pair formation that facilitates hydrogenating species transfers, efficiently completing the catalytic cycle and promoting methanol formation.

**Extended Data Fig. 7:**
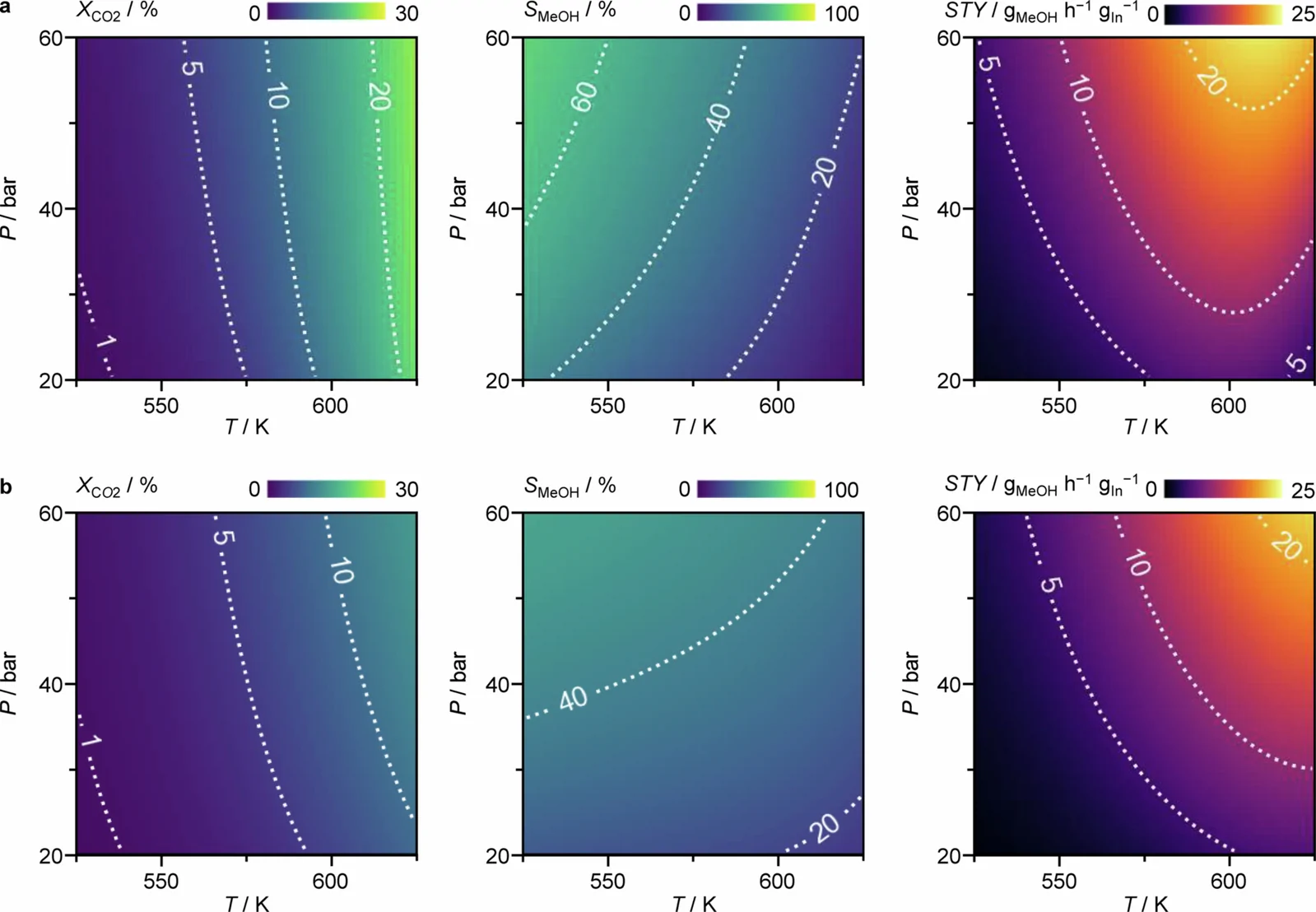
Kinetic modelling of 2InHfO_*x*_ and 2InZrO_*x*_ catalysts. Predicted CO_2_ conversion, methanol selectivity, and indium-specific methanol space–time yield from kinetic modelling of CO_2_ hydrogenation over **a**, 2InHfO_*x*_ and **b**, 2InZrO_*x*_ catalysts as a function of pressure and temperature at the same gas hourly space velocity (*GHSV* = 24,000 cm^3 ^h^−1^ g_cat_^−1^). The contour maps highlight the higher intrinsic activity and selectivity of 2InHfO_*x*_ compared to 2InZrO_*x*_ at different operating conditions. Source data

Building on a previously established kinetic framework for CO_2_ hydrogenation over reducible oxide catalysts^27,31^, we developed two-site Langmuir–Hinshelwood models for 2InHfO_*x*_ and 2InZrO_*x*_, following the same DFT-PBE-derived elementary steps. The model predictions reproduce the experimental performance (Supplementary Fig. 60), and the fitted parameters support the mechanistic picture (Supplementary Tables 13 and 14). Reaction orders for CO_2_ (*n*_CO2_) and H_2_ (*n*_H2_) are ~0.05 lower for 2InHfO_*x*_, consistent with easier CO_2_ activation and hydrogenation (Supplementary Fig. 61). The intrinsic kinetic constant for methanol formation (*k*_MeOH,ref_) is higher for 2InHfO_*x*_, whereas the RWGS activation barrier (*E*_a,RWGS_) for CO formation is lower for 2InZrO_*x*_, reflecting reduced formate stabilization. Overall, 2InHfO_*x*_ delivers superior CO_2_ conversion, methanol selectivity and methanol STY across all practical operating conditions (Extended Data Fig. 7 and Supplementary Fig. 62).

## Generalization of HfO_2_ promotional effect

**Extended Data Fig. 8:**
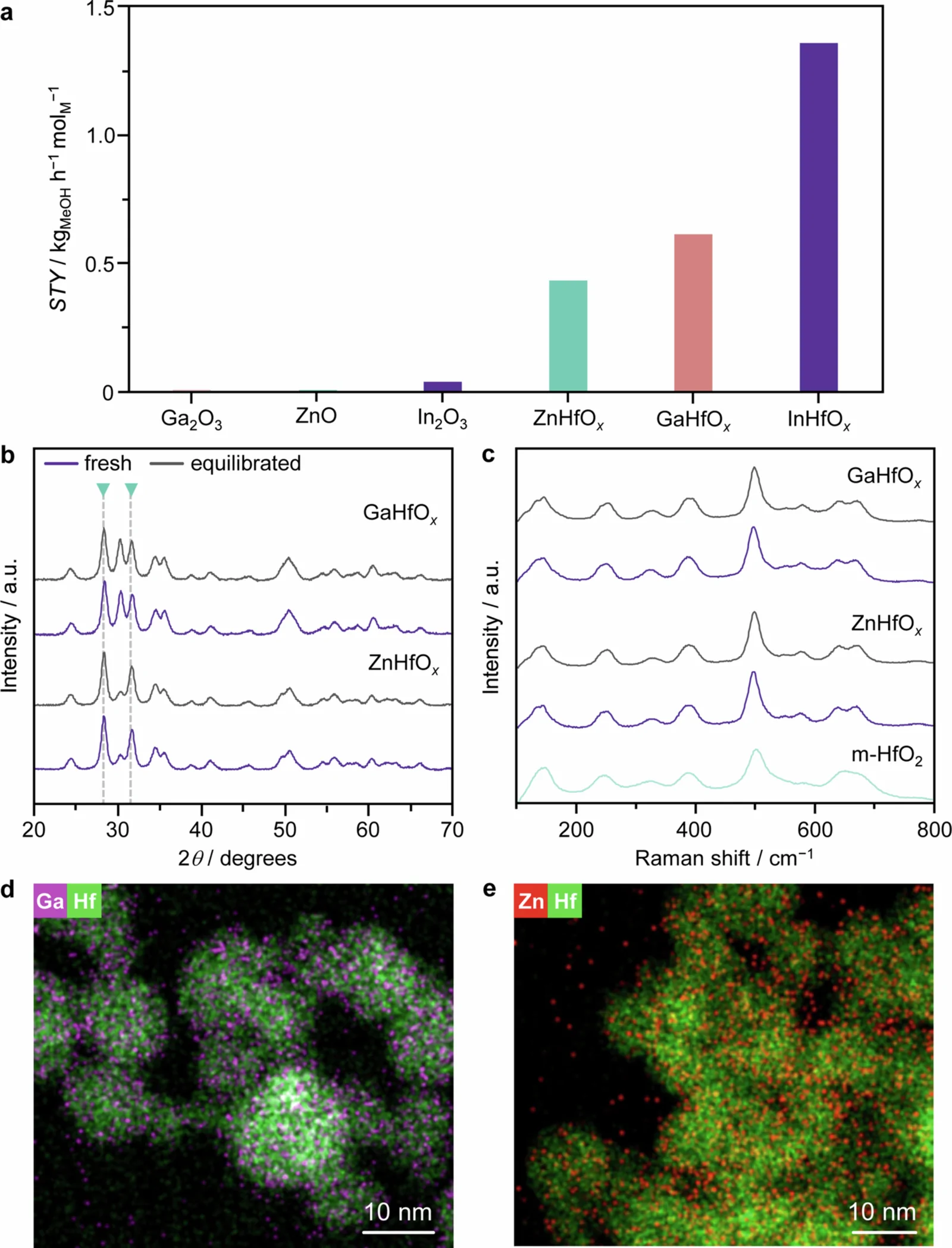
Generalization of the HfO_2_ promotional effect. **a**, Methanol *STY* per mole of active metal (M = In, Ga, or Zn) over InHfO_*x*_, ZnHfO_*x*_, and GaHfO_*x*_ containing 2 wt% In_2_O_3_, ZnO, or Ga_2_O_3_ compared to the bulk metal oxides. Reaction conditions: *T* = 573 K, *P* = 50 bar, H_2_:CO_2_ = 4, *GHSV* = 24,000 cm^3 ^h^−1^ g_cat_^−1^, *tos* = 20 h. **b**, XRD patterns and **c**, Raman spectra of ZnHfO_*x*_ and GaHfO_*x*_ catalysts. Representative elemental maps of fresh **d**, GaHfO_*x*_ and **e**, ZnHfO_*x*_ catalysts, highlighting the uniform dispersions of metal centres on m-HfO_2_ support. In (**a**), bars show mean values from repeated measurements (n = 20). Source data

To explore whether the promotional effect of flame-made HfO_2_ extends beyond indium, we synthesized analogous GaHfO_*x*_ and ZnHfO_*x*_ catalysts (2 wt% of Ga_2_O_3_ and ZnO). Although Ga_2_O_3_ and ZnO are commonly explored as alternatives to In_2_O_3_, they generally exhibit lower activity for CO_2_-to-methanol conversion^27,52–54^. The resulting materials feature similar surface areas and monoclinic support structures to InHfO_*x*_, and remain structurally stable post-reaction (Extended Data Fig. 8 and Supplementary Table 2). Under CO_2_ hydrogenation conditions (*T* = 573 K, *P* = 5 MPa, GHSV = 24,000 cm^3 ^h^−1^ g_cat_^−1^ and H_2_/CO_2_ = 4), GaHfO_*x*_ and ZnHfO_*x*_ showed metal-specific methanol productivity 268 and 137 times higher than pristine Ga_2_O_3_ and ZnO, respectively. Detailed characterization reveals that this generalized promotional effect arises from a dual-site mechanism: HfO_2_ primarily facilitates CO_2_ activation, whereas Ga and Zn single atoms split H_2_ during the reaction (Supplementary Note 5, Supplementary Table 15 and Supplementary Figs. 63–71). However, consistent with their lower activity, oxygen-vacancy formation occurs less readily on GaHfO_*x*_ and ZnHfO_*x*_, as corroborated by DFT and XPS analyses (Supplementary Table 16 and Supplementary Figs. 72 and 73). These findings demonstrate the broad applicability of the promotional effect of HfO_2_ across multiple metal oxide systems.

**Extended Data Fig. 9:**
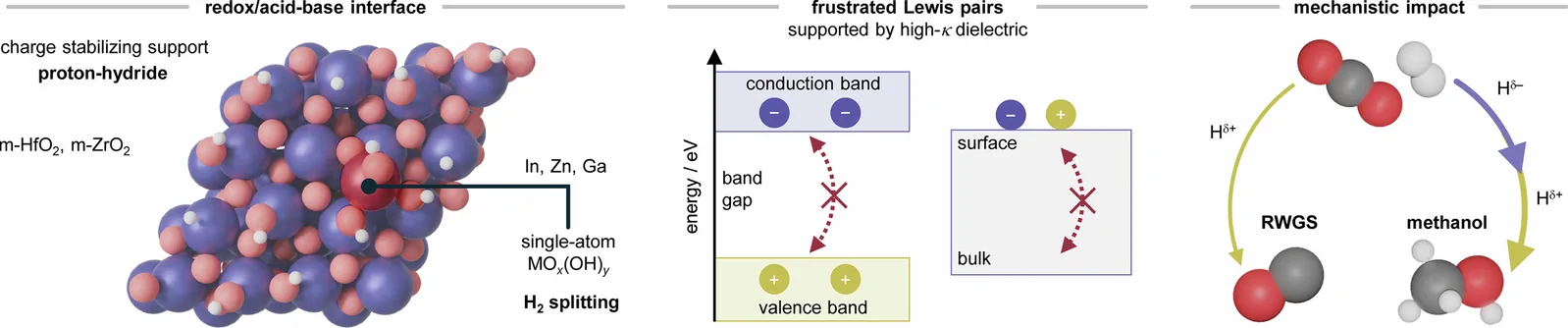
Design principles of high-*κ* dielectric catalysts. A wide bandgap combined with a high dielectric constant, provided most effectively by monoclinic HfO_2_, enables the surface stabilization of proton-hydride pairs essential for high methanol productivity over indium-based catalysts in CO_2_ hydrogenation. Hydroxylated support surfaces anchor atomically dispersed metals with flexible coordination environments (In and to a lesser extent Ga or Zn), which split H_2_. The high-*κ* dielectric prevents leakage of hydride-derived electrons from the surface into the bulk conduction band, maintaining frustrated Lewis pairs of protons bound to O^2−^ anions in the valence band and hydride electrons at the surface. This property is crucial for promoting hydrogenation steps leading to methanol, while its absence favours CO formation via the reverse water-gas shift pathway.

These results position InHfO_*x*_ as a benchmark CO_2_ hydrogenation catalyst. In particular, the effectiveness of HfO_2_ arises from the same electronic attributes that have made it transformative in microelectronics (Extended Data Fig. 9): a wide bandgap and a high dielectric constant. These features enable the simultaneous stabilization of protons on surface anions and the suppression of electron leakage from hydrides into the conduction band, preserving surface hydride–proton pairs required to close the catalytic cycle. Although this makes heterolytic H_2_ activation intrinsically difficult, the limitation is overcome by introducing single-atom hydrogen splitters (In, Ga or Zn). These metals, particularly indium, exhibit flexible coordination environments that allow variable O/H stoichiometries at nearly equivalent chemical potentials, enabling dynamic redox cycling.

High dispersion of these single atoms relies on precise synthesis control and the asymmetric monoclinic structure of HfO_2_, which suppresses InO_*x*_ aggregation. This structural requirement is underscored by the inactivity of InO_*x*_ species supported on high-bandgap but symmetric oxides like La_2_O_3_ and CeO_2_, which fail to promote CO_2_-to-methanol conversion and are unstable under reaction conditions (Supplementary Fig. 74). Additionally, hydroxylated surfaces obtained by FSP or hydrothermal synthesis, promote the formation and stabilization of single-atom hydrogen-splitting sites. The unique combination of electronic structure, surface stability and synthesis-controlled hydroxyl chemistry render HfO_2_ a compelling platform for next-generation CO_2_ hydrogenation catalysts.

## Conclusions

Monoclinic HfO_2_, a wide-bandgap, high-*κ* dielectric rarely explored in heterogeneous catalysis, unlocks superior methanol productivity in In_2_O_3_ systems, breaking a decade-long stagnation in catalyst design. This promotional effect reflects improved indium utilization, where indium single atoms anchored on the support surface act as highly efficient hydrogenation centres. This challenges the prevailing view that InO_*x*_ patches or clusters are the optimal active sites for CO_2_-to-methanol conversion. Combined experimental and theoretical analyses attribute the exceptional performance of InHfO_*x*_ to (1) higher monoclinic surface stability, (2) surface hydroxylation favoured by FSP and (3) electronic stabilization of surface hydride–proton pairs. Although the scarcity of HfO_2_ may constrain large-scale deployment^21,22^, these findings establish a generalizable design strategy for supported reducible oxides on wide-bandgap dielectrics, validated through the extension to Ga_2_O_3_ and ZnO systems. Beyond CO_2_ hydrogenation, the approach opens broader opportunities for applying dielectric-supported single-atom motifs across other challenging chemical transformations. Looking ahead, combining interface engineering, descriptor-guided design and data-driven exploration may unlock a wider class of asymmetric wide-bandgap dielectrics, ranging from pentavalent transition metal oxides to distorted perovskites, as next-generation catalytic supports.

## Methods

### Catalyst preparation

*y*InMO_*x*_ catalysts (M = Hf or Zr) with varying In_2_O_3_ content (*y* = 1–10 wt%) were prepared by FSP^15^. Metal precursors in the desired molar ratio were dissolved in 2-ethylhexanoic acid and tetrahydrofuran (1/3 mass content). The resulting solutions (total metal concentration, 0.40 mol kg^−1^) were pumped through a nozzle (0.4-mm diameter at 5.0 cm^3 ^min^−1^), dispersed into a fine spray by flowing oxygen (1.5 bar, 5.0 l min^−1^), and ignited using a supporting flame formed from a premixed flow of oxygen (2.4 l min^−1^) and methane (1.2 l min^−1^). The resulting powders were collected on a glass fibre filter (GF/A-6) and used for CO_2_ hydrogenation without further purification or treatment. GaHfO_*x*_ and ZnHfO_*x*_ catalysts, as well as reference HfO_2_(FSP) and ZrO_2_(FSP) supports, were prepared analogously using the corresponding metal precursors.

The HfO_2_(P) support was synthesized by precipitation, where aqueous NH_4_OH (25 wt%, 5 cm^3 ^min^−1^) was added to an aqueous solution of HfCl_4_ (4.8 g in 200-cm^3^ deionized water) under vigorous stirring until reaching a pH of 10. The resulting slurry was aged (48 h, 363 K, ramp of 3 K min^−1^), and the resulting precipitate was washed with deionized water (10 l) using high-pressure filtration, dried in a vacuum oven (323 K, 12 h), and calcined in air (973 K, 2 K min^−1^, 2 h). Coprecipitated InHfO_*x*_,CP catalysts were synthesized following the same protocol, except that In(NO_3_)_3_·H_2_O (0.15 g) was added together with the HfCl_4_ precursor. The HfO_2_(H) support was synthesized by a hydrothermal route. In brief, CO(NH_2_)_2_ (60 g) was dissolved in an aqueous solution of HfCl_4_ (30 g in 400-cm^3^ deionized water). The transparent mixture was subsequently transferred into a 30-ml Teflon-lined stainless steel autoclave and heated (473 K, 3 K min^−1^, 20 h). Products were then separated by centrifugation, washed with deionized water (1 l) and dried at 80 °C for 12 h. Finally, the obtained samples were calcined in air (473 K, 3 K min^−1^, 20 h). For wet-impregnated catalysts, In_2_O_3_/HfO_2_(P),WI, In_2_O_3_/HfO_2_(FSP),WI and In_2_O_3_/HfO_2_(H),WI, In(NO_3_)_3_·H_2_O (48 mg) was dissolved in deionized water (124 cm^3^) followed by the addition of HfO_2_(P), HfO_2_(FSP) or HfO_2_(H) (2 g), respectively. The suspension was stirred (700 r.p.m., 24 h, ambient conditions) before removing the solvent gradually under reduced pressure (323 K, Büchi Rotavap R-114), and the resulting powders were dried and calcined in air (773 K, 2 K min^−1^, 3 h). All chemicals, suppliers and purities are listed in Supplementary Table 17. Catalyst nomenclature and synthesis procedures are summarized in Supplementary Table 2.

### Catalyst composition and phase structure

Metal contents were quantified by inductively coupled plasma optical emission spectroscopy (Horiba Ultra 2 spectrometer, photomultiplier tube detector) after digestion in a 2:1 H_2_SO_4_:HNO_3_ mixture under microwave irradiation. Bulk crystalline structures were examined by XRD (Rigaku SmartLab diffractometer, D/teX Ultra 250 detector) using Cu Kα radiation (*λ* = 0.1541 nm) in the Bragg–Brentano geometry (2*θ* range of 10–80°, step size of 1° and 60 s per step). Average crystal sizes of m-MO_2_ phases were estimated via the Scherrer equation (*K* = 0.94) applied to selected diffraction peaks (Match! software, v3.15 Crystal Impact, instrumental broadening corrected using the LaB_6_ standard). The phase composition was estimated using the reference internal ratio method. Catalyst surface structures were confirmed by Raman spectroscopy (Horiba LabRAM HR system, 325-nm He–Cd laser, 2.5-mW power, ×40 objective lens, 0.95 numerical aperture, 1,800 lines mm^−1^ grating). Spectra were acquired in a single run (60 s). Porous properties were determined by N_2_ sorption at 77 K (Micromeritics TriStar II analyser) after degasification (473 K, 12 h, vacuum). InO_*x*_ dispersion and nanostructure were characterized by aberration-corrected STEM and EDX (probe-corrected FEI Titan Themis, Super-X detector, 300 kV) analysis of catalyst powders dusted on lacey-carbon-coated copper grids.

### Surface adsorption and reactivity

CO_2_ uptake was measured by volumetric chemisorption (Micromeritics 3Flex Surface Characterization analyser) after in situ pretreatment in H_2_ (*m*_cat_ = 0.15 g, 573 K, 1 h). After cooling to 313 K, CO_2_ was dosed stepwise (13.3-kPa increments, up to 93.3 kPa). The adsorption strength was evaluated using the dual-isotherm method; the uptake was calculated via Sinfelt extrapolation. CO_2_ adsorption was further investigated by CO_2_-TPD (Micromeritics AutoChem HP II analyser) with mass spectrometry detection (*m*/*z* = 44, Pfeiffer Vacuum OmniStar). Catalysts (0.1 g) were pretreated in He (553 K, 10 K min^−1^, 1 h), cooled to 313 K, saturated with 5%CO_2_/He (1 h), purged for 20 min under He and then heated to 1,073 K (10 K min^−1^) under He. Gas flows were 20 cm^3 ^min^−1^. CO_2_ temperature-programmed surface reaction followed the same procedure but used H_2_ in the final temperature ramp (313–973 K), monitoring *m*/*z* = 44 (CO_2_), 45 (formate) and 31 (methanol). MeOH-TPD was conducted using the same setup by pulsing methanol-saturated He (30 pulses, each 0.5 cm^3^, 2-min separation), followed by purging (313 K, 60 min) and ramping under He to 1,073 K (10 K min^−1^), monitoring *m*/*z* = 31 (methanol). Temperature-programmed H_2_–D_2_ exchange (H_2_–D_2_ TPE) was performed using the same setup, exposing the samples (*m*_cat_ = 0.05 g) to a 50% H_2_/D_2_ mixture (10 cm^3 ^min^−1^, 423 K, 60 min) and then cooling to 313 K and monitoring *m*/*z* = 2(H_2_), 3 (HD) and 4 (D_2_) on heating to 700 K. The reducibility of the catalysts was studied by H_2_-TPR (Micromeritics AutoChem HP III). After identical pretreatment, samples were exposed to a 5% H_2_/Ar mixture (20 cm^3 ^min^−1^) and heated to 1,073 K (10 K min^−1^), monitoring evolved gases by thermal conductivity detection. D_2_-H-TPE was used to quantify the hydroxyl group density of distinct supports.

### Electronic structure

XPS analyses (Physical Electronics Quantum 2000 spectrometer) were conducted using monochromatic Al Kα radiation (15-kV electron beam), using a hemispherical capacitor electron-energy analyser (46.95-eV pass energy, 45° electron take-off angle). Samples were mounted on aluminium foils. Binding energies were calibrated to the C1*s* aliphatic peak, set to 284.8 eV. Spectra were analysed using CasaXPS (v2.3.23PR1.0; Casa Software Ltd). XAS measurements were performed at the Swiss-Norwegian Beam Line at the European Synchrotron Radiation Facility using a liquid-nitrogen-cooled Si(111) double-crystal monochromator. Spectra were calibrated against In foil (27.9399 keV). The incident and transmitted intensities were monitored using N_2_-filled ionization chambers. Fluorescence signals were collected using a Peltier-cooled, single-element silicon drift detector. In K-edge spectra (27.6–28.6 keV) were acquired in the continuous-scan mode (0.5-eV step, 150-s scan duration; beam spot, 3 mm × 0.2 mm). Catalysts (10 mg) were packed in 1-mm-outer-diameter quartz capillaries (0.02-nm wall thickness), secured between quartz plugs. Operando XAS involved (1) recording the fresh catalyst spectra, (2) heating in He to 553 K (20 cm^3 ^min^−1^, 10 K min^−1^, 2 MPa), (3) switching to CO_2_ hydrogenation conditions (20 cm^3 ^min^−1^, 553 K, 2 MPa, H_2_/CO_2_ = 4) for 120 min and (4) switching to reducing conditions (20 cm^3 ^min^−1^, 553 K, 1.5 MPa, H_2_/He = 4) for 120 min, followed by cooling in He. High-purity gases were dosed using Bronckhorst digital mass flow controllers, and the effluent gases were monitored by mass spectrometry (Pfeiffer Vacuum OmniStar). Ex situ XAS was conducted with the same geometry. Reference In_2_O_3_ and In foils were measured in the transmission mode. Data processing (energy calibration, background corrected and normalized) was performed using Athena (v0.9.26) from the Demeter software package.

In situ EPR spectroscopy was conducted using a custom setup (Supplementary Table 8). Catalysts (20 mg) were loaded in 2.8-mm-inner-diameter quartz tubes (Wilmad), positioned at the centre of a water-cooled high-temperature resonator^55^, within a Bruker EMX continuous-wave EPR spectrometer operating at X-band frequencies. Gases were dosed with Bronkhorst digital mass flow controllers, and the effluent was monitored by mass spectrometry (Pfeiffer Vacuum OmniStar). The reactor was first pressurized to 10 bar under He and measured at room temperature (fresh catalyst). In situ time-resolved spectra were recorded continuously using the two-dimensional acquisition mode during the following steps: (1) heating to 553 K in He and stabilizing for 30 min, (2) cooling to room temperature in He, (3) reheating to 553 K in He until the spectra stabilized, (4) switching to a He–H_2_ flow (210 cm^3 ^min^−1^) for 90 min, (5) cooling to room temperature in a He–H_2_ flow, (6) reheating to 553 K in a He–H_2_ flow until stabilization, (7) switching to a He–CO_2_ flow (110 cm^3 ^min^−1^) for 90 min, (8) cooling to room temperature in a He–CO_2_ flow, (9) reheating to 553 K in a He–CO_2_ flow until stabilization of the EPR spectra, (10) switching to a H_2_–CO_2_ flow (200 cm^3 ^min^−1^, H_2_/CO_2_ = 4) for 90 min and (11) cooling to room temperature in a H_2_–CO_2_ flow for the final EPR spectrum. Ex situ continuous-wave EPR spectra were acquired on a Bruker Elexsys E500 spectrometer equipped with an Oxford helium (ESR900) cryostat operating at X-band frequencies using a ER4122SHQE Bruker EPR resonator (Supplementary Table 9). All spectra were analysed using EasySpin, and *g*-factors were offset-corrected against a known standard (free radical 1,1-diphenyl-2-picrylhydrazyl)^56^. The in situ spectra at room temperature were fitted using the pepper function in EasySpin. The data were optimized for *g*-values, linewidth and weights for the spin concentrations of the different spin species using the particle swarm optimization method. DR UV absorption spectra (JASCO V-770 spectrophotometer equipped with an ILN-725 integrating sphere) were recorded in the 220–800-nm range (internal, 0.5 nm).

The reaction mechanism and kinetics were studied by in situ DRIFTS using a custom-made setup previously described. The undiluted catalyst (~25 mg) was loaded into a high-temperature reaction chamber with ZnSe windows (Harrick Scientific HVC-DWM-3), mounted in a Praying Mantis accessory (Harrick Scientific). After purging with He (30 min), the cell was pressurized (2 MPa) for a leak test and then heated to the reaction temperature (5 K min^−1^). For background acquisition, spectra were collected continuously (2 cm^−1^ resolution, 256 scans per 60 s). The gas feed was then switched to the H_2_/CO_2_ = 4 reaction mixture (*F*_T_ = 20 cm^3 ^min^−1^) for 120 min, followed by H_2_/He = 4 (*F*_T_ = 20 cm^3 ^min^−1^) for another 120 min. Time-resolved signal evolution was analysed using the Bruker OPUS (v8.2) software. Ex situ DRIFTS was performed using KBr as the background.

### Catalytic evaluation

CO_2_ hydrogenation to methanol was performed in a continuous-flow high-pressure system with four parallel fixed-bed reactors (PID Eng&Tech), described elsewhere^57^. Catalyst powders (100 mg; 0.2–0.4-mm particle size) were loaded into reactor tubes (4-mm internal diameter) with quartz wool and frit. After purging with He (40 cm^3^ STP min^−1^, PanGas, 4.6, 30 min), the pressure was gradually elevated to 5.5 MPa for leak testing under an identical flow. Reactors were then heated to 553 K (5 K min^−1^), and testing proceeded at 553 K, 5 MPa, under the H_2_/CO_2_ reaction mixture (4:1 molar ratio, 40 vol% CO_2_) at a GHSV of 24,000 cm^3 ^h^−1^ g_cat_^−1^. Product analysis was performed by online gas chromatography every 1 h (parallel) or 15 min (single reactors). Response factors (*F*_*i*_) for each compound *i* were determined relative to an internal standard (20 vol% of C_2_H_6_ in He) using equation (1):

1 $${F}_{i}=\frac{{A}_{{{\rm{C}}}_{2}{{\rm{H}}}_{6}}/{n}_{{{\rm{C}}}_{2}{{\rm{H}}}_{6}}^{\mathrm{in}}}{{A}_{i}/{n}_{i}^{\mathrm{in}}},$$

where *A*_*i*_ is the integrated peak area of compound *i*, and *n*_*i*_ is the molar flow at the reactor inlet. An average of five points around the expected analyte concentration were used. The unknown effluent molar flow of compound *i* (${n}_{i}^{{\rm{out}}}$) was determined using equation (2):

2 $${n}_{i}^{\mathrm{out}}=\frac{{A}_{i}\times {F}_{i}}{{A}_{{{\rm{C}}}_{2}{{\rm{H}}}_{6}}}\times {n}_{{{\rm{C}}}_{2}{{\rm{H}}}_{6}}^{\mathrm{out}},$$

where ${n}_{{{\rm{C}}}_{2}{{\rm{H}}}_{6}}^{{\rm{out}}}$ is the known flow of C_2_H_6_ at the reactor outlet. Conversion (*X*_i_), selectivity (*S*_*i*_), yield (*Y*_*i*_) and methanol production rate (*r*_MeOH_) were derived using equations (3)–(6):

3 $${X}_{i}=\frac{{n}_{i}^{{\rm{in}}}-{n}_{i}^{{\rm{out}}}}{{n}_{i}^{{\rm{in}}}},$$

4 $${S}_{i}=\frac{{n}_{i}^{{\rm{out}}}}{{n}_{{{\rm{CO}}}_{2}}^{{\rm{in}}}-{n}_{{{\rm{CO}}}_{2}}^{{\rm{out}}}},$$

5 $${Y}_{i}={X}_{{{\rm{CO}}}_{2}}\times {S}_{i}=\frac{{n}_{i}^{{\rm{out}}}}{{n}_{{{\rm{CO}}}_{2}}^{{\rm{in}}}},$$

6 $${r}_{i}=\frac{{n}_{i}^{{\rm{out}}}}{{m}_{{\rm{cat}}}}.$$

The methanol STY is the product of *r*_MeOH_ and the molar weight (MW) of methanol (32.04 g mol^−1^), as detailed in equation (7):

7 $${\rm{S}}{\rm{T}}{\rm{Y}}={r}_{{\rm{MeOH}}}\times {\rm{M}}{{\rm{W}}}_{{\rm{MeOH}}}.$$

The carbon balance was calculated for each experiment as

8 $${\varepsilon }_{{\rm{C}}}=\left(1-\frac{{n}_{{\mathrm{CO}}_{2}}^{\mathrm{out}}+{n}_{\mathrm{MeOH}}^{\mathrm{out}}+{n}_{\mathrm{CO}}^{\mathrm{out}}}{{n}_{{\mathrm{CO}}_{2}}^{\mathrm{in}}+{n}_{\mathrm{CO}}^{\mathrm{in}}}\right)$$

and was always within a 5% difference.

### Computational methods

DFT simulations were conducted using the Vienna ab initio simulation package (VASP, v5.4.4) using the PBE exchange–correlation functional^58–60^. We used a plane-wave basis set with a kinetic energy cut-off of 500 eV to represent the valence electrons, and core electrons were described using projector augmented-wave pseudopotentials^61,62^. The Monkhorst–Pack method was used to generate a Γ-centred mesh with a reciprocal grid size narrower than 0.040 Å^−1^ to sample the Brillouin zone^63^. Spin polarization was included when required.

Lattice parameters of different bulk polymorphs of HfO_2_ and ZrO_2_ were optimized with a higher kinetic cut-off energy of 700 eV, and they are in line with the experimental values (Supplementary Table 18). For HfO_2_, we assessed monoclinic (m), tetragonal (t) and cubic (c) polymorphs corresponding to the space groups $P{2}_{1}/c$, $P{4}_{2}/nmc$ and $Fm\overline{3}m$, respectively (Supplementary Note 1). Analogous polymorphs were investigated for ZrO_2_. The possibility of substituting indium in all polymorphs was explored by replacing one metal atom (Hf or Zr) by In in supercells with dimensions of 2 × 2 × 2 (monoclinic and cubic) and 2 × 2 × 3 (tetragonal). The same supercells were used to assess the oxygen-vacancy formation in both pure supports and indium-containing systems.

We used periodic boundary conditions to represent the most stable surfaces of m-MO_2_ (–111) and t*-*MO_2_ (101) polymorphs (Supplementary Fig. 13 and Supplementary Note 1)^64–66^. Oxygen-terminated slabs were cleaved from optimized bulk structures and represented with *p*(1 × 1) and *p*(1 × 2) expansions for monoclinic and tetragonal polymorphs, respectively (Supplementary Fig. 13). All slabs contained four layers, the two outermost being allowed to relax and the two bottom ones fixed to their bulk positions. For the monoclinic polymorph, each slab layer consisted of 12 atomic layers arranged as O‒O‒O‒Zr‒O‒Zr‒Zr‒O‒Zr‒O‒O‒O, whereas the tetragonal counterpart consisted of five atomic layers with the sequence O‒O‒Zr_2_‒O‒O. Despite their structural differences, both slabs contained four formula units of MO_2_ per layer and maintained the identical ratios of relaxed to fixed atoms. We introduced a vacuum region of 15 Å perpendicular to the slab surface (*z* axis) with a dipole correction along the same direction to compensate for the asymmetry of relaxations^67^. The most favourable surface configurations of isolated indium atoms were assessed using three strategies: (1) by substituting one of the non-equivalent support metal atoms in the two outermost layers of the slab (surface and subsurface), (2) by adsorbing a hydroxylated In(OH)_3_ species on top of pristine m- and t-MO_2_ surfaces and (3) by adsorbing an isolated In adatom (Supplementary Figs. 14 and 19). Additionally, in the case of the adsorption of hydroxylated In species, the surface unit was increased to twice the size with respect to previous systems. Oxygen-vacancy formation was explored in all pristine and In-containing slabs models (Supplementary Fig. 15). Moreover, different amounts of hydrogen, oxygen and indium atoms were explored in systems with adsorbed hydroxylated In species using ab initio thermodynamics to assess their relative stability at different conditions (Supplementary Note 1).

To assess the electronic properties of 2InHfO_*x*_ and 2InZrO_*x*_, we computed the bandgaps of bulk and surface HfO_2_ and ZrO_2_ models, considering oxygen vacancies as well as different types of In doping. The bandgaps obtained using the PBE density functional were benchmarked against the hybrid HSE03 functional with 13% of non-local Hartree–Fock exchange and the meta-generalized gradient approximation r^2^SCAN functional (Supplementary Tables 19–21)^68–70^. The results indicate that PBE provides a good cost–accuracy trade-off and is an appropriate density functional for comparing the catalytic performance of Hf- and Zr-based oxides from a computational standpoint.

Energy profiles for methanol synthesis via CO_2_ hydrogenation and its competitive RWGS reaction were computed at the PBE level using the models containing both incorporated and adsorbed In with one oxygen vacancy (Supplementary Note 3). Gas-phase CO_2_, H_2_ and H_2_O molecules, as well as clean surfaces, were used as thermodynamic sinks. Transition states were located using the climbing image nudged elastic band method (CI-NEB)^71^, and their nature was confirmed by computing numerical frequencies with a step size of ±0.015 Å. Vibrational contributions to enthalpy and entropy were included for the adsorbed species, whereas rotational and translational contributions were also considered for gas-phase molecules only. Gibbs free energies were computed at 553 K in line with experimental reaction CO_2_ hydrogenation conditions.

## Online content

Any methods, additional references, Nature Portfolio reporting summaries, source data, extended data, supplementary information, acknowledgements, peer review information; details of author contributions and competing interests; and statements of data and code availability are available at https://doi.org/10.1038/s41565-026-02135-y.

## Supplementary information

Supplementary Information Supplementary Notes 1–5, Tables 1–21, Figs. 1–74, captions to Supplementary Videos and references.

Peer Review File

Supplementary Video 1 Top-view projections of ab initio MD simulations for pristine In(OH)_3_-m-HfO_2_, for the system with one oxygen vacancy, and for configurations with one oxygen vacancy and adsorbed HCOO or CH_3_O (left to right). Simulations were performed using a temperature ramp from 0 to 553 K over 5 ps in the *NVT* ensemble with a Nosé–Hoover thermostat and a 1-fs time step. Colour code: Hf, purple; O, salmon pink; In, red; C, black; H, white. The indium trajectory indicates that the single atom undergoes only confined diffusion within the first coordination shell of Hf atoms, and the local mobility is further attenuated in the presence of reaction intermediates.

Supplementary Video 2 Top-view projections of ab initio MD simulations for pristine In(OH)_3_-m-ZrO_2_, for the system with one oxygen vacancy, and for configurations with one oxygen vacancy and adsorbed HCOO or CH_3_O (left to right). Simulations were performed using a temperature ramp from 0 to 553 K over 5 ps in the *NVT* ensemble with a Nosé–Hoover thermostat and a 1-fs time step. Colour code: Zr, teal; O, salmon pink; In, red; C, black; H, white. The indium trajectory indicates that the single atom undergoes only confined diffusion within the first coordination shell of Zr atoms, and the local mobility is further attenuated in the presence of reaction intermediates.

Supplementary Video 3 Top-view projections of ab initio MD simulations for pristine In(OH)_3_-t-ZrO_2_, for the system with one oxygen vacancy, and for configurations with one oxygen vacancy and adsorbed HCOO or CH_3_O (left to right). Simulations were performed using a temperature ramp from 0 to 553 K over 5 ps in the *NVT* ensemble with a Nosé–Hoover thermostat and a 1-fs time step. Colour code: Zr, teal; O, salmon pink; In, red; C, black; H, white. The indium trajectory indicates that the single atom undergoes only confined diffusion within the first coordination shell of Zr atoms, and the local mobility stays attenuated in the presence of reaction intermediates.

## Source data

Source Data Fig. 1 Source data for Fig. 1b–d.

Source Data Fig. 2 Source data for Fig. 2a–c.

Source Data Fig. 3 Source data for Fig. 3b.

Source Data Fig. 4 Source data for Fig. 4a,b.

Source Data Fig. 5 Source data for Fig. 5a,b,d–h.

Source Data Fig. 6 Source data for Fig. 6a–c.

Source Data Extended Data Fig. 2 Source data for Extended Data Fig. 2.

Source Data Extended Data Fig. 3 Source data for Extended Data Fig. 3.

Source Data Extended Data Fig. 4 Source data for Extended Data Fig. 4.

Source Data Extended Data Fig. 5 Source data for Extended Data Fig. 5.

Source Data Extended Data Fig. 6 Source data for Extended Data Fig. 6.

Source Data Extended Data Fig. 7 Source data for Extended Data Fig. 7.

Source Data Extended Data Fig. 8 Source data for Extended Data Fig. 8.

## Data Availability

The data presented in the Article are available via Zenodo (https://doi.org/10.5281/zenodo.16881810)^72^. All DFT structures, inputs and outputs can be found online in the ioChem-BD repository^73,74^ at https://doi.org/10.19061/iochem-bd-1-387 (ref. ^75^). Further data supporting the findings of this study are available in the Article and its Supplementary Information. All other relevant source data are available from the corresponding author upon request. Source data are provided with this paper.
