# Supplementary material for: Single atoms of indium on hafnia enable superior CO2-based methanol synthesis
Source: Nat Nanotechnol. 2026 Mar 2;21(4):588–97. doi: 10.1038/s41565-026-02135-y (PMC13652736; doi:10.1038/s41565-026-02135-y)
Supplement: Supplementary file 1 [file 41565_2026_2135_MOESM1_ESM.pdf]

nature nanotechnology

Supplementary information https://doi.org/10.1038/s41565-026-02135-y

Single atoms of indium on hafnia enable superior CO_2-based methanol synthesis

In the format provided by the authors and unedited

Table of Contents

Supplementary Notes 1-5	2
Supplementary Tables 1-21	17
Supplementary Figures 1-74	29
Supplementary Video Captions	86
Supplementary References	87

Supplementary Notes

Supplementary Note 1: DFT modelling approach for In-Hf mixed oxides.

Selection of HfO_2 polymorph. At ambient pressure, HfO_2 crystallizes in three polymorphs; monoclinic (m), tetragonal (t), and cubic (c), which interconvert with temperature. m-HfO_2 is thermodynamically stable at room temperature and transforms into t-HfO_2 followed by c-HfO_2 upon heating. The monoclinic-to-tetragonal and tetragonal-to-cubic transitions occur at ~2000 K and ~2870 K, respectively, and c-HfO_2 melts at 3031 K. Alternatively, for ZrO_2, the monoclinic-to-tetragonal and tetragonal-to-cubic transitions occur at ~1480 K and ~2650 K, respectively, which are significantly lower than the transition temperature of HfO_2[1].

XRD analysis of bulk phases shows that, depending on the preparation method and In_2O_3 content, indium-hafnium mixed oxide catalysts may contain either only m-HfO_2, or a mixture of monoclinic, tetragonal, and cubic polymorphs. Surface-sensitive Raman spectroscopy indicates mainly m-HfO_2, except at high In_2O_3 content, where features shift toward c-HfO_2. Considering that the best-performing catalysts exhibited predominantly or exclusively the monoclinic polymorph, we focused on this phase. However, we also simulated structures based on t/c-HfO_2 for comparison.

Substitution of indium single atoms in the bulk of HfO_2. The crystal symmetry and coordination of HfO_2 polymorphs are characterized as follows:

m-HfO_2 $(P2_1/c)$: $a \neq b \neq c$; $\alpha = \gamma = 90^o \neq \beta$. Hf atoms are 7-fold coordinated, whereas O can have 3- or 4-fold coordination.

t-HfO_2 $(P4_2/nmc)$: $a = b \neq c$; $\alpha = \beta = \gamma = 90^o$. Two distinct Hf−O bond lengths. Hf atoms are 8-fold coordinated, and O atoms are 4-fold coordinated.

c-HfO_2 $(Fm\bar{3}m)$: $a = b = c$; $\alpha = \beta = \gamma = 90^o$. Hf−O bond distances are equivalent throughout, with the same coordination as t-HfO_2.

Supercells (2×2×2 for monoclinic and cubic, 2×2×3 for tetragonal) were constructed to match experimental indium contents for 2 wt% In_2O_3, which showed the best performance. Since each polymorph contains only a single type of metal cation, lattice substitution of indium was examined by replacing a Hf atom with an In atom (**Supplementary Fig. 8**) and was found to be energetically favourable in the monoclinic lattice.

Oxygen vacancy formation was studied for the pristine and In-containing bulk models. For m-HfO_2, vacancies were introduced at both 3-fold and 4-fold coordinated O sites, whereas only one symmetry unique O site exists in t-HfO_2 and c-HfO_2. For In-containing systems, all non-equivalent O atoms in the first coordination sphere were assessed (seven for m-HfO_2, two for t-HfO_2, and one for c-HfO_2. The results (**Supplementary Fig. 8**) show that the presence of oxygen vacancies makes the stabilization of lattice-substituted indium atoms more favourable within the bulk, particularly in the cubic polymorph, and that oxygen vacancy formation is favoured when indium atoms are present.

Analysis of indium speciation. AC-STEM images confirm the atomic dispersion of InO_x species, likely as indium single atoms. Since isolated indium sites have rarely been explored within the active ensembles of In_2O_3-based catalysts for CO_2 hydrogenation, density functional theory (DFT) simulations at the PPB level were performed on indium species adsorbed to the surface of the HfO_2 support in ligand-free or oxyhydroxylated form. The formation of neighbouring oxygen vacancies was also studied in each case. Gibbs free energy profiles for CO_2 hydrogenation to methanol and the competing reverse water-gas shift (RWGS) reaction were calculated.

Stabilization of isolated In sites at the surface and subsurface of HfO_2. To evaluate the stabilization of In single atoms by HfO_2, we constructed slab models by cleaving the optimized bulk lattices of the monoclinic and tetragonal phases (**Supplementary Fig. 13**). The most stable oxygen-terminated surfaces were selected: (–111) for m-HfO_2 and (101) for t-HfO_2,

respectively. The slabs were constructed with $p(1\times1)$ and $p(1\times2)$ surface unit cells, respectively, with an identical number of layers, formula units, and ratio of relaxed-to-fixed atoms[2].

Three types of In single-atom species were assessed. Substitution models involved replacing a surface or subsurface Hf atom with an In atom. Adsorption models considered the deposition of an isolated In atom on top of the surface oxygen atoms. Finally, hydroxylated adsorption models involved the deposition of $In(OH)_3$ species on the same surface, in which case surface unit cells twice the size of those mentioned were used to prevent the interaction between adsorbed species.

Each configuration was studied in the presence and absence of a neighbouring oxygen vacancy. In the substitution model for m-HfO_2 (–111), the slab contains eight non-equivalent Hf sites: five bulk-like 7-fold coordination (four subsurface, one surface) and three 6-fold-coordinated surface atoms (**Supplementary Fig. 14**). In t-MO_2 (101), the slab contains four Hf atoms in each of the surface and subsurface layers; the surface Hf atoms are 7-fold coordinated, while the subsurface atoms are 8-fold coordinated, as in the bulk. Non-equivalency arises from distinct M−O bond lengths. Substitution of In atoms is generally more favourable at surface or subsurface sites than in the bulk of the support (**Supplementary Fig. 16**), and lower incorporation energies are observed for the monoclinic (bulk: 2.45 eV, surface: 1.49 eV) than the tetragonal (bulk: 2.62 eV, surface: 1.58 eV) phase. The adsorption of isolated In atoms is endothermic, leading to physisorption. In contrast, the adsorption of $In(OH)_3$ adatoms is consistently exothermic, with oxygen atoms acting as anchoring sites on both monoclinic and tetragonal HfO_2 surfaces (**Supplementary Fig. 20**).

Oxygen vacancy formation was examined for pristine (m-/t-)HfO_2 slabs, for lattice-substituted In single atoms, and for adsorbed $In(OH)_3$ species, which are detailed in **Supplementary Figs. 14**, **17**, and **21**, respectively. For the pristine systems, vacancy formation was simulated at all non-equivalent oxygens. In m-HfO_2(–111), 16 non-equivalent oxygen sites were identified (8 subsurface, 8 surface), mainly 3- or 4-fold coordinated, with one 2-fold

coordinated surface site. In t-MO_2(101), eight non-equivalent oxygens were found (four subsurface, four surface), with each layer containing two 3-fold and two 4-fold (bulk-like) coordinated oxygens. Substituting a single Hf atom with In (**Supplementary Fig. 14**) and testing each non-equivalent O site revealed that oxygen vacancies stabilize surface In incorporation, particularly in the tetragonal polymorph (**Supplementary Fig. 17**). In addition to the lattice O sites, in $In(OH)_3$-HfO_2 systems, three potential vacant sites from hydroxyl groups were examined. The most favourable oxygen vacancy is located directly above (on top) the adsorbed In atom (**Fig. 3, Supplementary Fig. 21**).

Energetics and stability of the InHfO$_x$ models. The relative stability of pristine m-, t-, and c-HfO_2 polymorphs was evaluated by calculating their formation energies (E_f) at the PBE level, as defined in **Supplementary Equations 1** and **2**. Reference energies were taken from DFT simulations by formula unit of each HfO_2 polymorph ($E^{DFT}_{HfO_2,bulk}$), bulk hafnium ($E^{DFT}_{Hf,bulk}$), and molecular oxygen ($E^{DFT}_{O_2,gas}$):

$$Hf + O_2 \longrightarrow HfO_2 \quad (1)$$

$$E_f = E^{DFT}_{HfO_2,\,bulk} - E^{DFT}_{Hf,\,bulk} - E^{DFT}_{O_2,\,bulk} \quad (2)$$

PBE-computed potential energies (E) of the bulk and slab HfO_2 systems were then determined (**Supplementary Equations 3** and **4**) to assess the relative stability of models with incorporated or adsorbed In /$In(OH)_3$ species, with and without oxygen vacancies. The reference energies for these calculations were molecular H_2 ($E^{DFT}_{H_2,gas}$), H_2O ($E^{DFT}_{H_2O,gas}$), bulk In metal ($E^{DFT}_{In,bulk}$), and pristine bulk supercell or slab (Hf_xO_y) of each polymorph. Here, v denotes the number of oxygen vacancies and s the number of incorporated or the number of adsorbed In atoms:

$$Hf_xO_y + (s+a)In + (-2s - \frac{3a}{2} + v) + H_2 \longrightarrow In_{s+a}(OH)_{3a}Hf_{x\text{-}s}O_{y\text{-}v} + sHfO_2 + (-2s - 3a + v)H_2O$$

(3)

$$E = E^{\mathrm{DFT}}_{\mathrm{In}_{s+a}\mathrm{(OH)}_{3a}\mathrm{Hf}_{x-s}\mathrm{O}_{y-v}} + sE^{\mathrm{DFT}}_{\mathrm{HfO_2,bulk}} + (-2s-3a+v)E^{\mathrm{DFT}}_{\mathrm{H_2O,g}} - E^{\mathrm{DFT}}_{\mathrm{Hf}_x\mathrm{O}_y} - (s+a)E^{\mathrm{DFT}}_{\mathrm{In,bulk}} - (-2s-\frac{3a}{2}+v)E^{\mathrm{DFT}}_{\mathrm{H_2,g}}$$

(4)

The oxygen vacancy formation energy (E_{vac}) was obtained using **Supplementary Equation 5** to identify the most stable vacancy site and assess the likelihood of vacancy formation:

$$E_{\mathrm{vac}} = E^{\mathrm{DFT}}_{\mathrm{In}_{s+a}\mathrm{(OH)}_{3a}\mathrm{Hf}_{x-s}\mathrm{O}_{y-v-1}} + E^{\mathrm{DFT}}_{\mathrm{H_2O,g}} - E^{\mathrm{DFT}}_{\mathrm{In}_{s+a}\mathrm{(OH)}_{3a}\mathrm{Hf}_{x-s}\mathrm{O}_{y-v}} - E^{\mathrm{DFT}}_{\mathrm{H_2,g}} \quad (5)$$

PBE-computed segregation energies (E_{seg}) of each polymorph were calculated to quantify the tendency of In atoms to migrate from bulk to subsurface or surface sites (**Supplementary Equation 6**):

$$E_{\mathrm{seg}} = E_{\mathrm{In@subsurface/surface}} + E_{\mathrm{In@bulk}} \quad (6)$$

Finally, PBE-computed aggregation energies indicate the tendency of $In(OH)_3$ species to form dimers on the surface (**Supplementary Equation 7**):

$$E_{\mathrm{agg}} = 2E_{\mathrm{In(OH)_3@surface}} - E_{\mathrm{2(In(OH)_3)@surface}} - E_{\mathrm{surface}} \quad (7)$$

Simulation of In-Zr mixed oxides. Since atomically dispersed In atoms have not previously been modelled as active sites in CO_2 hydrogenation to methanol, for comparison, an identical set of simulations was conducted for ZrO_2 by replacing Hf with Zr[3,4]. ZrO_2 undergoes the same polymorphic sequence as HfO_2, but the m-t transition occurs at a lower temperature (~1480 K) and the t-c transition at ~2650 K. XRD analyses indicate the bulk crystalline phase consists either of m-ZrO_2, or a mixture of m- and t-ZrO_2. A small fraction of t-ZrO_2 is also detectable at the surface, but it decreases during CO_2 hydrogenation.

Ab initio thermodynamics. Grand canonical free energies (Ω_{In}) were evaluated using ab initio thermodynamics to determine the most stable surface configurations as a function of

temperature and H_2 partial pressure[5]. The examined set included adsorbed $In(OH)_3$ adatoms that were progressively stripped of H and/or O atoms down to bare In, as well as the corresponding dimer $2In(OH)_3$ and oxygen-vacancy containing analogues. These species were referenced either to bulk In (**Supplementary Equation 8**) or gas-phase $In(OH)_3$ (**Supplementary Equation 9**) to ensure that the relative stabilities of different configurations do not depend on the selection of chemical potential reference. All grand canonical free energies were normalized per indium atom. The reference energies for these simulations correspond to the total PBE-computed energies of the slab system (E_{slab}) and the bare surface (E_{bare}). The expressions used to compute Ω_{In} are:

$$\Omega_{In} = \frac{E_{slab} - E_{bare} - n_H \cdot \mu_H - n_O \cdot \mu_O - n_{In,bulk} \cdot \mu_{In,bulk}}{n_{In,bulk}} \quad (8)$$

or

$$\Omega_{In} = \frac{E_{slab} - E_{bare} - m_H \cdot \mu_H - m_O \cdot \mu_O - m_{In(OH)_3,g} \cdot \mu_{In(OH)_3,g}}{m_{In(OH)_3,g}} \quad (9)$$

The chemical potentials of H, O, and $In(OH)_3$ were obtained using **Supplementary Equations 10-12**[6]. Enthalpies (ΔH), zero-point vibrational energies (ZPVE, $E_{H_2}^{vib}(T=0K)$) and entropies (S) were taken from NIST thermochemical database when available[7]; otherwise, they were computed at the relevant temperature using Gaussian 09[8]. Electronic energies for isolated molecules were employed from DFT-PBE simulations. The reaction conditions were chosen according to **Supplementary Fig. 2** for $2InHfO_x$ (T = 553 K, P = 50 bar, and a $CO_2:H_2$ = 1:4 with 5% CO_2 conversion), which were used to approximate the partial pressures of the gas molecules.

$$\mu_H = \frac{1}{2}\mu_{H_2} = \frac{1}{2}\left(\Delta H_{H_2,g}(T,p^0) + E_{H_2,g}^{vib}(T=0K) + E_{H_2,g} - TS_{H_2,g}(T,p^0) + kT \cdot \ln(\frac{p_{H_2,g}}{p^0})\right) \quad (10)$$

$$\mu_{\mathrm{O}}=\mu_{\mathrm{H_2O}}-\mu_{\mathrm{H_2}}=\left(\Delta H_{\mathrm{H_2O},g}(T,p^0)+E^{vib}_{\mathrm{H_2O},g}(T=0K)+E_{\mathrm{H_2O},g}-TS_{\mathrm{H_2O},g}(T,p^0)+kT\cdot\ln(\frac{p_{\mathrm{H_2O},g}}{p^0})\right)$$
$$-\left(\Delta H_{\mathrm{H_2,g}}(T,p^0)+E^{vib}_{\mathrm{H_2O},g}(T=0K)+E_{\mathrm{H_2,g}}-TS_{\mathrm{H_2,g}}(T,p^0)+kT\cdot\ln(\frac{p_{\mathrm{H_2},g}}{p^0})\right) \quad (11)$$

$$\mu_{\mathrm{In(OH)_3,g}}=\Delta H_{\mathrm{In(OH)_3},g}(T,p^0)+E^{vib}_{\mathrm{In(OH)_3},g}(T=0K)+E_{\mathrm{In(OH)_3},g}-TS_{\mathrm{In(OH)_3},g}(T,p^0)+kT\cdot\ln(\frac{p_{\mathrm{In(OH)_3},g}}{p^0}) \quad (12)$$

The chemical potential of indium was approximated by the electronic energy of bulk In (**Supplementary Equation 13**).

$$\mu_{\mathrm{In,bulk}}=E_{\mathrm{In,bulk}} \quad (13)$$

Supplementary Note 2: Electronic structure and oxygen vacancy dynamics.

Oxygen vacancy density is a key descriptor in In_2O_3-based catalysts for CO_2 hydrogenation to methanol[9,10], and depends on the synthesis methods and history of thermal or chemical treatments of the catalyst. For binary catalytic systems prepared by flame spray pyrolysis, formation of intrinsic defects is favoured owing to the rapid quenching rate and presence of heterovalent dopants. Fundamentally, oxygen vacancy generation is associated with the removal of (sub)surface lattice oxygen, releasing two electrons that may be trapped at the vacancy site and adjacent M^{4+} ion (M = Hf, Zr). This process traps a single electron in the oxygen vacancy ($V_O^{\bullet}$, g_{iso} = 2.004) and forms an M^{3+} ion (g_{xx} = g_{yy} = 1.98, g_{zz} = 1.96). While both signals are observed in the EPR profile of fresh 2InZrO$_x$, only the former is recorded in fresh 2InHfO$_x$ catalyst (**Fig. 5b**, **Supplementary Figs. 38-39**). This difference points to distinct electronic structures of the vacancy and neighbouring M^{4+} ion. Formation of the $V_O^{\bullet}$-M^{3+} structure is thermodynamically preferred in m-ZrO_2, whereas the vacancy with two electrons ($V_O^{\bullet\bullet}$-M^{4+}) is more stable in m-HfO_2. Still, the presence of a $V_O^{\bullet}$ signal in 2InHfO$_x$ indicates that some electrons are trapped at isolated defective sites. Under controlled operating conditions (He, H_2, CO_2, and CO_2+H_2), the $V_O^{\bullet}$ signal in 2InHfO$_x$ remains virtually unchanged, indicating that it is likely a spectator species. In contrast, $V_O^{\bullet\bullet}$ is likely the more relevant fingerprint for

active sites, though it remains silent in electron paramagnetic resonance (EPR) since the vacancy contains paired electrons.

For 2InZrO$_x$, thermal treatment in He reoxidises Zr^{3+} to Zr^{4+}, attributed to the transfer of trapped electrons from M sites to thermally-induced oxygen vacancies[5,6]. Exposure to CO_2 heals the isolated oxygen vacancies, leaving only stable superoxides (O^{2-}, $g_{zz} = 2.035$, $g_{yy} = 2.01$, $g_{xx} = 2.002$) on the surface. Upon H_2 treatment, additional vacant sites form via reduction, and the released lattice oxygens combine with surface electrons to yield superoxide species. Finally, introducing the reaction mixture ($CO_2 + H_2$) removes the superoxide signal and increases the oxygen vacancy density, as evidenced by the higher $V_O^{\bullet}$ signal and a more intense ferromagnetic signal (V_O-f, ~5 G) from strongly coupled oxygen vacancies.

Supplementary Note 3: DFT reaction energy profiles and molecular dynamics simulations

We computed reaction energy profiles at the PBE level for CO_2 hydrogenation to methanol via the formate pathway, along with the competitive RWGS path yielding CO (**Fig. 6a, Supplementary Figs. 46-48**). Simulations were performed on m-HfO_2, m-ZrO_2, and t-ZrO_2, as the experimentally observed surfaces. In each case, we considered adsorbed $In(OH)_3$ species with one oxygen vacancy on top of the adsorbed In atom, as the most representative models of the 2InHfO$_x$ and 2InZrO$_x$ catalysts (see **Supplementary Note 1**).

On $In(OH)_3$-m-HfO_2 and $In(OH)_3$-m-ZrO_2, CO_2 adsorption occurs via carbonate formation; one oxygen atom of CO_2 binds to the adsorbed In atom, while the carbon bonds to an oxygen from a hydroxide ligand. On $In(OH)_3$-t-ZrO_2, in contrast, CO_2 binds at a lattice oxygen site adjacent to the indium atom. H_2 activation proceeds heterolytically, generating an M−H/OH pair on the surface. In the methanol pathway, the hydride is transferred to the carbon of the adsorbed CO_2 species, producing formate (CHO_2). A second heterolytic H_2 splitting precedes two additional hydrogenation steps: a hydride transfer to the carbon forming CH_2O_2,

followed by proton transfer to the oxygen, yielding formic acid (CH_2OOH). Dehydration of CH_2OOH produces CH_2O and H_2O. A third heterolytic H_2 activation and hydride/proton transfers lead to methoxy (CH_3O) formation and finally methanol. In the RWGS route, the initial intermediate is carboxylate (COOH) rather than formate. Its dehydration yields CO and H_2O.

DFT-PBE simulations were also employed to evaluate reactant adsorption. For the most representative models of the $2InHfO_x$ and $2InHfO_x$ catalysts, the adsorption of dissociated H_2O and CO_2 is predicted to be exothermic, ranging from –0.72 to –1.30 eV $H_2O_{molec}^{-1}$and from –0.25 to –0.95 eV CO_{2molec}^{-1} at high and low coverages, respectively. Consistent with these calculations, in-situ DRIFTS analysis of $2InHfO_x$ and $2InZrO_x$ reveals two bands characteristic of adsorbed CO_2 (**Supplementary Figs. 54-55**), assigned to asymmetric and symmetric stretching C–O models, in excellent agreement with DFT-derived vibrational frequencies (**Supplementary Table 10**). Notably, the presence of these spectator species does not significantly perturb CO_2 hydrogenation reaction energy profiles (**Supplementary Figs. 57-58**). Except for the $In(OH)_3$-t-ZrO_2 model, which is a minor phase, we observe a detrimental contribution for activation of the reagents.

We further evaluated the stability of the single-atom sites by conducting 5 ps of PBE ab initio molecular dynamics (AIMD) simulations with a temperature ramp from 0 K to 553 K over $In(OH)_3$-m-HfO_2, $In(OH)_3$-m-ZrO_2, and $In(OH)_3$-t-ZrO_2 models (**Supplementary Videos 1-3**). The trajectories of the In single atom during CO_2 hydrogenation indicate that it diffuses within a small region defined by the first coordination shell of Hf/Zr atoms of the support, which is partially mitigated by the presence of the reaction intermediates (**Supplementary Fig. 51**). Thus, the In single atom remains spatially confined and its local environment is preserved, in line with the long-term stability test (**Supplementary Fig. 3**), in-situ XAS and EPR measurements (**Fig. 5a,b**), and ex-situ STEM-EDX analyses (**Fig. 2**). We also assessed the trajectories of In single atoms along the intermediates involved in the CO_2

hydrogenation (**Supplementary Fig. 53**) and obtained the same conclusions as those derived from MD simulations.

Supplementary Note 4: Kinetic modelling and performance predictions.

Notation. A microkinetic model developed previously[10-12] was adapted and fitted to experimental data for CO_2 hydrogenation over the $2InHfO_x$ and $2InZrO_x$ catalysts. Experimental conditions varied across *T*, *P*, *GHSV*, and H_2/CO_2 ratio. Over 100 catalytic tests were conducted to obtain a robust dataset for kinetic model fitting. The full experimental data is available through the Zenodo repository (doi:10.5281/zenodo.16881810).

Two reaction pathways were considered consistent with mechanistic insights: Methanol formation from CO_2 (denoted subscript 'MeOH') and CO formation through the reverse water-gas shift reaction (denoted subscript 'RWGS'). Surface reactions were modelled using a two-site Langmuir-Hinshelwood-Hougen-Watson (LHHW) mechanism. The following notations are used:

p_i	partial pressure of compound *i*
r_n	reaction rate of elementary step *n*
k_n, k_{-n}	forward and backward rate coefficients for elementary step *n*
θ_i	surface coverage of compound *i*
$K_i = \frac{k_{\text{ads},i}}{k_{\text{des},i}} = \frac{\theta_i}{p_i}$	adsorption equilibrium for compound *i*
θ_*	fraction of free adsorption sites for CO_2, CO, HCOO, CH_3O, CH_3OH
$\theta_\#$	fraction of free adsorption sites for H and H_2O

Rate expressions. In the two-site model, gaseous CO_2, CO, and CH_3OH competitively adsorb on site *, where carbon-containing intermediates such as formate (HCOO), methoxy (CH_3O)

also adsorb. Meanwhile, H_2O adsorption and dissociative H_2 adsorption occur on site #. The elementary steps considered for MeOH formation are:

(1) k_1, k_{-1} $CO_2^* + H^\# \rightleftharpoons HCOO^* + \#$

(2) k_2, k_{-2} $HCOO^* + H^\# \rightleftharpoons H_2COO^* + \#$

(3) k_3, k_{-3} $H_2COO^* + H^\# \rightleftharpoons H_2COOH^* + \#$

(4) k_4, k_{-4} $H_2COOH^* + H^\# \rightleftharpoons CH_2O^* + H_2O^\#$

(5) k_5, k_{-5} $CH_2O^* + H^\# \rightleftharpoons CH_3O^* + \#$

(6) k_6, k_{-6} $CH_3O^* + H^\# \rightleftharpoons CH_3OH^* + \#$

For CO formation via RWGS, the elementary steps considered are:

(7) k_7, k_{-7} $CO_2^* + H^\# \rightleftharpoons COOH^* + \#$

(8) k_8, k_{-8} $COOH^* + H^\# \rightleftharpoons CO^* + H_2O^\#$

The rate-determining steps are assumed as follows: For MeOH formation, the combination of steps (4) and (5) leads to the rate expressions shown in **Equations 14** and **15**:

$$r_{\mathrm{MeOH}} = k_4 k_5 \theta_{\mathrm{H_2COOH}} \theta_{\mathrm{H}}^2 - k_{-4} k_{-5} \theta_{\mathrm{CH_3O}} \theta_{\mathrm{H_2O}} \theta_{\#} \quad (14)$$

$$= k_{\mathrm{A}} \frac{\theta_{\#}^2 \theta_{*}}{\sqrt{p_{\mathrm{H_2}}}} \left(p_{\mathrm{CO_2}} p_{\mathrm{H_2}}^3 - \frac{p_{\mathrm{CH_3OH}} p_{\mathrm{H_2O}}}{K_{\mathrm{eq,MeOH}}} \right) \quad (15)$$

where k_A denotes the reaction rate coefficient for methanol formation. For CO formation, step (8) is rate-determining resulting in the rate expressions given in **Equations 16** and **17**:

$$r_{\mathrm{RWGS}} = k_8 \theta_{\mathrm{COOH}} \theta_{\mathrm{H}} - k_{-8} \theta_{\mathrm{H_2O}} \theta_{\mathrm{CO}} \quad (16)$$

$$= k_{\mathrm{B}} \theta_{\#} \theta_{*} \left(p_{\mathrm{CO_2}} p_{\mathrm{H_2}} - \frac{p_{\mathrm{H_2O}} p_{\mathrm{CO}}}{K_{\mathrm{eq,RWGS}}} \right) \quad (17)$$

where k_B denotes the reaction rate coefficient for CO formation.

Site balance and free site fraction. In addition to adsorbed CO_2, CO, and CH_3OH, operando DRIFTs experiments (**Fig. 6b,c**, **Extended Fig. 6**, **Supplementary Figs. 54-55**) confirmed the presence of formate (HCOO) and methoxy (CH_3O) intermediates, which are included explicitly in the site balance. The free adsorption site fractions on sites * and # are given by the site balance **Equations 18** and **19**:

$$\theta_* = \left(1 + K_{CO_2} p_{CO_2} + K_{CO} p_{CO} + K_{CH_3OH} p_{CH_3OH} \right.$$

$$\left. + K_1 K_{CO_2} p_{CO_2} \sqrt{K_{H_2} p_{H_2}} + \frac{K_6 K_{CH_3OH} p_{CH_3OH}}{\sqrt{K_{H_2} p_{H_2}}} \right)^{-1} \tag{18}$$

$$\theta_\# = \frac{1}{1 + \sqrt{K_{H_2} p_{H_2}} + K_{H_2O} p_{H_2O}} \tag{19}$$

Temperature dependence of rate constants. Overall rate constants for both reactions follow Arrhenius behaviour, referenced to T_{ref} = 573 K, as shown in **Equations 20** and **21**:

$$k_{MeOH}(T) = k_{MeOH} \exp\left(\frac{E_{a,MeOH}}{R} \left(\frac{1}{T_{ref}} - \frac{1}{T} \right) \right) \tag{20}$$

$$k_{RWGS}(T) = k_{RWGS} \exp\left(\frac{E_{a,RWGS}}{R} \left(\frac{1}{T_{ref}} - \frac{1}{T} \right) \right) \tag{21}$$

where $E_{a,MeOH}$ and $E_{a,RWGS}$ are the respective activation energies, R = 8.314×10^{-3} kJ mol^{-1} K^{-1}, and k_{MeOH} or k_{RWGS} are the pre-exponential factors. Detailed derivations of these expressions are available in our previous work[6].

Parameter fitting. Predicted molar flows, $F_{i,\mathrm{predicted}}$, for species i were calculated by numerically integrating the relevant plug flow reactor (PFR) design equations using the experimental conditions (*T*, *P*, and inlet flow rates, $F_{i,\mathrm{inlet}}$, derived from the H_2:CO_2 feed ratio and *GHSV*). The fitting minimized the objective function defined in **Equation 22**, representing the normalized sum of squared differences between predicted and experimental outlet molar flows of CH_3OH and CO:

$$\varepsilon = \sum_{n=1}^{n_{\text{total}}} \left(\frac{\left(F_{\text{CH}_3\text{OH,predicted}} - F_{\text{CH}_3\text{OH,measured}}\right)^2 + \left(F_{\text{CO,predicted}} - F_{\text{CO,measured}}\right)^2}{F_{\text{CH}_3\text{OH,predicted}} + F_{\text{CO,predicted}}} \right)_n \tag{22}$$

Fitted kinetic parameters are reported in **Supplementary Tables 11-12**. The mean absolute percentage error (*MAPE*) between predicted and measured catalytic performance metrics, q, including X_{CO2}, S_{MeOH}, Y_{MeOH}, or STY_{MeOH}, as defined in **Equation 23**:

$$MAPE_q = \frac{1}{N_{\text{total}}} \sum_{N=1}^{N_{\text{total}}} \left(\frac{\left| q_{\text{predicted}} - q_{\text{measured}} \right|}{q_{\text{predicted}}} \right)_N \tag{23}$$

Supporting Note 5: Translation of the HfO_2 promotional effect to reducible oxides.

Extending the HfO_2 support beyond In_2O_3 demonstrates that the promotional effect is transferable to Ga_2O_3 and ZnO-based catalysts, resulting in more than two orders of magnitude higher methanol productivity compared with the bulk (unsupported) oxides (**Extended Fig. 8**). To establish the principles underpinning this remarkable behaviour, we conducted extensive characterizations.

Dual-site mechanism of metal centre and HfO_2 support. CO_2 chemisorption, CO_2-temperature-programmed desorption (CO_2-TPD), and H_2-D_2 temperature-programmed exchange (H_2-D_2-TPE) measurements were performed to identify the respective roles of metal centres and the HfO_2 support. Relative to the pristine oxides (HfO_2, In_2O_3, Ga_2O_3, and ZnO), all HfO_2-supported catalysts (InHfO$_x$, GaHfO$_x$, and ZnHfO$_x$) exhibit increased CO_2 uptake (**Supplementary Table 15**), indicating that HfO_2 promotes CO_2 activation, which is supported by the CO_2-TPD profiles of GaHfO$_x$ and ZnHfO$_x$ resembling that of HfO_2 (**Supplementary Fig. 63**). Additionally, H_2-D_2-TPE analysis shows that GaHfO$_x$ and ZnHfO$_x$ exchange temperatures are like their respective bulk oxides (**Supplementary Fig. 64**), and HAADF-STEM measurement indicate the absence of nanoclusters (**Supplementary Fig. 65**). Collectively, these results show that Ga and Zn single atoms in these systems remain active for

H_2 dissociation, supporting a dual-site mechanism between the active metals (In, Ga, or Zn), which split H_2, and the support (HfO_2), which activates CO_2 and stabilizes surface hydride-proton pairs. Similar to $InHfO_x$, XPS and in-situ XAS analyses show that both Ga and Zn remain oxidic and stable under CO_2 hydrogenation conditions, highlighting the strong electronic metal-support interaction (**Supplementary Figs. 66-68**).

Metal-dependent promotional effect. While HfO_2 promotes all three active metals, the exceptional methanol productivity of $InHfO_x$ suggests an additional metal-specific synergistic interaction. In-situ DRIFTS analysis of $InHfO_x$, $GaHfO_x$, and $ZnHfO_x$, conducted under equivalent CO_2 hydrogenation conditions, reveals that all catalysts show characteristic stretching frequencies of formate and methoxy species (**Supplementary Figs. 69-71**), consistent with the formate pathway of CO_2-based methanol synthesis in all systems. Nonetheless, quantitative analysis of the intermediate kinetics reveals that surface formate concentrations reach steady state after ~60 min for $InHfO_x$. Comparatively, formate increases continuously for $GaHfO_x$ and $ZnHfO_x$. After switching to hydrogenation conditions by replacing CO_2 with He, the surface methoxy species reach maxima at ~180 min for $InHfO_x$ and $GaHfO_x$, while they keep increasing for $ZnHfO_x$. These trends are consistent with superior CO_2 activation (fast formate turnover) and facilitated hydrogenation on $InHfO_x$. Indeed, XPS analysis reveals that $InHfO_x$ exhibits a higher density of oxygen vacancy after reaction, while fewer oxygen vacancies are detected in the equilibrated $GaHfO_x$ and $ZnHfO_x$ catalysts (**Supplementary Fig. 72**).

To complement our experimental findings, DFT-PBE simulations were employed to further rationalize the metal speciation and oxygen vacancy formation in $GaHfO_x$ and $ZnHfO_x$ systems. Since Raman and STEM-EDX analyses indicate the formation of Ga and Zn single atoms on a pure monoclinic surface, we have considered the surface incorporation of Ga and Zn, as well as the adsorption of the $Ga(OH)_3$ or $Zn(OH)_2$ species over the m-HfO_2 surface (**Supplementary Fig. 73** and **Supplementary Table 15**). Analogous to $InHfO_x$ systems,

adsorption of Ga- and Zn-hydroxylated species is favoured over surface incorporation. Interestingly, the vacancy formation energy is exothermic in $In(OH)_3$-m-HfO_2 (E_{vac} = –0.58 eV) while is endothermic for $Ga(OH)_3$-m-HfO_2 and $In(OH)_3$-m-HfO_2 models (E_{vac} = 0.86 and 1.02 eV, respectively), in agreement with the XPS results.

Supplementary Tables

Supplementary Table 1. Summary of a Materials Project database search for experimentally reported monoclinic metal oxides with band gaps exceeding 3.5 eV[13].

ID[a]	Formula	Energy above Hull[b] (eV atom^{-1})	Band gap (eV)
mp-2858	ZrO_2	0	3.53
mp-352	HfO_2	0	4.02
mp-1582	As_2O_3	0	4.03
mp-1581	As_2O_3	<0.01	3.63
mp-1194715	As_2O_3	<0.01	3.62
mp-669426	SiO_2	<0.01	5.54
mp-556654	SiO_2	0.01	5.61
mp-1200292	SiO_2	0.02	5.70

[a] Unique Materials Project identifier. [b] Stability metric, where 0 eV atom^{-1} indicates thermodynamic stability.

Supplementary Table 2. Overview of synthesis, nomenclature, composition, and textural properties of mixed binary $InMO_x$ catalysts (M = Hf, Zr).

Catalyst code	Synthesis route[a]	In_2O_3[b] (wt%)	In_2O_3[c] (wt%)	S_{BET}[d] ($m^2 g^{-1}$)	d_{MOx}[e] (nm)
$1InHfO_x$	FSP	1	0.95	53	13.2
$2InHfO_x$	FSP	2	1.93	51	14.3
$3InHfO_x$	FSP	3	3.10	60	13.2
$5InHfO_x$	FSP	5	5.06	57	17.2
$7InHfO_x$	FSP	7	7.11	52	14.3
$10InHfO_x$	FSP	10	9.89	61	14.3
In_2O_3/HfO_2(P),WI	WI of HfO_2(P)	2	2.21	75	6.4
$InHfO_x$,CP	CP	2	2.14	80	10.7
In_2O_3/HfO_2(FSP),WI	WI of HfO_2(FSP)	2	1.98	69	12.3
HfO_2(P)	P	-	-	81	12.3
HfO_2(FSP)	FSP	-	-	63	13.2
$1InZrO_x$	FSP	1	1.13	102	10.7
$2InZrO_x$	FSP	2	2.04	96	13.2
$3InZrO_x$	FSP	3	3.01	91	9.5
$5InZrO_x$	FSP	5	4.99	95	12.3
$7InZrO_x$	FSP	7	7.19	100	11.5
$10InZrO_x$	FSP	10	9.98	110	12.3
ZrO_2	FSP	-	-	96	12.4
$GaHfO_x$	FSP	2	1.83	58	10.9
$ZnHfO_x$	FSP	2	1.51	55	13.4

[a] Notation: P - precipitation; FSP - flame spray pyrolysis; CP - coprecipitation. [b] Targeted. [c] Determined by ICP-OES. [d] Determined by the BET method. [e] Determined from the Scherrer equation applied to the m-ZrO_2(11−1) or m-HfO_2(11−1) reflections.

Supplementary Table 3. Catalytic performance metrics of the $InHfO_x$ and $InZrO_x$ catalysts in CO_2 hydrogenation.

Catalyst[a]	X_{CO2}[b] (%)	S_{MeOH}[c] (%)	STY_{MeOH}[d] (g_{MeOH} h^{-1} g_{In}^{-1})	STY_{MeOH}[d] (g_{MeOH} h^{-1} g_{cat}^{-1})	STY_{MeOH}[d] (mg_{MeOH} h^{-1} m_{cat}^{-2})
$1InHfO_x$	3.1	31	6.16	0.06	0.95
$2InHfO_x$	4.9	57	9.31	0.15	3.02
$3InHfO_x$	5.8	55	7.69	0.19	3.18
$5InHfO_x$	5.6	83	6.72	0.28	4.87
$7InHfO_x$	7.3	70	5.27	0.31	2.87
$10InHfO_x$	6.8	70	3.37	0.28	4.57
In_2O_3/HfO_2(P),WI	1.4	1	0.00	0.00	0.00
$InHfO_x$,CP	1.6	3	0.00	0.00	0.00
In_2O_3/HfO_2(FSP),WI	3.7	17	2.52	0.04	0.97
HfO_2(FSP)	2.7	1	0.00	0.00	0.00
HfO_2(P)	0.0	0	0.00	0.00	0.00
In_2O_3	5.8	46	0.20	0.17	2.07
$1InZrO_x$	2.4	28	5.51	0.05	0.45
$2InZrO_x$	3.2	44	5.56	0.09	0.91
$3InZrO_x$	4.5	43	5.16	0.13	1.41
$5InZrO_x$	5.7	62	5.14	0.21	2.13
$7InZrO_x$	5.4	64	3.93	0.22	2.28
$10InZrO_x$	6.6	58	3.07	0.25	2.65
ZrO_2	2.9	0	0.00	0.00	0.00

[a] Catalyst overview in **Supplementary Table 1**. [b] CO_2 conversion. [c] Methanol selectivity. [d] Methanol space-time yield normalized per gram of indium, gram of catalyst, or specific surface area, respectively. Reaction conditions: $T = 553$ K, $P = 50$ bar, $CO_2:H_2 = 1:4$, $GHSV = 24'000$ cm^3 h^{-1} g_{cat}^{-1}.

Supplementary Table 4. Comparative CO_2 hydrogenation performance of $InHfO_x$ and benchmarked supported In_2O_3 catalysts evaluated under similar conditions.

Catalyst	In_2O_3 (wt%)	T (K)	$GHSV$ ($cm^3\ h^{-1}\ g_{cat}^{-1}$)	X_{CO2} (%)	S_{MeOH} (%)	STY_{MeOH} ($g_{MeOH}\ h^{-1}\ g_{In}^{-1}$)
$InHfO_x$[a]	2	553	24’000	4.9	57	9.31
$InZrO_x$[a]	2	553	24’000	3.2	44	5.56
In_2O_3/ZrO_2[b,14]	7	573	20’000	8.6	65	7.76
In_2O_3/t-ZrO_2[15]	6	543	24’000	6.8	78	6.97
In_2O_3-ZrO_2[c,16]	10	573	18’000	7.5	86	4.81
In_2O_3/m-ZrO_2[17]	5	553	24’000	4.5	84	4.44
In_2O_3/ZrO_2[18]	2.5	553	24’000	2.5	60	4.21
In_2O_3/ZrO_2[19]	9	553	16’000	5.2	99	3.96
In_2O_3/ZrO_2[20]	15	533	48’000	n.a.	91	3.93
In_2O_3/ZrO_2[18]	2.5	553	24’000	2.5	60	3.46
In_2O_3/m-ZrO_2[21]	5	533	48’000	2.9	91	2.75
In_2O_3/ZrO_2[d,22]	13	553	12’000	7.9	74	1.58
In_2O_3/ZrO_2[18]	5	553	24’000	1.8	68	1.29
In_2O_3/Al_2O_3[17]	5	553	24’000	0.4	82	0.97
In_2O_3/t-ZrO_2[17]	5	553	24’000	0.5	82	0.65
In_2O_3/CeO_2[17]	5	553	24’000	0.4	82	0.49
In_2O_3/TiO_2[23]	5	553	24’000	6	2	0.24
In_2O_3/CeO_2[22]	13	553	12’000	0.21	80	0.07
In_2O_3[15]	100	543	24’000	3.5	81	0.22
In_2O_3[17]	100	553	24’000	3.9	84	0.22
In_2O_3[18]	100	553	24’000	2.2	61	0.21

[a] This work. $H_2/CO_2 = 4$, except for [b] and [c], which are equal to 3. $P = 50$ bar, except for [d], which is equal to 30 bar.

Supplementary Table 5. PBE-computed formation energies (E_f) associated with monoclinic, tetragonal, and cubic HfO_2 and ZrO_2 polymorphs.

Crystal system	Space group	$E_{f,ZrO2}$ (eV f.u.$^{-1}$)	$E_{f,HfO2}$ (eV f.u.$^{-1}$)
monoclinic	$P2_1/c$	–10.39	–10.73
tetragonal	$P4_2/nmc$	–10.28	–10.56
cubic	$Fm\bar{3}m$	–10.19	–10.46

Supplementary Table 6. Surface composition of selected InHfO$_x$ and InZrO$_x$ catalysts.

Catalyst[a]	Form	In/M[b] (at%)	O/M[b] (at%)
HfO_2(P)	fresh	-	2.04
HfO_2(FSP)	fresh	-	1.73
2InHfO$_x$	fresh	0.09	1.83
2InHfO$_x$	equilibrated[c]	0.09	1.73
2InZrO$_x$	fresh	0.06	1.68
2InZrO$_x$	equilibrated	0.06	1.57

[a] Catalyst overview in **Supplementary Table 1**.
[b] Determined by XPS. [c] After 20 h of CO_2 hydrogenation. Reaction conditions in **Supplementary Table 3**.

Supplementary Table 7. PBE-computed adsorption energies (E_{ads}) of monomeric and dimeric In(OH)$_3$ species, and aggregation energy (E_{agg}) for monomer-to-dimer conversion.

Surface	E_{ads} (eV)		E_{agg} (eV)
	In(OH)$_3$ monomer	In(OH)$_3$ dimer	
m-HfO_2	–1.50	–1.04	1.97
m-ZrO_2	–1.30	–0.84	1.76

Supplementary Table 8. Electron paramagnetic resonance (EPR) spectrometer settings.

Experiment (-)	*T* (K)	Microwave frequency (GHz)	Central field (G)	Sweep width (G)	Modulation amplitude[a] (G)	Conversion time (ms)	Time constant (ms)
ex-situ	298	9.4	4050	8000	5	81.92	40.96
in-situ	298	9.2	3000	5900	3	84.46	40.96
in-situ	553	9.2	3000	5900	3	84.46	40.96

[a] Modulation frequency (kHz) and power attenuation (dB) were set to 100 kHz and 20 dB.

Supplementary Table 9. Relative amount of EPR active species in $2InZrO_x$ under distinct working conditions detected via in-situ EPR experiments. The data was fitted using the particle swarm optimization method (See **Method**).

Conditions	$V_O^{\bullet}$[a] (%)	Zr^{3+}[a] (%)	O^{2-} (%)
fresh	60	40	0
He	100	0	0
H_2	8	0	92
CO_2	0	0	100
CO_2+H_2	100	0	0

[a] Oxygen vacancy with single electron trapped ($V_O^{\bullet}$) and corresponding reduced metal cation Zr^{3+} upon vacancy generation. See **Supplementary Note 2**.

Supplementary Table 10. PBE-computed and experimental frequencies of asymmetric (v_1) and symmetric stretching (v_2) C-O mode of adsorbed $*CO_3^{2-}$.

Catalytic system	Vibrational mode	Frequency / cm^{-1}	
		Computational value	Experimental value
$In(OH)_3$-m-HfO_2	$v_1(*CO_3^{2-})$	1542	1600
	$v_2(*CO_3^{2-})$	1290	1385
$In(OH)_3$-m-ZrO_2	$v_1(*CO_3^{2-})$	1526	1596
	$v_2(*CO_3^{2-})$	1322	1391
$In(OH)_3$-t-ZrO_2	$v_1(*CO_3^{2-})$	1539	1596
	$v_2(*CO_3^{2-})$	1288	1391

Supplementary Table 11. Methanol adsorption energies (E_{ads}) over hydroxylated In single atoms anchored on monoclinic and tetragonal HfO_2 or ZrO_2 surfaces computed with PBE.

Model	E_{ads} (eV)
$In(OH)_3$-m-HfO_2	–1.04
$In(OH)_3$-m-ZrO_2	–1.11
$In(OH)_3$-t-ZrO_2	–1.20

Supplementary Table 12. H_2 chemisorption of HfO_2 or ZrO_2 supports prepared by flame spray pyrolysis (FSP).

Support	H_2 uptake (mm^3 m^{-2})
HfO_2(FSP)	0.81
ZrO_2(FSP)	0.47

Supplementary Table 13. Rate expressions and thermodynamic parameters of the two-site LHHW kinetic model describing methanol (MeOH) and CO (RWGS) formation from CO_2 over the 2InHfO_x and 2InZrO_x catalysts (see **Supplementary Note 4**).

Parameter	Expression	Unit
r_{MeOH}	$k_{\mathrm{MeOH}} \frac{\theta_{\#}^{2}\theta_{*}}{\sqrt{p_{\mathrm{H_2}}}}\left(p_{\mathrm{CO_2}}p_{\mathrm{H_2}}^{3}-\frac{p_{\mathrm{CH_3OH}}p_{\mathrm{H_2O}}}{K_{\mathrm{eq,MeOH}}}\right)$	mol s^{-1} kg$_{\mathrm{cat}}$$^{-1}$
r_{RWGS}	$k_{\mathrm{RWGS}}\theta_{\#}\theta_{*}\left(p_{\mathrm{CO_2}}p_{\mathrm{H_2}}-\frac{p_{\mathrm{H_2O}}p_{\mathrm{CO}}}{K_{\mathrm{eq,RWGS}}}\right)$	mol s^{-1} kg$_{\mathrm{cat}}$$^{-1}$
k_{MeOH}	$k_{\mathrm{MeOH,ref}}\exp\left(\frac{E_{\mathrm{a,MeOH}}}{R}\left(\frac{1}{T_{\mathrm{ref}}}-\frac{1}{T}\right)\right)$	mol s^{-1} bar^{-4} kg$_{\mathrm{cat}}$$^{-1}$
k_{RWGS}	$k_{\mathrm{RWGS,ref}}\exp\left(\frac{E_{\mathrm{a,RWGS}}}{R}\left(\frac{1}{T_{\mathrm{ref}}}-\frac{1}{T}\right)\right)$	mol s^{-1} bar^{-2} kg$_{\mathrm{cat}}$$^{-1}$
θ_{*}	$\left(1+K_{\mathrm{CO_2}}p_{\mathrm{CO_2}}+K_{\mathrm{CO}}p_{\mathrm{CO}}+K_{\mathrm{CH_3OH}}p_{\mathrm{CH_3OH}} +K_{1}K_{\mathrm{CO_2}}p_{\mathrm{CO_2}}\sqrt{K_{\mathrm{H_2}}p_{\mathrm{H_2}}}+\frac{K_{6}K_{\mathrm{CH_3OH}}p_{\mathrm{CH_3OH}}}{\sqrt{K_{\mathrm{H_2}}p_{\mathrm{H_2}}}}\right)^{-1}$	-
$\theta_{\#}$	$\left(1+\sqrt{K_{\mathrm{H_2}}p_{\mathrm{H_2}}}+K_{\mathrm{H_2O}}p_{\mathrm{H_2O}}\right)^{-1}$	-
$K_{\mathrm{eq,MeOH}}$	$\exp\left(39.85+\frac{3.17\times10^{3}}{T}-9.52\ln(T) +56.77\times10^{-4}T-57.5\times10^{-8}T^{2}+\frac{64.95\times10^{3}}{T^{2}}\right)$	bar^{-2}
$K_{\mathrm{eq,RWGS}}$	$\exp\left(18.01-\frac{5.87\times10^{3}}{T}-1.86\ln(T)+2.70\times10^{-4}T+\frac{58.2\times10^{3}}{T^{2}}\right)$	-

Supplementary Table 14. Fitted parameters of the two-site LHHW kinetic model for the 2InHfO$_x$ and 2InZrO$_x$ catalysts.

Parameter	2InHfO$_x$	2InZrO$_x$	Unit
$K_{\mathrm{MeOH,ref}}$	6.01×10^{-5}	3.08×10^{-5}	mol s^{-1} bar^{-4} kg$_{\mathrm{cat}}^{-1}$
$k_{\mathrm{RWGS,ref}}$	1.39×10^{-3}	7.71×10^{-4}	mol s^{-1} bar^{-2} kg$_{\mathrm{cat}}^{-1}$
$E_{\mathrm{a,MeOH}}$	82	87	kJ mol^{-1}
$E_{\mathrm{a,RWGS}}$	124	96	kJ mol^{-1}
K_1	0.269	0.346	-
K_6	0.315	1.07	-
K_{H2}	3.08	3.28	bar^{-1}
K_{CO2}	0.316	0.168	bar^{-1}
K_{CO}	0.090	15.2	bar^{-1}
K_{CH3OH}	16.3	8.02	bar^{-1}
K_{H2O}	3.14	2.71	bar^{-1}

Supplementary Table 15. CO_2 adsorption capacity of HfO_2, *M*O$_x$, and *M*HfO$_x$ materials prepared by FSP (*M* = In, Ga, Zn), showing the increased CO_2 uptake in *M*HfO$_x$ catalysts.

Catalysts code	CO_2 uptake[a] (m^3 mm^{-2})
HfO_2	1.30
In_2O_3	1.22
Ga_2O_3	0.00
ZnO	3.40
InHfO$_x$	5.65
GaHfO$_x$	6.41
ZnHfO$_x$	3.58

[a] Determined by volumetric CO_2 chemisorption measurement at 573 K.

Supplementary Table 16. DFT-derived speciation analysis and vacancy formation energy of $MHfO_x$ systems (M = In, Ga or Zn) obtained at PBE level.

Model	Energy	Catalytic system		
		$In/In(OH)_3$	$Ga/Ga(OH)_3$	$Zn/Zn(OH)_2$
substitution	E_{sub} (eV)	1.49	1.38	3.35
vacancy	E (eV)	0.50	0.63	–0.05
adsorption	E_{ads} (eV)	–1.50	–2.54	–1.43
vacancy	E_{vac} (eV)	–0.58	0.86	1.17
	E (eV)	–2.09	–1.68	–0.26

Supplementary Table 17. Summary of chemicals used for catalyst synthesis.

Catalyst[a]	Precursor[b]	Supplier	Purity
HfO_2(FSP)	$Hf(OnBu)_4$	ABCR	95%
$InHfO_x$	In, $Hf(OnBu)_4$	Polymet, ABCR	100%, 95%
HfO_2(P)	$HfCl_4$	Strem	99%
In_2O_3/HfO_2(P),WI	$In(NO)_3$, $HfCl_4$	ABCR, Strem	100%, 99%
$InHfO_x$(CP)	$In(NO)_3$, $HfCl_4$	ABCR, Strem	100%, 99%
In_2O_3/HfO_2(FSP),WI	$In(NO)_3$, $Hf(OnBu)_4$	ABCR, ABCR	100%, 95%
ZrO_2(FSP)	$Zr(OEt)_4$	Polymet, ABCR	97%
$InZrO_x$	In, $Zr(OEt)_4$	Polymet, ACROS	100%, 97%
$GaHfO_x$	Ga, $Hf(OnBu)_4$	Polymet, ABCR	100%, 95%
$ZnHfO_x$	$Zn(EHA)_2$, $Hf(OnBu)_4$	Polymet, ABCR	97%, 95%

[a] Catalyst overview in **Supplementary Table 2**. [b] Metal precursors in the desired molar ratio were dissolved in 2-ethylhexanoic acid and tetrahydrofuran (1/3 mass content).

Supplementary Table 18. PBE-computed and experimentally measured[24,25] lattice parameters for m-HfO_2 and m-ZrO_2.

System	Lattice constant	DFT	Experimental
m-HfO_2	*a* (Å)	5.14	5.15
	b (Å)	5.19	5.21
	c (Å)	5.32	5.31
	β (°)	99.67	99.23
m-ZrO_2	*a* (Å)	5.24	5.15
	b (Å)	5.26	5.21
	c (Å)	5.33	5.32
	β (°)	99.23	99.30

Supplementary Table 19. Band-gap values of bulk and surface models using PBE, r^2SCAN, and HSE-13 functionals for m-HfO_2 models compared with experimental results.

	Model/ catalyst	E_g (eV)			
		PBE	r^2SCAN	HSE	UV-visible
bulk	m-HfO_2	4.02	4.52	4.67	-
	m-$InHfO_x$	0.74	1.15	1.12	-
surface	m-HfO_2	2.59	2.98	3.12	-
	In@m-HfO_2	0.83	0.82	1.02	-
	$In(OH)_3$-m-HfO_2	2.42	2.73	2.73	-
fresh catalyst	HfO_2(FSP)	-	-	-	5.45
	$InHfO_x$(FSP)	-	-	-	5.10

Supplementary Table 20. Band-gap values of bulk and surface models using PBE, r^{2}SCAN, and HSE-13 functionals for m-ZrO_2 models compared with experimental results.

	Model/ catalyst	E_g (eV)			
		PBE	r^{2}SCAN	HSE	UV-visible
bulk	m-ZrO_2	3.62	4.10	4.27	-
	m-$InZrO_x$	0.69	1.07	1.04	-
surface	m-ZrO_2	2.55	2.90	3.09	-
	In@m-ZrO_2	1.00	0.85	1.19	-
	$In(OH)_3$-m-ZrO_2	2.26	2.53	2.58	-
fresh catalyst	ZrO_2(FSP)	-	-	-	4.95
	$InZrO_x$(FSP)	-	-	-	4.90

Supplementary Table 21. Band-gap values of bulk and surface models using PBE, r^{2}SCAN, and HSE-13 functionals for t-ZrO_2 models compared with experimental results.

	Model/ catalyst	E_g (eV)			
		PBE	r^{2}SCAN	HSE	UV-visible
bulk	t-ZrO_2	3.95	4.48	4.64	-
	t-$InZrO_x$	0.52	0.94	0.91	-
surface	t-ZrO_2	3.32	3.76	3.95	-
	In@t-ZrO_2	0.39	0.92	0.79	-
	$In(OH)_3$-t-ZrO_2	2.71	3.00	3.06	-
fresh catalyst	ZrO_2(FSP)	-	-	-	4.95
	$InZrO_x$(FSP)	-	-	-	4.90

Supplementary Figures

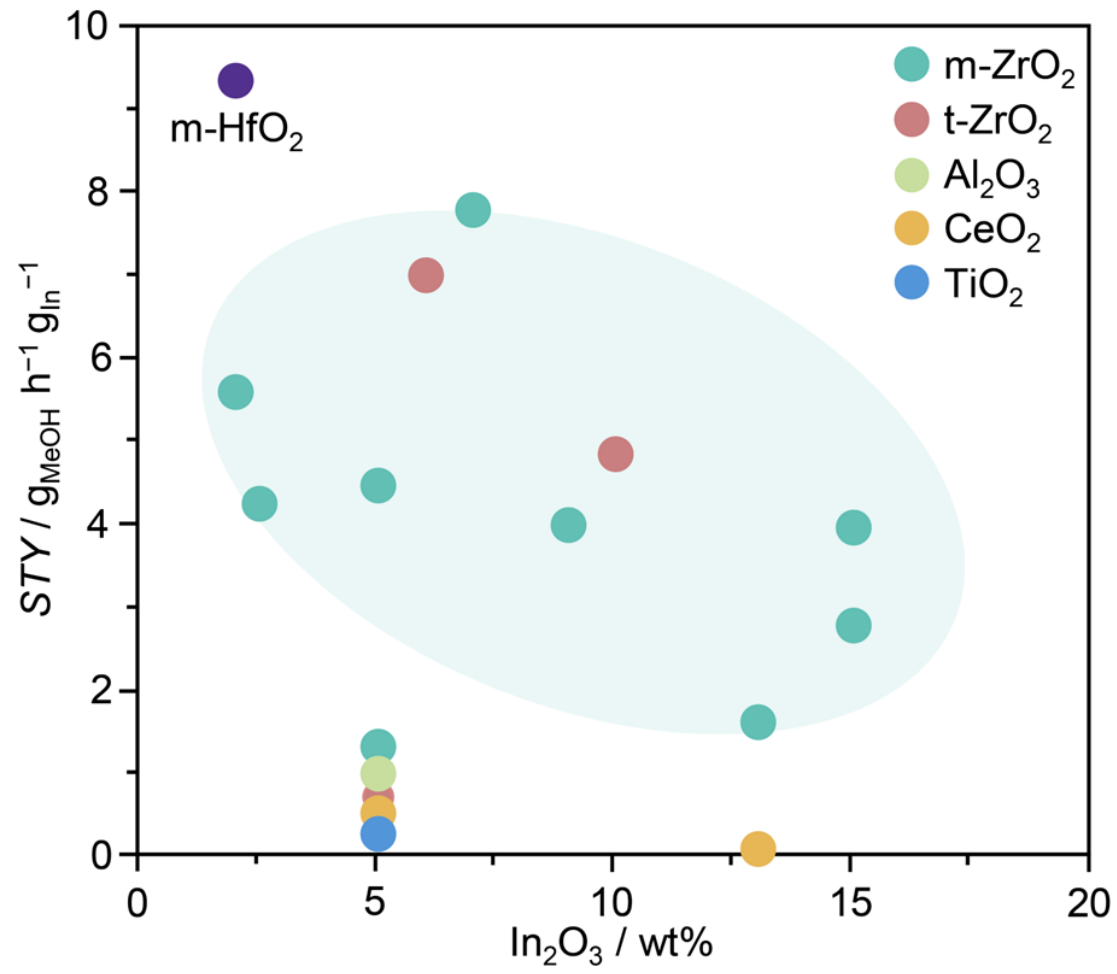

Supplementary Fig. 1. Methanol space-time yield (*STY*) per gram of indium of InHfO$_x$ and reported supported In_2O_3 catalysts with distinct In_2O_3 contents evaluated under similar conditions (**Supplementary Table 4**), underscoring that m-HfO_2 is the first oxide carrier surpassing benchmarked ZrO_2-supported system. The substantially reduced metal oxide content required for m-HfO_2 support highlights the exceptional atom efficiency characteristic of the single-atom catalyst.

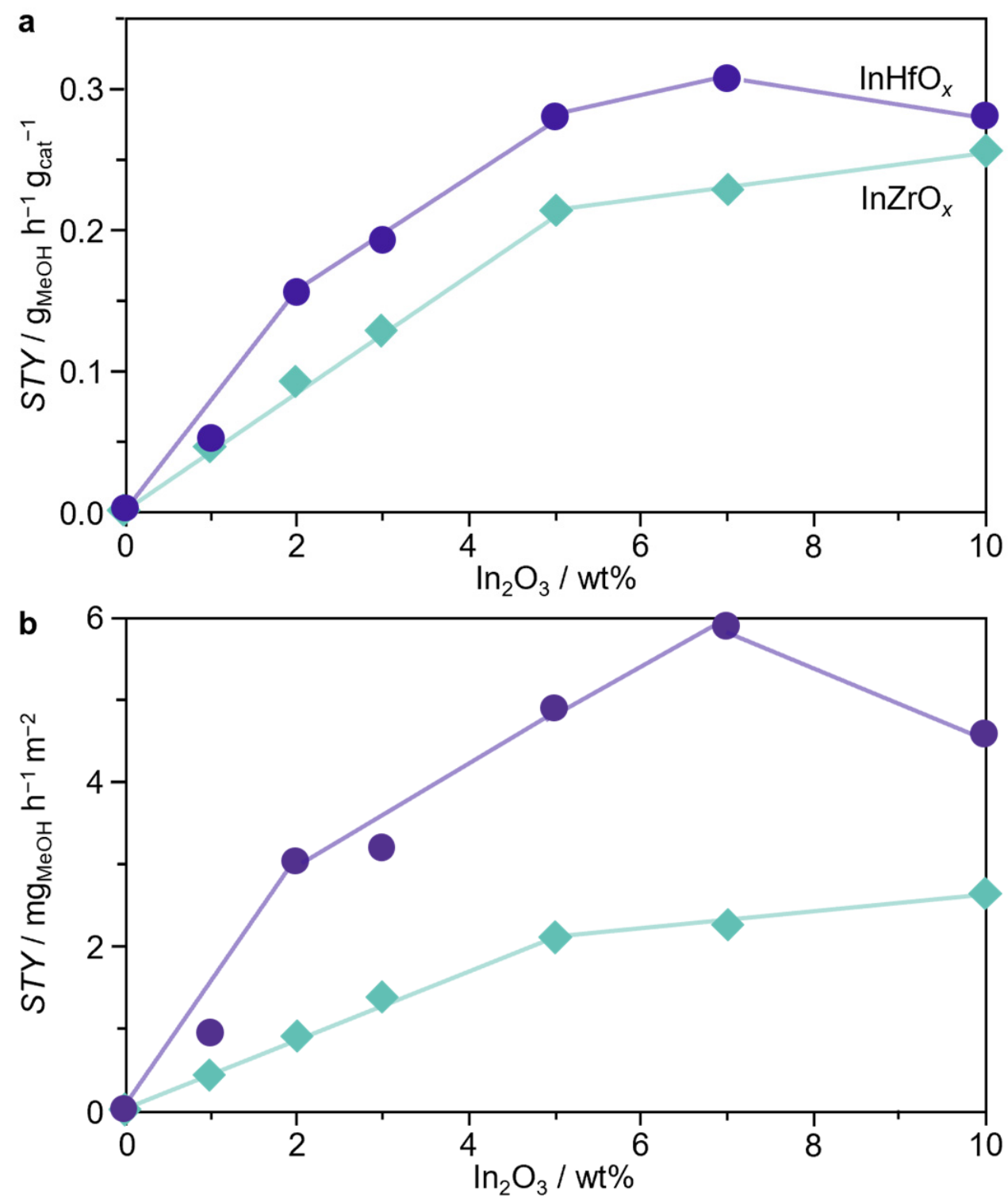

Supplementary Fig. 2. Methanol space-time yield (*STY*) of $InHfO_x$ and $InZrO_x$ catalysts with varying In_2O_3 content, normalized per **a**, gram of catalysts or **b**, specific surface area. Reaction conditions: $T = 553$ K, $P = 50$ bar, $H_2/CO_2 = 4$, $GHSV = 24{,}000$ cm^3 h^{-1} g_{cat}^{-1}, *tos* = 20 h.

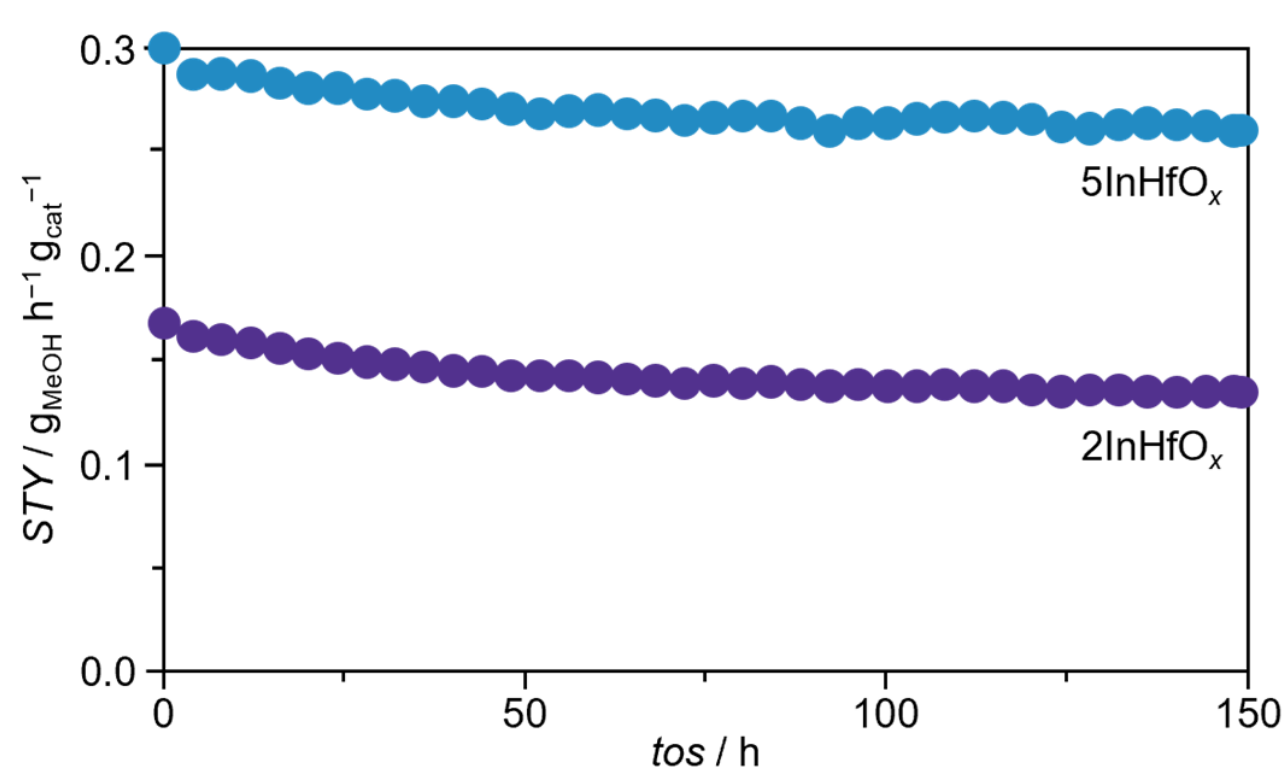

Supplementary Fig. 3. Methanol *STY* versus time on stream (*tos*) over selected $InHfO_x$ catalysts. Reaction conditions: $T = 553$ K, $P = 50$ bar, $H_2/CO_2 = 4$, $GHSV = 24{,}000$ cm^3 h^{-1} g_{cat}^{-1}, *tos* = 150 h.

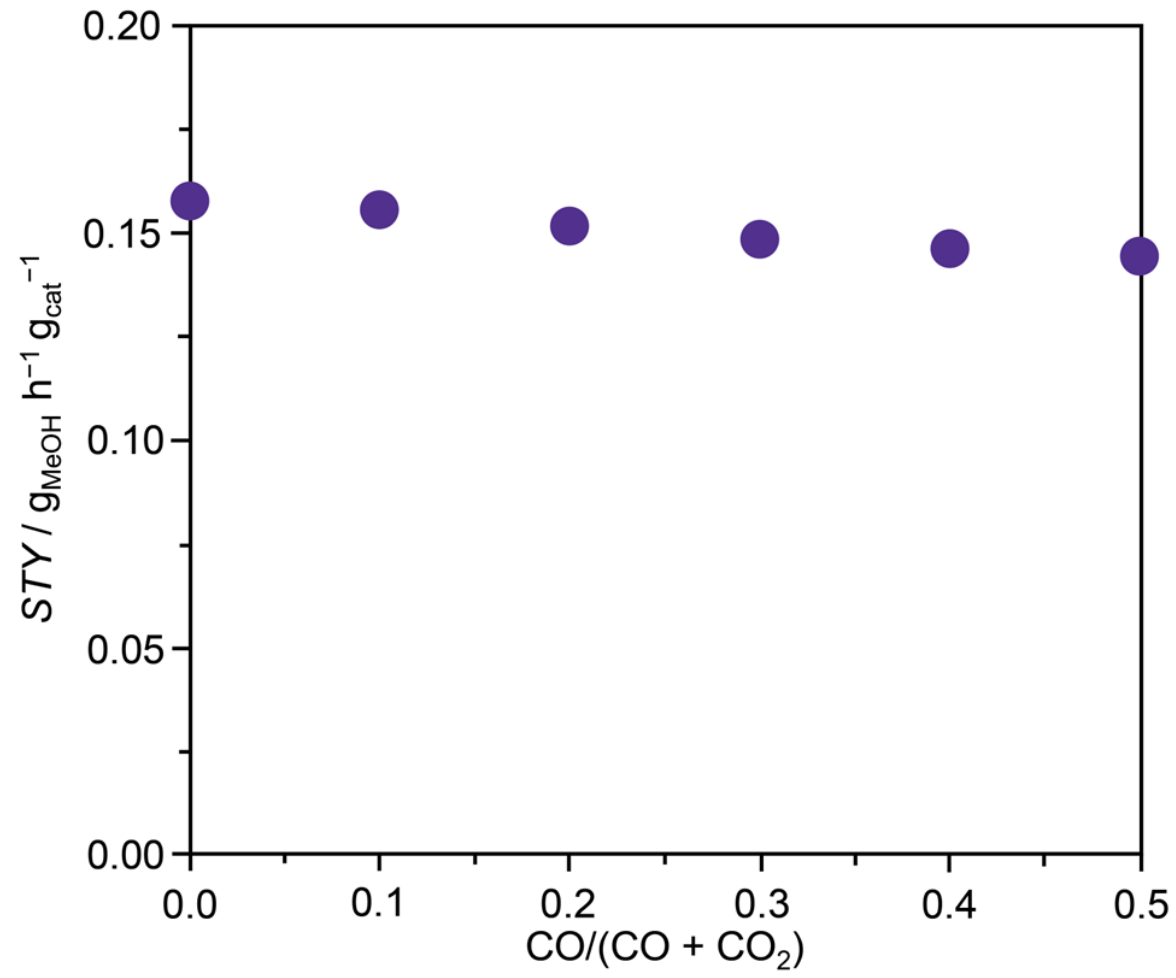

Supplementary Fig. 4. Methanol *STY* over 2InHfO_x catalyst during CO_x hydrogenation, where CO_2 in the feed was gradually replaced by CO stepwise until CO/(CO+CO_2) = 0.5. Reaction conditions: $T = 553$ K, $P = 50$ bar, $H_2/CO_2 = 4$, $GHSV = 24{,}000$ cm^3 h^{-1} g_{cat}^{-1}, $tos = 4$ h for each session. The unchanged methanol productivity indicates the high catalyst stability under practical CO-rich conditions.

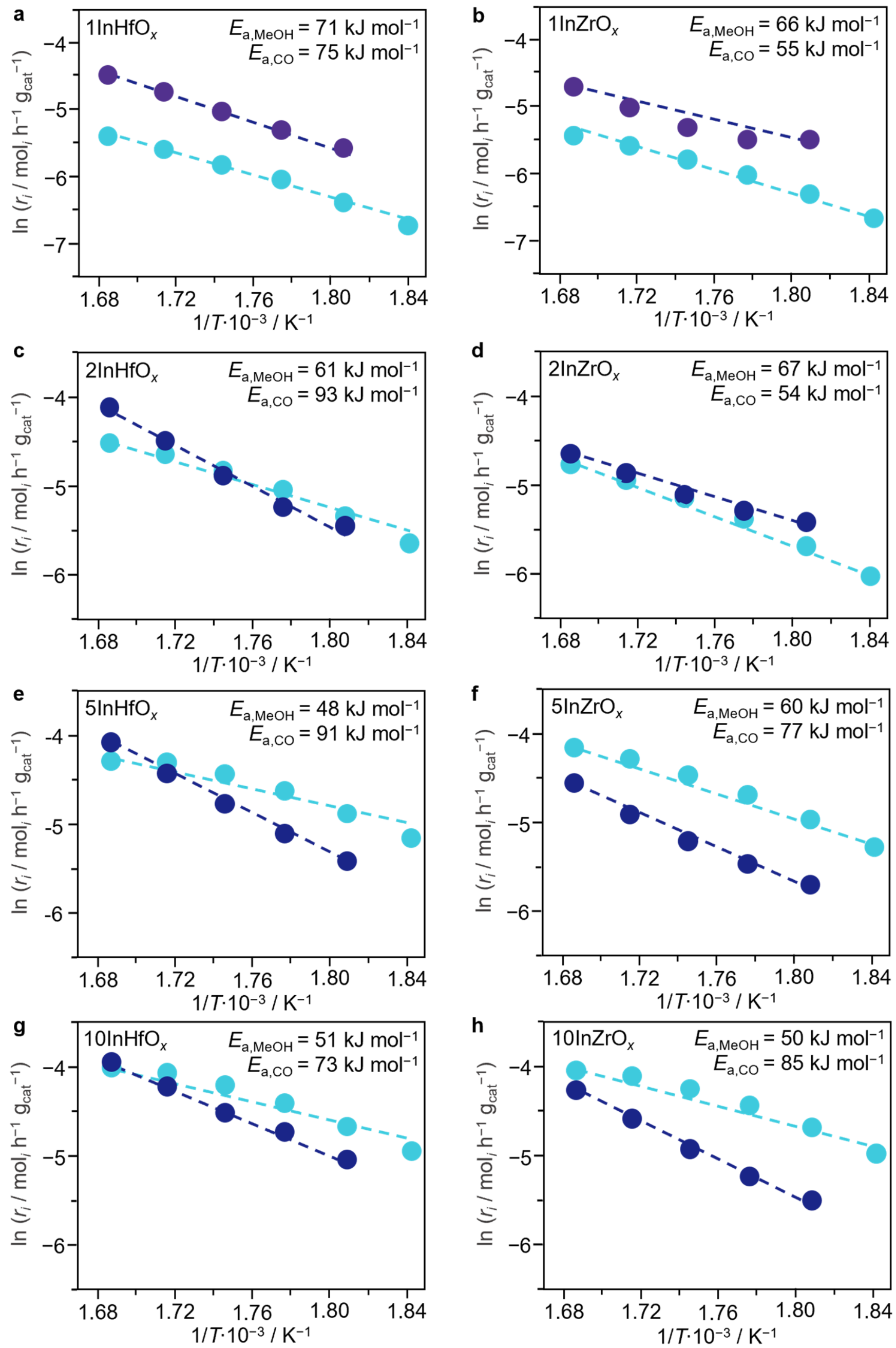

Supplementary Fig. 5. Arrhenius plots used to determine the apparent activation energies for CO_2 hydrogenation to methanol and to CO (RWGS) over selected $InHfO_x$ and $InZrO_x$ catalysts. Reaction conditions: $T = 533\text{-}593$ K, $P = 50$ bar, $H_2/CO_2 = 4$, and $GHSV = 48{,}000$ cm^3 h^{-1} g$_{cat}^{-1}$.

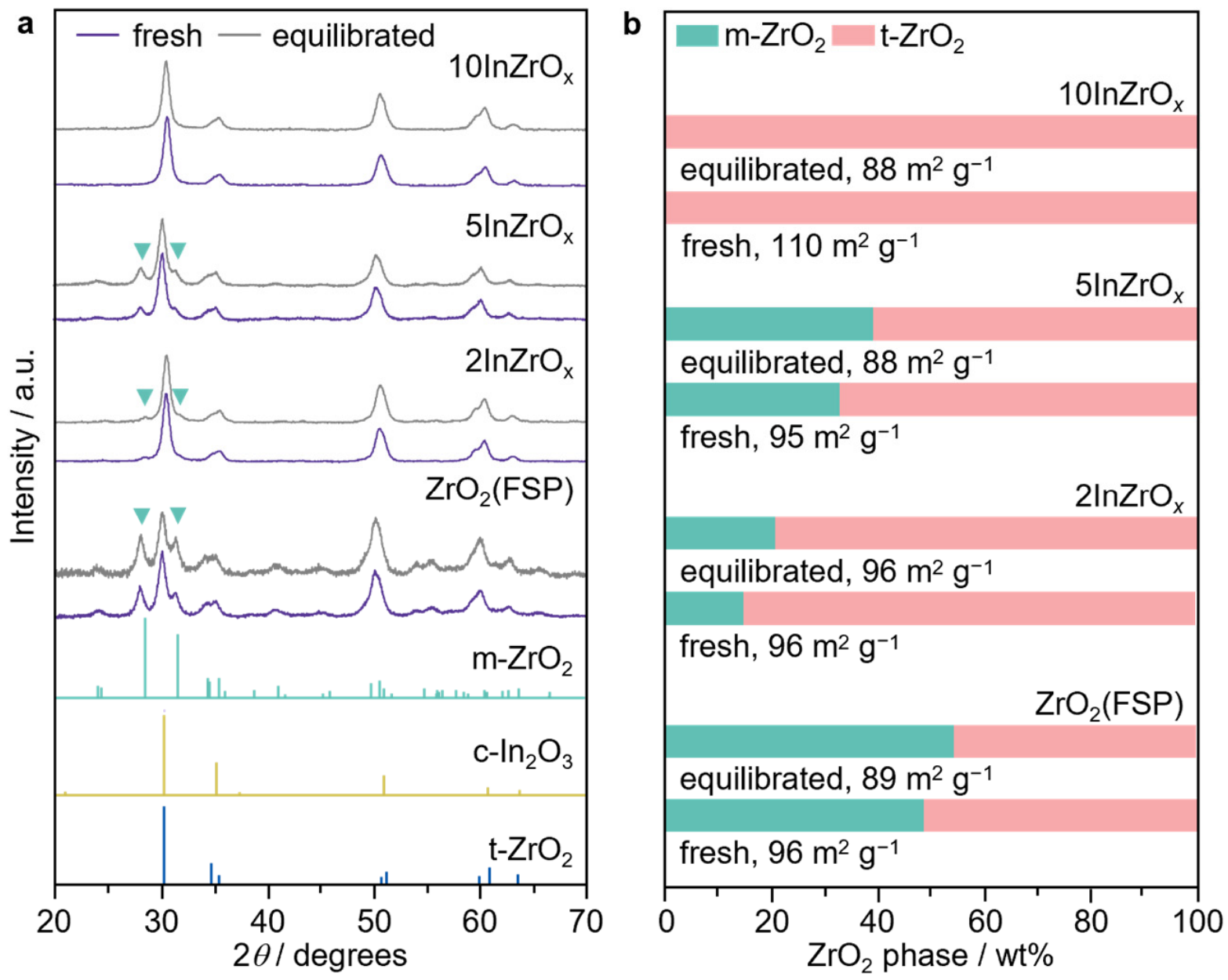

Supplementary Fig. 6. a, XRD patterns of $InZrO_x$ catalysts in fresh (purple lines) and equilibrated (grey lines) forms. **b**, Corresponding phase composition and surface areas of the catalysts. Reaction conditions as indicated in **Supplementary Fig. 2.**

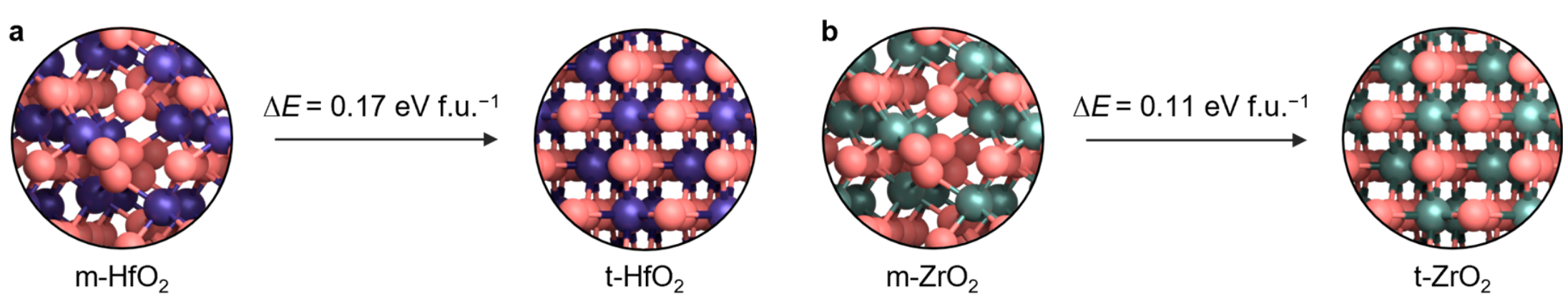

Supplementary Fig. 7. PBE-computed potential energy differences between monoclinic and tetragonal polymorphs of **a**, HfO_2 and **b**, ZrO_2.

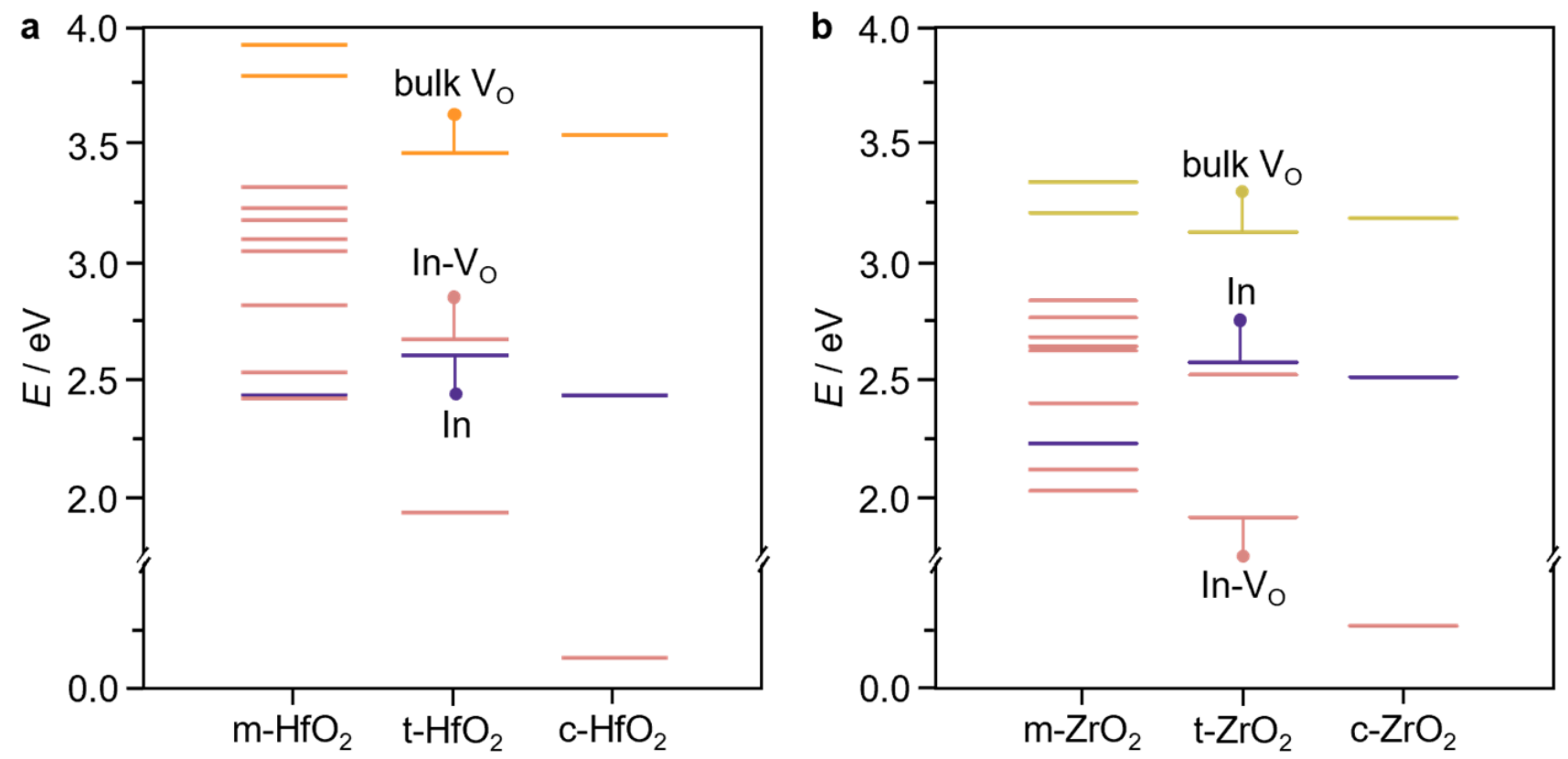

Supplementary Fig. 8. a,**b**, PBE-computed potential energies of monoclinic (m), tetragonal (t), and cubic (c) in the HfO_2 (**a**) and ZrO_2 (**b**) bulk supercells upon lattice substitution of an indium single atom in the presence (In-V_O) or absence (In) of an oxygen vacancy. Oxygen vacancy formation was assessed for all non-equivalent oxygen atoms of undoped MO_2 systems. For $InMO_x$, we explored vacancy formation for all distinct oxygens coordinated to indium.

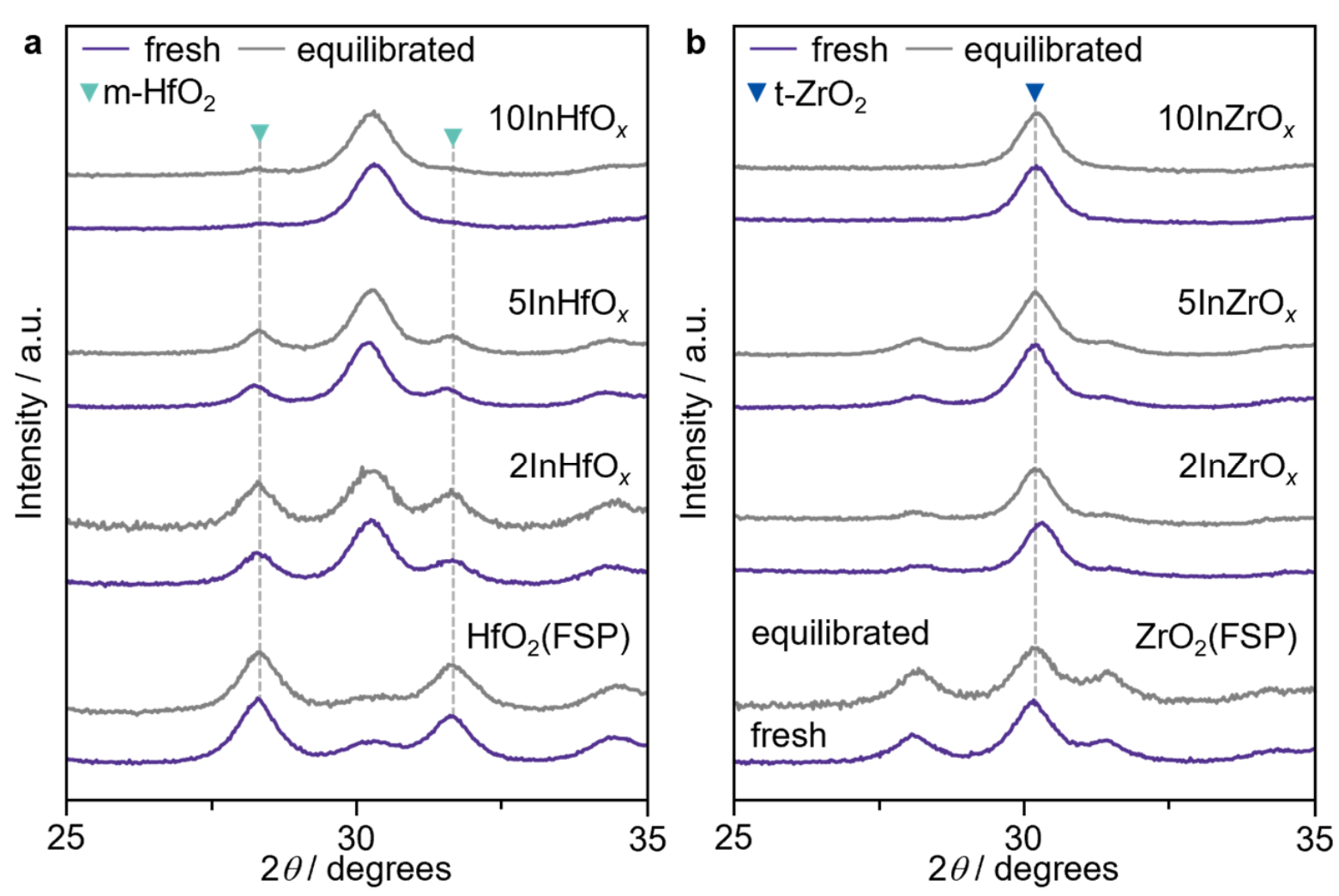

Supplementary Fig. 9. a, XRD patterns of **a**, InHfO_x and **b**, InZrO_x catalysts in fresh (purple lines) and equilibrated (grey lines) forms. The main reflections remain unchanged for both systems, indicating that indium atoms exist on the support surface as isolated sites, patches, or particles. Reaction conditions as indicated in **Supplementary Fig. 2**.

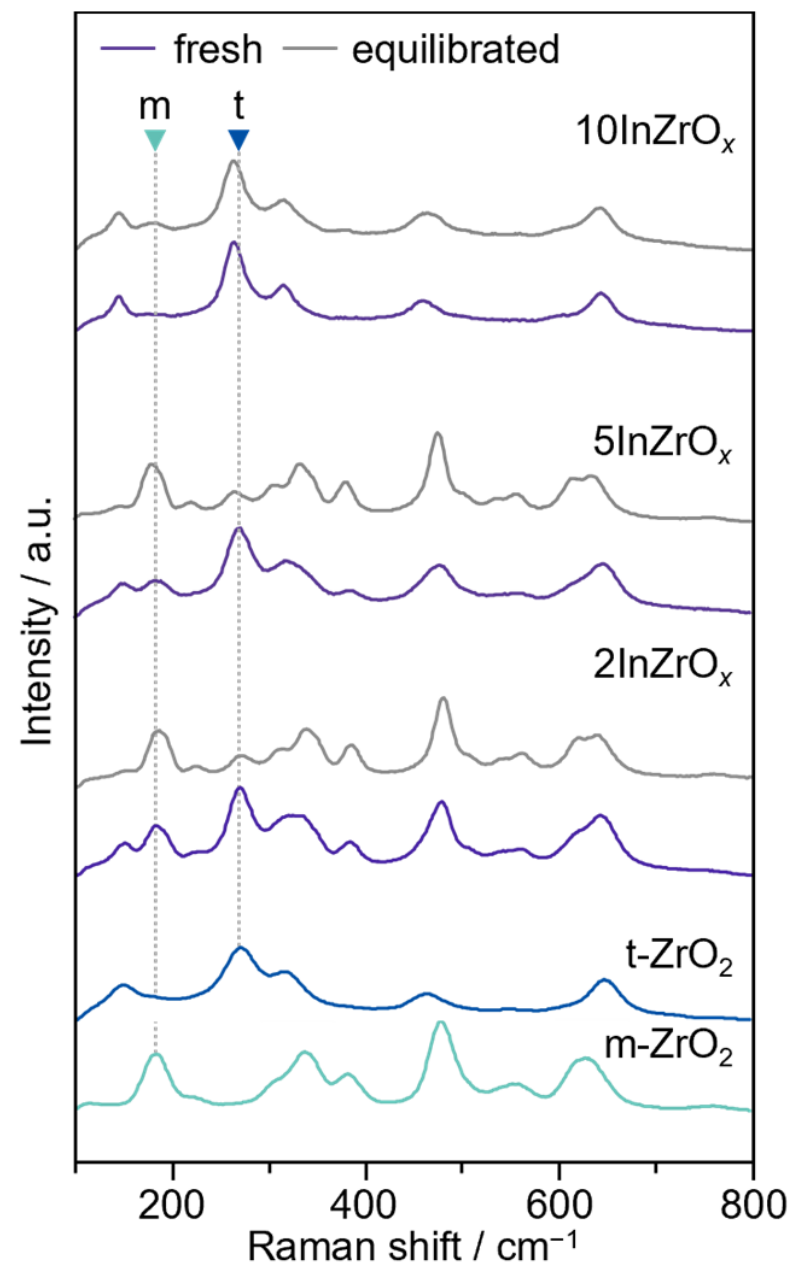

Supplementary Fig. 10. Raman spectra of $InZrO_x$ catalysts in fresh (purple lines) and equilibrated (grey lines) forms. Reaction conditions as indicated in **Supplementary Fig. 2**.

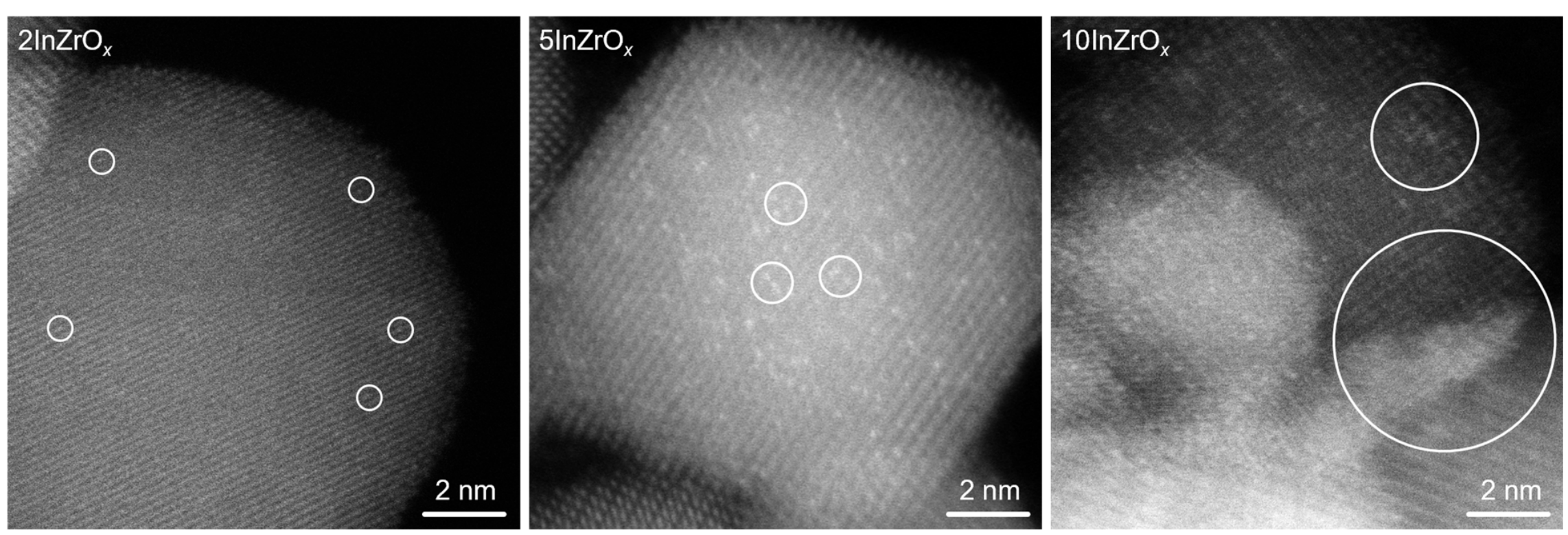

Supplementary Fig. 11. HAADF-STEM images of selected $InZrO_x$ catalysts in fresh form. White circles highlight distinct InO_x species, ranging from isolated single atoms to larger clusters and patches.

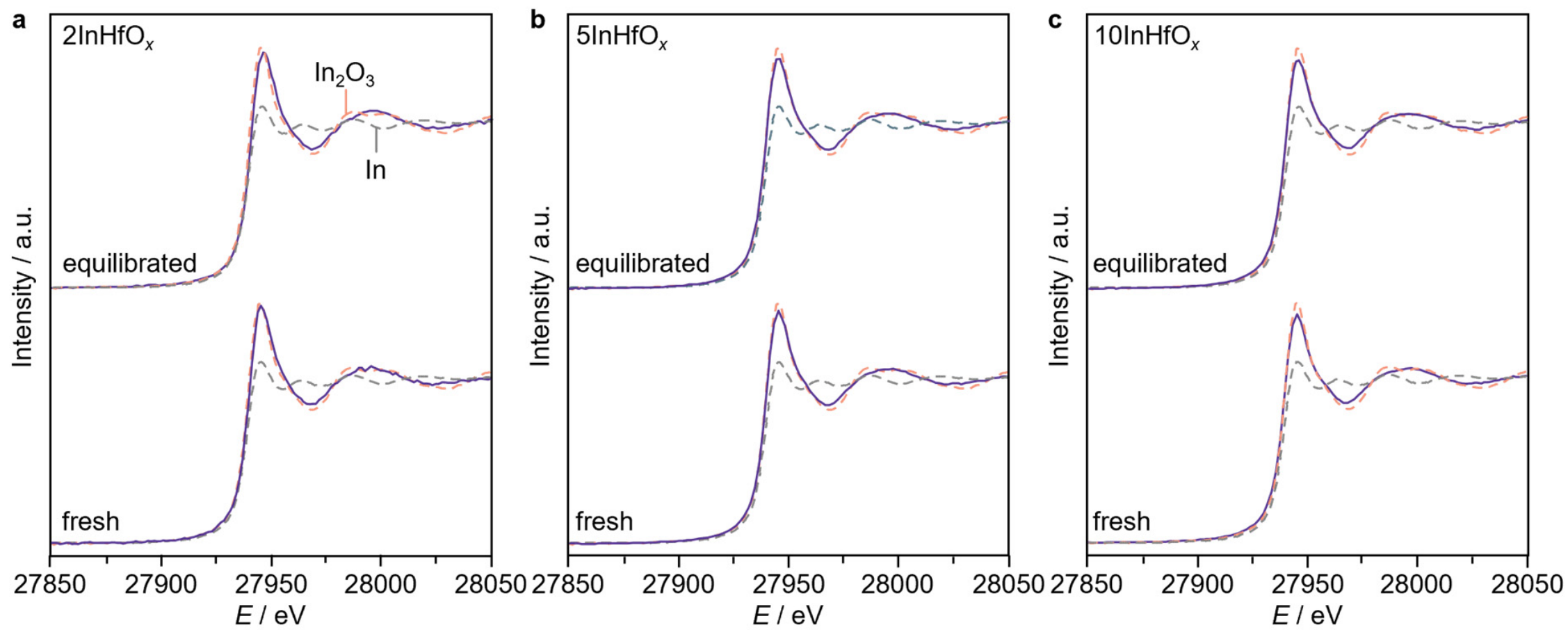

Supplementary Fig. 12. Ex-situ In *K*-edge XANES spectra of **a**, 2InHfO$_x$, **b**, 5InHfO$_x$, and **c**, 10InHfO$_x$ catalysts in fresh and equilibrated forms. Reference spectra of bulk In_2O_3 and In metal are shown as pink and grey dashed lines, respectively. Reaction conditions as indicated in **Supplementary Fig. 2**.

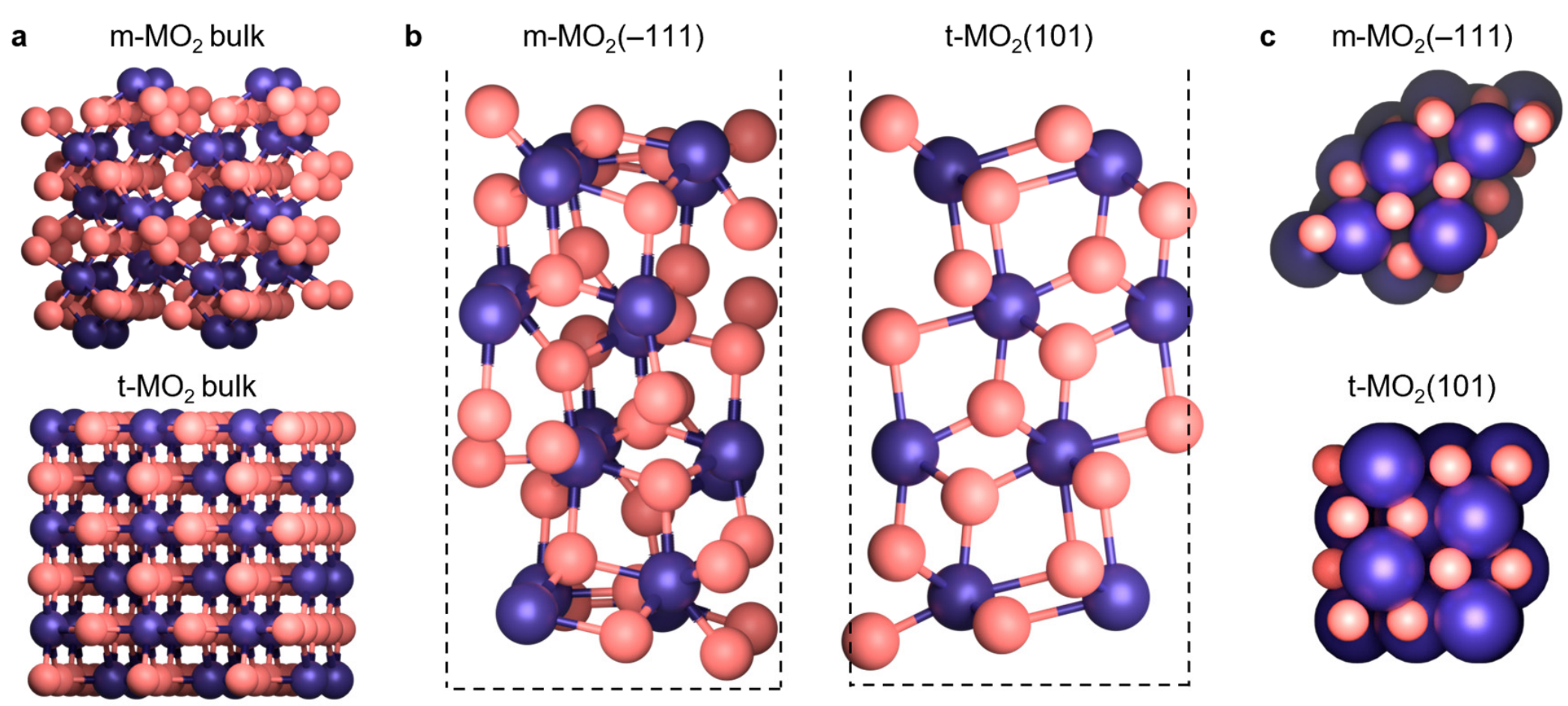

Supplementary Fig. 13. DFT models of **a**, bulk structures, and **b**,**c**, side and top views of m-HfO_2 and t-HfO_2 slab models used to evaluate the incorporation of single-atom indium species and oxygen vacancy formation. Colour code: Hf - purple; O - salmon pink. Equivalent models for monoclinic and tetragonal ZrO_2 were used in the corresponding simulations, differing only in lattice parameters.

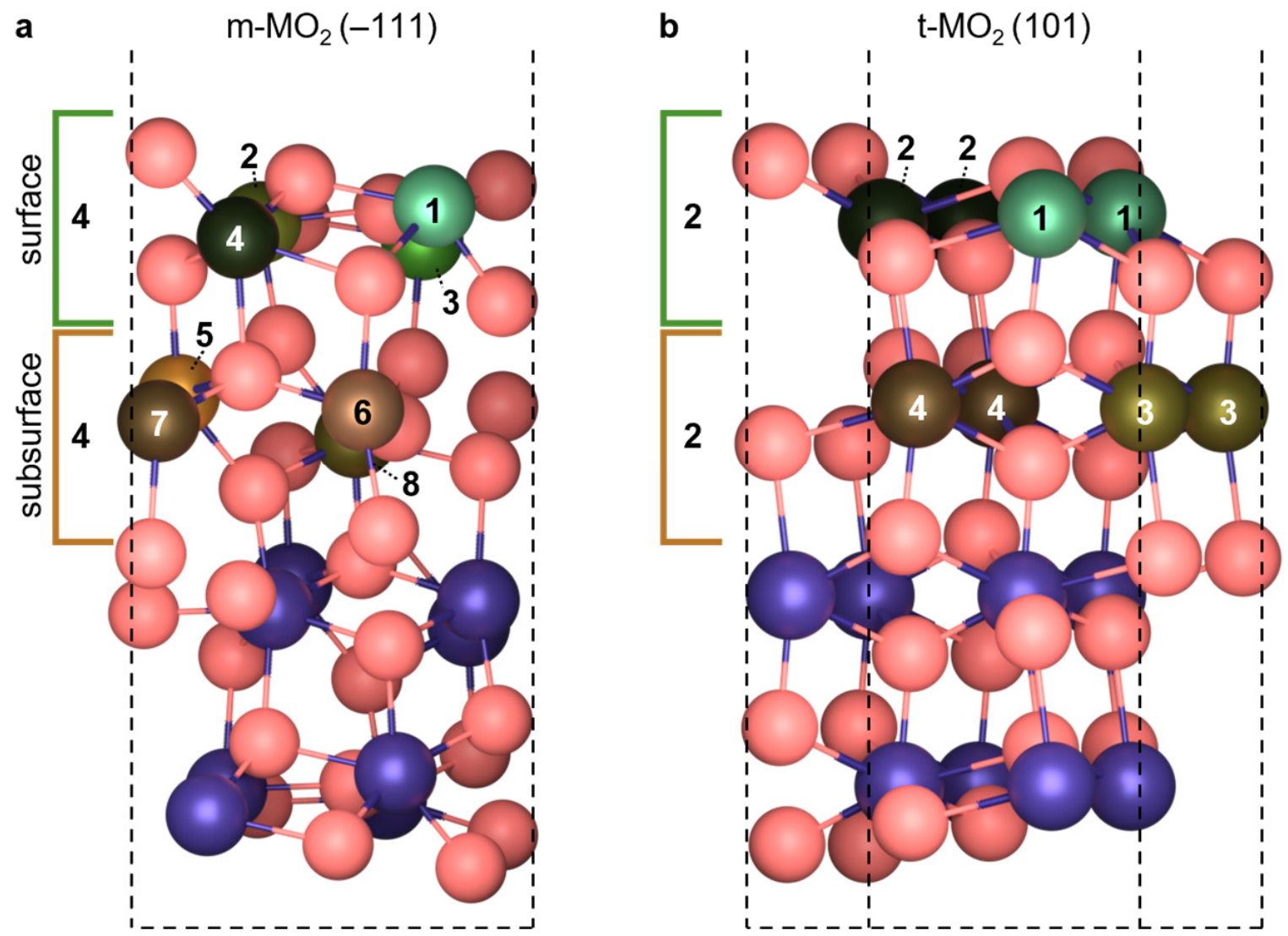

Supplementary Fig. 14. Side view of **a**, m-HfO_2 and **b**, t-HfO_2 slab models used in DFT simulations to assess indium incorporation at all non-equivalent Hf sites. Colour code: Distinct surface and subsurface Hf sites numbered 1-8 - varying shades of green and orange; Hf atoms in bulk positions - dark purple; O atoms - salmon pink. Equivalent models for m-ZrO_2 and t-ZrO_2 were used in the corresponding simulations, differing only slightly in lattice parameters.

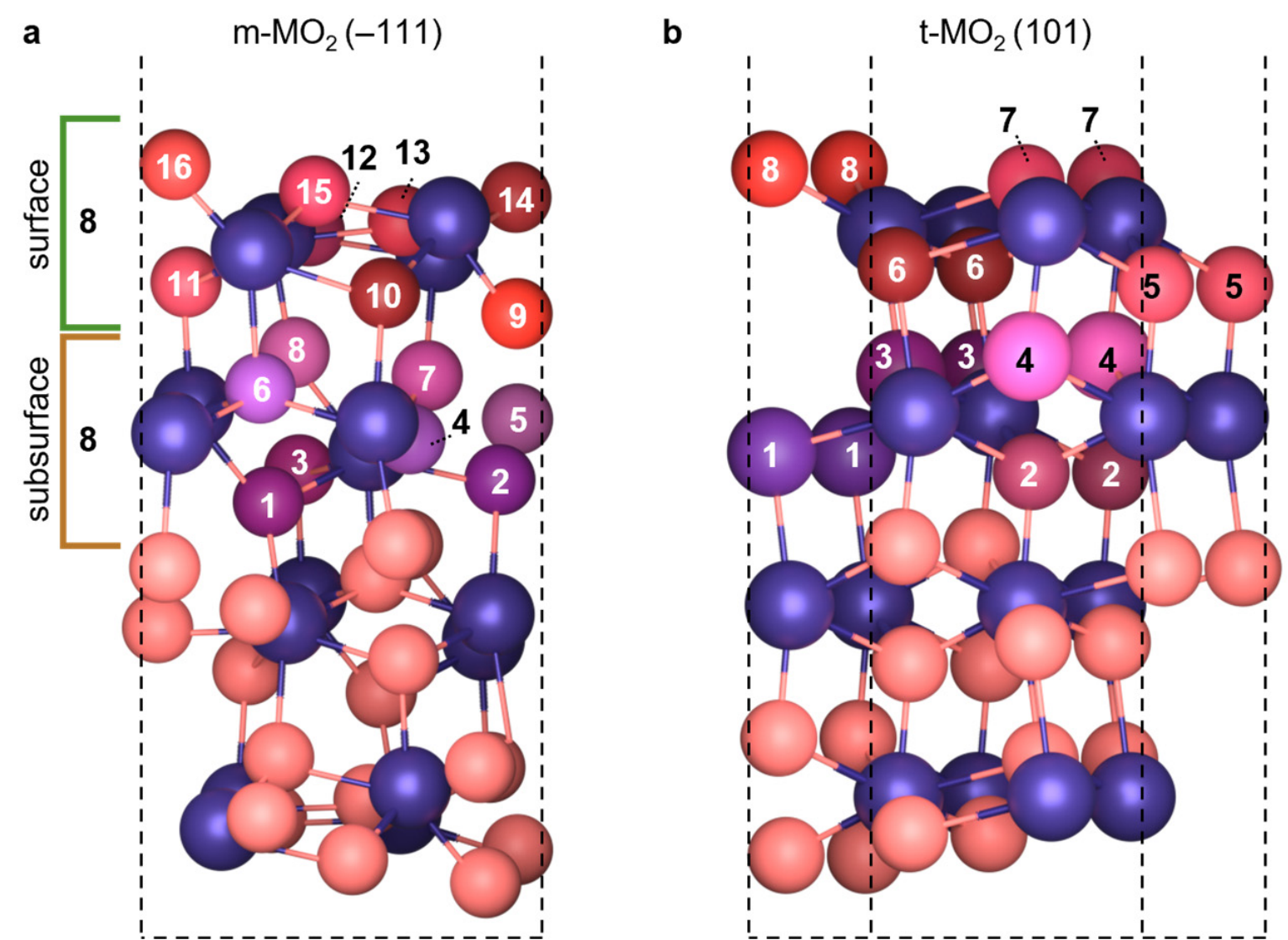

Supplementary Fig. 15. Side views of **a**, m-HfO_2 and **b**, t-HfO_2 slab models used in DFT simulations to evaluate oxygen vacancy formation at all non-equivalent oxygen sites. Colour code: Distinct surface and subsurface O sites numbered 1-16 – distinct red and purple shades; O atoms in bulk positions - salmon pink, Hf atoms - dark purple. Equivalent monoclinic and tetragonal ZrO_2 models were also used, differing only in lattice parameters.

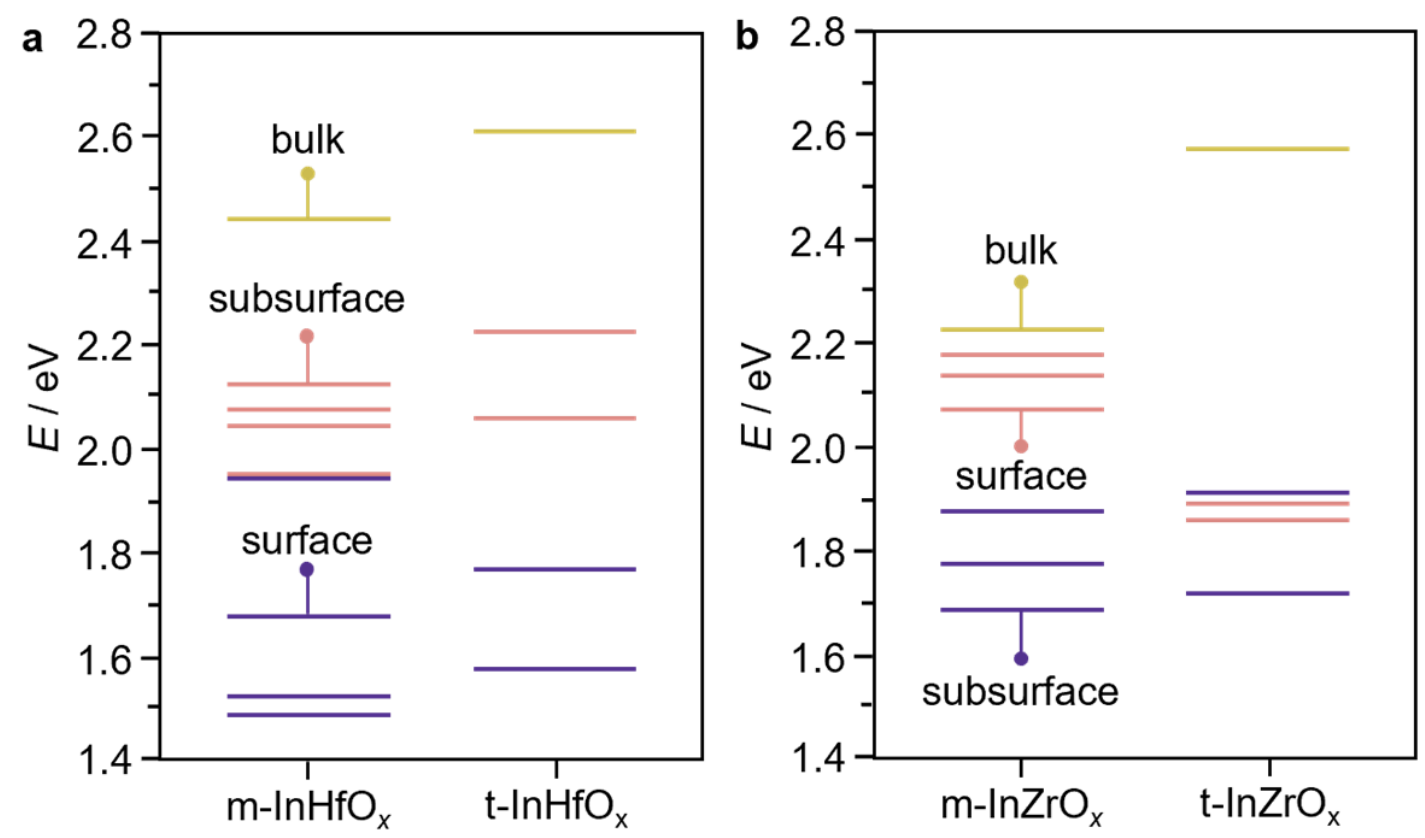

Supplementary Fig. 16. PBE-computed potential energies of substituting an indium single atom into monoclinic and tetragonal slab models of **a**, HfO_2 and **b**, ZrO_2. Indium atoms were substituted at all non-equivalent surface and subsurface Hf or Zr sites, respectively. The energy for substituting an indium atom in the bulk, representing one site per polymorph, is shown for reference.

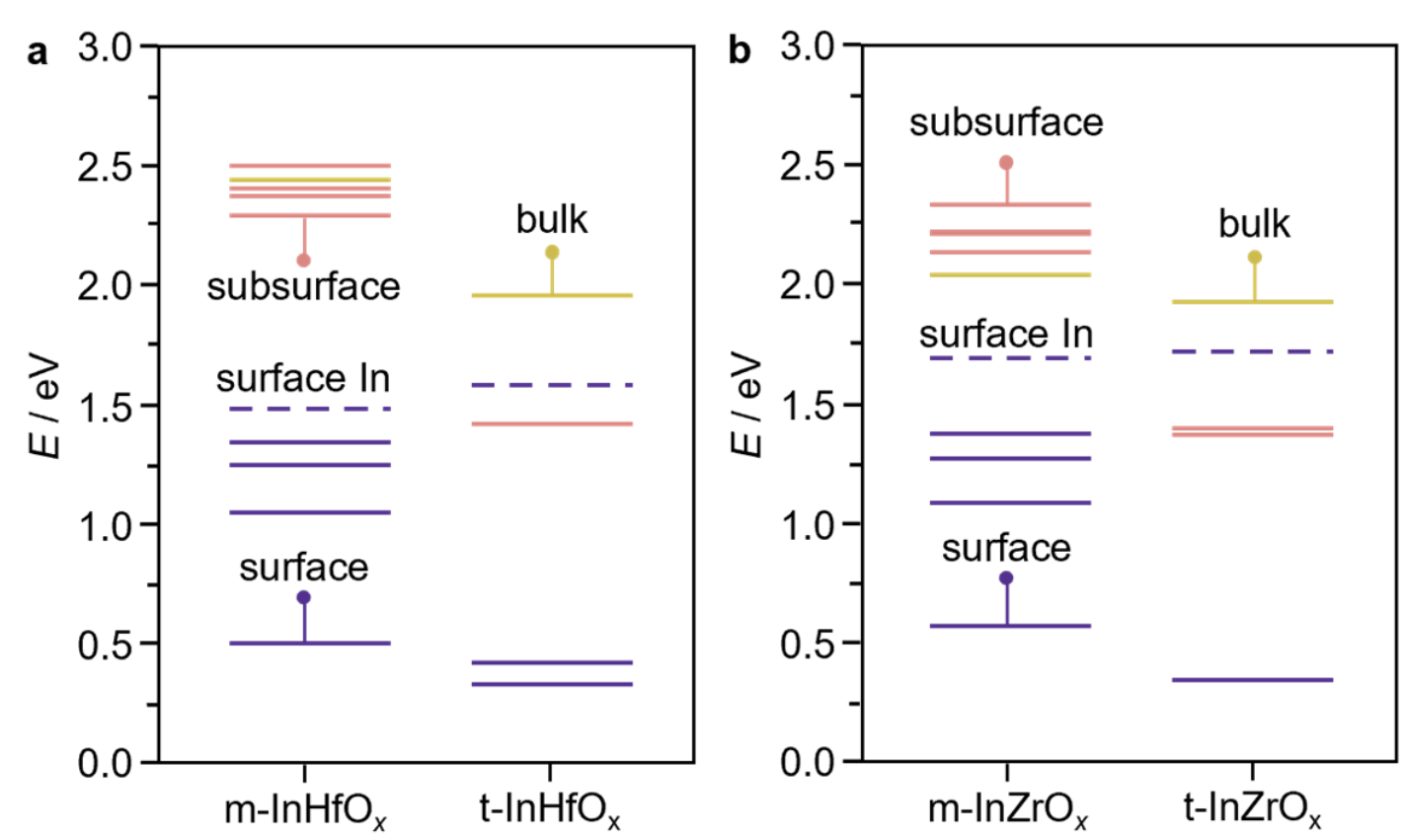

Supplementary Fig. 17. PBE-computed potential energies for substituting an indium single atom into monoclinic and tetragonal models of **a**, HfO_2 and **b**, ZrO_2 containing one oxygen vacancy. Indium atoms were substituted into all non-equivalent surface and subsurface Hf or Zr sites, respectively, and oxygen vacancy formation was assessed for all non-equivalent oxygens. The energy of substituting an indium atom in the bulk, representing a single site per polymorph, is shown in yellow, and the lowest energy for substituting an indium atom without a vacancy (**Supplementary Fig. 16**) is shown with dashed lines for reference.

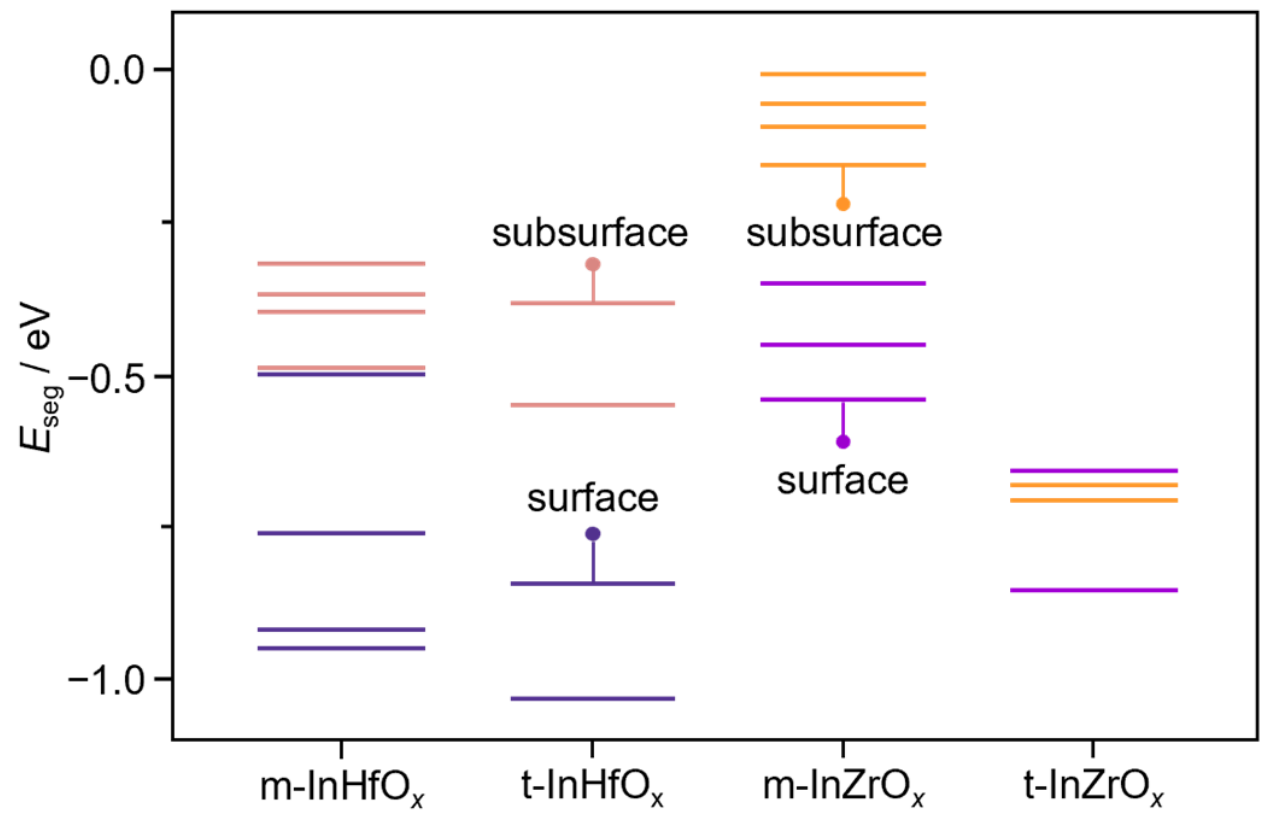

Supplementary Fig. 18. PBE-computed segregation energies (E_{seg}) for the migration of an indium atom from bulk to surface and subsurface sites in distinct tetragonal and monoclinic slab models of HfO_2 and ZrO_2.

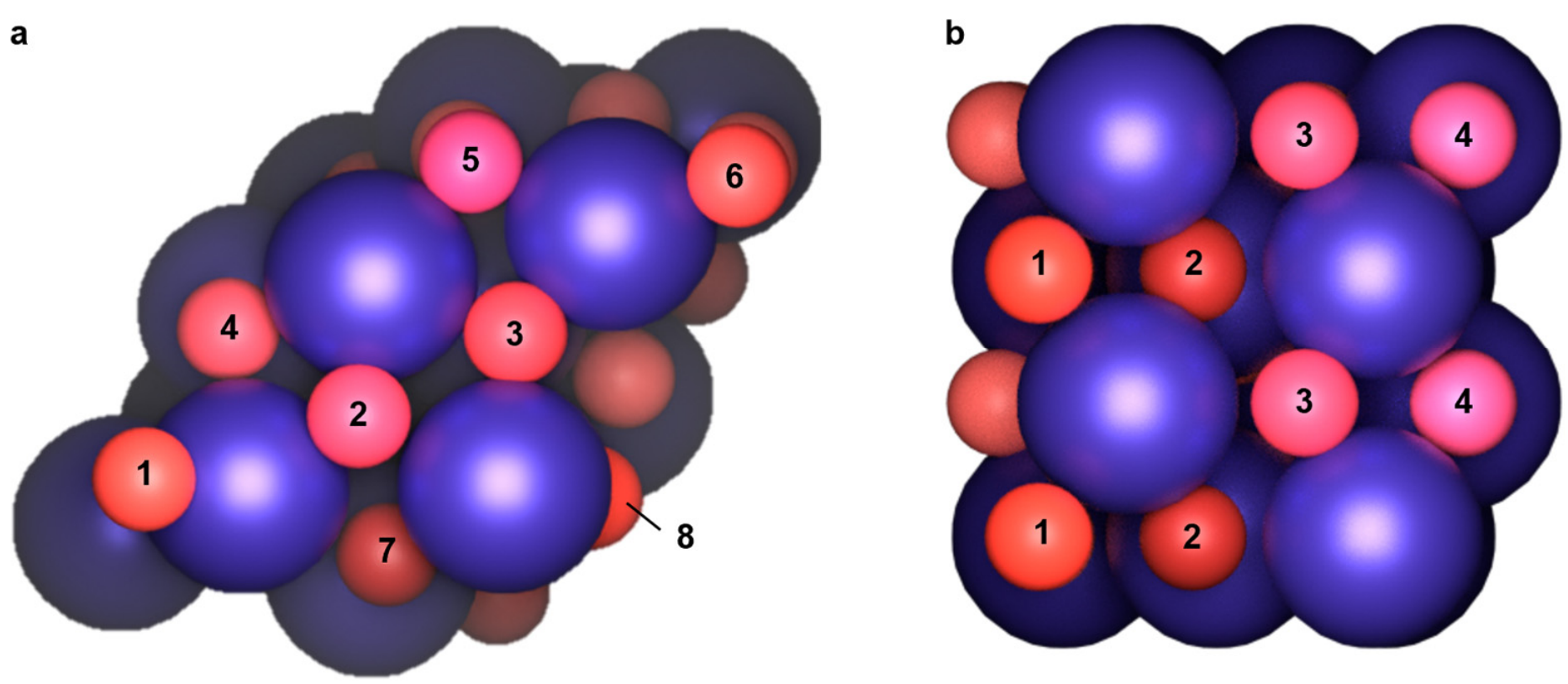

Supplementary Fig. 19. Top view of slab models of **a**, m-HfO_2 and **b**, t-HfO_2 used in DFT simulations to assess the anchoring of single bare and hydroxylated indium adatoms at various surface oxygen sites. Colour code: Distinct surface O sites numbered 1-8 (varying shades of red); M atoms in bulk positions (dark purple); O atoms (salmon pink). Models of monoclinic and tetragonal ZrO_2 are analogous, with minor lattice differences.

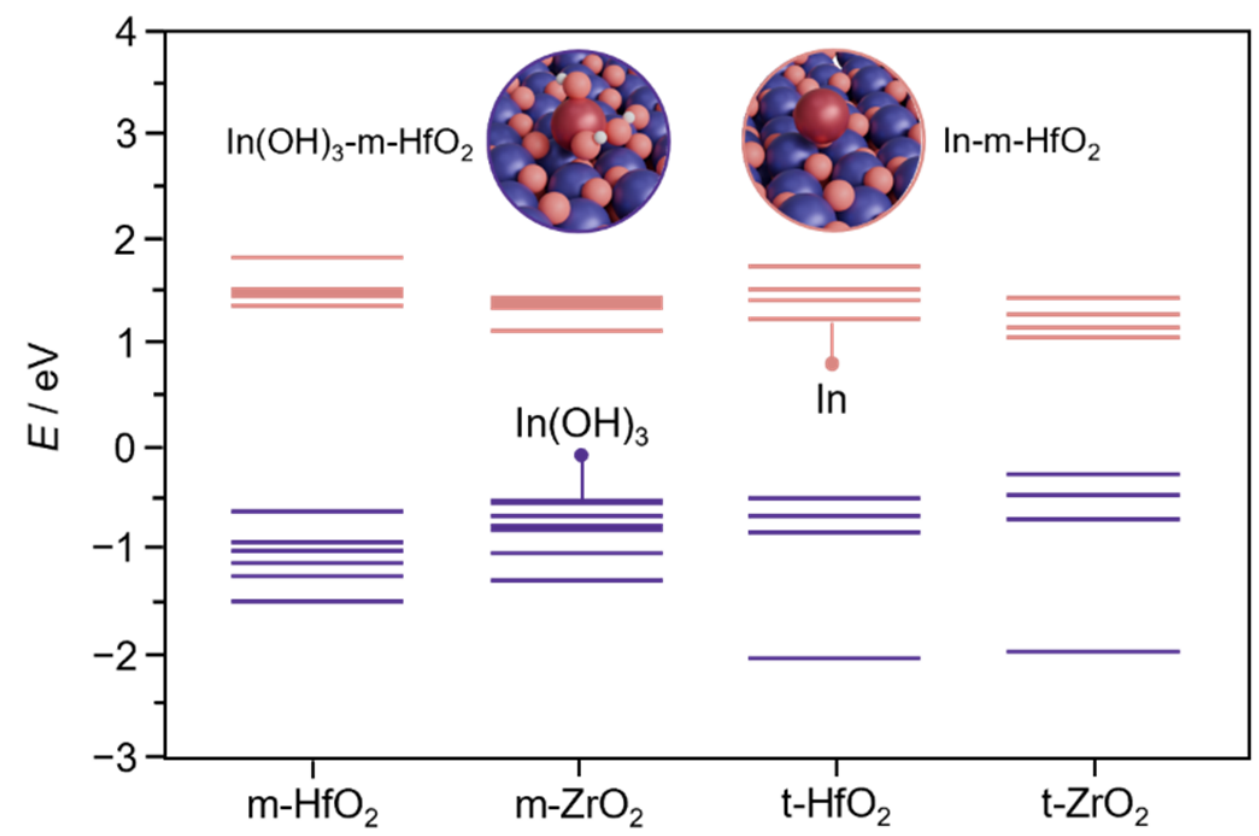

Supplementary Fig. 20. PBE-computed adsorption energies of single bare or hydroxylated indium adatoms on monoclinic and tetragonal HfO_2 and ZrO_2 polymorphs in the non-equivalent positions shown in **Supplementary Fig. 19**.

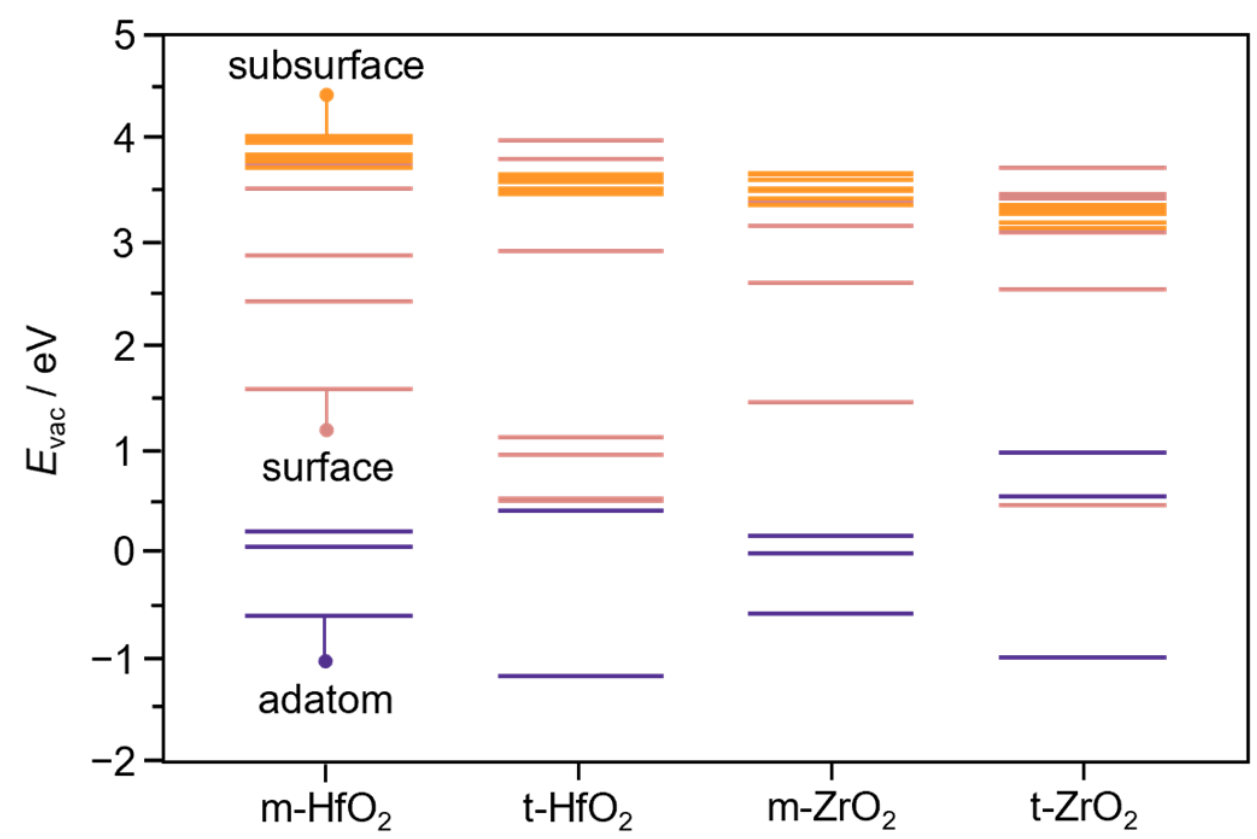

Supplementary Fig. 21. PBE-computed vacancy formation energies (E_{vac}) of a single oxygen vacancy for monoclinic and tetragonal HfO_2 and ZrO_2 models with $In(OH)_3$ adsorbed. Vacancy formation was explored for all non-equivalent oxygen atoms in the surface and subsurface, as well as at three sites corresponding to the hydroxyl oxygen atoms of the adsorbed species.

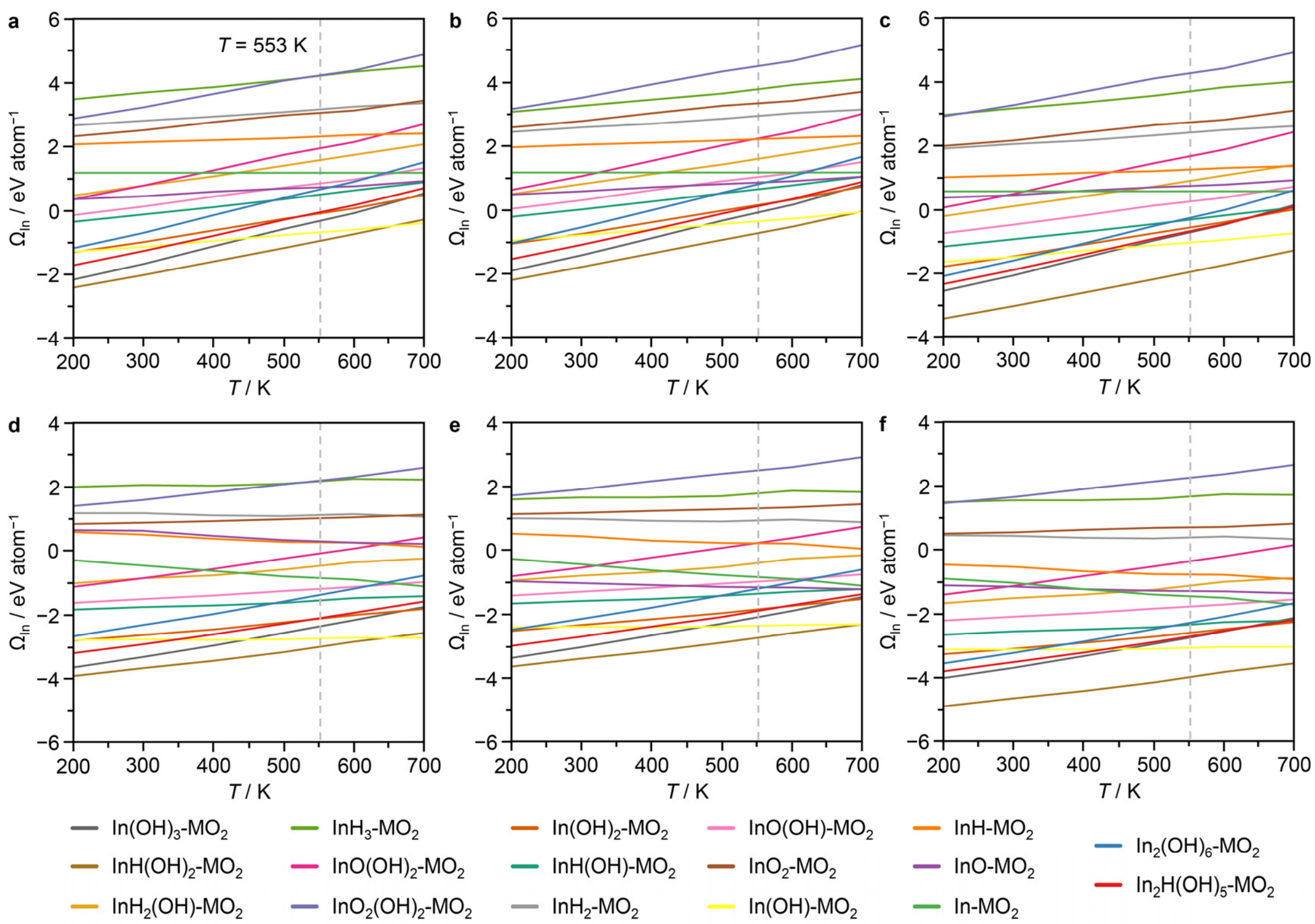

Supplementary Fig. 22. Calculated grand chemical potential of $InH_xO_y(OH)_{3-x-y}$ species adsorbed on **a**,**d**, m-HfO_2, **b**,**e**, m-ZrO_2, **c**,**f**, t-ZrO_2 plotted as a function of temperature (T) with respect to the **a-c**, bulk In and **d-f**, gas-phase $In(OH)_3$ chemical potential (see **Supplementary Note 1**).

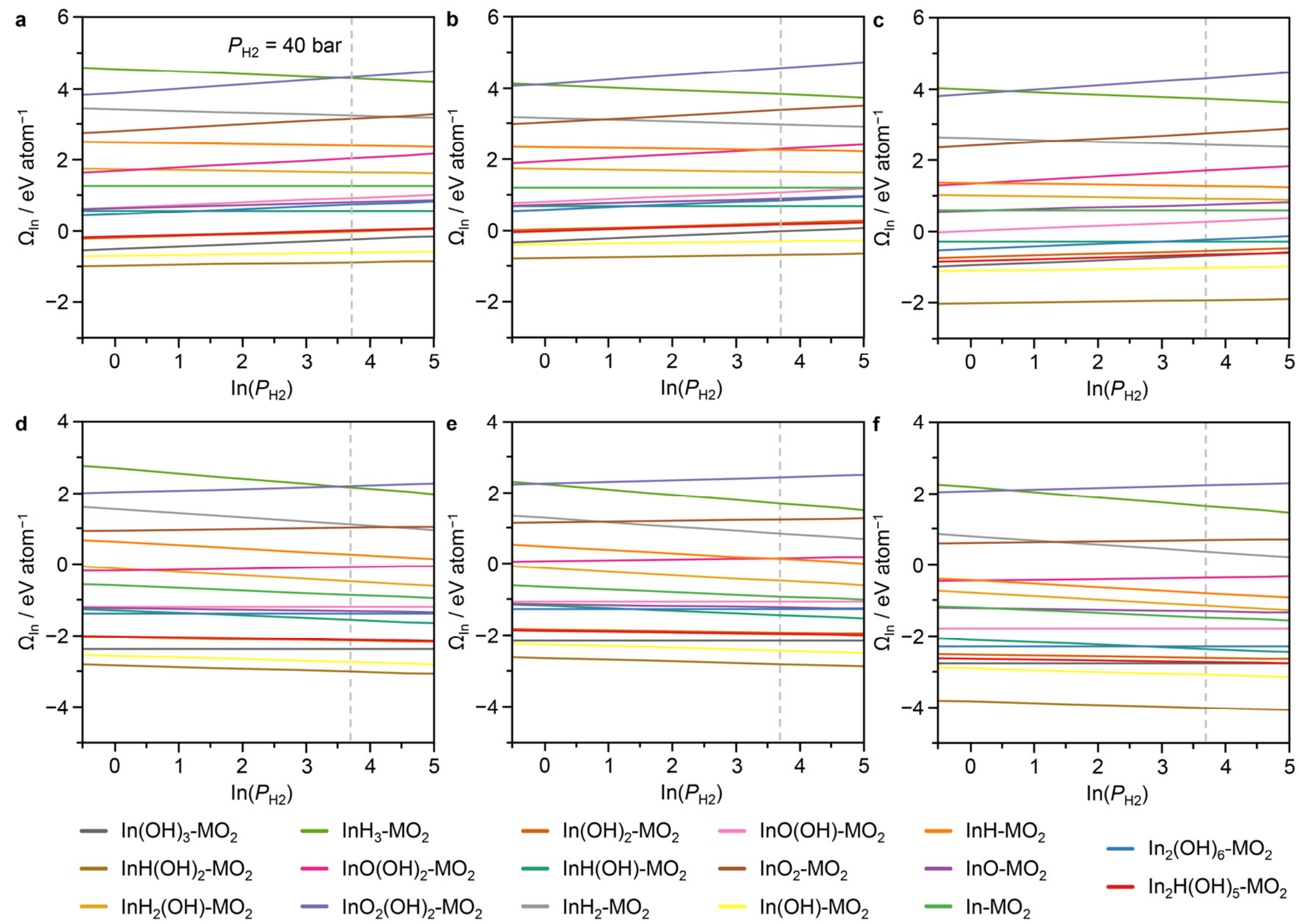

Supplementary Fig. 23. Calculated grand chemical potential of $InH_xO_y(OH)_{3-x-y}$ species adsorbed on **a**,**d**, m-HfO_2, **b**,**e**, m-ZrO_2, **c**,**f**, t-ZrO_2 plotted as a function of H_2 partial pressure (P_{H2}) with respect to the **a-c**, bulk In and **d-f**, gas-phase $In(OH)_3$ chemical potential (see **Supplementary Note 1**).

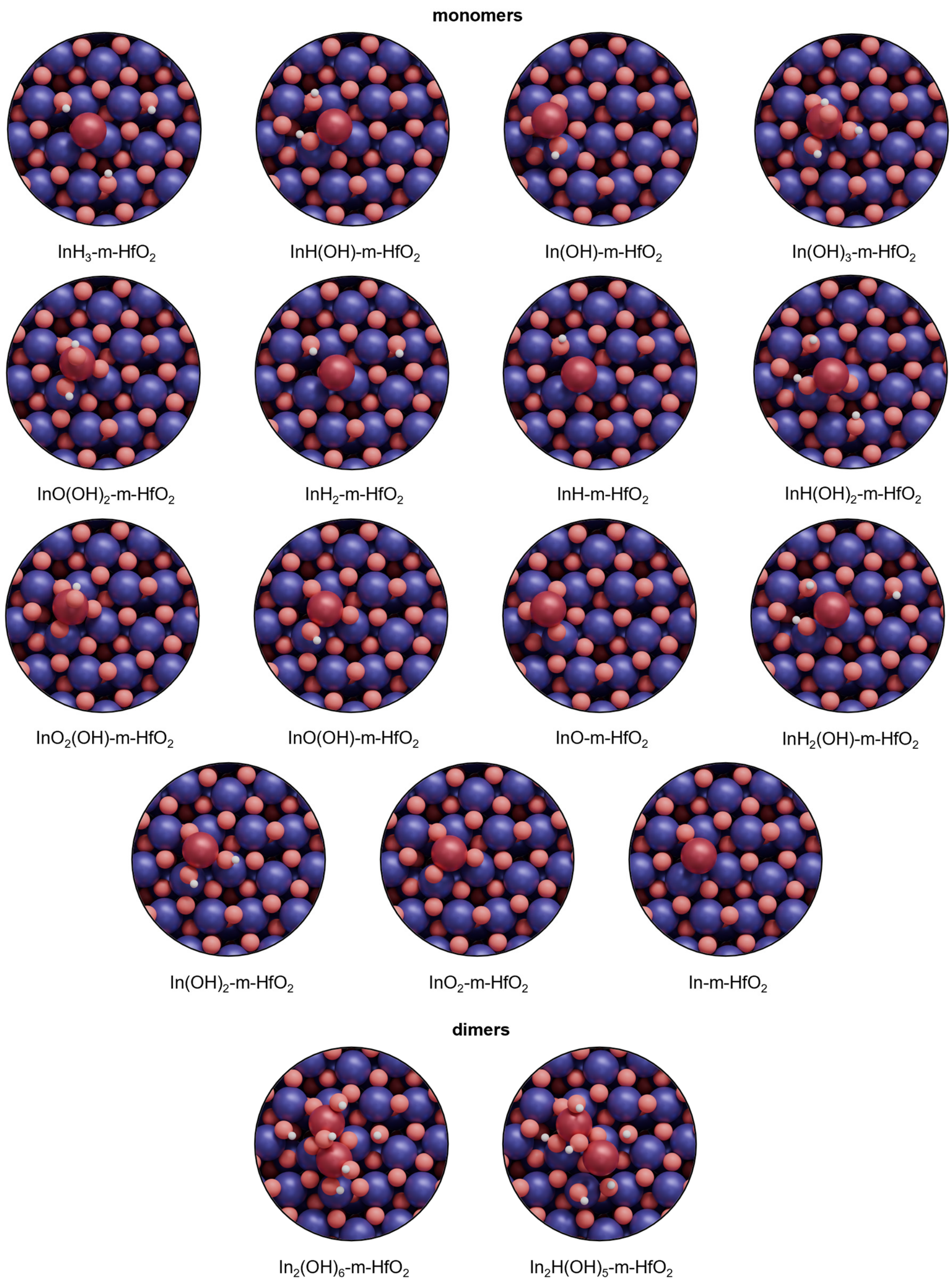

Supplementary Fig. 24. Top-view projections of representative adsorbed $InH_xO_y(OH)_{3-x-y}$ structures on m-HfO_2 surfaces are indicated in the stability diagrams shown in **Supplementary Figs. 22-23**. Colour code: Hf - purple; In - red; O - salmon pink; H - white.

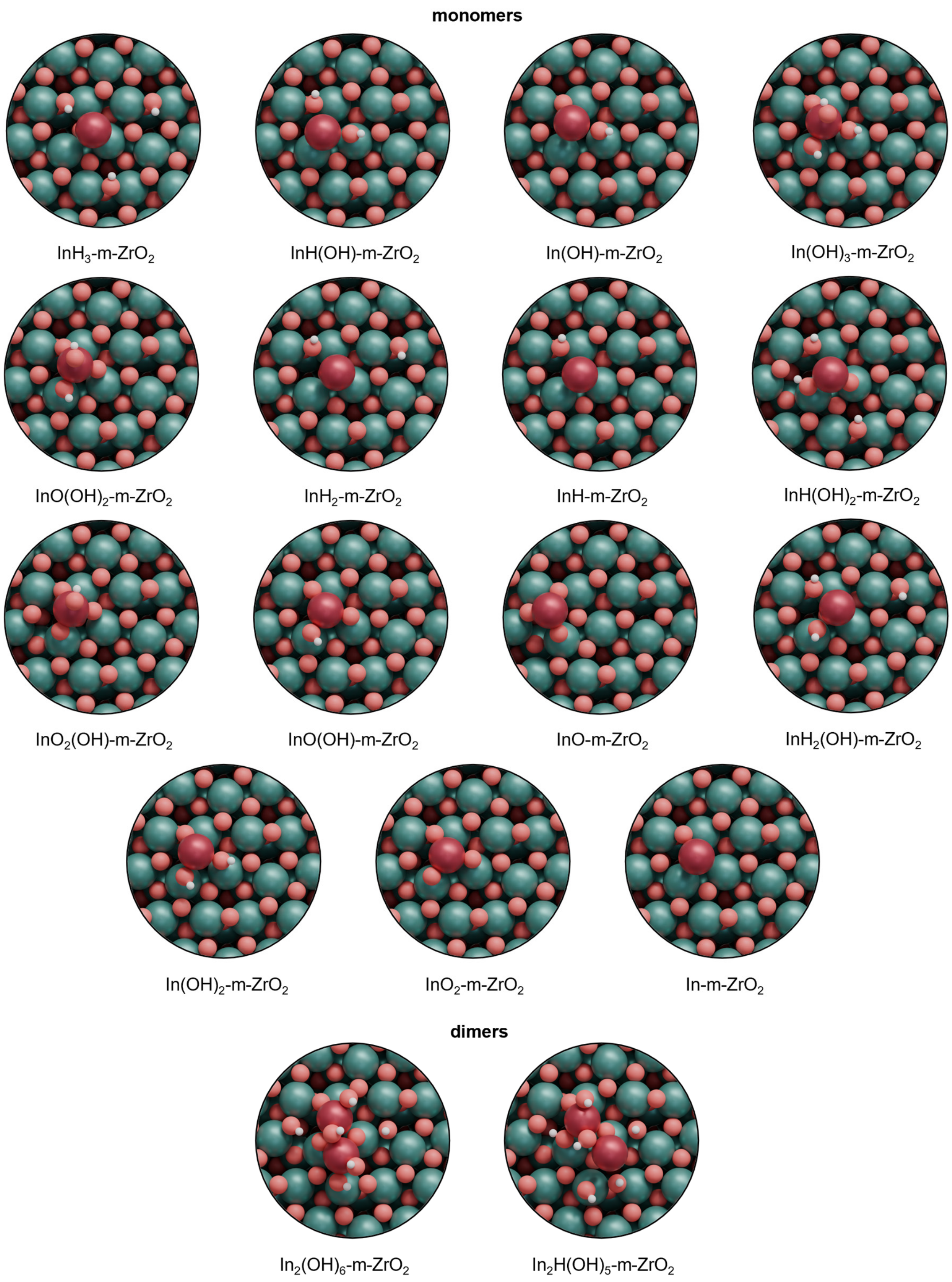

Supplementary Fig. 25. Top-view projections of representative adsorbed $InH_xO_y(OH)_{3-x-y}$ structures on m-ZrO_2 surfaces corresponding to stability diagrams shown in **Supplementary Figs. 22-23**. Colour code: Zr - teal; In - red; O - salmon pink; H - white.

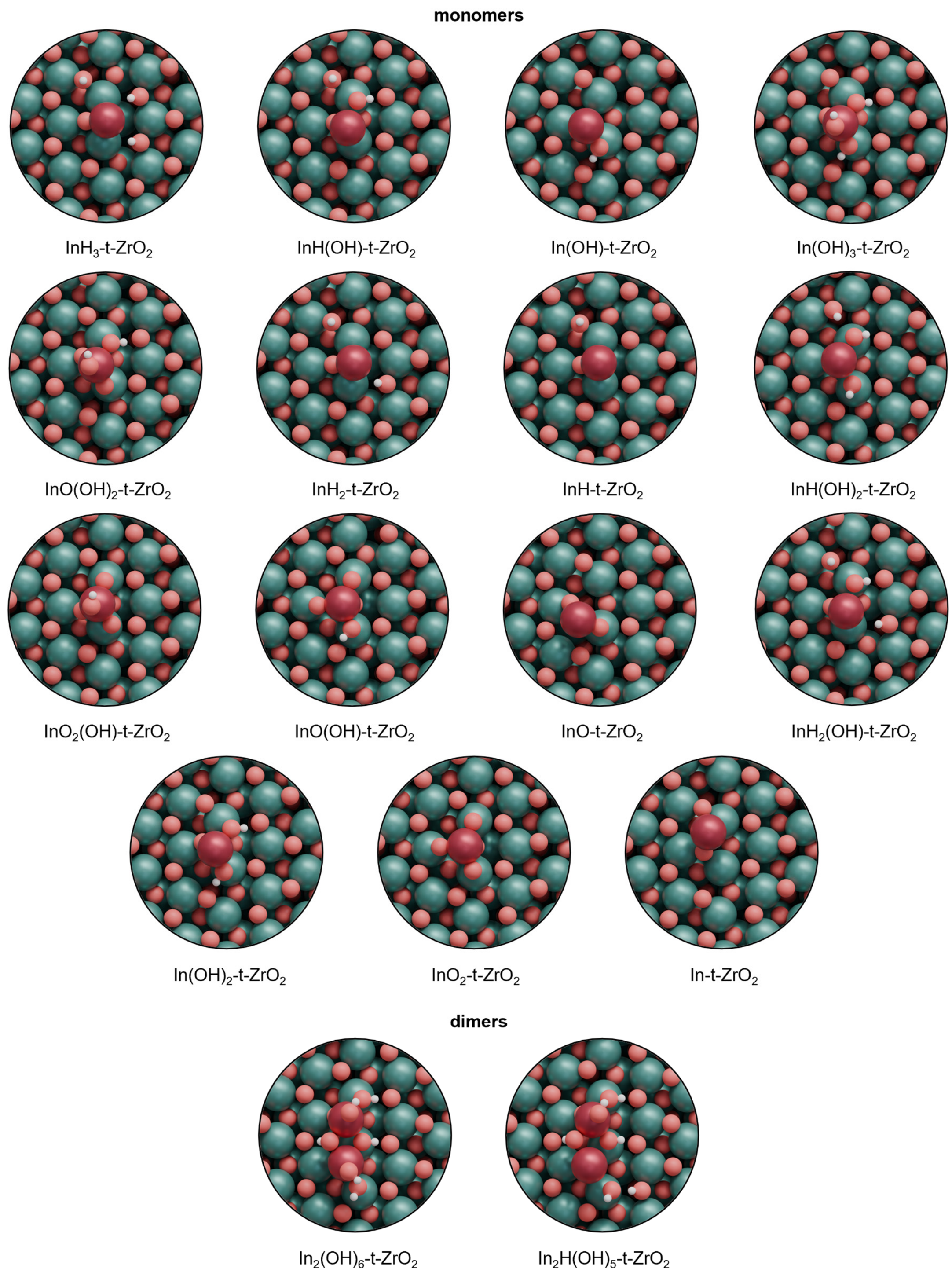

Supplementary Fig. 26. Top-view projections of representative adsorbed $InH_xO_y(OH)_{3-x-y}$ structures on t-ZrO_2 surfaces corresponding to stability diagrams shown in **Supplementary Figs. 22-23**. Colour code: Zr - teal; In - red; O - salmon pink; H - white.

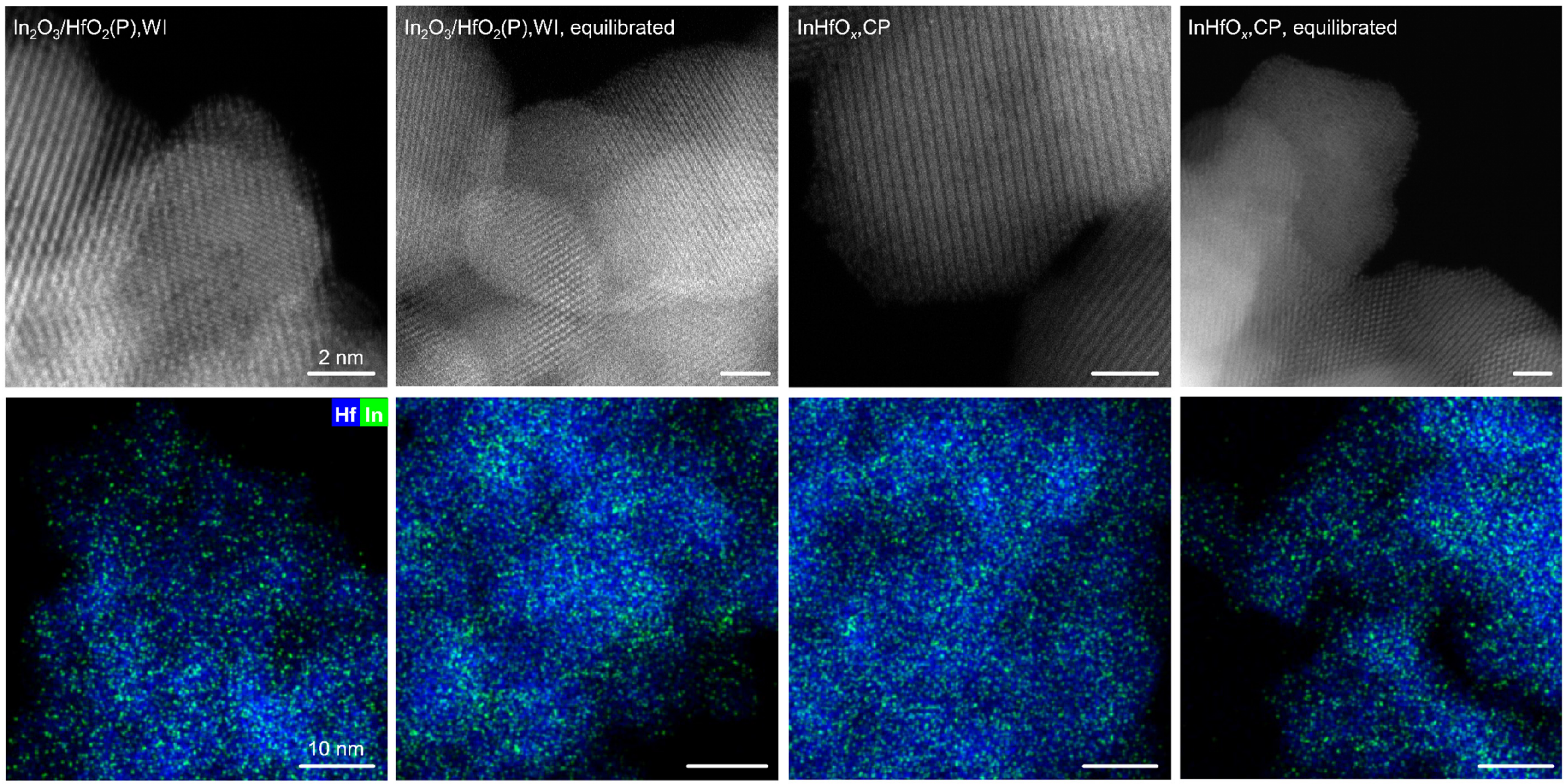

Supplementary Fig. 27. Representative HAADF-STEM images (top) and corresponding elemental maps (bottom) of In_2O_3/HfO_2(P),WI and $InHfO_x$,CP in fresh and equilibrated forms. The results confirm atomic dispersions of InO_x species in all cases. Catalyst codes as reported in **Supplementary Table 2**. Reaction condition as described in **Supplementary Fig. 2**.

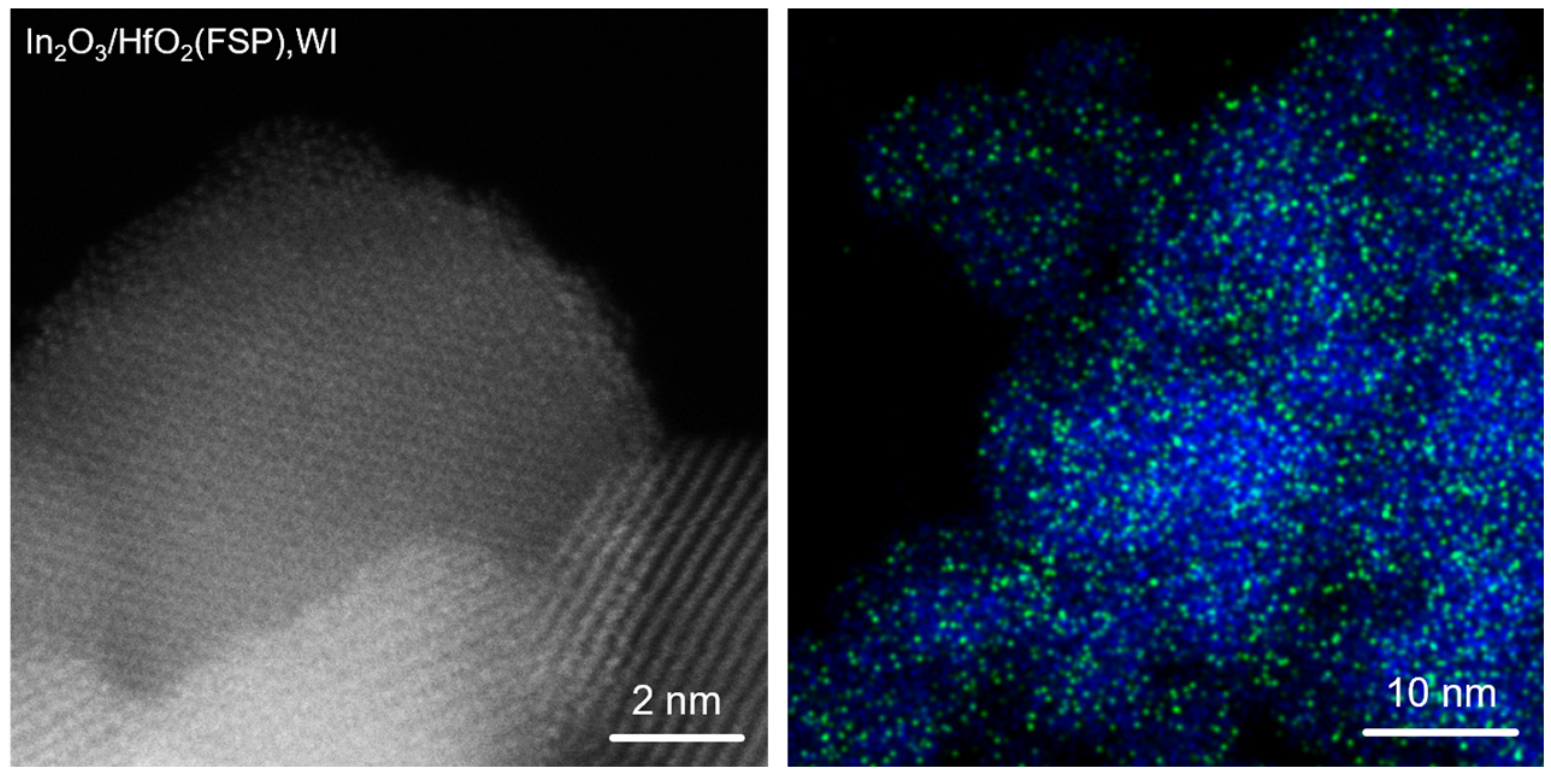

Supplementary Fig. 28. HAADF-STEM image (left) and corresponding elemental map (right) of In_2O_3/HfO_x(FSP),WI in fresh and equilibrated forms. Reaction conditions as described in **Supplementary Fig. 2**.

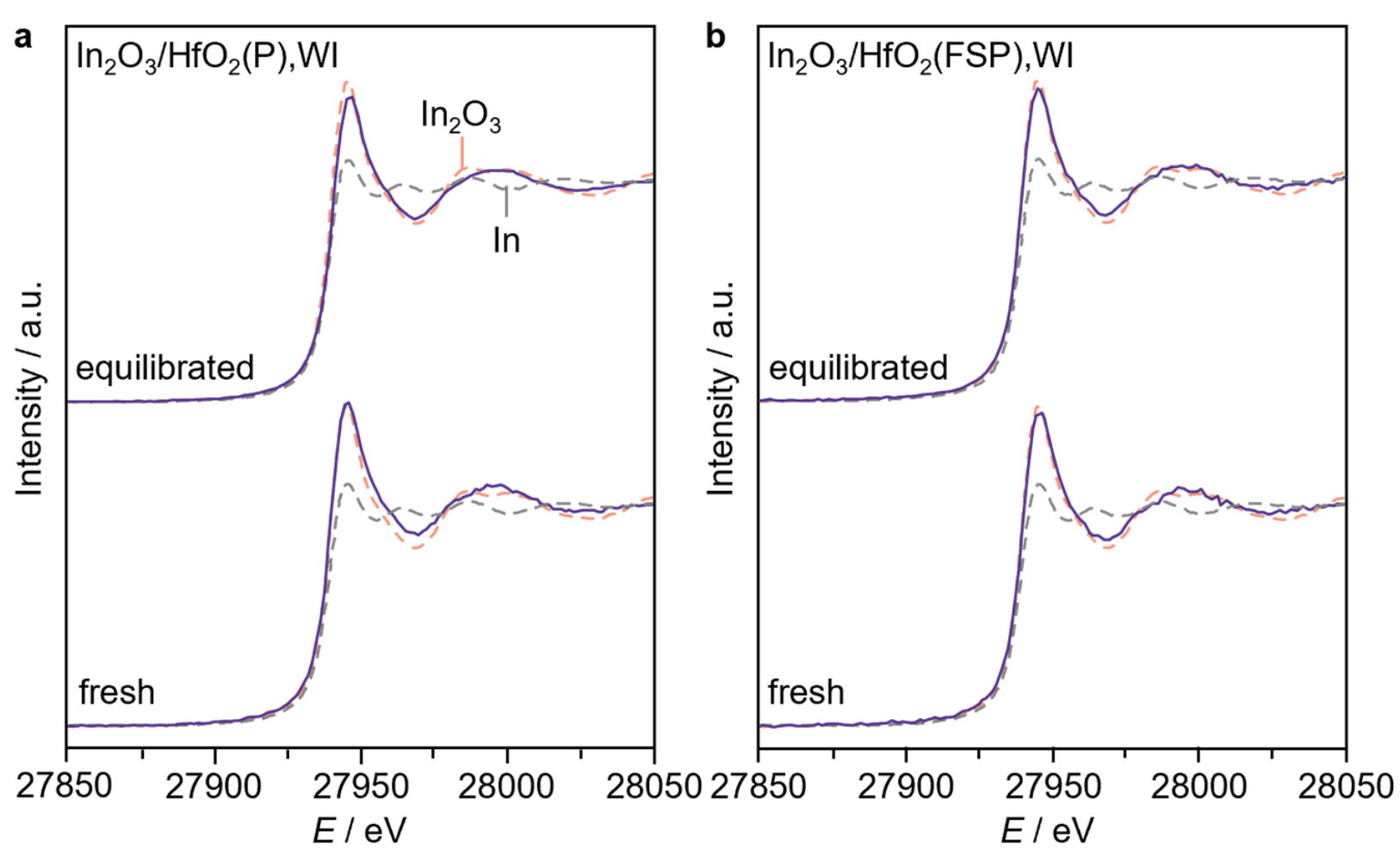

Supplementary Fig. 29. Ex-situ In *K*-edge XANES spectra of **a**, In_2O_3/HfO_2(P),WI and **b**, In_2O_3/HfO_2(FSP),WI catalysts in fresh and equilibrated forms. Reference spectra of bulk In_2O_3 and In metal are shown with orange and grey dashed lines, respectively. Reaction conditions as described in **Supplementary Fig. 2**.

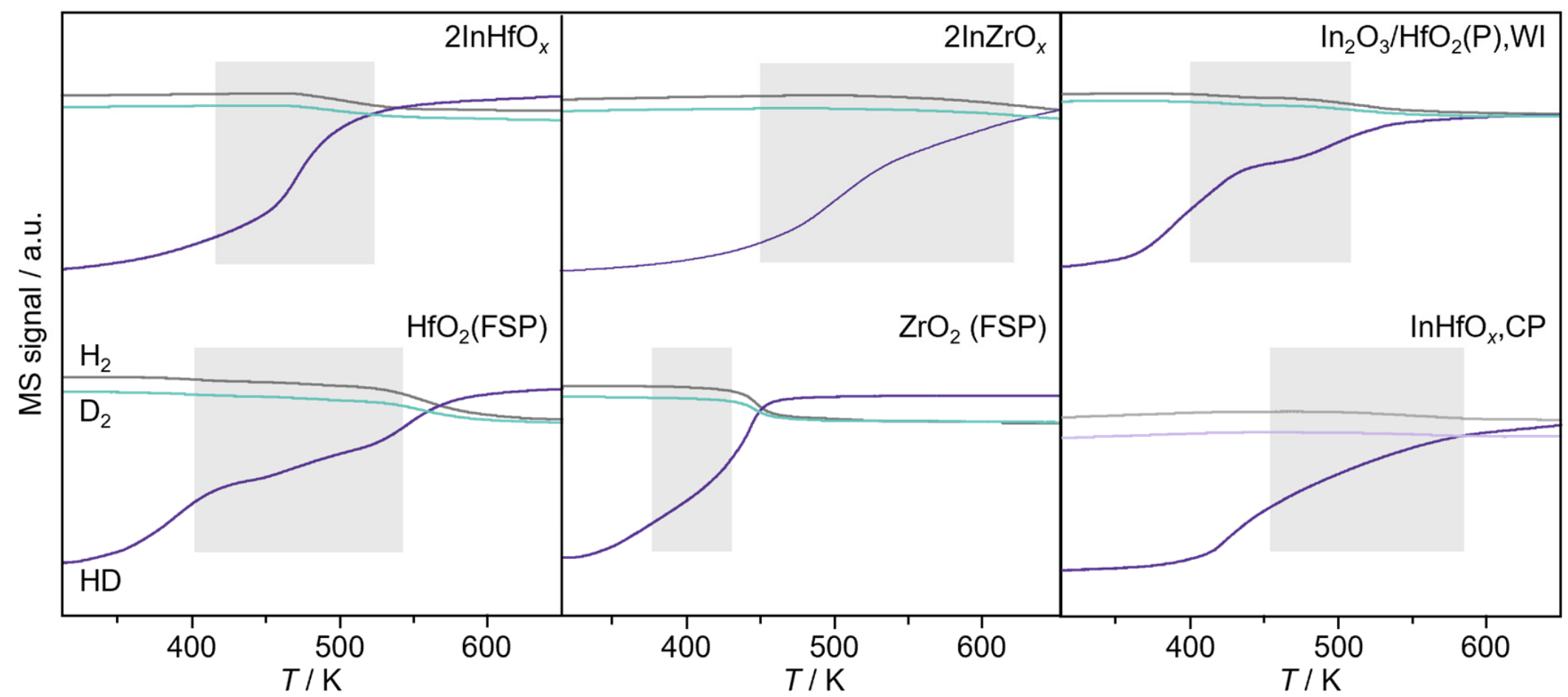

Supplementary Fig. 30. Temperature-programmed H_2-D_2 exchange profiles of selected catalysts. Shaded areas indicate temperature ranges where isotopic exchange occurs. Corresponding profiles of the bare supports are shown for comparison.

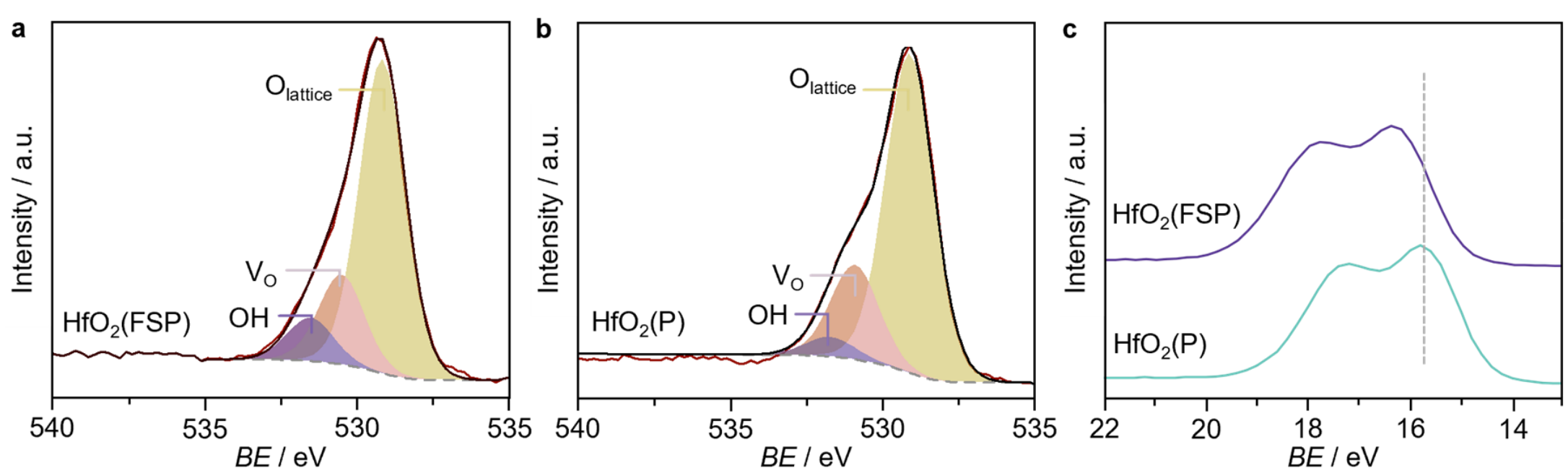

Supplementary Fig. 31. XPS O 1*s* core-level spectra for **a**, HfO_2(FSP) and **b**, HfO_2(P), highlighting differences in oxygen coordination environments. **c**, XPS Hf 4*f* core-level spectra of both samples.

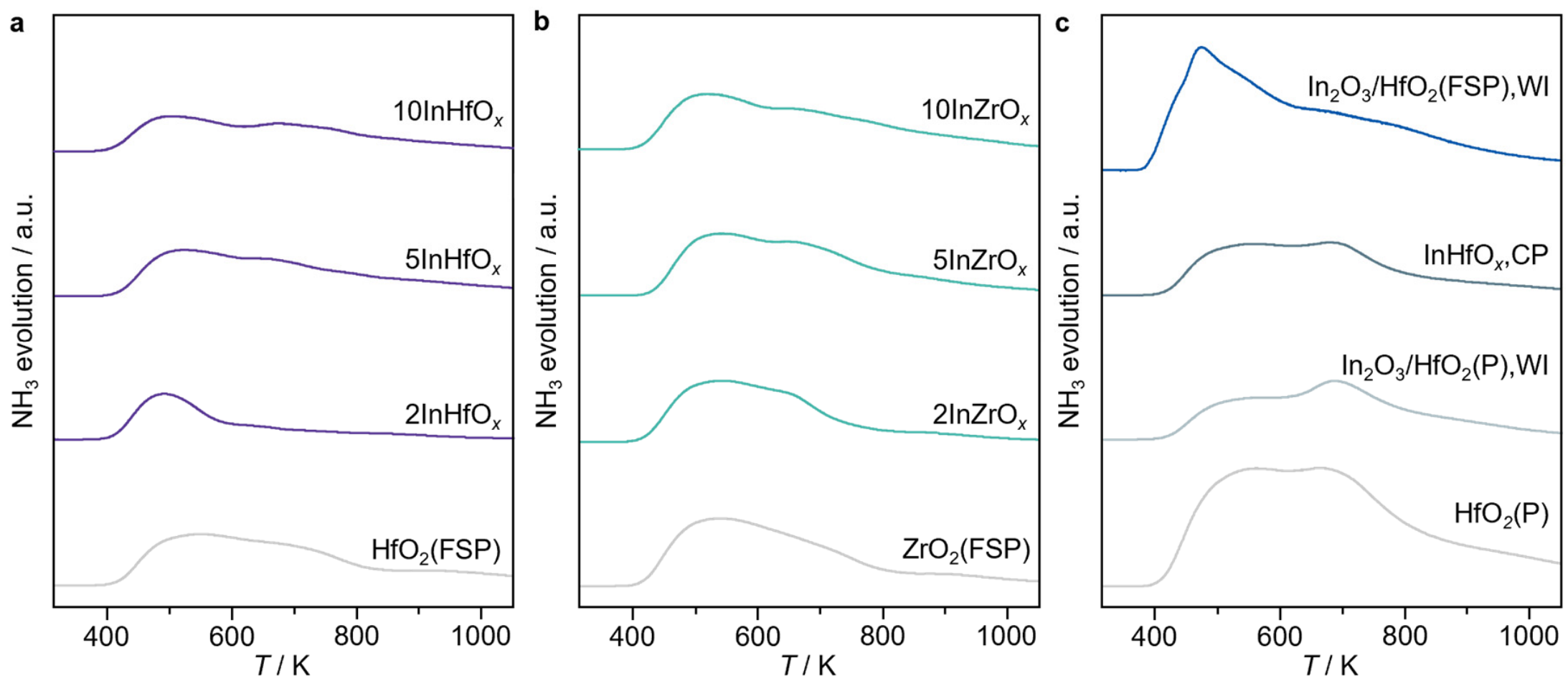

Supplementary Fig. 32. NH_3-TPD profiles of **a**, InHfO$_x$ catalysts, and reference **b**, InZrO$_x$ catalysts, and **c**, HfO_2-supported systems. The profiles of the pure supports are shown for reference.

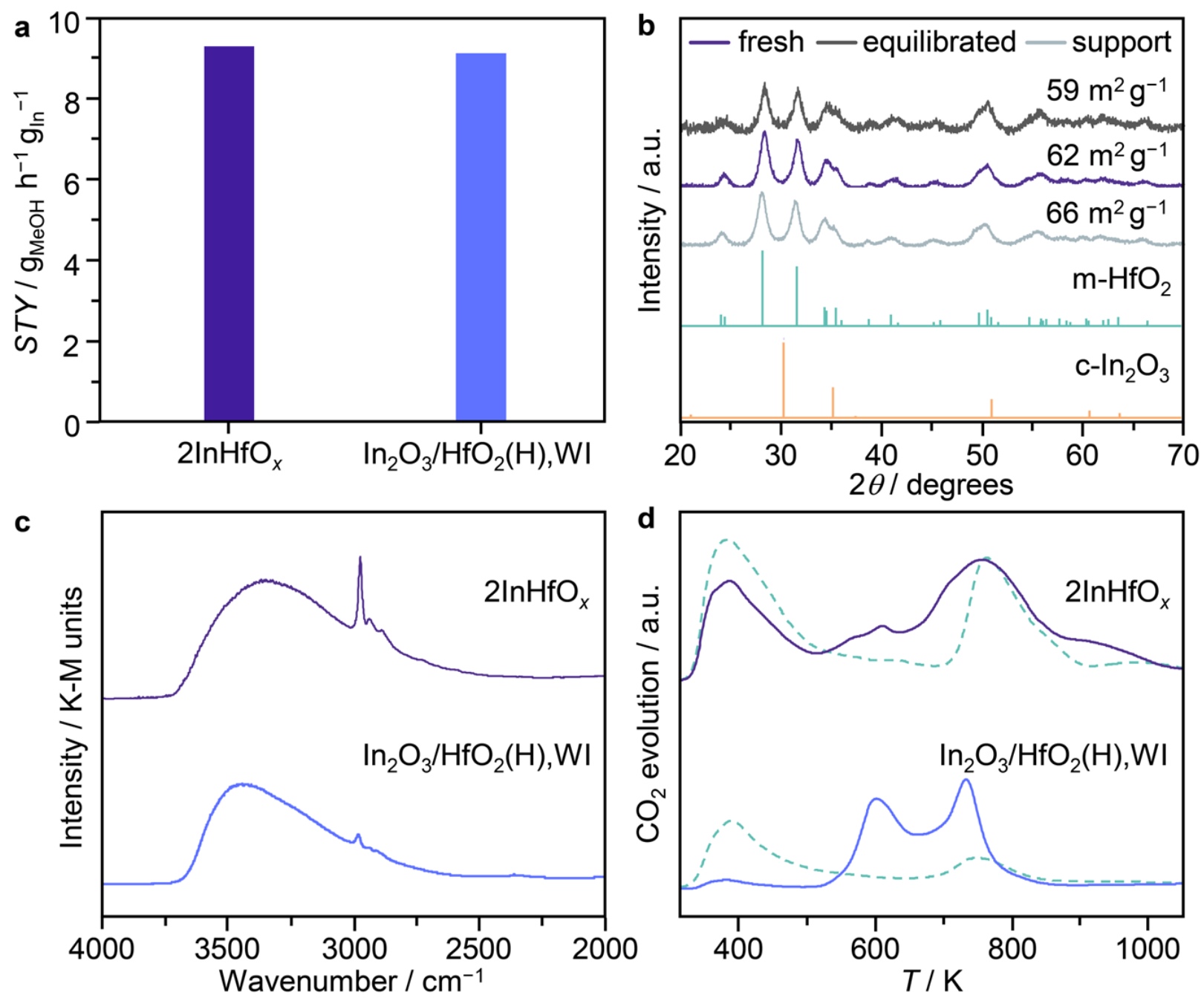

Supplementary Fig. 33. a, Methanol space-time yield (*STY*) of 2InHfO_x and In_2O_3/HfO_2(H), WI catalysts. In the latter, indium is introduced via wet impregnation of a HfO_2 support prepared by a controlled hydrothermal method (see **Methods**). Reaction conditions: $T = 553$ K, $P = 50$ bar, $H_2/CO_2 = 4$, $GHSV = 24{,}000$ cm^3 h^{-1} g_{cat}^{-1}, *tos* = 20 h. **b**, XRD profiles of In_2O_3/HfO_2(H), WI catalyst. **c**, Ex-situ DRIFT spectra of the hydroxyl region and **d**, CO_2-TPD profiles of 2InHfO_x and In_2O_3/HfO_2(H), WI catalysts. These results indicate that the surface hydroxyl groups of HfO_2(H) are preserved during hydrothermal synthesis, enabling efficient CO_2 adsorption and activation.

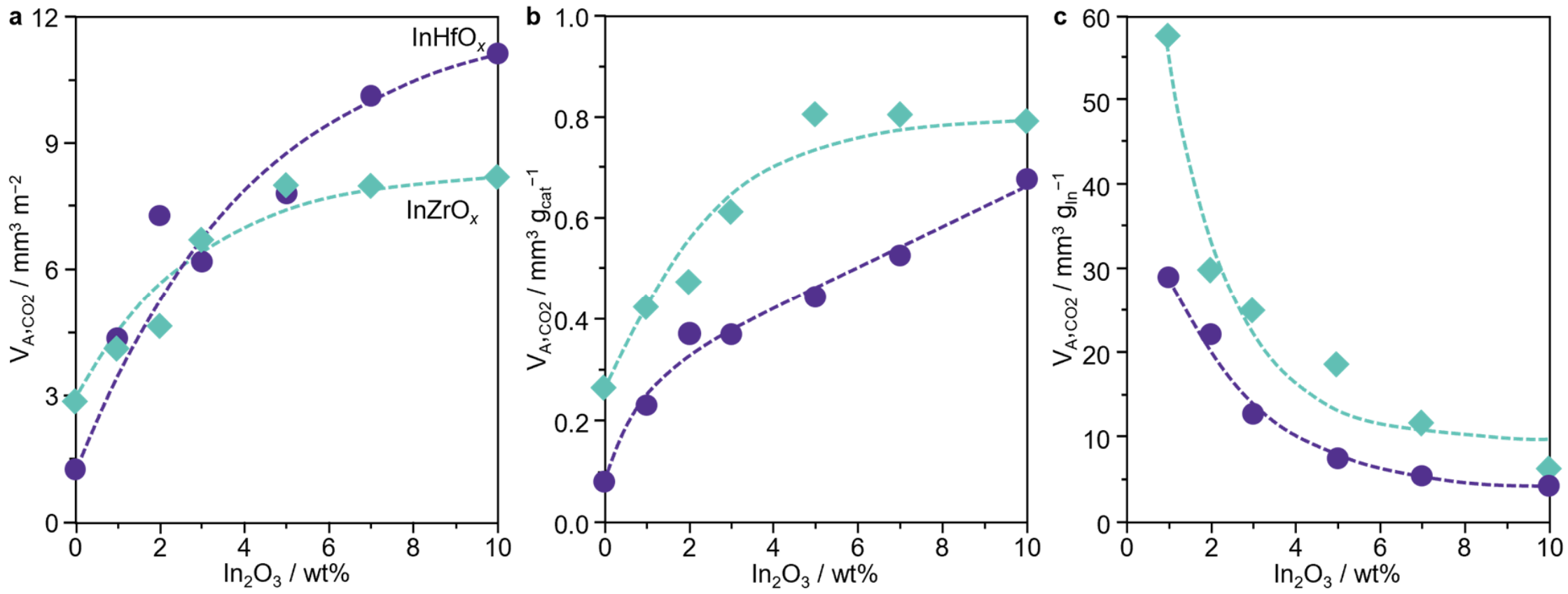

Supplementary Fig. 34. Volumetric CO_2 uptake of InHfO$_x$ and InZrO$_x$ catalysts normalized by **a**, specific surface area, **b**, catalyst mass, and **c**, indium content.

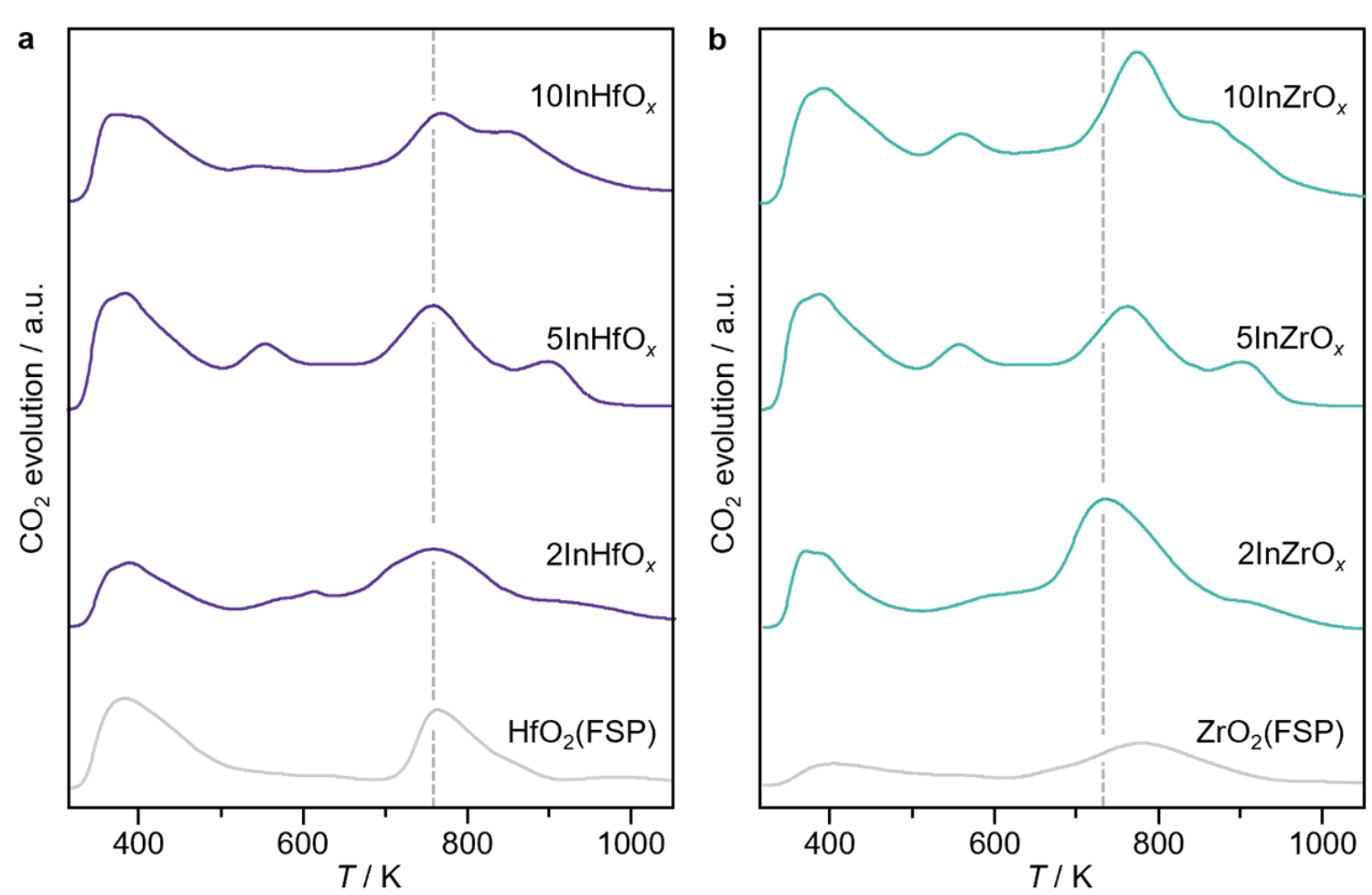

Supplementary Fig. 35. CO_2-TPD profiles of **a**, InHfO$_x$ and **b**, InZrO$_x$ catalysts. Grey lines show the profiles of the corresponding pure support. Vertical dashed lines highlight the change in the position of high-temperature desorption peaks.

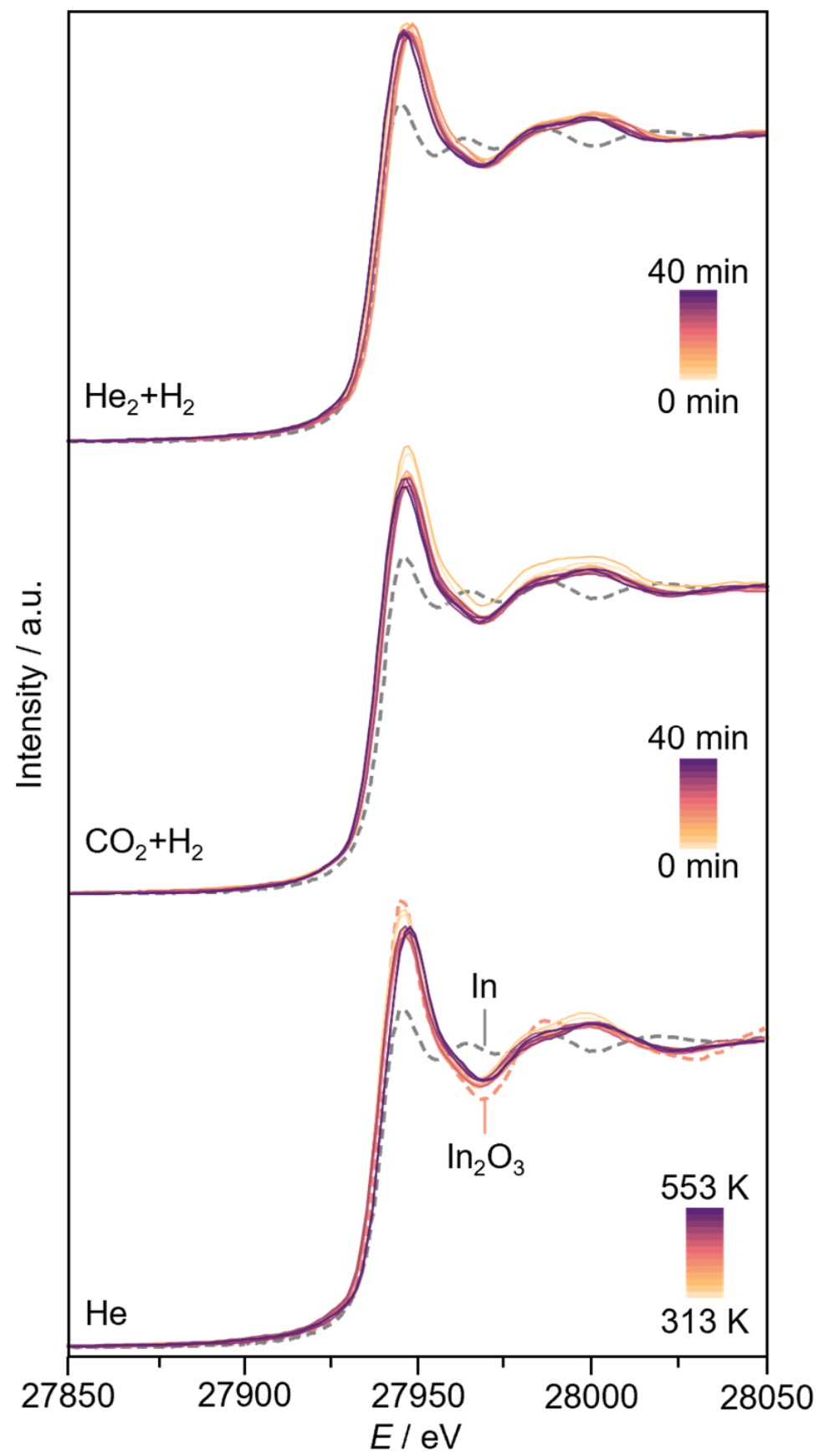

Supplementary Fig. 36. In-situ In *K*-edge XANES spectra of $2InZrO_x$ (m_{cat} = 0.013 g) during heating in He (rate = 5 K min^{-1}, F_T = 20 cm^3 min^{-1}, T = 553 K, P = 15 bar, dwell time = 60 min), under CO_2+H_2 (F_T = 20 cm^3 min^{-1}, T = 553 K, P = 15 bar, H_2/CO_2 = 4, dwell time = 60 min), and under He+H_2 (F_T = 20 cm^3 min^{-1}, T = 553 K, P = 15 bar, H_2/He = 4, dwell time = 60 min).

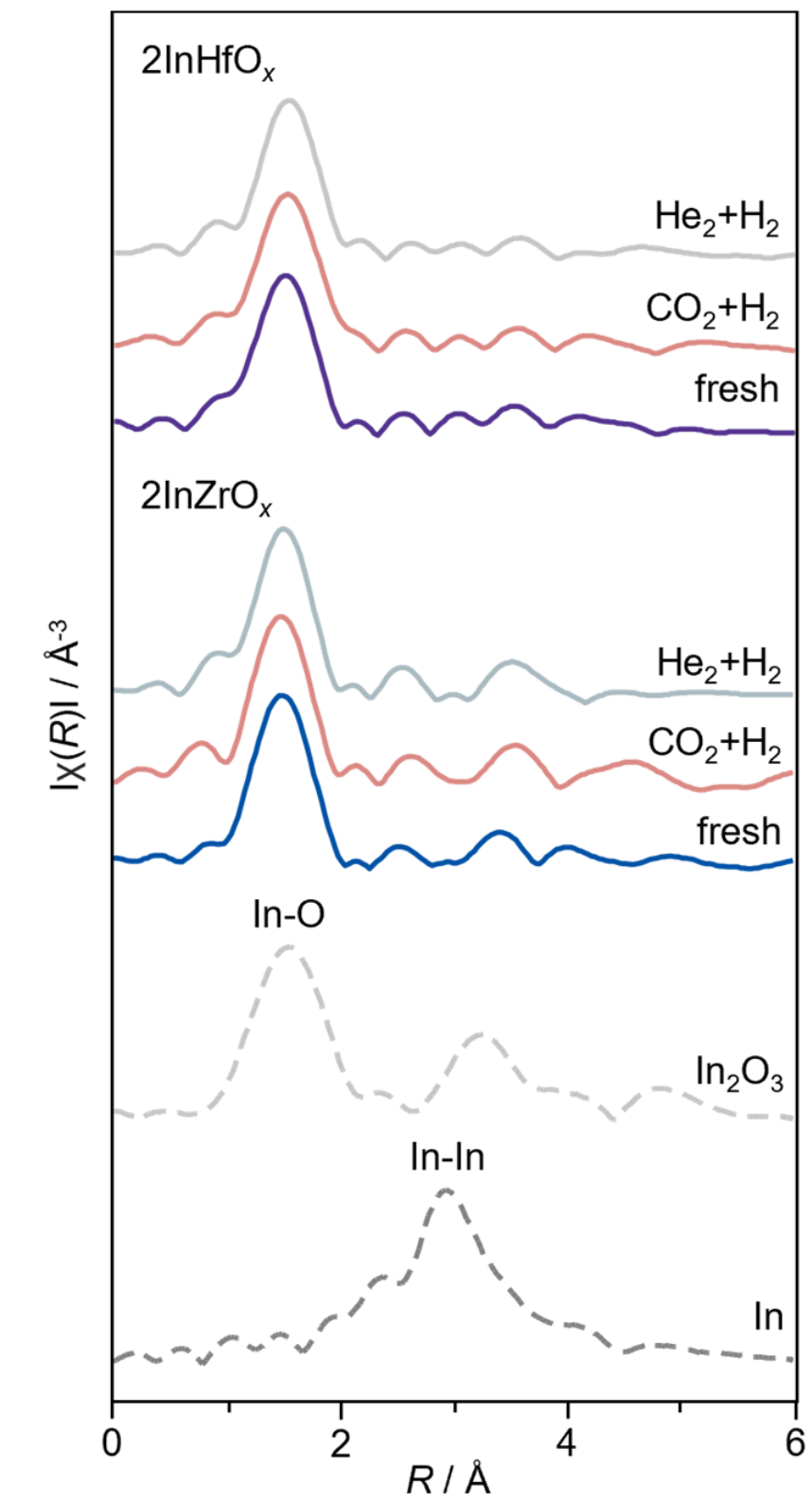

Supplementary Fig. 37. In-situ In *K*-edge EXAFS spectra of 2InHfO$_x$ and 2InZrO$_x$ in fresh form and during treatment in CO_2+H_2 or He+H_2 mixtures. The spectra of bulk In and In_2O_3 are shown for reference.

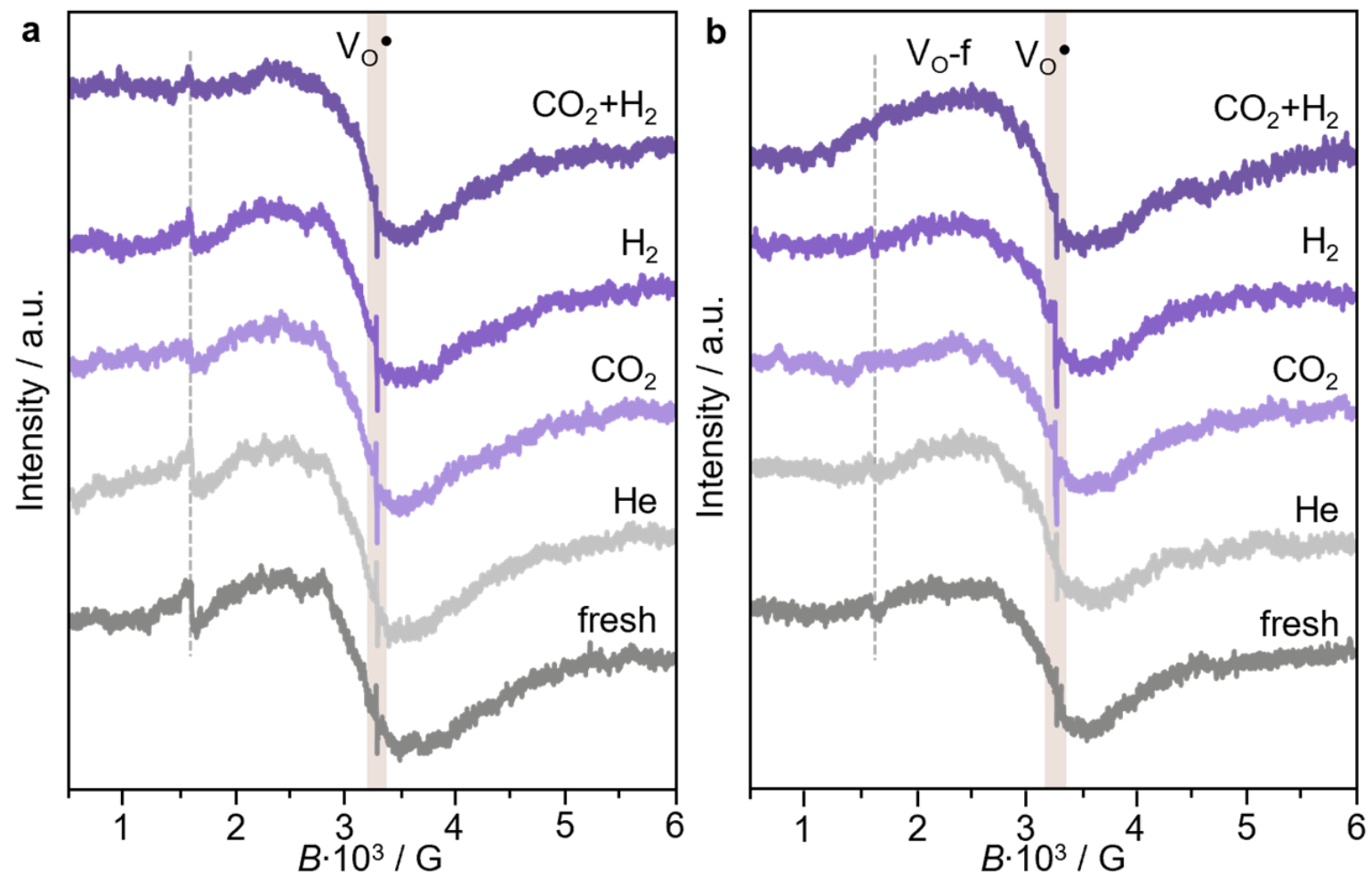

Supplementary Fig. 38. a,**b**, Full in-situ EPR spectra for 2InHfO$_x$ (**a**) and 2InZrO$_x$ (**b**) catalysts, recorded at room temperature and during heating in He (F_T = 210 cm^3 min^{-1}, heating rate = 50 K min^{-1}, T = 553 K, P = 10 bar), after reducing in H_2 (F_T = 210 cm^3 min^{-1}, T = 553 K, P = 10 bar), after exposing to CO_2 (F_T = 110 cm^3 min^{-1}, T = 553 K, P = 10 bar), and after CO_2 hydrogenation (F_T = 200 cm^3 min^{-1}, T = 553 K, P = 10 bar, H_2/CO_2 = 4). The broad signal, attributed to unpaired electrons delocalized over several oxygen vacancies that interact (exchange-coupled vacancy polarons, V_O-f), varies under distinct conditions for 2InZrO$_x$, being most prominent under CO_2+H_2. Dashed lines indicate the linewidth of V_O-f in the fresh catalysts.

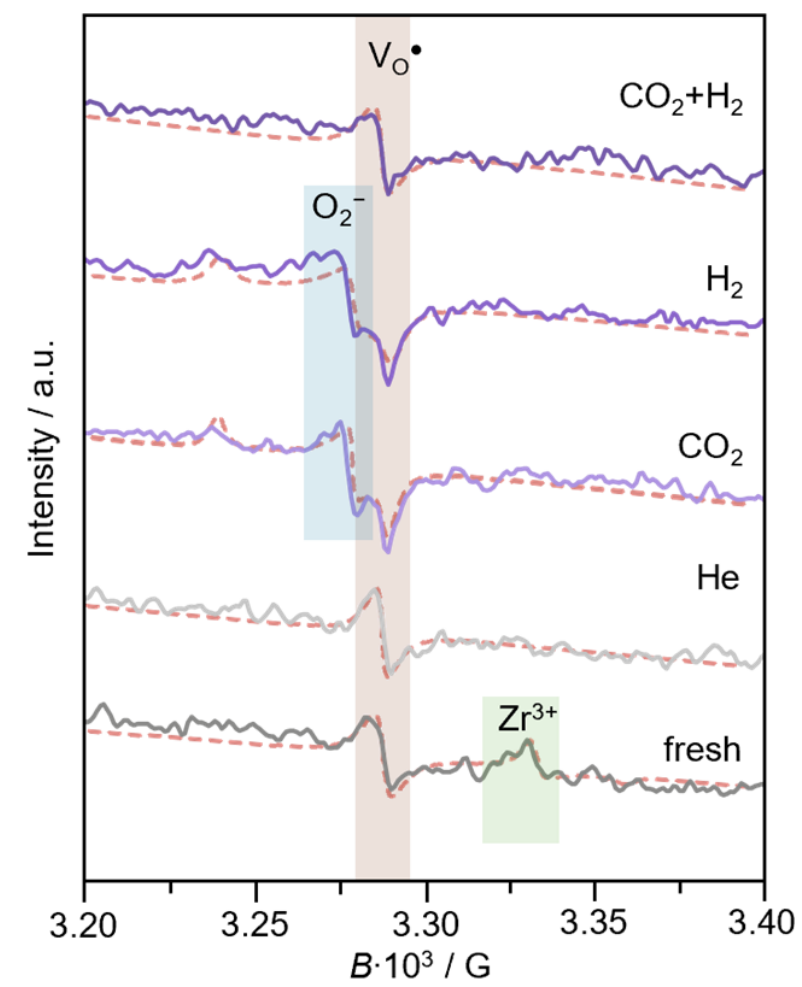

Supplementary Fig. 39. Zoom of the spectra of 2InZrO*x* showing reduced Zr^{3+} species in the fresh state and the emergence of superoxide (O^{2-}) signals under reaction and reducing conditions. Dashed lines denote the fitted results using the particle swarm optimization method.

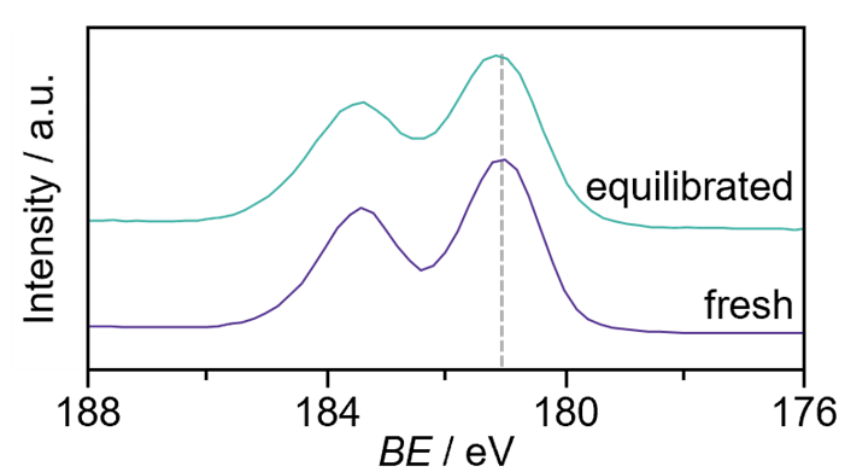

Supplementary Fig. 40. XPS Zr 3*d* core-level spectra of the 2InZrO*x* catalyst in fresh and equilibrated forms. Reaction conditions as described in **Supplementary Fig. 2**.

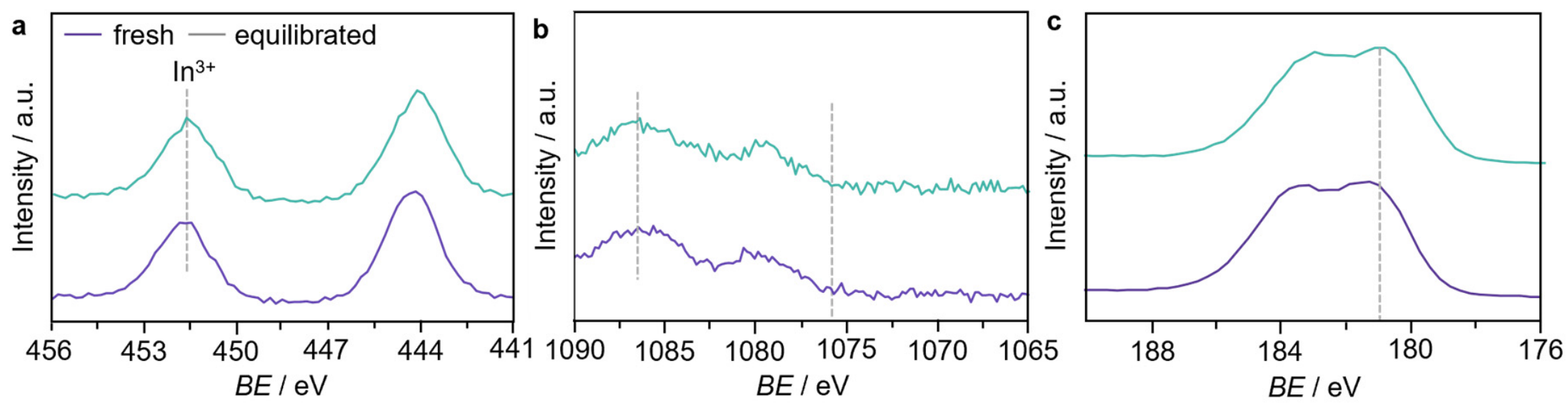

Supplementary Fig. 41. a, In 3*d* and **b**, In MNN XPS spectra of fresh and equilibrated $5InHfO_x$ catalysts. **c**, Hf 4*f* XPS core-level spectra of $5InHfO_x$ catalyst. Reaction conditions as described in **Supplementary Fig. 2**.

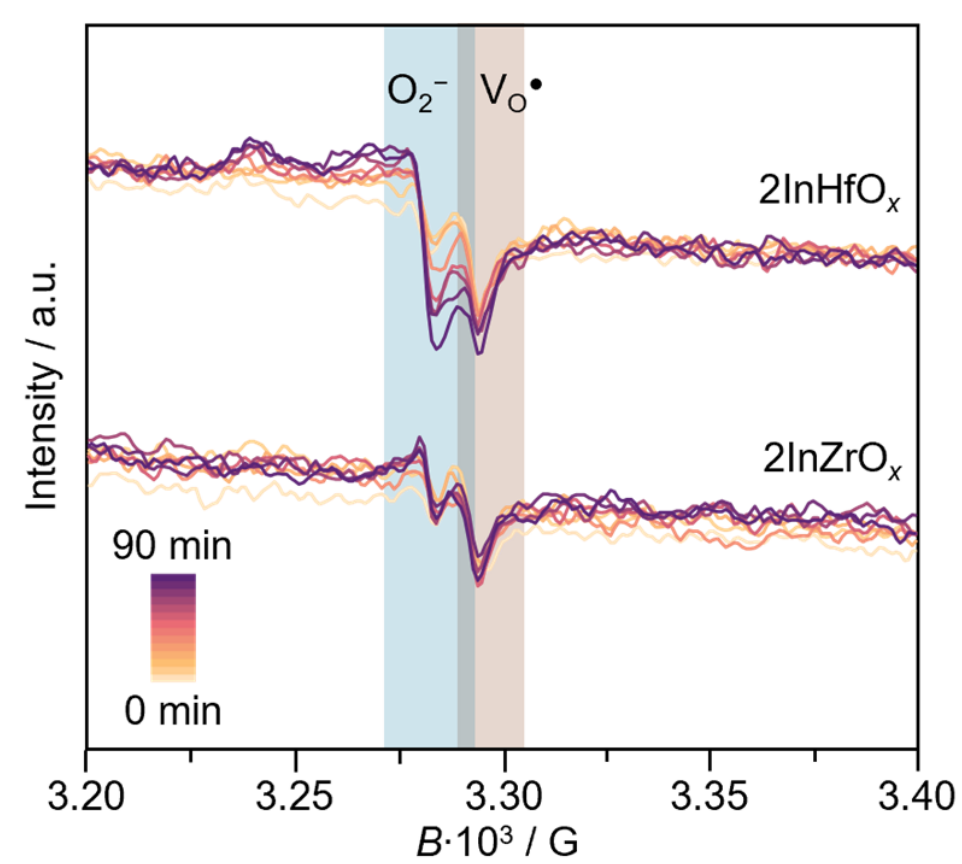

Supplementary Fig. 42. Paramagnetic signals from EPR spectroscopy of $2InHfO_x$ and $2InZrO_x$ catalysts following in-situ measurement and exposure to air. Highlighted regions indicate superoxide (O^{2-}) signals, evidencing the presence of surface electrons, with a stronger signal for $2InHfO_x$.

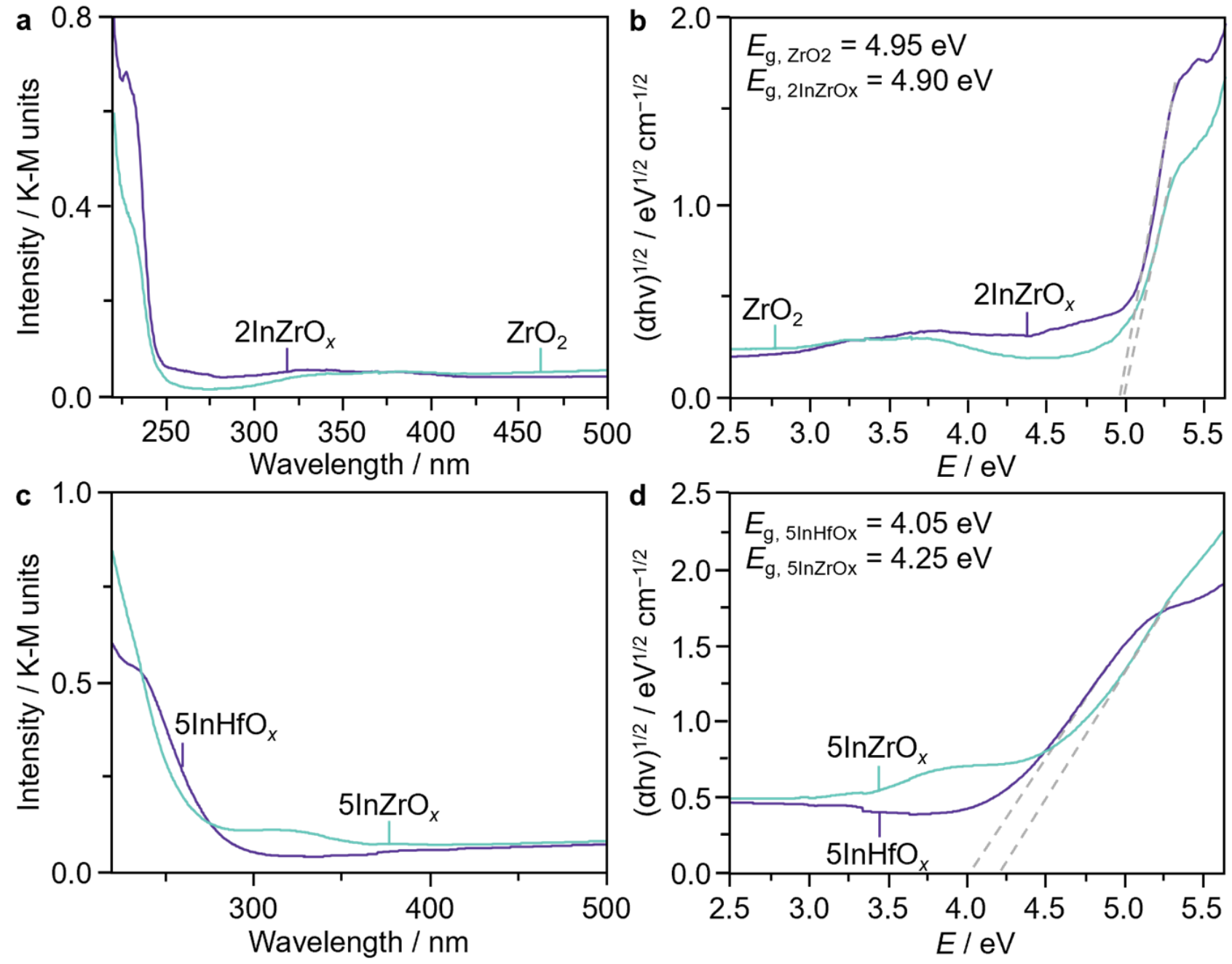

Supplementary Fig. 43. DR-UV-vis spectra of **a**, ZrO_2 and 2InZrO_x catalyst and **b**, 5InHfO_x and 5InZrO_x catalysts. **c**,**d**, Corresponding Tauc plots for bandgap determination.

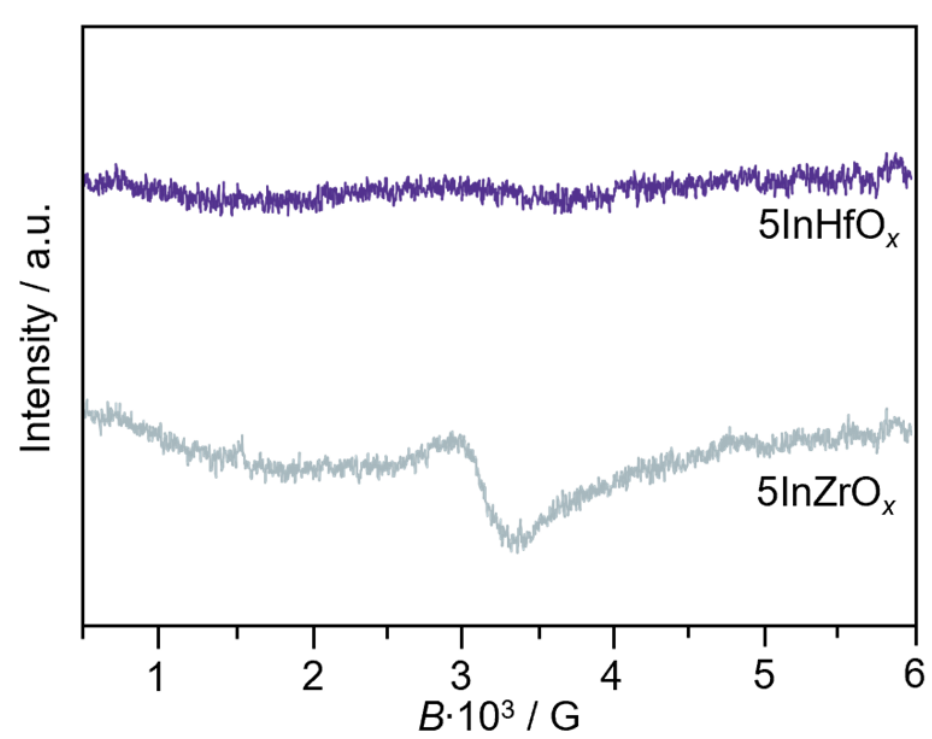

Supplementary Fig. 44. Ex-situ EPR spectra of 5InHfO_x and 5InZrO_x catalysts. Broad ferromagnetic signals in 5InZrO_x arise from electron hopping between oxygen vacancies and Zr 3*d* orbitals. For 5InHfO_x, these signals are further broadened and suppressed as the energy level of oxygen vacancies overlaps with the conduction band, allowing electron transitions to Hf 4*f* orbitals, indicative of greater electronic coupling.

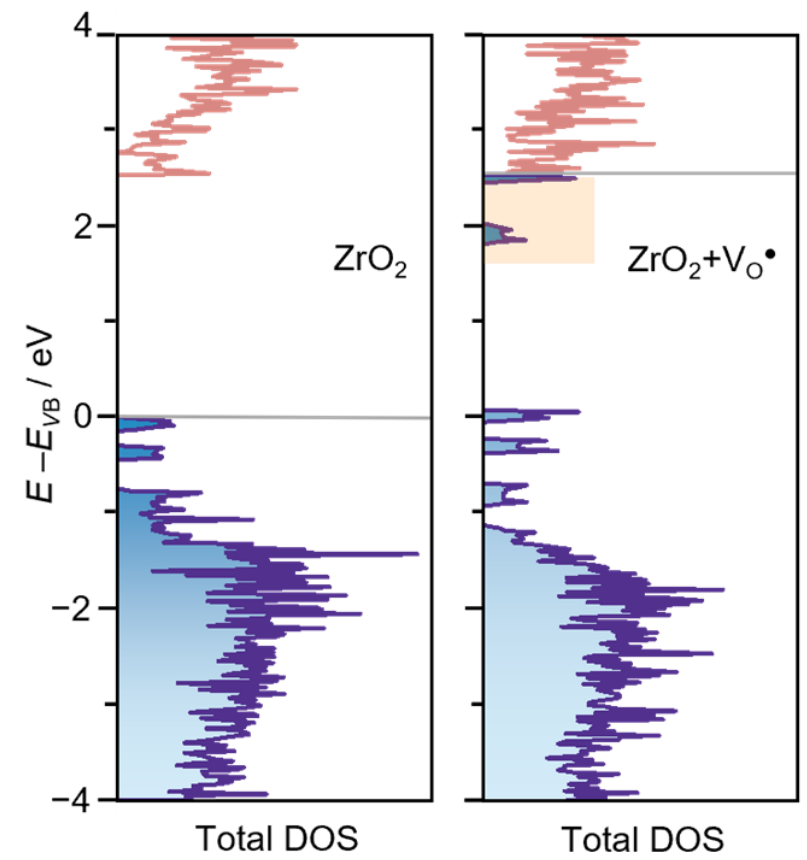

Supplementary Fig. 45. PBE-computed density of states (DOS) for pristine m-ZrO_2 and m-ZrO_2 with one vacancy. The DOS are aligned with respect to the valence band of the pristine support oxide. The valence band is marked in grey, with the states arising from excess electrons highlighted with red dotted circles. Upon oxygen vacancy formation, electronic states appear in the band gap and near the conduction band, which are associated with the formation of a Zr^{3+} and one electron stored in the oxygen vacancy, respectively.

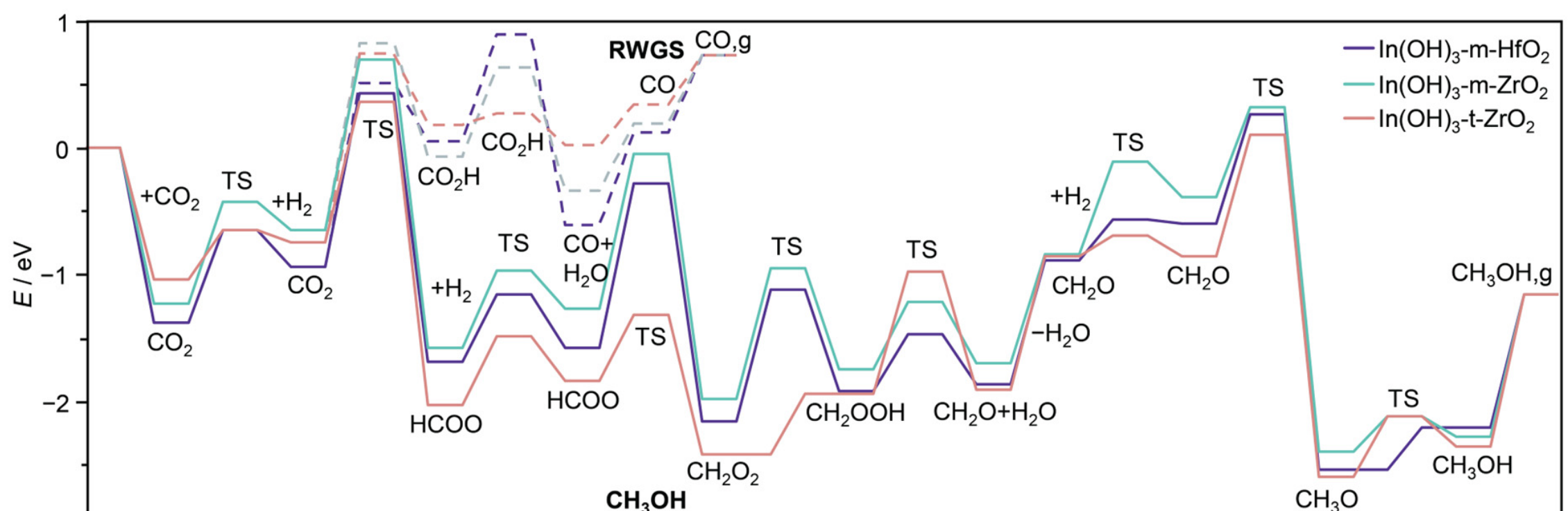

Supplementary Fig. 46. Reaction energy profiles for CO_2 hydrogenation to methanol (solid lines) and the competing RWGS reactions (dashed line) without Gibbs free energy correction on $In(OH)_3$-m-HfO_2, $In(OH)_3$-m-ZrO_2, and $In(OH)_3$-t-ZrO_2 model surfaces.

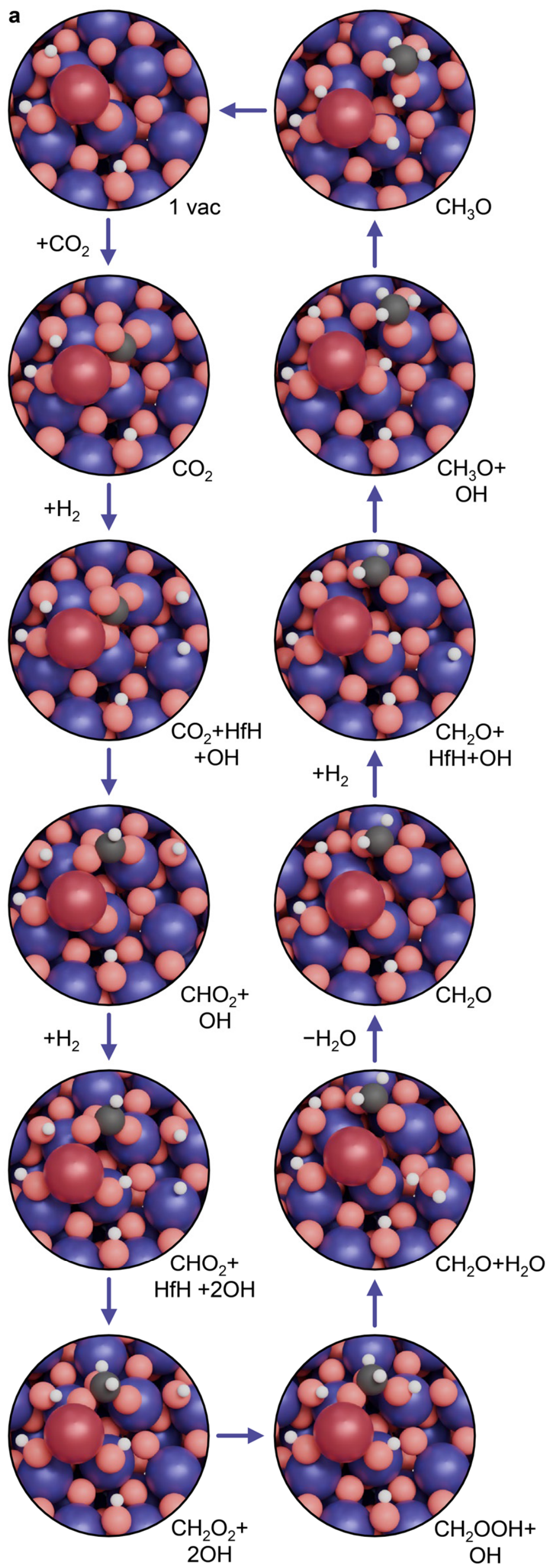

Supplementary Fig. 47. Intermediates for CO_2 conversion to **a**, methanol and **b**, CO over $In(OH)_3$-m-HfO_2 containing one vacancy. Colour code: Hf - purple; In - red; O - salmon pink; C - dark grey; H - white.

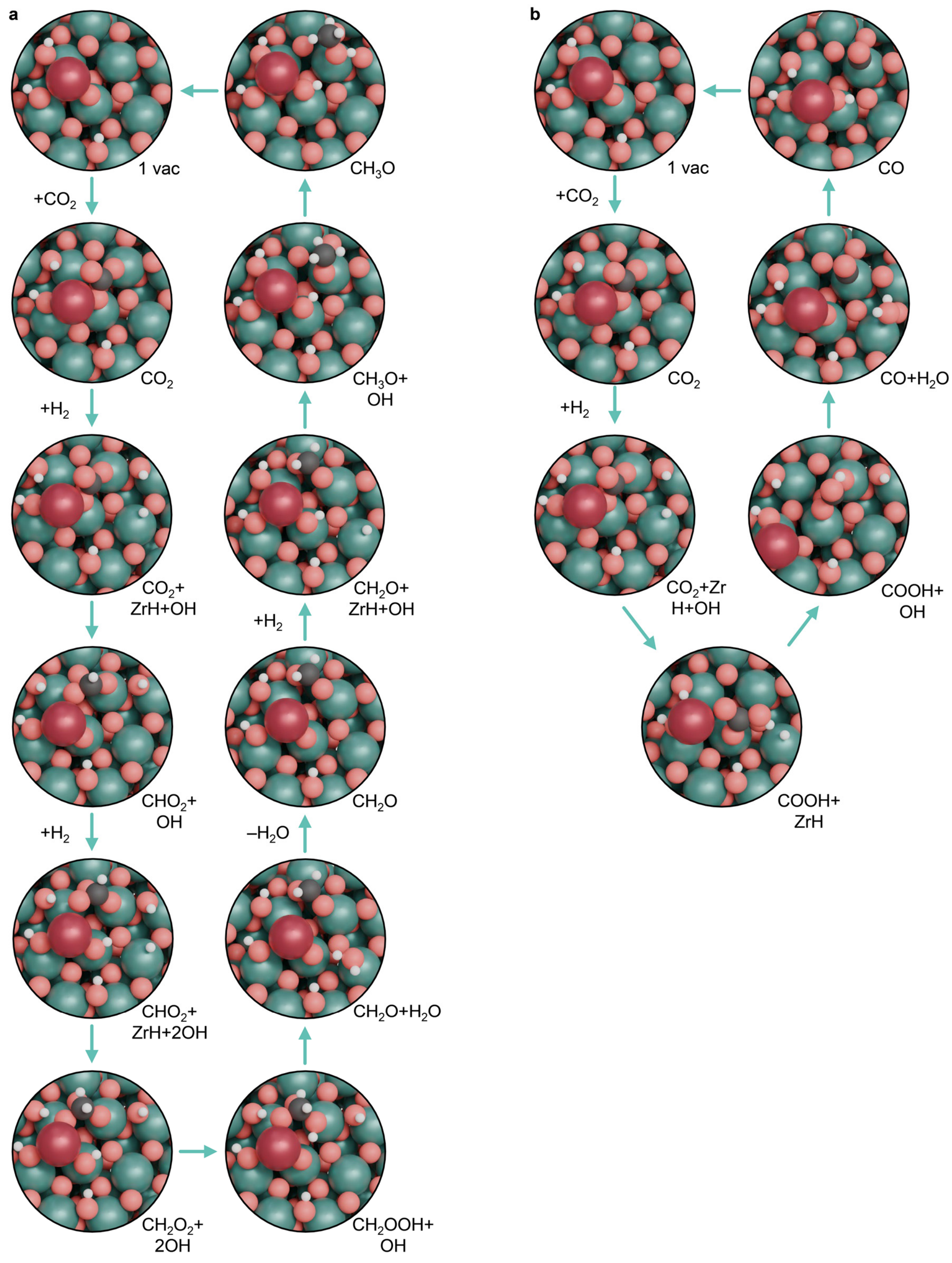

Supplementary Fig. 48. Intermediates for CO_2 conversion to **a**, methanol and **b**, CO over $In(OH)_3$/m-ZrO_2 containing one vacancy. Colour code: Zr - teal; In - red; O - salmon pink; C - dark grey; H - white.

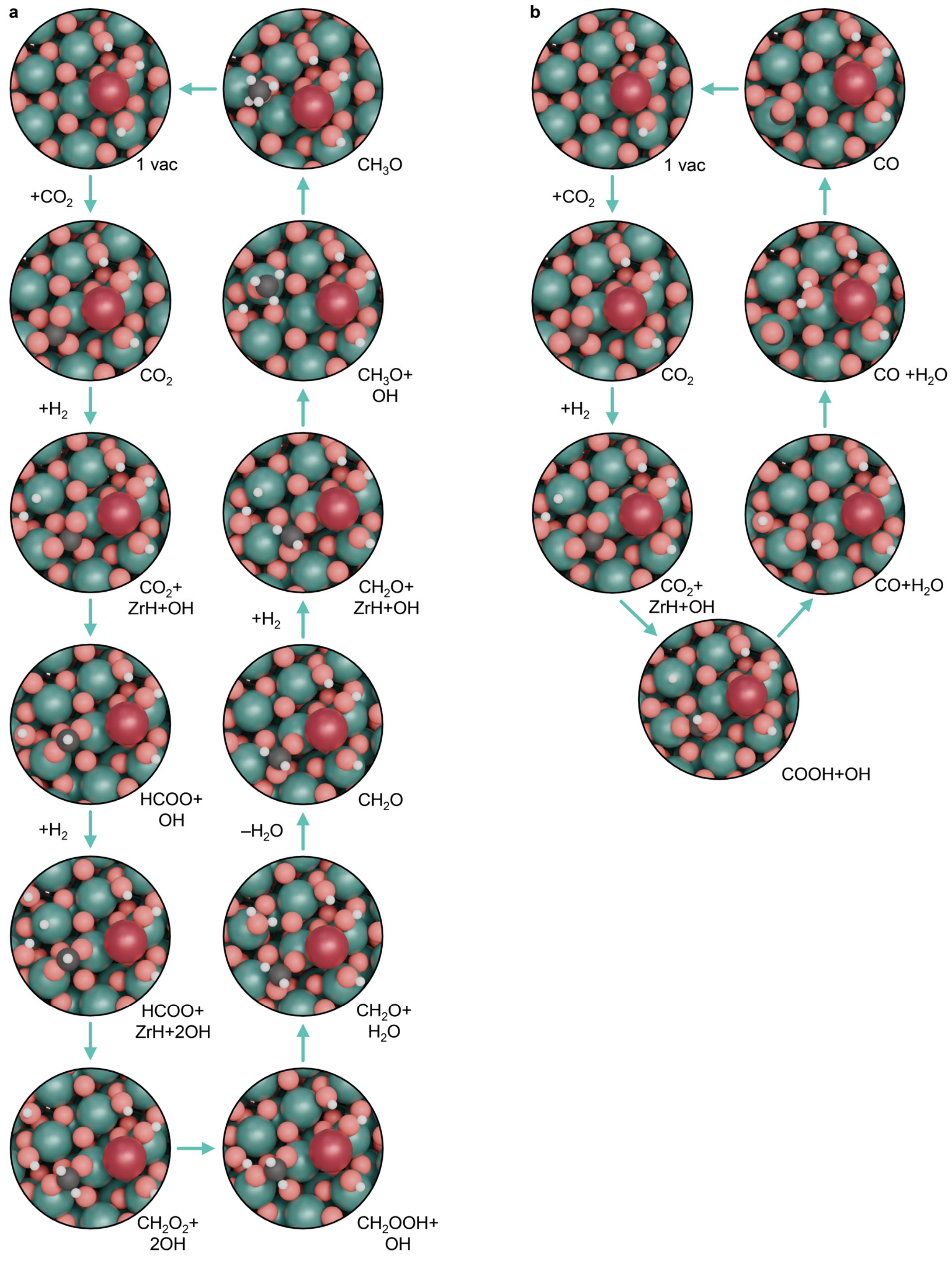

Supplementary Fig. 49. Intermediates for CO_2 conversion to **a**, methanol and **b**, CO over $In(OH)_3$/t-ZrO_2 containing one vacancy. Colour code: Zr - teal; In - red; O - salmon pink; C - dark grey; H - white.

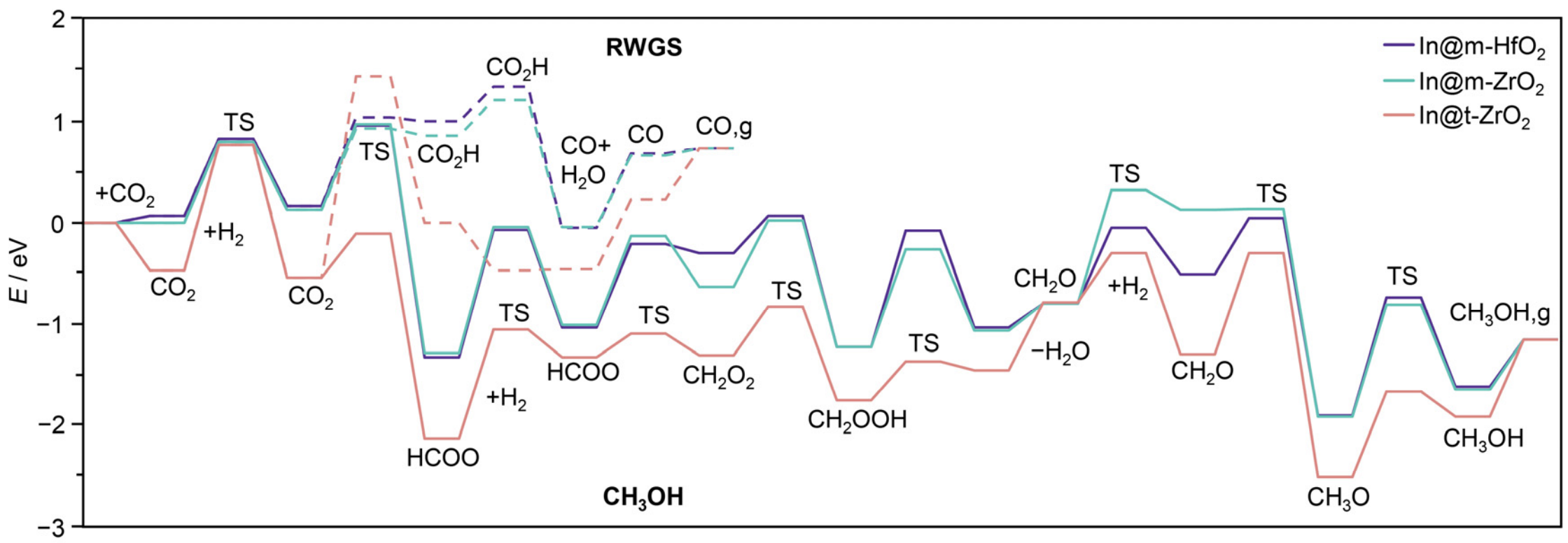

Supplementary Fig. 50. PBE-computed reaction energy profile for CO_2 hydrogenation to methanol (solid lines) and the competing RWGS reactions (dashed line), on m-HfO_2, m-ZrO_2, and t-HfO_2 models containing an incorporated indium atom and one oxygen vacancy.

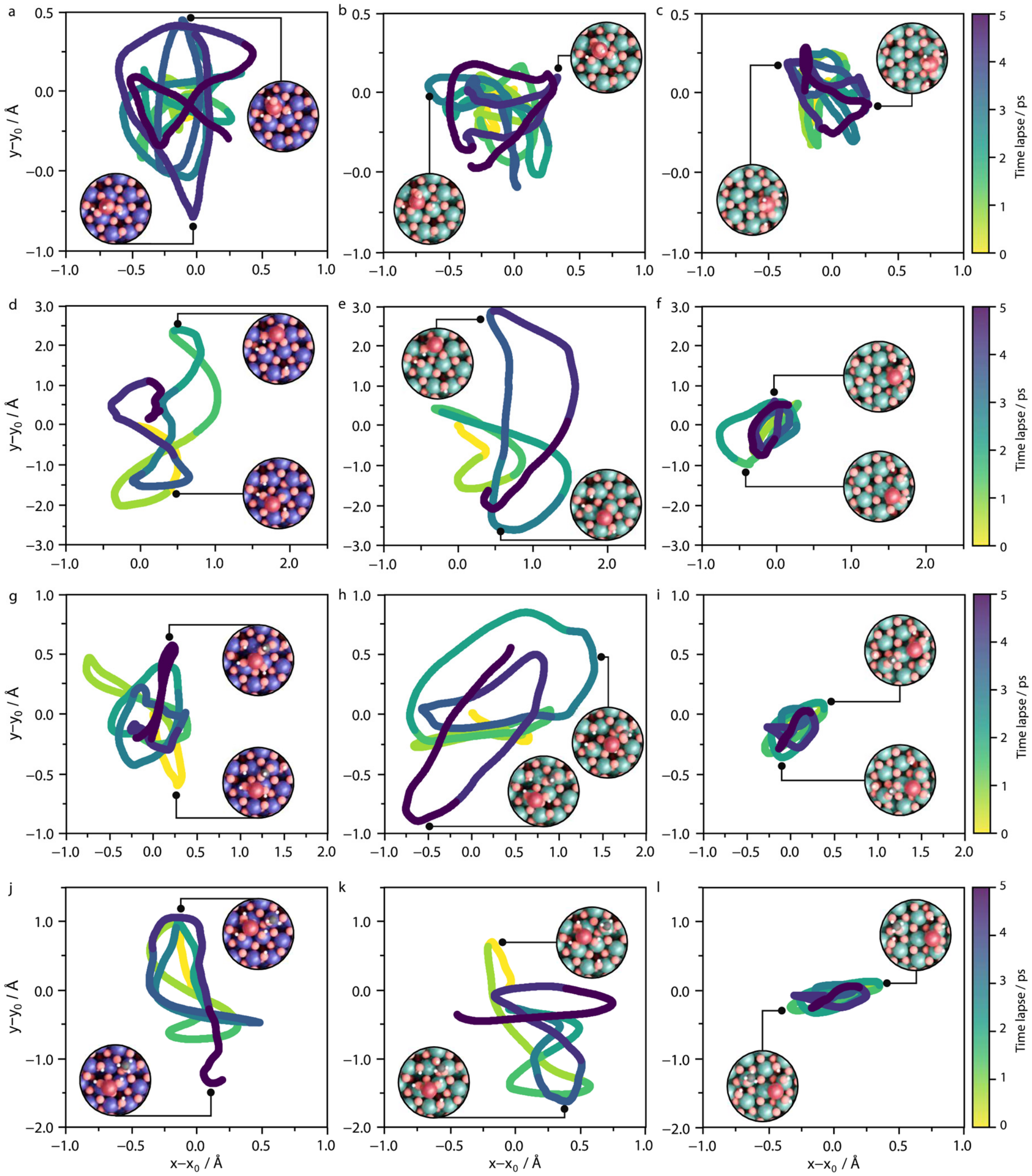

Supplementary Fig. 51. Trajectories of In single atom from ab initio molecular dynamics (AIMD) simulations (**Supplementary Videos 1-3**) for **a**, $In(OH)_3$-m-HfO_2, **d**, $In(OH)_3$-m-HfO_2 with one oxygen vacancy, **g**, with a vacancy and adsorbed HCOO intermediate, and **j**, with a vacancy and adsorbed CH_3O intermediate. Panels **b**,**e**,**h**,**k** present the corresponding $In(OH)_3$--m-ZrO_2 structures, and panels **c**,**f**,**i**,**l** those for $In(OH)_3$-t-ZrO_2. Simulations were conducted with a temperature ramp from 0 K to 553 K over 5 ps in the NVT ensemble with Nosé-Hoover thermostat and a time step of 1 fs.

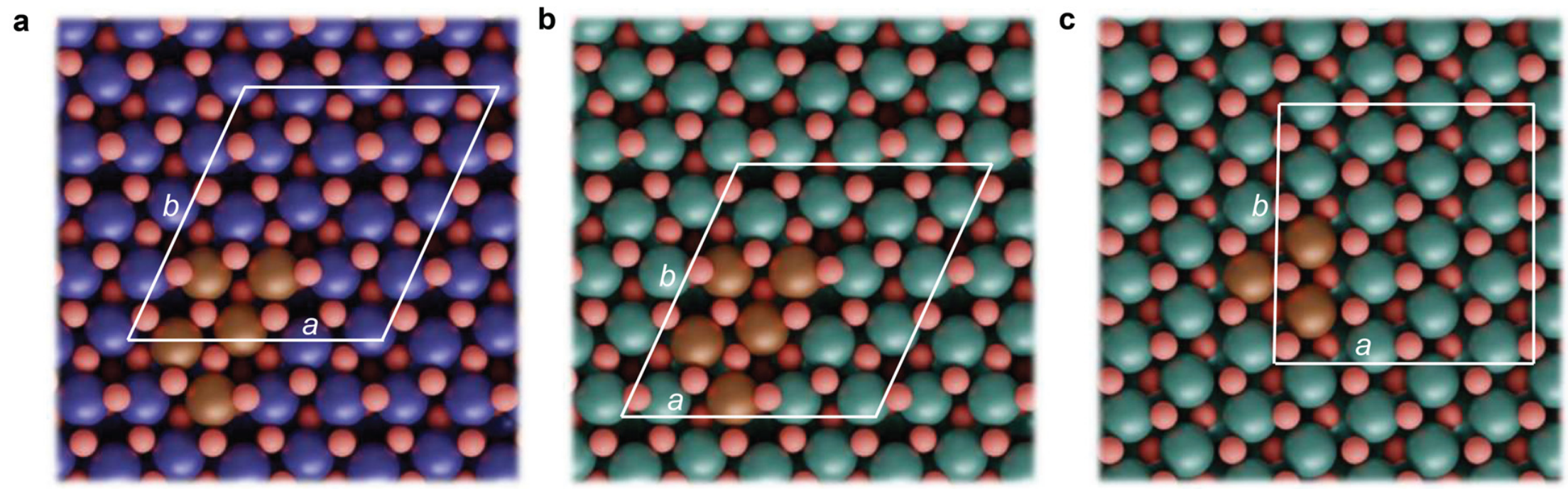

Supplementary Fig. 52. Top-view projections highlight in brown the metal atoms (Hf or Zr) bonded to In during the MD run (**Supplementary Videos 1-3**). The white line corresponds to the AIMD cell. Simulations were conducted with a temperature ramp from 0 K to 553 K over 5 ps in the NVT ensemble with Nosé-Hoover thermostat and a time step of 1 fs.

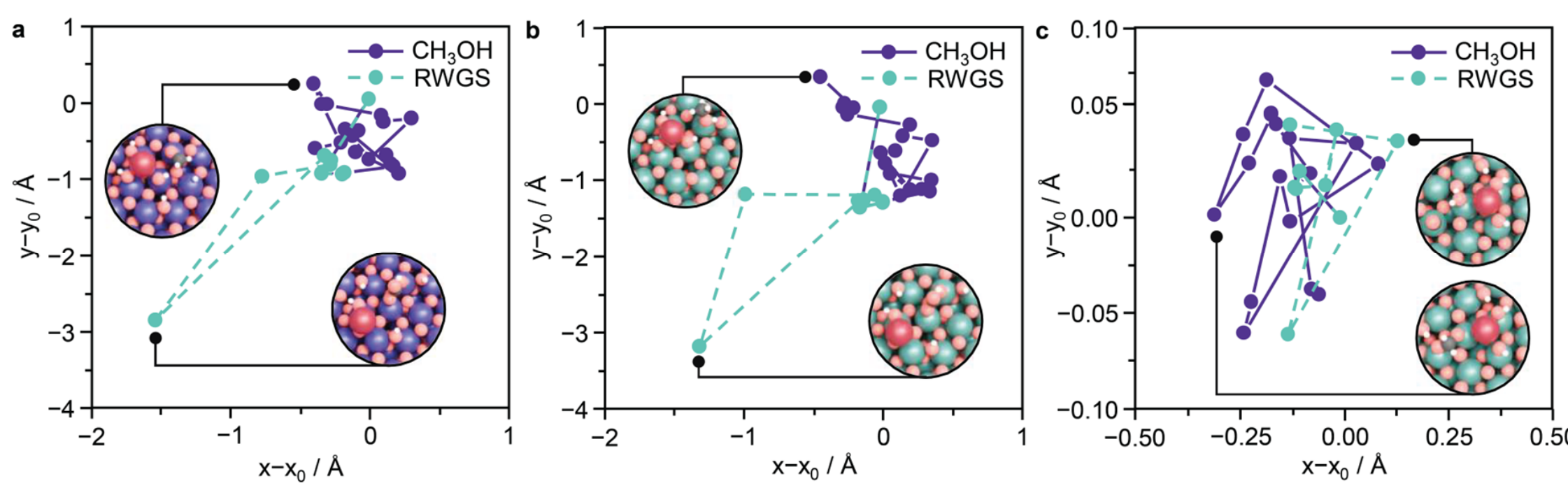

Supplementary Fig. 53. Trajectories of indium single atoms during the CO_2 hydrogenation reaction for **a**, $In(OH)_3$-m-HfO_2 **b**, $In(OH)_3$-m-ZrO_2, and $In(OH)_3$-t-ZrO_2 models.

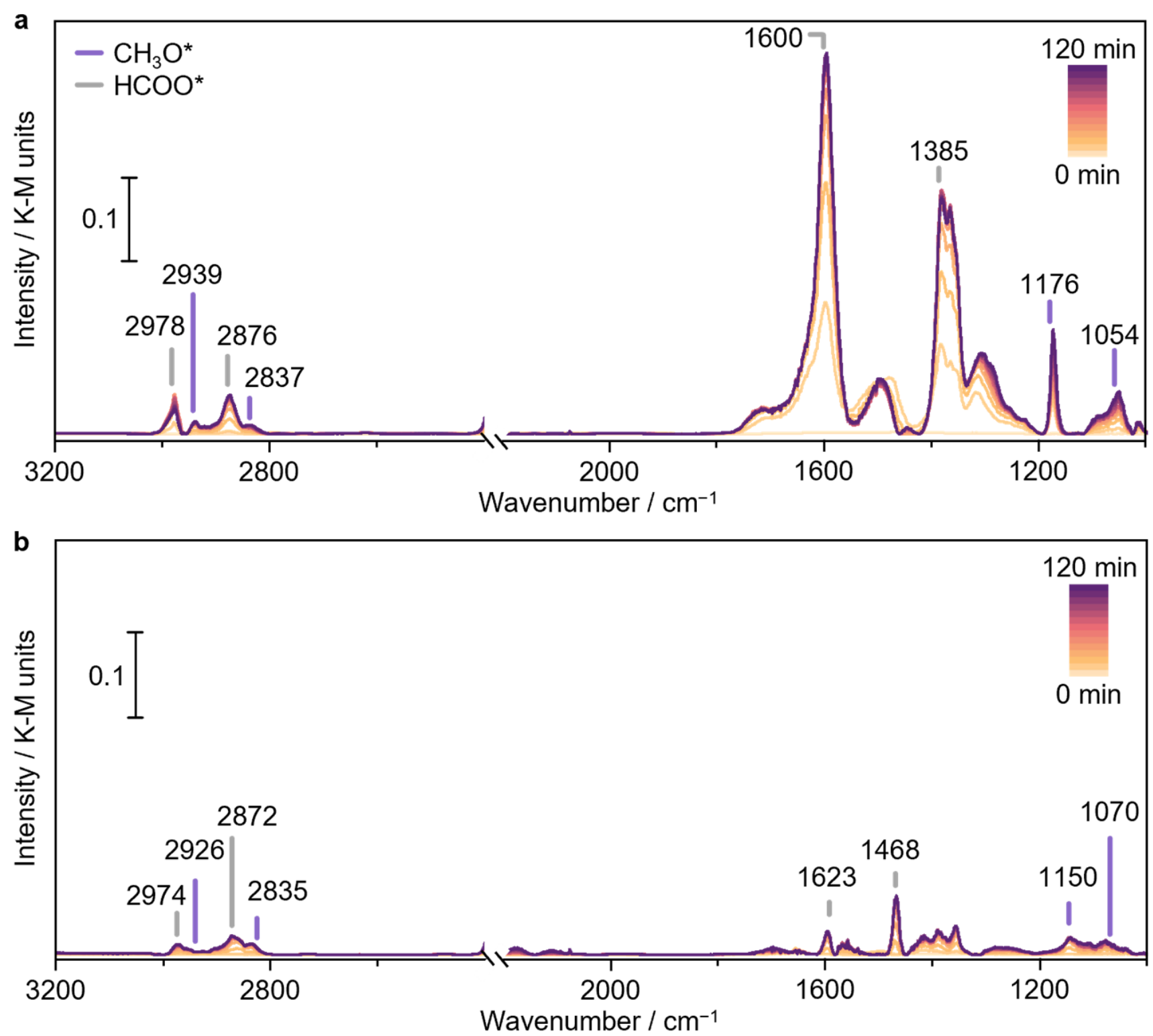

Supplementary Fig. 54. In-situ DRIFT spectra of **a**, 2InHfO_x and **b**, 2InZrO_x under CO_2 hydrogenation conditions ($F_T = 20$ cm^3 min^{-1}, $T = 553$ K, $P = 20$ bar, $H_2/CO_2 = 4$, dwell time = 120 min).

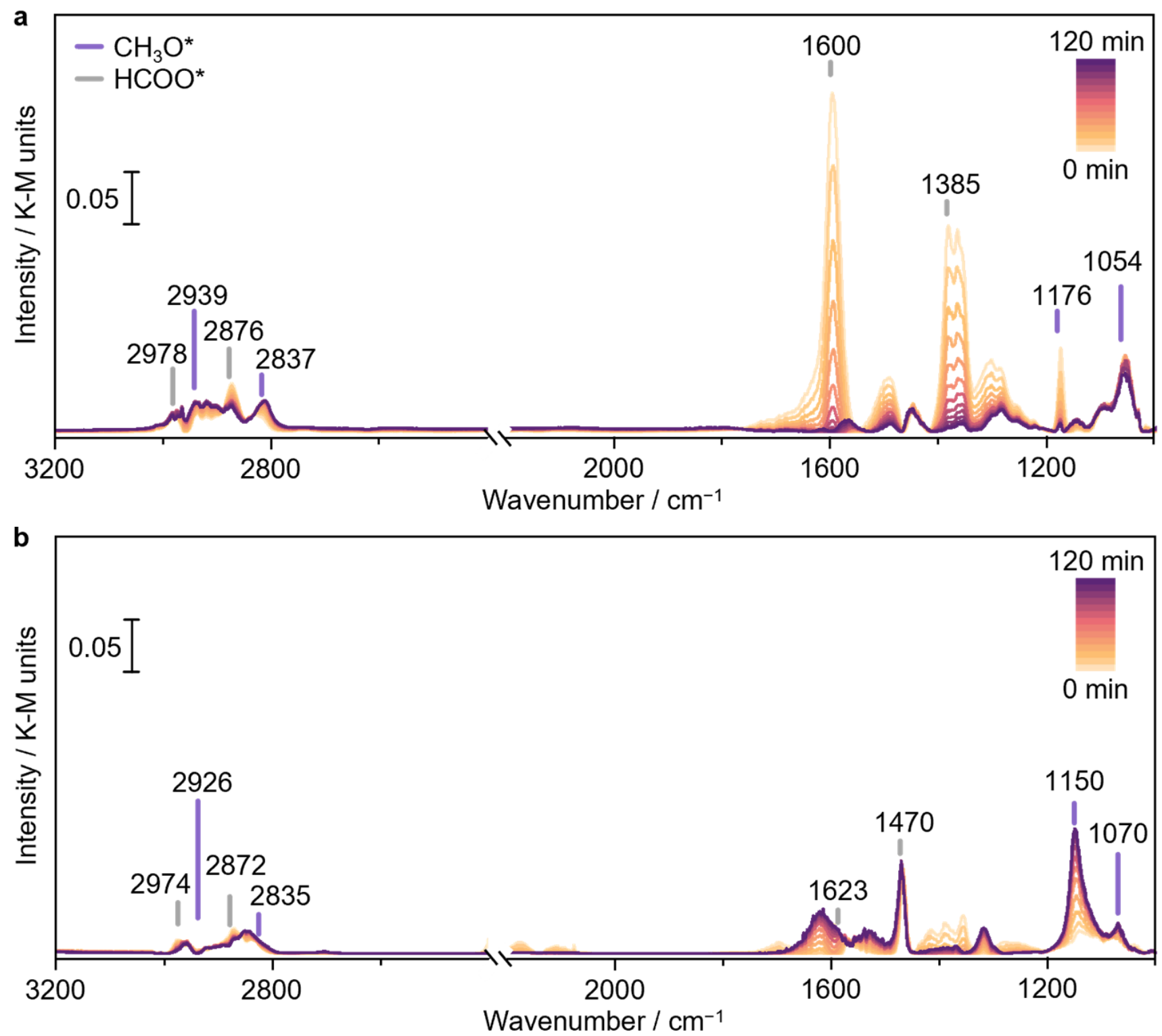

Supplementary Fig. 55. In-situ DRIFT spectra of **a**, 2InHfO$_x$ and **b**, 2InZrO$_x$ after switching to H_2 balanced with He (F_T = 20 cm^3 min^{-1}, T = 553 K, P = 20 bar, H_2/He = 4, dwell time = 120 min).

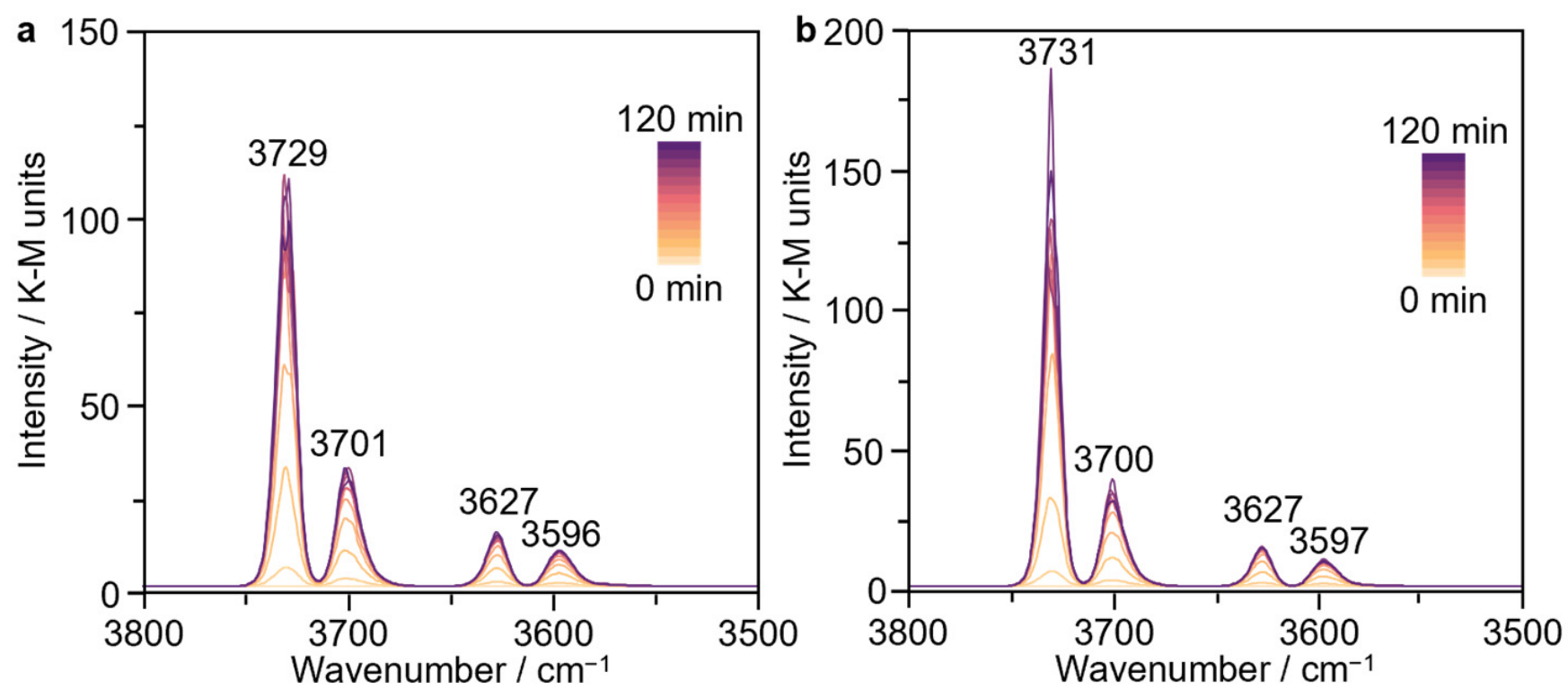

Supplementary Fig. 56. Enlarged hydroxyl region of the in-situ DRIFT spectra of **a**, 2InHfO$_x$ and **b**, 2InZrO$_x$ under CO_2 hydrogenation conditions (F_T = 20 cm^3 min^{-1}, T = 553 K, P = 20 bar, H_2/CO_2 = 4, dwell time = 120 min), confirming the formation of surface hydroxyl groups during reaction.

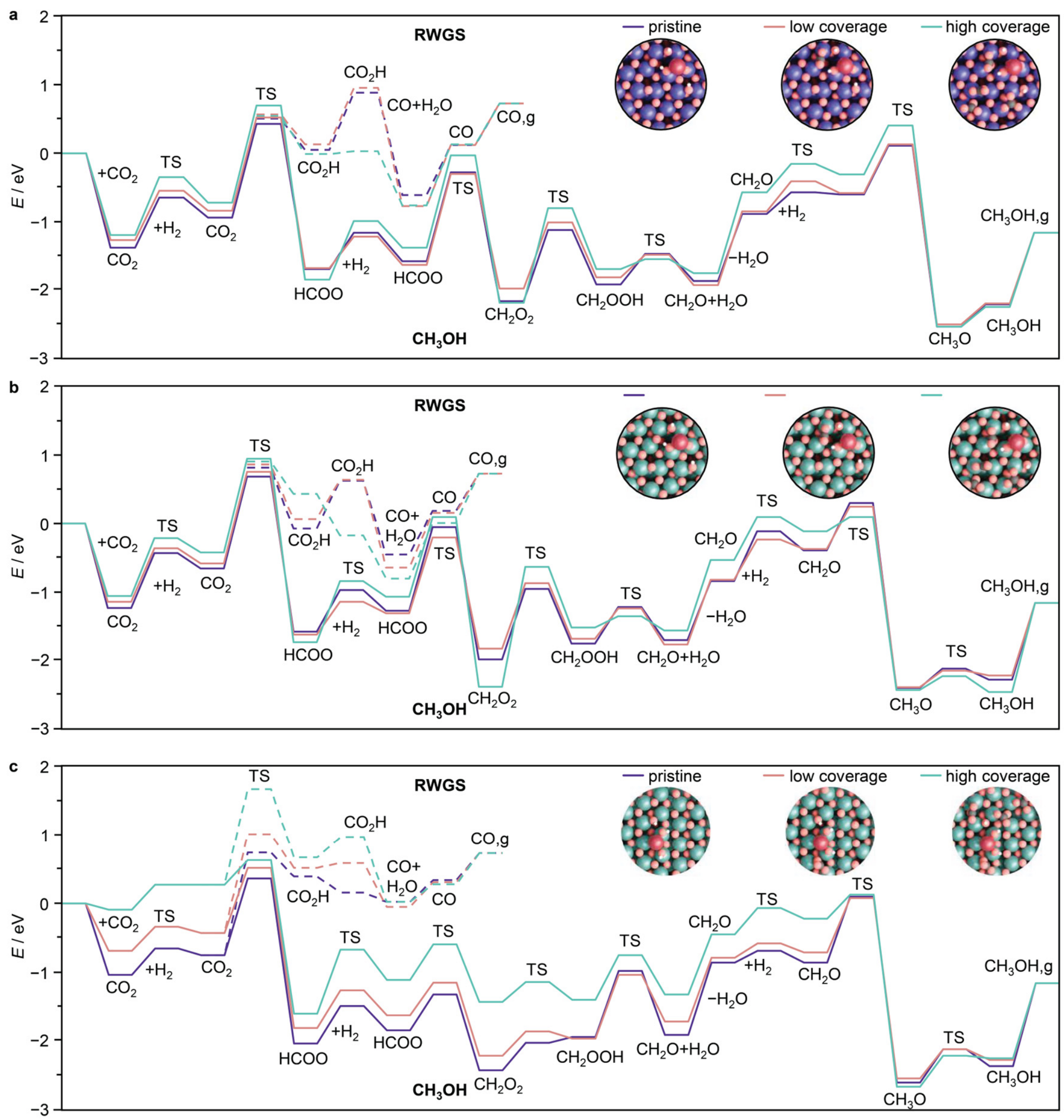

Supplementary Fig. 57. PBE-computed reaction energy profiles for CO_2 hydrogenation on oxygen-deficient **a**, $In(OH)_3$-m-HfO_2 **b**, $In(OH)_3$-m-ZrO_2, and **c**, $In(OH)_3$-t-ZrO_2 systems with carbonate spectator species at low (one CO_2 molecule) and high coverages (four CO_2 molecules). At low coverage, the spectator carbonate was placed on the most stable site near the active site. For high coverage, we added the maximum number of spatially allowed chemisorbed CO_2 molecules, preserving always the most stable configurations.

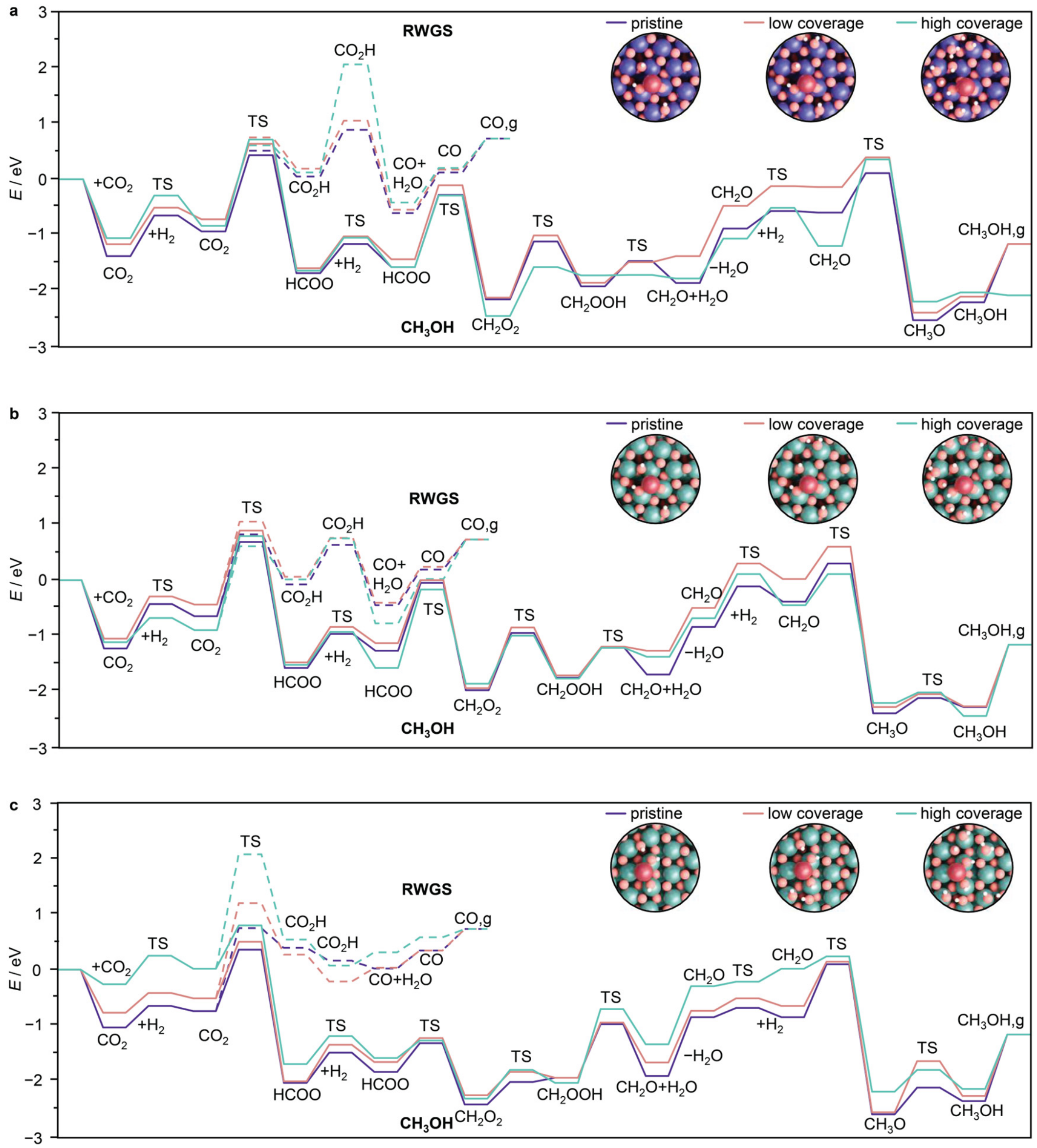

Supplementary Fig. 58. PBE-computed reaction energy profiles for CO_2 hydrogenation on oxygen-deficient **a**, In(OH)$_3$-m-HfO$_2$ **b**, In(OH)$_3$-m-ZrO$_2$, and **c**, In(OH)$_3$-t-ZrO$_2$ systems with water dissociated (as a proxy of hydroxyl groups) to simulate low (one H_2O molecule) and high coverages (eight H_2O molecules) of spectator species. The dissociated water at low coverage was placed on the most stable site near the active site. For high coverage, we added the maximum number of spatially allowed chemisorbed dissociated H_2O molecules, preserving the most stable configurations. In some systems, some dissociated water molecules spontaneously

evolved to molecularly adsorbed water without a significant impact on the CO_2 hydrogenation energy profiles.

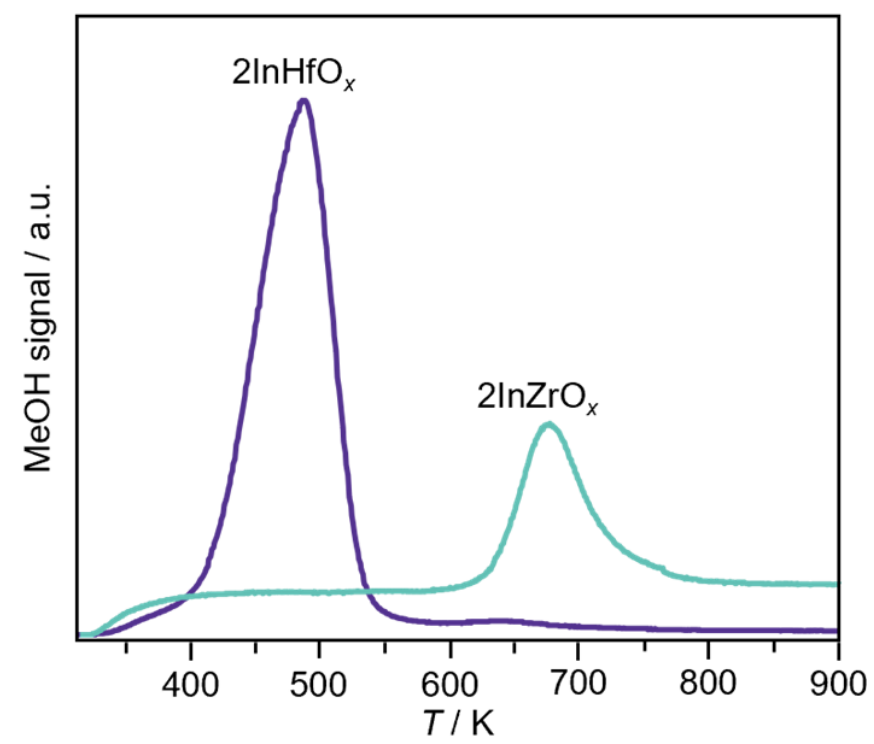

Supplementary Fig. 59. MeOH-TPD profiles of fresh $2InHfO_x$ and $2InZrO_x$ catalysts.

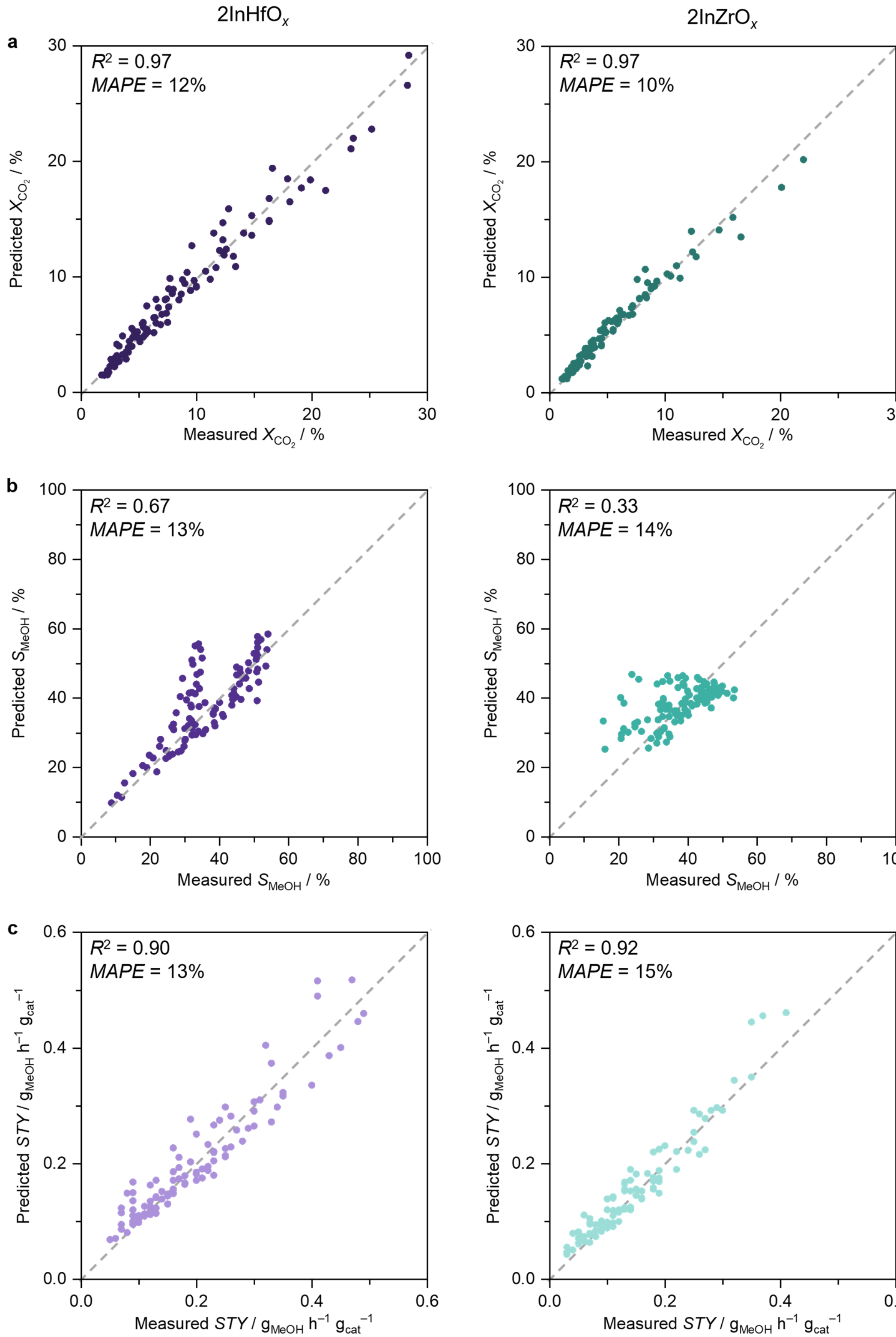

Supplementary Fig. 60. Parity plots comparing **a**, X_{CO2}, **b**, S_{MeOH}, **c**, STY during CO_2 hydrogenation over 2InHfO$_x$ and 2InZrO$_x$ predicted by the two-site LHHW kinetic model (**Supplementary Table 13**) versus experimental data. Mean absolute percentage errors

($MAPE$) and coefficients of determination (R^2) are provided for each metric. Dashed lines indicate the identity line ($x = y$).

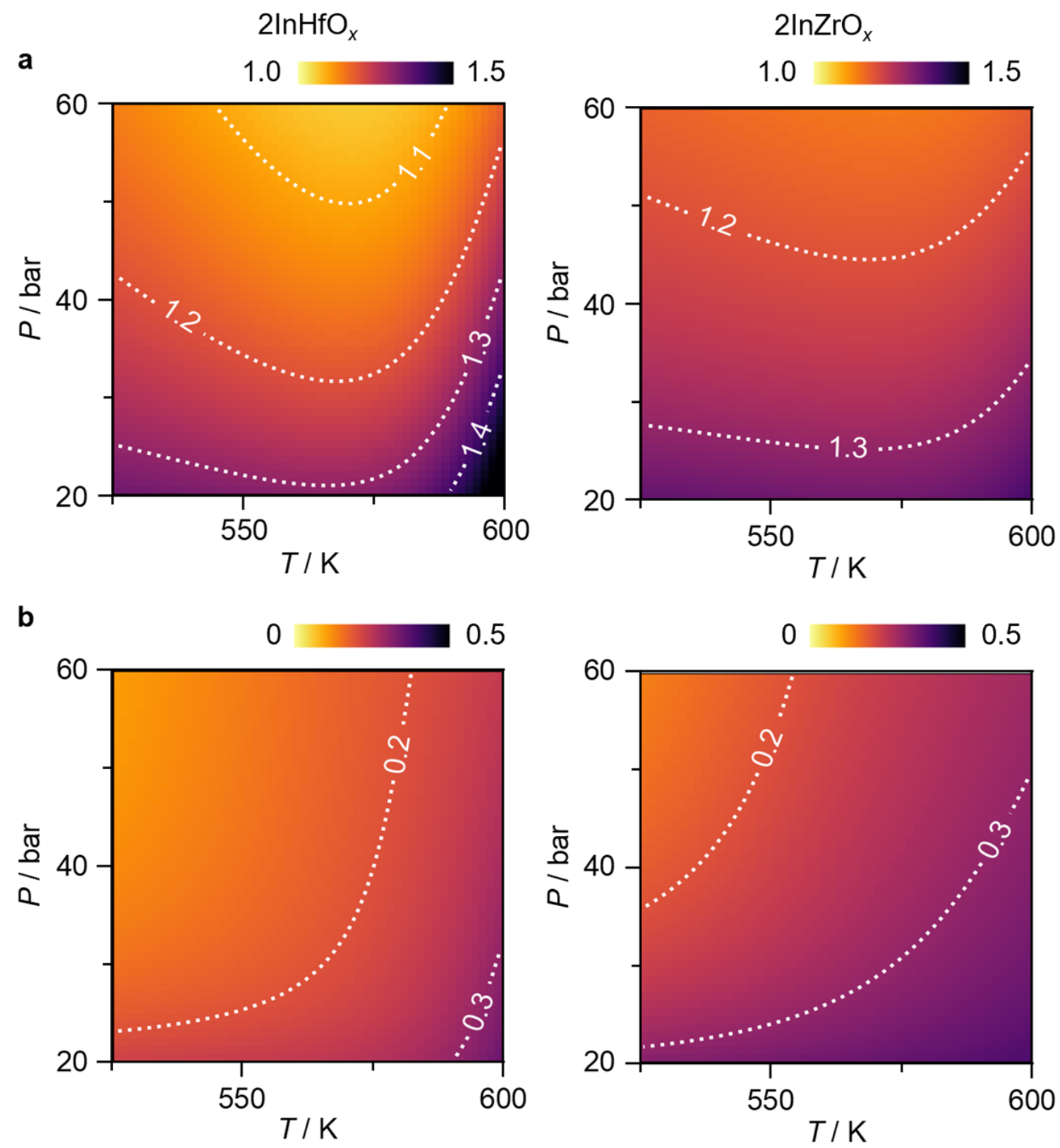

Supplementary Fig. 61. Apparent reaction orders with respect to **a**, H_2 and **b**, CO_2 for methanol formation as a function of T and P at $GHSV$ = 24,000 cm^3 h^{-1} g$_{cat}$$^{-1}$, for 2InHfO$_x$ and 2InZrO$_x$ as calculated by the kinetic model.

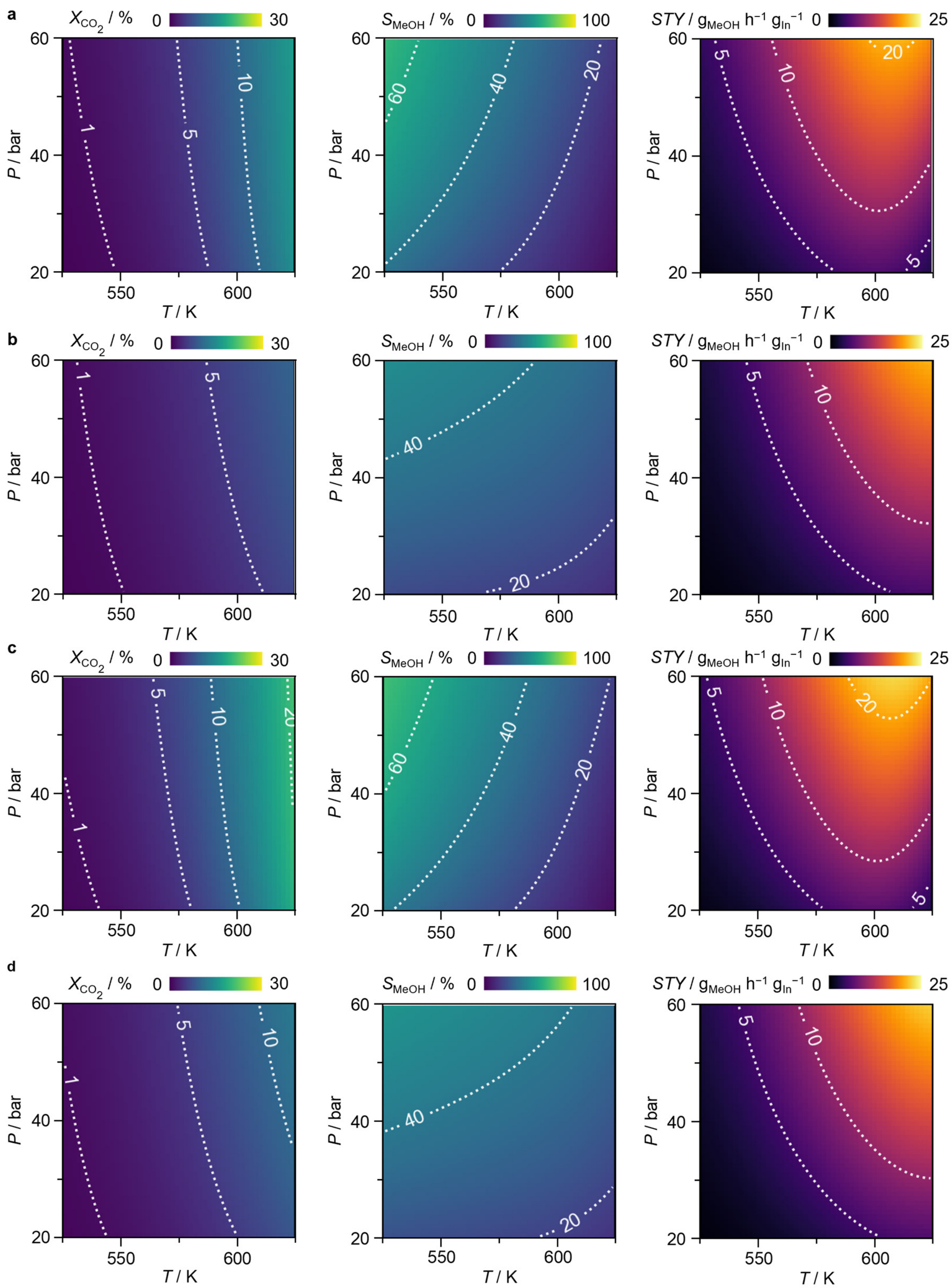

Supplementary Fig. 62. Kinetic model prediction of X_{CO2}, S_{MeOH}, and methanol *STY* during CO_2 hydrogenation over at **a**,**b**, H_2/CO_2 = 2 and **c**,**d**, H_2/CO_2 = 3 over 2InHfO$_x$ (**a**,**c**) and 2InZrO$_x$ (**b**,**d**) catalysts as a function of *P* and *T* at *GHSV* = 24,000 cm^3 h^{-1} g$_{cat}$$^{-1}$.

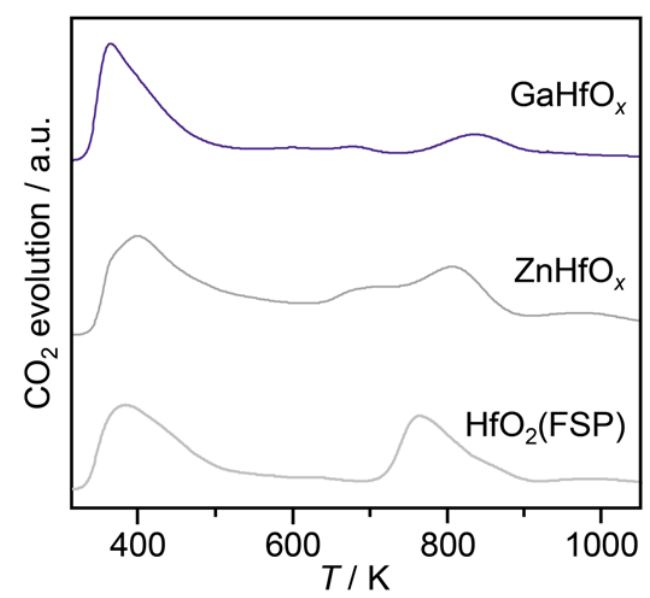

Supplementary Fig. 63. CO_2-TPD profiles of GaHfO$_x$ and ZnHfO$_x$ catalysts. HfO_2(FSP) is shown for reference.

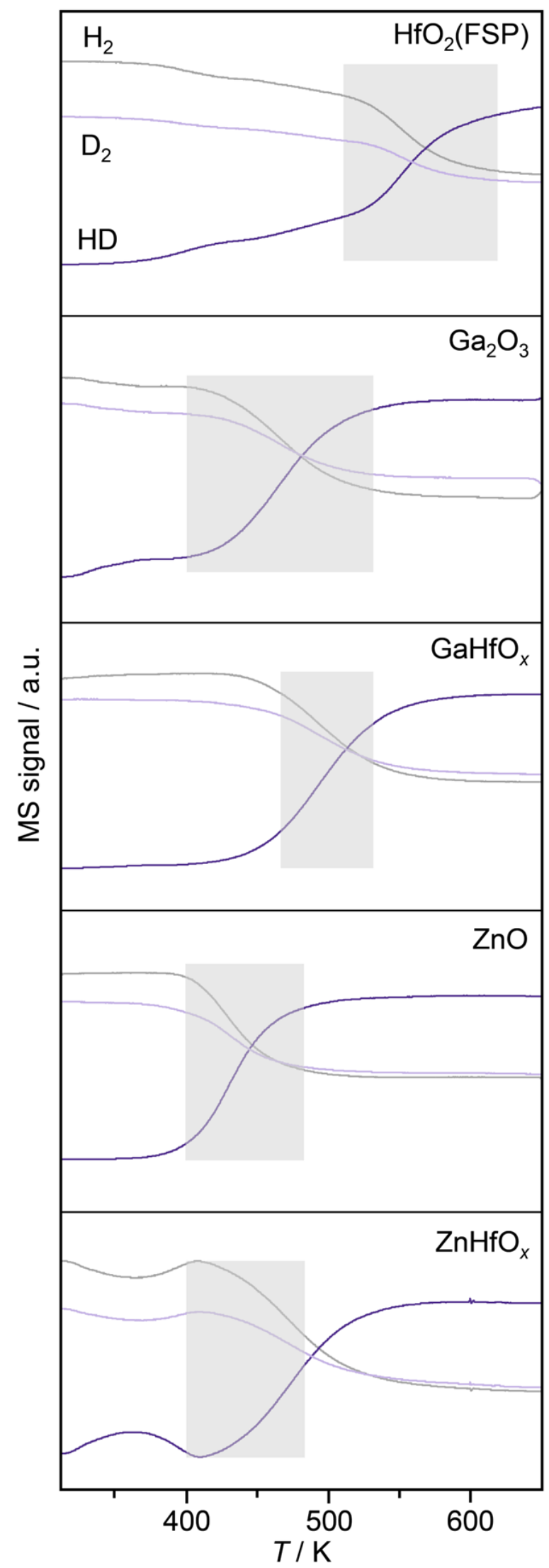

Supplementary Fig. 64. Temperature-programmed H_2-D_2 exchange profiles of $GaHfO_x$ and $ZnHfO_x$ catalysts. Shaded areas indicate temperature ranges of isotopic exchange. The pristine Ga_2O_3, ZnO, and HfO_2 supports prepared by FSP are shown for comparison.

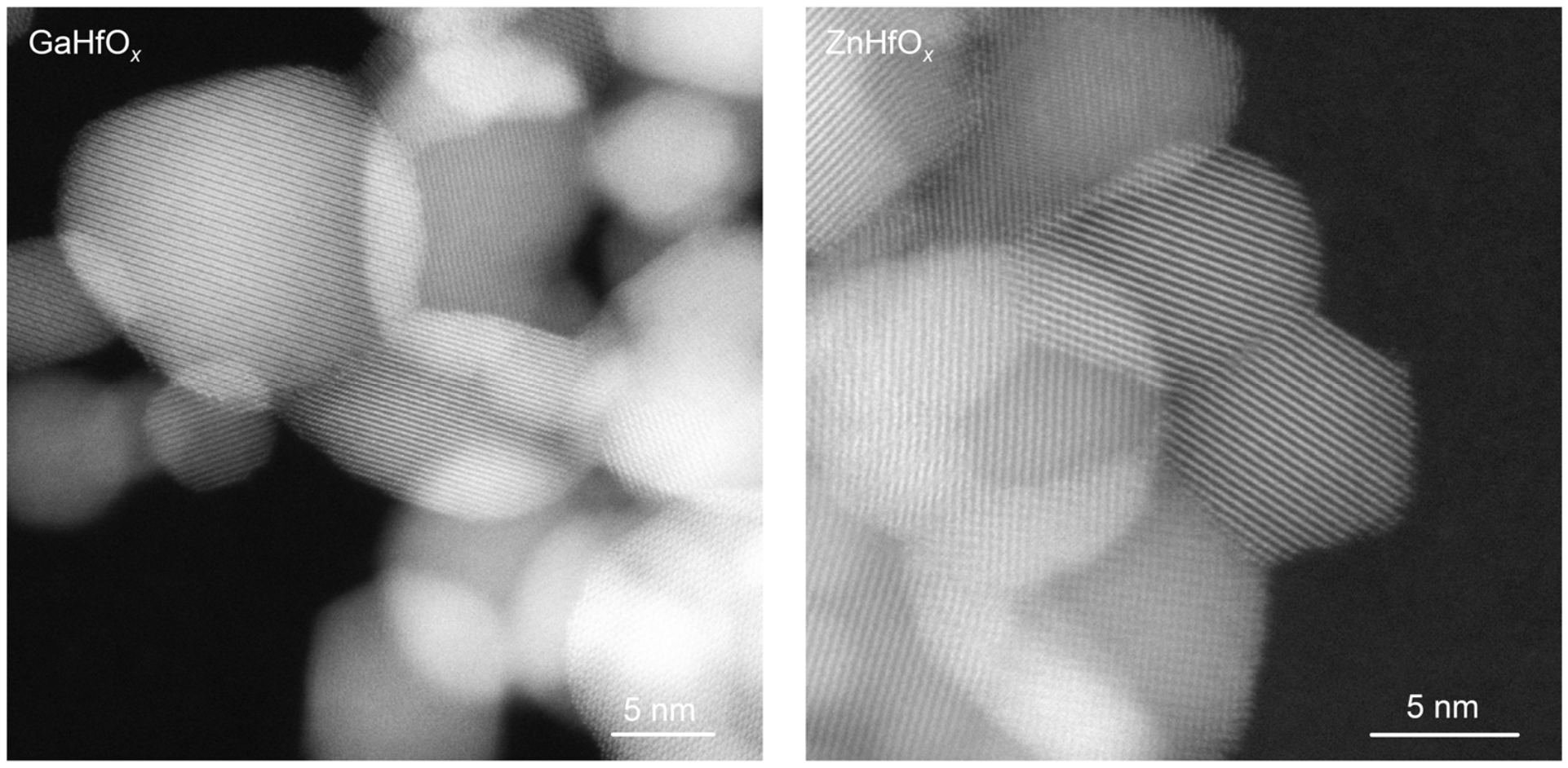

Supplementary Fig. 65. Representative HAADF-STEM image of fresh **a**, GaHfO$_x$ and **b**, ZnHfO$_x$ catalysts. The absence of clusters indicates a high metal dispersion.

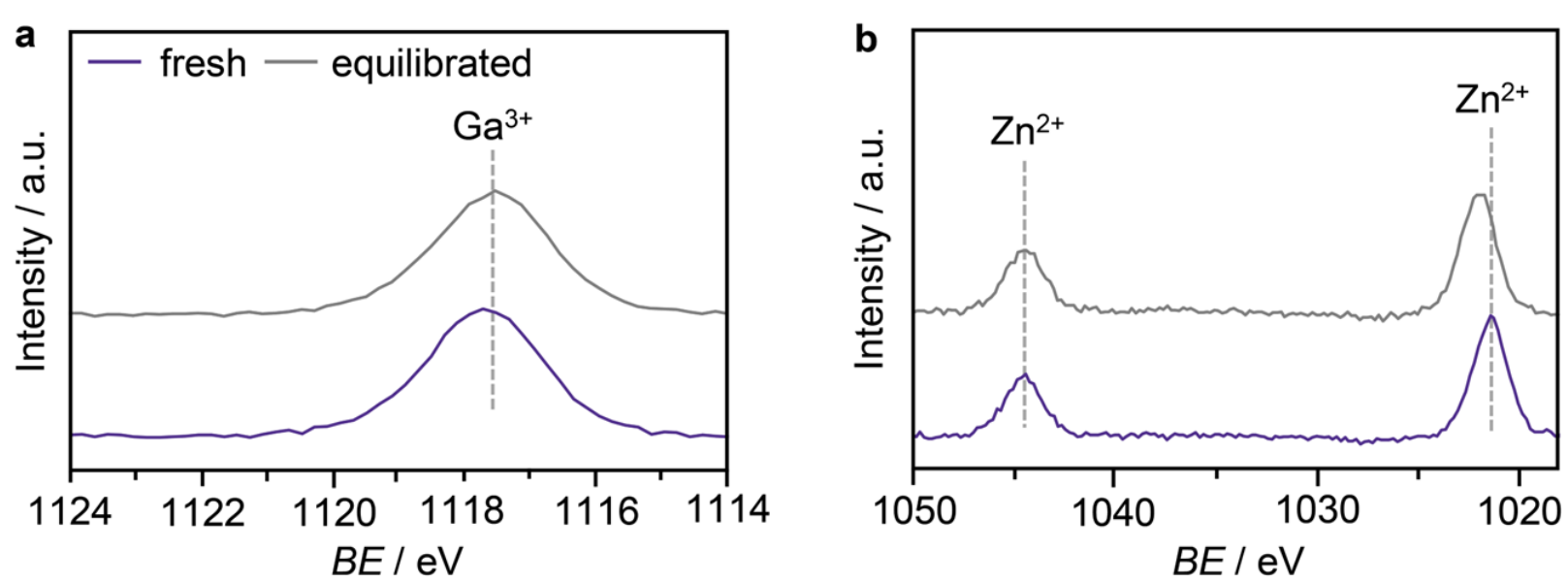

Supplementary Fig. 66. XPS Ga and Zn 2*p* core-level spectra of the GaHfO$_x$ and GaHfO$_x$ catalysts in fresh and equilibrated forms. Reaction conditions as described in **Extended Fig. 8**.

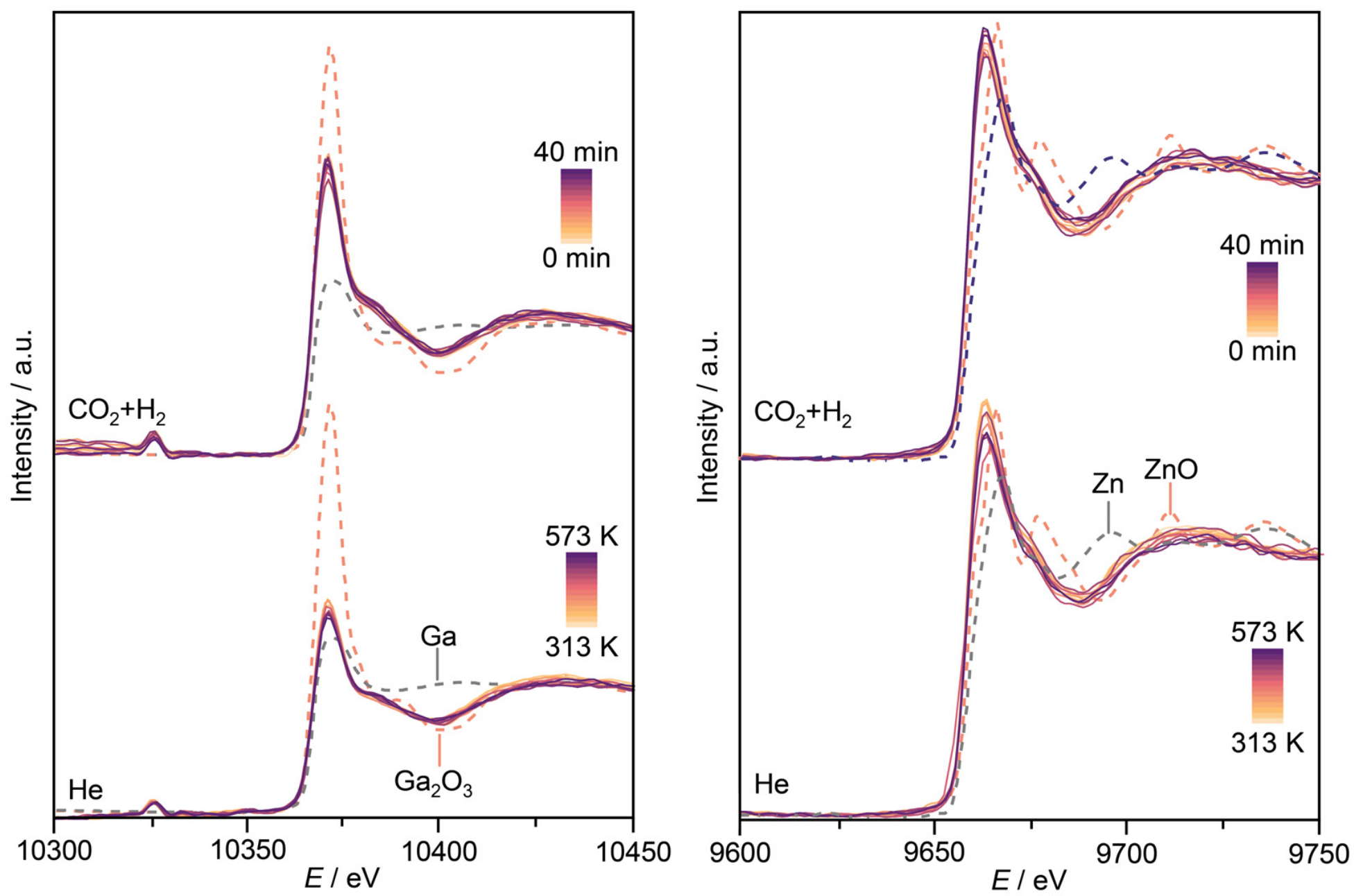

Supplementary Fig. 67. In-situ Ga and Zn *K*-edge XANES spectra of $GaHfO_x$ and $ZnHfO_x$ (m_{cat} = 0.013 g) during heating in He (rate = 5 K min^{-1}, F_T = 20 cm^3 min^{-1}, T = 573 K, P = 15 bar, dwell time = 60 min) and CO_2+H_2 (F_T = 20 cm^3 min^{-1}, T = 573 K, P = 15 bar, H_2/CO_2 = 4, dwell time = 60 min).

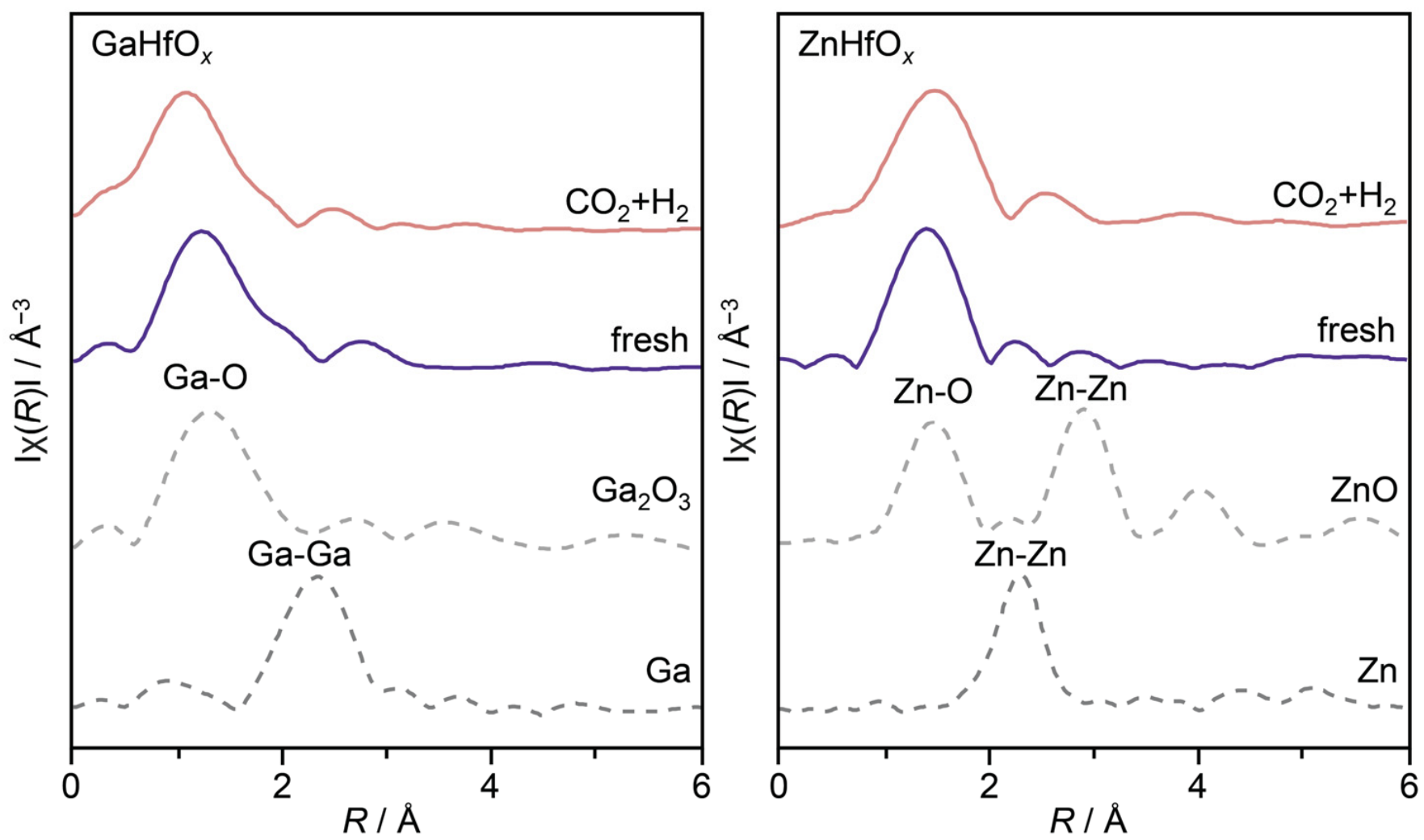

Supplementary Fig. 68. Operando *K*-edge EXAFS spectra of $GaHfO_x$ and $ZnHfO_x$ in fresh form and in CO_2+H_2 mixtures. The spectra of bulk metallic foils (Ga and Zn), Ga_2O_3, and ZnO are shown for reference. Reaction conditions as described in **Supplementary Fig. 64**.

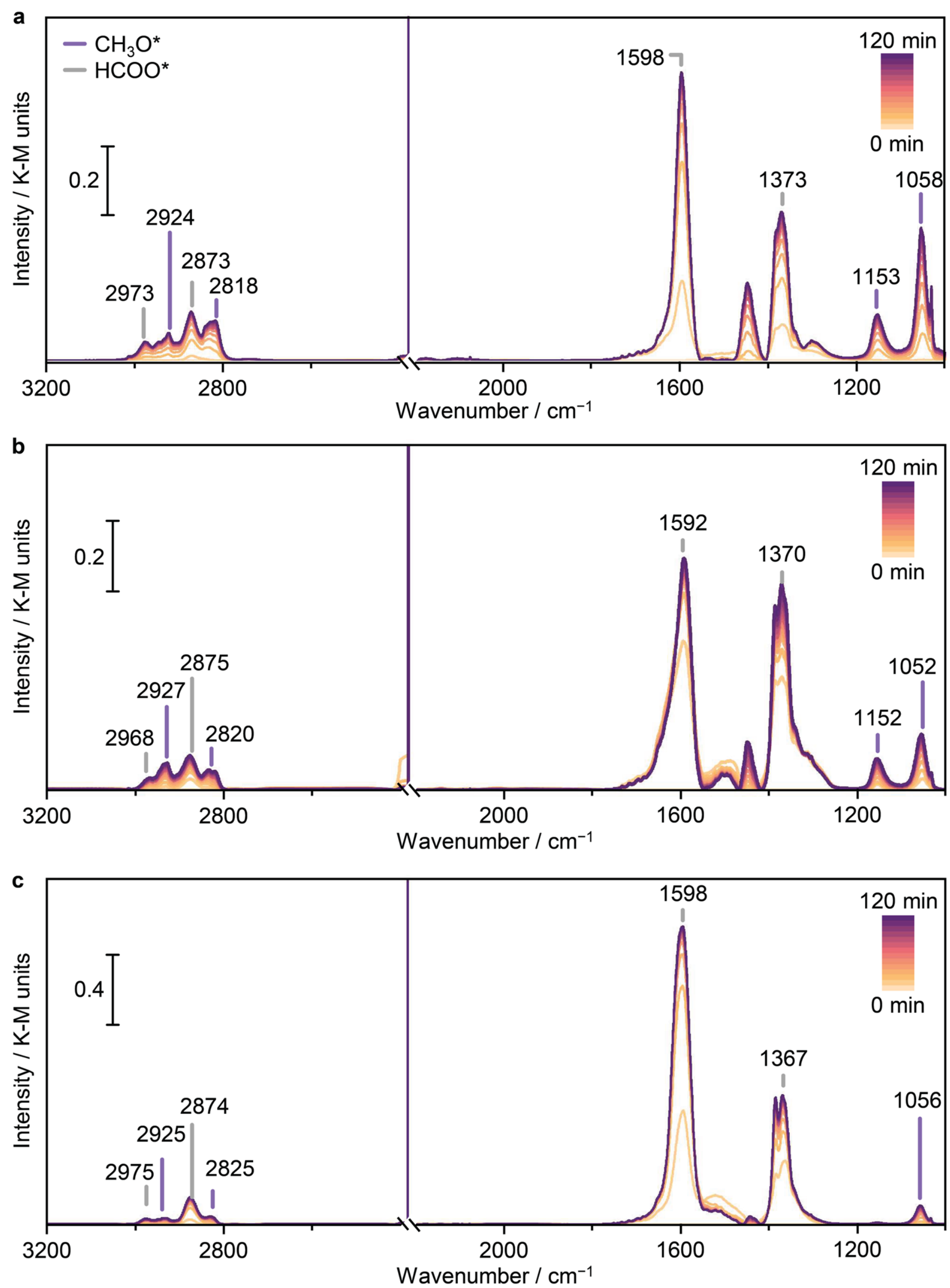

Supplementary Fig. 69. Full in-situ DRIFT spectra of **a**, $InHfO_x$ **b**, $GaHfO_x$, and **c**, $ZnHfO_x$ catalysts under CO_2 hydrogenation conditions ($F_T = 20$ cm^3 min^{-1}, $T = 573$ K, $P = 20$ bar, $H_2/CO_2 = 4$, dwell time = 120 min).

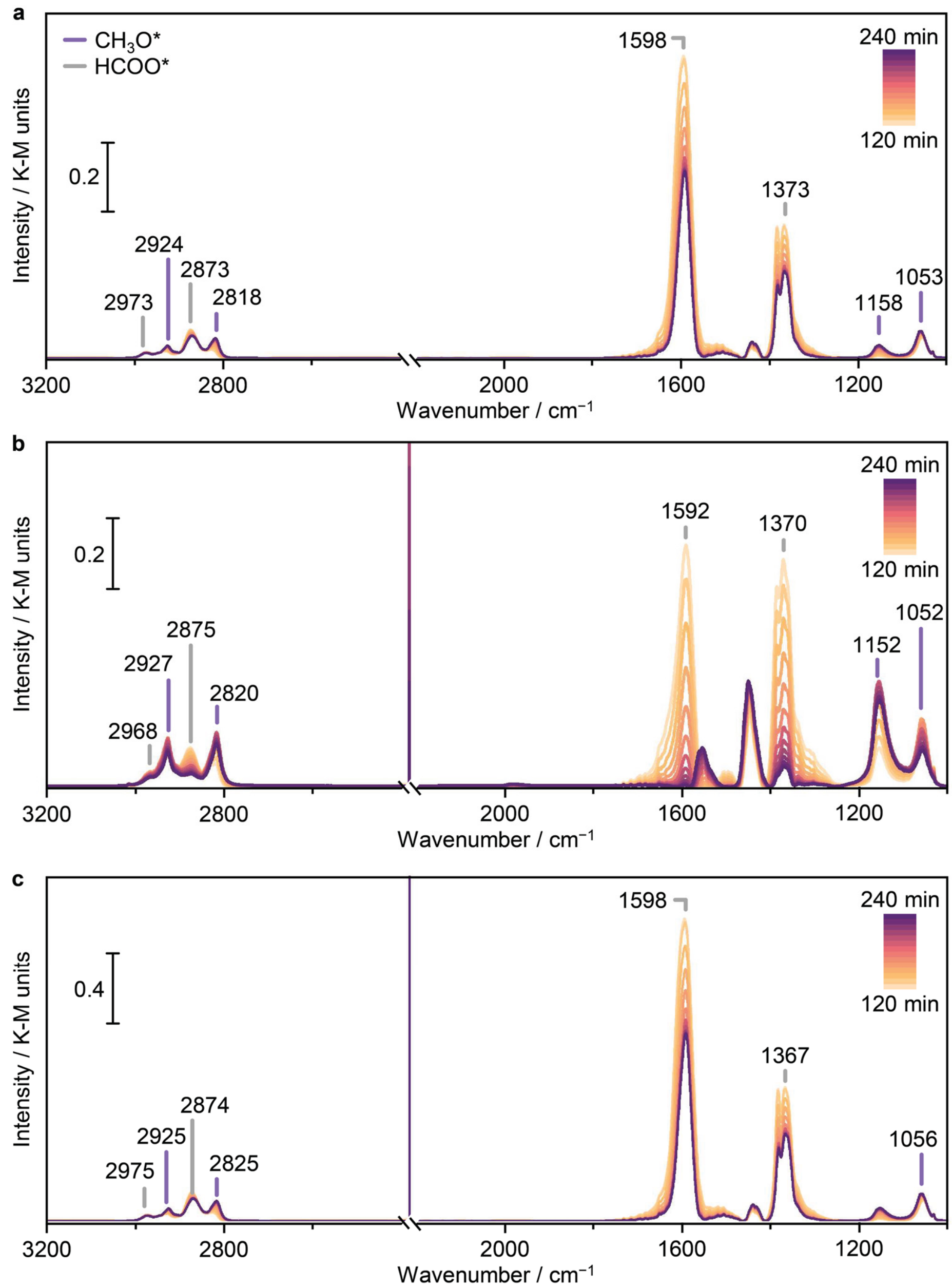

Supplementary Fig. 70. Full in-situ DRIFT spectra of **a**, $InHfO_x$ **b**, $GaHfO_x$, and **c**, $ZnHfO_x$ catalysts under hydrogenation conditions (after switching to H_2 balanced with He, $F_T = 20\ cm^3\ min^{-1}$, $T = 573$ K, $P = 20$ bar, H_2/He = 4, dwell time = 120 min).

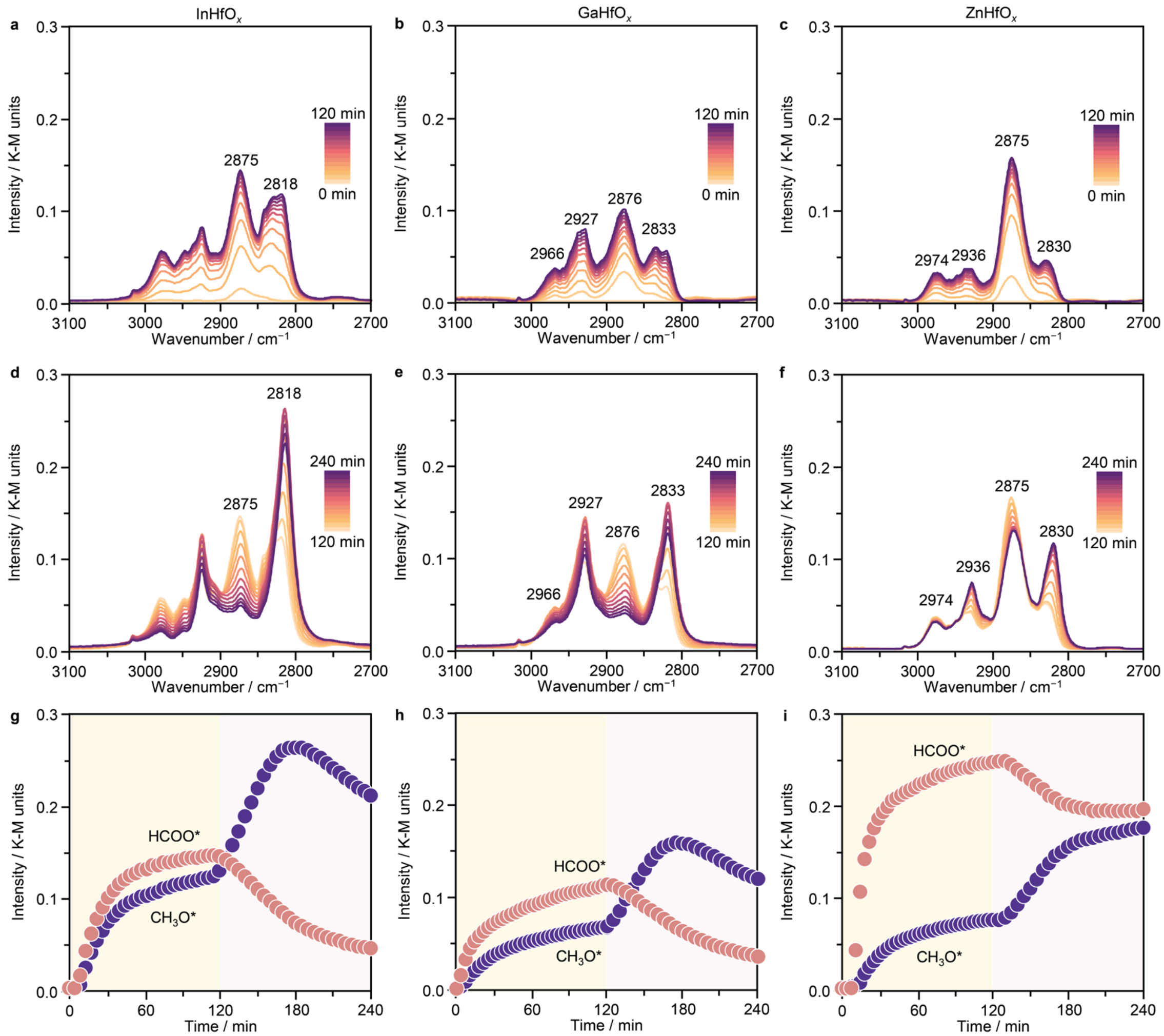

Supplementary Fig. 71. In-situ DRIFT spectra of $InHfO_x$, $GaHfO_x$, and $ZnHfO_x$ **a-c**, under CO_2 hydrogenation conditions (F_T = 20 cm^3 min^{-1}, T = 573 K, P = 20 bar, H_2/CO_2 = 4, dwell time = 120 min) and **d-f**, after switching to H_2 balanced with He (F_T = 20 cm^3 min^{-1}, T = 573 K, P = 20 bar, H_2/He = 4, dwell time = 120 min). **h-j**, Evolution of the intensities of formate (HCOO*, 2876 cm^{-1}, pink) and methoxy (CH_3O*, 2827-2837 cm^{-1}, purple) bands over during CO_2 hydrogenation (shaded yellow, F_T = 20 cm^3 min^{-1}, T = 573 K, P = 2.0 MPa, H_2/CO_2 = 4, dwell time = 120 min) and subsequent H_2/He flow.

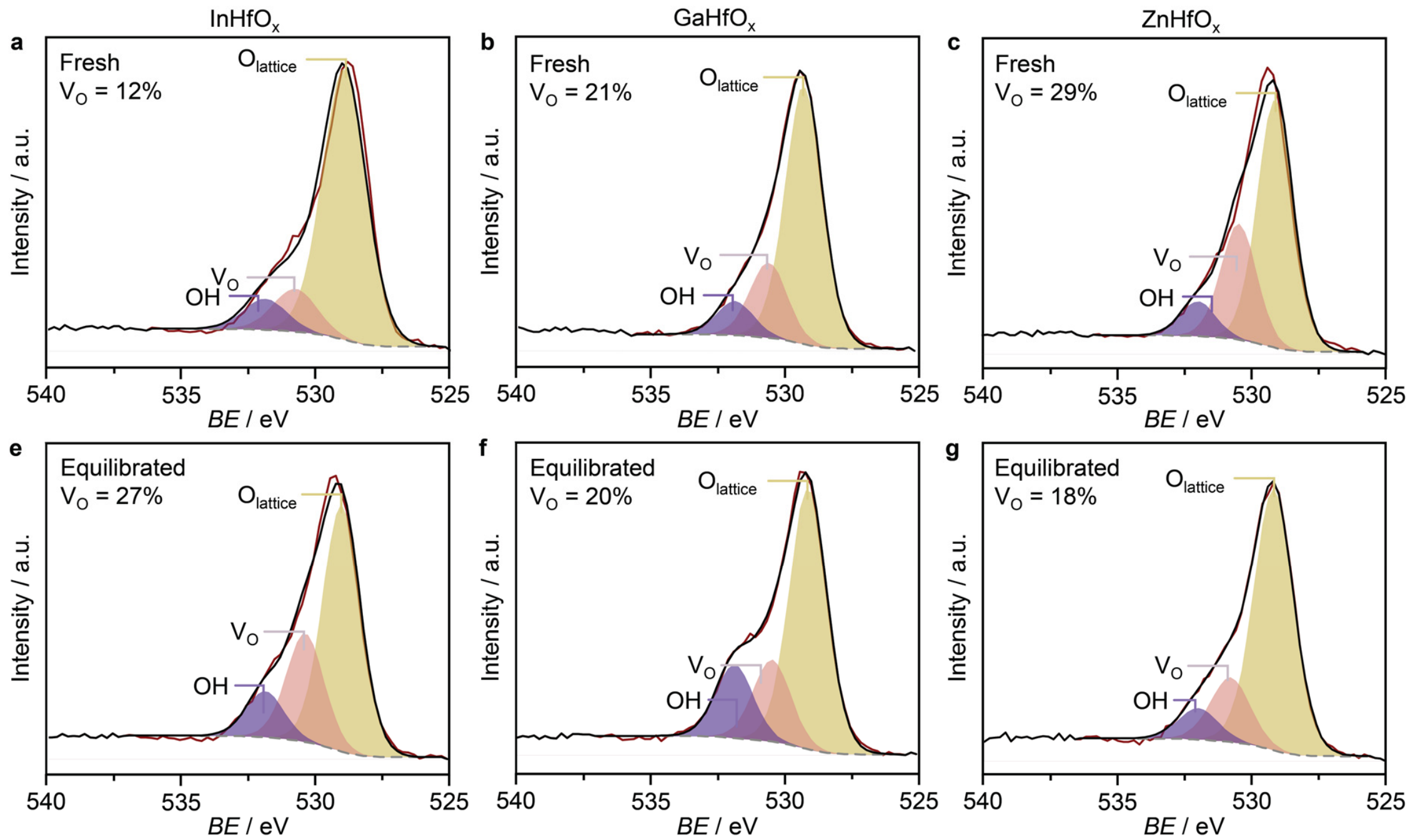

Supplementary Fig. 72. XPS O 1s core-level spectra of **a-c**, fresh and **d-f**, equilibrated InHfO$_x$, GaHfO$_x$, and ZnHfO$_x$ catalysts, highlighting differences in oxygen vacancy generation. Reaction conditions as indicated in **Extended Fig. 8**.

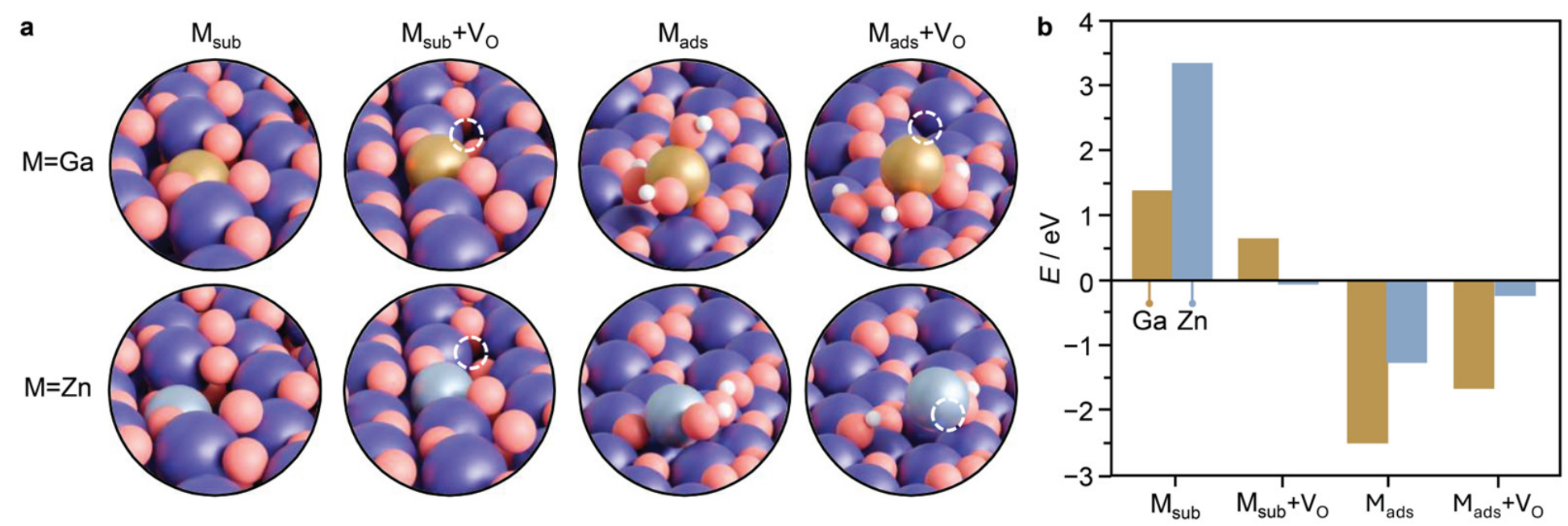

Supplementary Fig. 73. **a**, Schematic of PBE-computed structures of the lowest-energy configurations containing Ga and Zn single atom substituted in the support surface (M_{sub}) or adsorbed as $Ga(OH)_3$/$Zn(OH)_2$ single atoms (M_{ads}) with (M-V_O) or without (M) an oxygen vacancy over m-HfO_2. Colour code of DFT models: Hf - blue; Ga - yellow; Zn - grey; O - pink. Analogous to $InHfO_x$, the substitution of Ga/Zn and adsorption of $Ga(OH)_3$ and $Zn(OH)_2$ (**Supplementary Figs. 15** and **19**) are investigated. Moreover, all possible oxygen vacancy sites were explored according to **Supplementary Figs. 17** and **21**. **b**, Corresponding potential energy (E) values of the systems shown in **a**. The specific values associated with these models, and the comparison with $InHfO_x$ can be found in **Supplementary Table 16**.

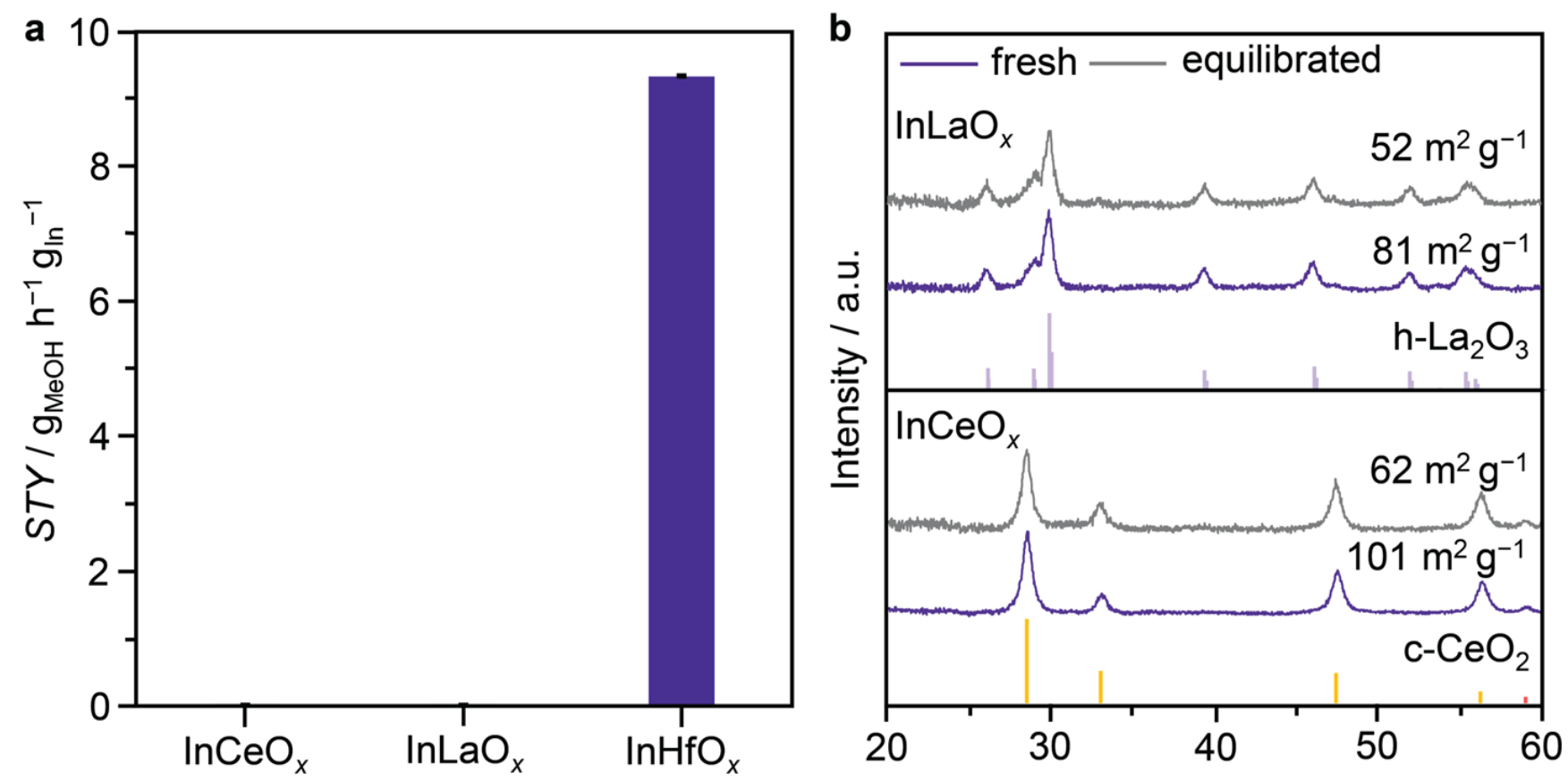

Supplementary Fig. 74. a, Methanol space-time yield (*STY*) of $InCeO_x$ and $InLaO_x$ catalysts with 2 wt% In_2O_3 content, normalized by gram of indium. $InHfO_x$ is shown for comparison. **b**, XRD profiles and BET surface areas of fresh and equilibrated $InCeO_x$, and $InLaO_x$ catalysts. Despite the wide bandgap in CeO_2 and La_2O_3 supports, no promotional effect was observed due to the high symmetry of cubic (c-) and hexagonal (h-) lattice, respectively. Moreover, significant sintering arising from their reducible character was also observed. Reaction conditions: $T = 553$ K, $P = 50$ bar, $H_2/CO_2 = 4$, $GHSV = 24{,}000$ cm^3 h^{-1} g_{cat}^{-1}, *tos* = 20 h.

Supplementary Video Captions

Supplementary Video 1. Top-view projections of ab initio Molecular Dynamics (AIMD) simulations for pristine $In(OH)_3$-m-HfO_2, for the system with one oxygen vacancy, and for configurations with one oxygen vacancy and adsorbed HCOO or CH_3O (left to right). Simulations were performed using a temperature ramp from 0 to 553 K over 5 ps in the NVT ensemble with Nosé-Hoover thermostat and a 1 fs time step. Colour code: Hf - purple; O - salmon pink, In - red; C - black, and H - white. The indium trajectory indicates that the single atom undergoes only confined diffusion within the first coordination shell of Hf atoms, and the local mobility is further attenuated in the presence of reaction intermediates.

Supplementary Video 2. Top-view projections of ab initio Molecular Dynamics (AIMD) simulations for pristine $In(OH)_3$-m-ZrO_2, for the system with one oxygen vacancy, and for configurations with one oxygen vacancy and adsorbed HCOO or CH_3O (left to right). Simulations were performed using a temperature ramp from 0 to 553 K over 5 ps in the NVT ensemble with Nosé-Hoover thermostat and a 1 fs time step. Colour code: Zr - teal; O - salmon pink, In - red; C - black, and H - white. The indium trajectory indicates that the single atom undergoes only confined diffusion within the first coordination shell of Zr atoms, and the local mobility is further attenuated in the presence of reaction intermediates.

Supplementary Video 3. Top-view projections of ab initio Molecular Dynamics (AIMD) simulations for pristine $In(OH)_3$-t-ZrO_2, for the system with one oxygen vacancy, and for configurations with one oxygen vacancy and adsorbed HCOO or CH_3O (left to right). Simulations were performed using a temperature ramp from 0 to 553 K over 5 ps in the NVT ensemble with Nosé-Hoover thermostat and a 1 fs time step. Colour code: Zr - teal; O - salmon pink, In - red; C - black, and H - white. The indium trajectory indicates that the single atom undergoes only confined diffusion within the first coordination shell of Zr atoms, and the local mobility stays attenuated in the presence of reaction intermediates.

Supplementary References

1. Shin, H. et al. Zirconia and hafnia polymorphs: Ground-state structural properties from diffusion Monte Carlo. *Phys. Rev. Mater.* **2**, 075001 (2018).
2. Mukhopadhyay, A. B., Sanz, J. F. & Musgrave, C. B. First-principles investigation of hydroxylated monoclinic HfO_2 surfaces. *Chem. Mater.* **18**, 3397-3403 (2006).
3. Christensen, A. & Carter, E. A. First-principles study of the surfaces of zirconia. *Phys. Rev. B* **58**, 8050-8064 (1998).
4. Ricca, C., Ringuedé, A., Cassir, M., Adamo, C. & Labat, F. A. comprehensive DFT investigation of bulk and low-index surfaces of ZrO_2 polymorphs. *J. Comput. Chem.* **36**, 9-21 (2015).
5. Baidun, M. S., Kolganov, A. A., Alexandrova, A. N. & Pidko, E. A**.** Hydride accessibility and reactivity in the configurational and stoichiometric space of β-Ga_2O_3 for CO_2 hydrogenation. *J. Phys. Chem. Lett.* **16**, 7732-7737 (2025).
6. Bollinger, M. V., Jacobsen, K. W. & Nørskov, J. K. Atomic and electronic structure of MoS_2 nanoparticles. *Phys. Rev. B* **67**, 085410 (2003).
7. Allison, T. C. *NIST–JANAF Thermochemical Tables (SRD 13)* (NIST, 2013). Available at: janaf.nist.gov (accessed 10 Nov 2025)
8. Frisch, M. J. et al. *Gaussian 09* (Revision A.02).
9. Araújo, T. P. et al. Flame-made ternary Pd-In_2O_3-ZrO_2 catalyst with enhanced oxygen vacancy generation for CO_2 hydrogenation to methanol. *Nat. Commun.* **13**, 5610 (2022).
10. Zou, T. et al. Descriptors of $InZrO_x$ vs $ZnZrO_x$ catalysts for CO_2 hydrogenation to methanol. *Adv. Energy Mater.* **15**, 2404967 (2025).
11. Araújo, T. P. et al. Design of flame-made $ZnZrO_x$ catalysts for sustainable methanol synthesis from CO_2. *Adv. Energy Mater.* **13**, 2204122 (2023).
12. Zou, T. et al. Design of technical ZnO/ZrO_2 catalysts for CO_2 hydrogenation to green methanol. *J. Catal.* **430**, 115344 (2024).

13. Jain, A. et al. Commentary: the Materials Project: A materials genome approach to accelerating materials innovation. *APL Mater.* **1**, 011002 (2013).

14. Augusto, T. de M. et al. Influence of electronic and structural properties on $Au/In_2O_3/ZrO_2$ catalysts for CO_2 hydrogenation to methanol. *Chem. Eng. J.* **505**, 159750 (2025).

15. Xu, L. et al. Interfacial-Confined Oxygen Vacancy Clusters in ZrO_2-Supported In_2O_{3-x} Catalysts Boost Methanol Production from CO_2. *Angew. Chem. Int. Ed.* **64**, e202508091 (2025).

16. Wei, Y., Liu, F., Ma, J., Yang, C., Zhang, H. & Chen, L. Catalytic roles of In_2O_3 in ZrO_2-based binary oxides for CO_2 hydrogenation to methanol. *Mol. Catal.* **525**, 112354 (2022).

17. Frei, M. S. et al. Role of zirconia in indium oxide-catalyzed CO_2 hydrogenation to methanol. *ACS Catal.* **10**, 1133-1145 (2019).

18. Chen, T.-Y. et al. Unraveling highly tunable selectivity in CO_2 hydrogenation over bimetallic In–Zr oxide catalysts. *ACS Catal.* **9**, 8785-8797 (2019).

19. Martin, O. et al. Indium oxide as a superior catalyst for methanol synthesis by CO_2 hydrogenation. *Angew. Chem. Int. Ed.* **55**, 6261-6265 (2016).

20. Gao, F. et al. Redox-Mediated Interfacial Restructuring of Supported In_2O_3 to Drive CO_2 Hydrogenation to Methanol. *ACS Catal.* **15**, 2785-2795 (2025).

21. Luo, A., Chang, H., Gao, F., Liu, Y., He, H. & Cao, Y. Towards maximizing the In_2O_3/m-ZrO_2 interfaces for CO_2-to-methanol hydrogenation. *Chem. Commun.* **59**, 12747-12750 (2023).

22. Sharma, P., Hoang H. P., Shao, J., Creaser, D. & Olsson, L. Role of ZrO_2 and CeO_2 support on the In_2O_3 catalyst activity for CO_2 hydrogenation. *Fuel* **331**, 125878 (2023).

23. Eliasson, H. et al. Tracking dynamics of supported indium oxide catalysts in CO_2 hydrogenation to methanol by in situ TEM. *Adv. Mater.* **37**, 2419859 (2025).

24. Hann, R. E., Suitch, P. R. & Pentecost, J. L. Monoclinic crystal structures of ZrO_2 and HfO_2 refined from X-ray powder diffraction data. *J. Am. Ceram. Soc.* **68**, 285-286 (1985).

25. Ricca, C., Ringuedé, A., Cassir, M., Adamo, C. & Labat, F. A comprehensive DFT investigation of bulk and low-index surfaces of ZrO_2 polymorphs. *J. Comput. Chem.* **36**, 9-21 (2015).
