# Supplementary material for: Single atoms of indium on hafnia enable superior CO2-based methanol synthesis
Source: Nat Nanotechnol. 2026 Mar 2;21(4):588–97. doi: 10.1038/s41565-026-02135-y (PMC13652736; doi:10.1038/s41565-026-02135-y)
Supplement: Supplementary file 2 [file 41565_2026_2135_MOESM2_ESM.pdf]

nature portfolio

Peer Review File

Single atoms of indium on hafnia enable superior CO2-based methanol synthesis

Corresponding Author: Professor Javier Pérez-Ramírez

Version 0:

Reviewer comments:

Reviewer #1

(Comments for the Author)
The current study reports a promising catalyst by forming single In-sites in m-HfO2 for selective CO2 to methanol conversion. Multiple characterizations and DFT calculations were performed to gain mechanistic understanding and lead to the design of catalysts for improvements. The results are intriguing, but needs clarifications and additional evidences.

1. In-based catalysts have been extensively studied for CO2 hydrogenation to methanol. To increase the impact of current effort, the comparison in activity, selectivity and durability of catalysts in the work with the reported is necessary. The advantages over the previous needs to be emphasized, which is not clear at the moment.

2. Structures and bindings under the reaction conditions are more relevant to the catalysis observed than that of the as-prepared or spent. Therefore, the in situ characterizations help. While the impact of reaction conditions does not seem to be considered in the DFT calculations, which can enable the interaction with the in situ results. What is the possible variation in surface species, surface morphology and possible composition driven interaction with reaction intermediates under the reaction condition. According to DRIFTS, the surface sites likely are not stay free, but can be continuously occupied by some reaction intermediates. As a result, the surface and active sites may not be the ideal as currently considered in DFT. The difference between ideal surface vs. stable surface under reaction should be addressed. Would surface hydroxylation or formation of carbonate occur? What are the effects on catalysis?

3. According to the DFT-calculated thermodynamics (figure 6), it is difficult to tell the difference in activity for the three systems. It will be helpful to calculate the barriers on the stable surface under reaction and provide kinetic analysis based on that.

4. The stability of sing-atom sites is challenging. Is there a stability test to address that? How is the stability ensured with the generation of vacancy? Does single atom of In stay embedded in the surface on interaction with active intermediates like HCOO or CH3O, get pulled on to the surface or undergo transition to other structures? Why?

Reviewer #2

(Comments for the Author)
This manuscript comprehensive study on the superior catalytic performance of $InHfO_x$ catalysts for CO_2 hydrogenation to methanol, outperforming the established benchmark $InZrO_x$ system. Compared to InZrOx, nanostructured InHfOx prepared by flame spray pyrolysis (FSP) shows an enhanced indium-specific methanol productivity by up to 70%. The promotional effect is most pronounced for atomically dispersed indium species. The work is exceptionally timely, addressing a long-standing challenge in catalyst design by moving beyond Zr-based supports. However, some issues still need further revision and improvement. The specific comments are as follows:

1.The manuscript indicates that the FSP method generates a unique surface hydroxyl environment, which enhances the CO2 adsorption and hydrogenation capabilities. Is this effect universal across the FSP method? Does it apply to other metal-oxide systems, or is it specific to the InHfOx system?
2.While the results for ZnHfOx and GaHfOx are mentioned in the catalyst comparison, there is a lack of in-depth mechanistic explanation. It is suggested to provide a more detailed comparison to clarify whether the universal promotional mechanism of HfO2 truly exists.
3.The manuscript notes that the scarcity of HfO2 may restrict its large-scale application. However, is it possible to explore the

potential of rare-earth oxides such as CeO_2, La_2O_3, or other wide-bandgap oxides as alternatives?
4.Although the manuscript highlights the importance of CO_2 hydrogenation to methanol and the current research status of InZrOx catalysts, it falls short in delving into the limitations of existing catalyst systems, which somewhat weakens the focus and motivation of this study.
5.The transitions between some sections are not sufficiently smooth or logically coherent. For instance, after presenting experimental results, the manuscript directly proceeds to theoretical calculations without providing a thorough summary of the experimental findings or a clear connection to the calculation part.
6.All the DFT calculations are at 0 K, overlooking temperature effects and configurational entropy in the thermodynamics. Gibbs free-energy correction should be incorporated.
7.The DFT+U method is widely recognized to better reproduce experimental observables (e.g., lattice constants and band gaps) for metal oxides. Please compare experimental and computed band gaps and justify why DFT+U was not employed.
8.The Authors are suggested to perform ab initio thermodynamics to assess free-energy changes as a function of temperature and pressure, using the chemical potential of $In(OH)_3$ (molecule in gas phase) and In (atom in the bulk).
9.In-situ DRIFT assigns the vibrational peak of CO_2 adsorption. DFT can also predict the $^*CO_2$ vibrational frequencies to guide the local environment of CO_2 adsorption configuration.
10.“In@” denotes a substituted indium atom. Please define this notation at its first occurrence in Fig. 4c.
11.In Fig. 6a, $^*CH_2O$ formation is identified as the rate-determining step, since the $In(OH)_3$-m-HfO_2 has a higher ΔE value than $In(OH)_3$-m-ZrO_2. Some statements should be clarified.
12. The outlook could be slightly expanded to suggest specific other wide-bandgap, high-κ dielectrics (beyond HfO_2 and ZrO_2) that might be explored based on the proposed design principle, perhaps guided by materials databases.

Version 1:

Reviewer comments:

Reviewer #1

(Comments for the Author)
The reviewer's comments are addressed and the revised manuscript is recommended for acceptance.

Reviewer #2

(Comments for the Author)
This is a top-tier, potentially field-defining study. The authors have successfully addressed every point raised by Reviewers with substantial new data, rigorous analyses, and clear revisions. The manuscript is now significantly stronger in terms of mechanistic insight, generalizability, and methodological rigor. The work is suitable for publication in Nature Nanotechnology. I recommend acceptance pending incorporation of the minor revisions and clarifications suggested below.

Minor Points & Typos

Page 8, line 176: “Hudium” → “Indium”.
Page 22, line 525: “hearing” → “heating”.
Page 30, line 716: “References” – Please check that all references are formatted consistently (some have full journal names, others abbreviations).
Please ensure all acronyms are defined at first use (e.g., TPSR, HD exchange).

Open Access This Peer Review File is licensed under a Creative Commons Attribution 4.0 International License, which permits use, sharing, adaptation, distribution and reproduction in any medium or format, as long as you give appropriate credit to the original author(s) and the source, provide a link to the Creative Commons license, and indicate if changes were made.
In cases where reviewers are anonymous, credit should be given to 'Anonymous Referee' and the source.
The images or other third party material in this Peer Review File are included in the article's Creative Commons license, unless indicated otherwise in a credit line to the material. If material is not included in the article's Creative Commons license and your intended use is not permitted by statutory regulation or exceeds the permitted use, you will need to obtain permission directly from the copyright holder.
To view a copy of this license, visit https://creativecommons.org/licenses/by/4.0/

Response to Referees for NNANO-25083313
Comments in blue | Replies in black | **Actions in bold**
Page, line, figure, or reference numbers refer to the revised files with tracked changes.

Reviewer #1

The current study reports a promising catalyst by forming single In-sites in m-HfO_2 for selective CO_2 to methanol conversion. Multiple characterizations and DFT calculations were performed to gain mechanistic understanding and lead to the design of catalysts for improvements. The results are intriguing, but needs clarifications and additional evidences.

We warmly thank Reviewer #1 for their interest and constructive comments on our study. Following these, we have substantially revised the manuscript, adding the requested clarifications and new experimental and computational results, which strengthen the mechanistic conclusions and address all points raised.

1. In-based catalysts have been extensively studied for CO_2 hydrogenation to methanol. To increase the impact of current effort, the comparison in activity, selectivity and durability of catalysts in the work with the reported is necessary. The advantages over the previous needs to be emphasized, which is not clear at the moment.

To enable a rigorous comparison with existing In-based catalysts, **we compiled the most relevant reports on supported In_2O_3 systems (In_2O_3 content <20 wt%, comparable testing conditions) to pinpoint the effect of the carrier (Supplementary Table 4, Supplementary Fig. 1)**. The analysis shows that our $InHfO_x$ catalyst prepared via flame-spray pyrolysis achieves the highest methanol space-time yield per indium with the lowest metal content reported to date, establishing monoclinic HfO_2 as the first support to outperform state-of-the-art ZrO_2-based systems.

2. Structures and bindings under the reaction conditions are more relevant to the catalysis observed than that of the as-prepared or spent. Therefore, the in situ characterizations help. While the impact of reaction conditions does not seem to be considered in the DFT calculations, which can enable the interaction with the in situ results. What is the possible variation in surface species, surface morphology and possible composition driven interaction with reaction intermediates under the reaction condition. According to DRIFTS, the surface sites likely are not stay free, but can be continuously occupied by some reaction intermediates. As a result, the surface and active sites may not be the ideal as currently considered in DFT. The difference between ideal surface vs. stable surface under reaction should be addressed. Would surface hydroxylation or formation of carbonate occur? What are the effects on catalysis?

Surface speciation under reaction conditions has been examined using in-situ DRIFTS, which shows continuous hydroxylation (3200-3700 cm^{-1}) and the formation of carbonate species (1100-1600 cm^{-1}) during CO_2 hydrogenation to methanol. **These results are now included in the supplementary information (Supplementary Figs. 54,56) and discussed in the revised manuscript (page 17, lines 12-16).**

To assess the impact of these surface species on the active site structure, we extended the DFT simulations to evaluate the role of surface hydroxyls and carbonates on the reaction profiles of the most relevant models, viz. $In(OH)_3$-m-HfO_2, $In(OH)_3$-m-ZrO_2, and $In(OH)_3$-t-ZrO_2. Briefly, the dissociated adsorption of H_2O and CO_2 is exothermic across all systems (from –0.72 to –1.30 eV per H_2O molecule and –0.25 to –0.95 eV per CO_2 molecule, at high and low coverages), consistent with experimental observations. The presence of these spectator species does not noticeably alter the reaction profiles for the monoclinic HfO_2 or ZrO_2 models. However, a detrimental effect on reactant activation is observed in the case of the t-ZrO_2 phase. **We have now added these results and discussion to the revised manuscript (page 17, lines 12-15), along with new Supplementary Figs. 56-58.**

3. According to the DFT-calculated thermodynamics (figure 6), it is difficult to tell the difference in activity for the three systems. It will be helpful to calculate the barriers on the stable surface under reaction and provide kinetic analysis based on that.

We have now included the full reaction profiles with reaction barriers, temperature effect, and Gibbs free-energy corrections for the $In(OH)_3$-m-HfO_2, $In(OH)_3$-m-ZrO_2, and $In(OH)_3$-t-ZrO_2 models (Fig. 6a). We observe that while the activation energies are similar across the three systems, the formation of HCOO ($\Delta G_{CO2+H2\rightarrow HCOO}$) over CO_2H ($\Delta G_{CO2+H2\rightarrow CO2H}$), which is key to achieve high methanol selectivity, is more thermodynamically favored for $In(OH)_3$-m-HfO_2 compared to $In(OH)_3$-m-ZrO_2. This is in line with the higher selectivity exhibited by $InHfO_x$. In contrast, the $In(OH)_3$-t-ZrO_2 model exhibits overstabilization of HCOO, CH_2O_2, and CH_3O intermediates, indicating that the t-ZrO_2 fraction in 2$InZrO_x$ is likely detrimental to its catalytic performance. **These results have been included and discussed in the revised manuscript (page 16, lines 21-26).**

4. The stability of sing-atom sites is challenging. Is there a stability test to address that? How is the stability ensured with the generation of vacancy? Does single atom of In stay embedded in the surface on interaction with active intermediates like HCOO or CH_3O, get pulled on to the surface or undergo transition to other structures? Why?

A comprehensive stability evaluation was provided in the original manuscript, combining a 150 h catalytic test, in-situ XAS and EPR measurements under reaction conditions, and ex-situ AC-STEM-EDX analyses (**Fig. 5a,b** and **Supplementary Fig. 3**). These results demonstrate the outstanding robustness of the isolated indium sites. **We have further emphasized this in the revised manuscript, explicitly noting that no spectral changes are observed under reaction conditions (page 14, lines 15-16)**.

To further probe site stability under operating conditions, **we performed DFT molecular dynamics (MD) simulations (5 ps, temperature ramp 0-553 K) for $In(OH)_3$-m-HfO_2, $In(OH)_3$-m-ZrO_2, and $In(OH)_3$-t-ZrO_2 models both in their pristine form and with oxygen vacancies, and in the presence or absence of adsorbed HCOO or CH_3O intermediates (Supplementary Videos 1-3).** In all cases, the indium atom remains confined within a narrow region defined by the first-neighbor hafnium or zirconium atoms, and the local environment is preserved. Reaction intermediates slightly reduce the mobility but do not induce detachment,

surface migration, or structural rearrangement. These results conclusively demonstrate the high stability of the indium single atoms under reaction conditions. **The outcome of these important analyses has been included in the revised manuscript (page 17, lines 1-6) and Supplementary Figs. 51-53.**

Reviewer #2

This manuscript comprehensive study on the superior catalytic performance of $InHfO_x$ catalysts for CO_2 hydrogenation to methanol, outperforming the established benchmark $InZrO_x$ system. Compared to $InZrO_x$, nanostructured $InHfO_x$ prepared by flame spray pyrolysis (FSP) shows an enhanced indium-specific methanol productivity by up to 70%. The promotional effect is most pronounced for atomically dispersed indium species. The work is exceptionally timely, addressing a long-standing challenge in catalyst design by moving beyond Zr-based supports. However, some issues still need further revision and improvement. The specific comments are as follows:
We sincerely appreciate Reviewer #2 for highlighting the timeliness and scientific relevance of our study. We have carefully considered all points raised and strengthened the manuscript accordingly through targeted new experiments and theoretical analyses, as detailed below.

1. The manuscript indicates that the FSP method generates a unique surface hydroxyl environment, which enhances the CO_2 adsorption and hydrogenation capabilities. Is this effect universal across the FSP method? Does it apply to other metal-oxide systems, or is it specific to the $InHfO_x$ system?
Surface hydroxylation is frequently observed in flame-made oxides due to rapid quenching in air (*Appl. Surf. Sci.* **550**, 150536 (2021); *J. Colloid Interface Sci.* **467**, 235 (2016); *Sens. Actuators B Chem.* **371**, 132495 (2022); *Langmuir* **26**, 5815 (2010); *J. Colloid Interface Sci.* **507**, 95 (2017)), but its extent depends on both the synthesis conditions and intrinsic properties of the oxide. For ZrO_2, both precipitation and FSP yield hydroxylated surfaces. In contrast, to prepare monoclinic HfO2 by precipitation, high-temperature thermal treatment is required to crystallize the desired phase, which readily removes surface hydroxyls. Our observations demonstrate that the fast cooling in FSP results in a hydroxylated surface. As the synthetic accessibility of hydroxylated hafnium oxide is a pertinent consideration, we have explored other preparation methods.
We have now devised a hydrothermal synthesis route that produces hydroxylated m-HfO_2, which when used as a support for indium single atoms. The resulting catalyst achieves identical methanol productivity to FSP-prepared analog, proving that effective hafnia supports can be accessed by other methods. These key results have been incorporated in the revised manuscript (page 12, lines 25-26, page 13, lines 1-4) and Supplementary Fig. 33.

2. While the results for $ZnHfO_x$ and $GaHfO_x$ are mentioned in the catalyst comparison, there is a lack of in-depth mechanistic explanation. It is suggested to provide a more detailed comparison to clarify whether the universal promotional mechanism of HfO_2 truly exists.
A more detailed characterization of the $GaHfO_x$ and $ZnHfO_x$ catalysts has now been incorporated. Accordingly, CO_2 chemisorption, CO_2 temperature-programmed desorption, and H_2–D_2 exchange measurements show that both systems exhibit enhanced CO_2 uptake and reduced H_2 activation temperatures relative to the pristine oxides, indicating that HfO_2 generally facilitates CO_2 activation and hydrogen spillover. In addition, HAADF-STEM, XPS, and in-situ XAS analyses confirm that Ga and Zn remain atomically dispersed and stable on m-HfO_2 under

reaction conditions. These results agree with the DFT results, which evidence a favorable adsorption of hydroxylated Ga and Zn single atoms. Despite this shared promotional effect of hafnium oxide, in-situ DRIFTS measurements reveal metal-dependent (In, Ga, Zn) reaction kinetics: formate-to-methoxy conversion is fastest on $InHfO_x$, whereas $GaHfO_x$ shows weaker CO_2 activation and $ZnHfO_x$ slower hydrogen-transfer steps. Equilibrated $InHfO_x$ also displays the highest oxygen vacancy density, consistent with DFT predicted vacancy formation energies.

These results demonstrate that HfO_2 exerts a broadly beneficial effect across reducible oxides, while the magnitude of the enhancement is metal specific. **The new experimental and computational analyses are provided in the revised manuscript (page 19, lines 19-25) and supplementary information (pages 15-17 and Supplementary Figs. 63-73).**

3. The manuscript notes that the scarcity of HfO_2 may restrict its large-scale application. However, is it possible to explore the potential of rare-earth oxides such as CeO_2, La_2O_3, or other wide-bandgap oxides as alternatives?

The design requirements for effective supports for reducible oxides in CO_2 hydrogenation, viz., wide band gap, high structural anisotropy, and the ability to stabilize indium single atoms, were outlined in the original submission. **To further examine whether other rare-earth or wide-bandgap oxides could promote CO_2 hydrogenation, we prepared 2 wt% In_2O_3 supported on CeO_2 and La_2O_3 by FSP ($InCeO_x$ and $InLaO_x$).** CeO_2, with its relatively narrow band gap (~3.0-3.6 eV) and cubic lattice, and La_2O_3, with its wide bandgap hexagonal structure, do not satisfy the required geometric and electronic descriptors. Consistently, both $InCeO_x$ and $InLaO_x$ were inactive for CO_2-to-methanol conversion, and post-reaction analyses revealed significant sintering under CO_2 hydrogenation conditions. **These results are included in the revised manuscript (page 20, lines 17-20) and supplementary information (page 87).**

4. Although the manuscript highlights the importance of CO_2 hydrogenation to methanol and the current research status of $InZrO_x$ catalysts, it falls short in delving into the limitations of existing catalyst systems, which somewhat weakens the focus and motivation of this study.

Thank you for this comment. The limitations of existing catalytic systems have now been made more explicit in the revised discussion (page 18, lines 8,9). Current CO_2-to-methanol technologies rely either on $Cu/Zn/Al_2O_3$ catalysts, which are highly active but suffer from instability and poor selectivity, or on reducible oxides such as In_2O_3-based systems, which are selective and stable but intrinsically less active and, to date, restricted to ZrO_2 supports. Moreover, most supported In_2O_3 catalysts have used relatively high In_2O_3 content (>5%) to generate active ensembles (typically as extended InO_x nanoplatelets), reflecting suboptimal design principles for supports capable of stabilizing low-valence indium species while enhancing activity.

Our study tackles these decade-long constraints by providing a mechanistic rationale for maximizing indium utilization and by establishing m-HfO_2 as an advanced support that surpasses m-ZrO_2. **We have added Supplementary Fig. 1 to highlight this conceptual advance and articulate the transferable structural-electronic descriptors that can guide the design of future reducible oxide catalysts.**

5. The transitions between some sections are not sufficiently smooth or logically coherent. For instance, after presenting experimental results, the manuscript directly proceeds to theoretical calculations without providing a thorough summary of the experimental findings or a clear connection to the calculation part.

We have carefully revised the whole manuscript to streamline section transitions and ensure coherent integration of the experimental and computational results (for example, page 8, lines 14-21; page 10, lines 10-16).

6. All the DFT calculations are at 0 K, overlooking temperature effects and configurational entropy in the thermodynamics. Gibbs free-energy correction should be incorporated.

Temperature effects and Gibbs free-energy corrections have now been incorporated in DFT analyses (see revised manuscript (page 16, lines 10-26) and supplementary information (pages 10-12). The resulting profiles show that the inclusion of thermal and entropic contributions does not appreciably alter the relative energetic and, thus, does not change the mechanistic conclusions.

7. The DFT+U method is widely recognized to better reproduce experimental observables (e.g., lattice constants and band gaps) for metal oxides. Please compare experimental and computed band gaps and justify why DFT+U was not employed.

DFT+U is typically applied for systems with strongly correlated *d* or *f* electrons, which is not the case for HfO_2 or ZrO_2. Although a U correction provides a good accuracy-cost balance to mitigate the well-known underestimation of band gaps by generalized gradient approximation (GGA) based density functionals, there is no universal criterion for selecting an appropriate U value. Indeed, reported studies span a wide range of U values U = 2-11 eV (*ACS Catal.* **5**, 5486 (2015); *J. Adv. Ceram*. **6**, 43 (2017); *Crystals* **12**, 90 (2022); *Catal. Sci. Technol.* **13**, 4387 (2023)), while others use no U correction (*Phys. Rev. B* **58**, 8050 (1998); *Mater. Chem. Phys.* **254**, 123474 (2020); *npj Comput. Mater.* **10**, 188 (2024)). To ensure reliability, **we benchmarked the band gaps of relevant models against higher-level theory methods such as HSE hybrid and r^2SCAN meta-GGA density functionals**. As expected, the band gap of the bulk monoclinic pristine materials computed with PBE underestimates the experimental value by approximately 1.3 eV, and HSE or r^2SCAN reduce this discrepancy only partially (by 0.6-0.7 eV). Importantly, the relative shifts are similar for both Hf- and Zr-containing models. Additionally, the PBE-computed lattice parameters for monoclinic ZrO_2 and HfO_2 agree with the experimental values to within <2%. Given these considerations, PBE offers a consistent and reliable basis for comparing the catalytic impact of these two supports. **The comparison with experimental lattice parameters, and the band gaps computed with different levels of theory, have been added to Supplementary Tables 18 and 19-21).**

8. The Authors are suggested to perform ab initio thermodynamics to assess free-energy changes as a function of temperature and pressure, using the chemical potential of In(OH)3 (molecule in gas phase) and In (atom in the bulk).

Ab initio thermodynamic analyses have been performed to evaluate the free-energy changes as a function of temperature and pressure, using the chemical potentials of $In(OH)_3$ (gas phase) and bulk indium as references (see revised manuscript (page 10 lines 7-9) and supplementary information (pages 8-9 and Supplementary Figs. 22-26). The resulting trends confirm that $In(OH)_3$ species with one oxygen vacancy and deposited on m-HfO_2, m-ZrO_2, t-ZrO_2 are the most stable structures under reaction conditions and are representative of 2InHfO_x and 2InZrO_x catalysts.

9. In-situ DRIFT assigns the vibrational peak of CO_2 adsorption. DFT can also predict the *CO_2 vibrational frequencies to guide the local environment of CO_2 adsorption configuration.

Vibrational analyses of the adsorbed CO_2 species by DFT were performed to complement the in-situ DRIFTS results and clarify the local adsorption configurations. The DRIFTS spectra for InHfO_x and InZrO_x show two main signals belonging to the asymmetric (ν_1) and symmetric stretching (ν_2) C−O modes of adsorbed *CO_3^{2-}. These features are reproduced in the DFT-calculated vibrational frequencies, which are systematically underestimated by 83 cm^{-1} on average, consistent with the known behavior of GGA functionals (*Chem. Soc. Rev.* 2015, 115, 6051). **The computed frequencies and corresponding assignments are summarized in the Supplementary Table 10.**

10. “In@” denotes a substituted indium atom. Please define this notation at its first occurrence in Fig. 4c.

The notation In@ is now defined in the caption of Fig. 4c.

11. In Fig. 6a, *CH_2O formation is identified as the rate-determining step, since the $In(OH)_3$-m-HfO_2 has a higher ΔE value than $In(OH)_3$-m-ZrO_2. Some statements should be clarified.

We have included in Supplementary Fig 46 the full reaction profiles with reaction barriers. The combination of *CH_2O and *CH_3O formation steps are confirmed as the rate-determining step based on the highest calculated ΔE value for $In(OH)_3$-m-HfO_2 and $In(OH)_3$-m-ZrO_2, in agreement with experimental kinetic model.

12. The outlook could be slightly expanded to suggest specific other wide-bandgap, high-κ dielectrics (beyond HfO_2 and ZrO_2) that might be explored based on the proposed design principle, perhaps guided by materials databases.

As discussed in the Introduction, our initial screening of the Materials Project database indicates that m-HfO_2 is currently the only synthetically accessible environmentally benign, and low-toxicity oxide combining a wide band gap, high dielectric constant, and structural anisotropy, attributes that appear essential to the observed promotional effect. Within this design space, the monoclinic baddeleyite lattice uniquely provides the local asymmetry required to stabilize the active indium coordination sphere and promote vacancy formation, while the high dielectric constant facilitates

hydrogenation steps by ensure surface proton and hydride stability. The principles identified here serve as a strong foundation for rational exploration. Several directions can be envisioned, including exploring other asymmetric lattices such as pentavalent transition metal oxides and non-centrosymmetric perovskites. Future studies integrating computational screening and materials synthesis will be essential to determine whether these candidates can provide competitive alternatives to ZrO_2 or HfO_2. **As suggested by the Reviewer, we have expanded the Outlook to reflect these perspectives (page 21, lines 17-21).**

Response to Referees for NNANO-25083313A

Comments in blue | Replies in black

Page, line, figure, or reference numbers refer to the revised files with tracked changes.

Reviewer #1

The reviewer's comments are addressed and the revised manuscript is recommended for acceptance.

We appreciate Reviewer #1's thoughtful evaluation, and we are pleased that the revised manuscript is recommended for assessment.

Reviewer #2

This is a top-tier, potentially field-defining study. The authors have successfully addressed every point raised by Reviewers with substantial new data, rigorous analyses, and clear revisions. The manuscript is now significantly stronger in terms of mechanistic insight, generalizability, and methodological rigor. The work is suitable for publication in Nature Nanotechnology. I recommend acceptance pending incorporation of the minor revisions and clarifications suggested below.

We warmly thank Reviewer #2 for their positive assessment and recognition of the scientific impact of our study. We have carefully addressed the remaining minor revisions as outlined below.

Minor Points & Typos

Page 8, line 176: “Hudium” → “Indium”.

Page 22, line 525: “hearing” → “heating”.

Page 30, line 716: “References” – Please check that all references are formatted consistently (some have full journal names, others abbreviations).

Please ensure all acronyms are defined at first use (e.g., TPSR, HD exchange).

We have carefully revised the manuscript for typographical errors. All references have been formatted to comply with the journal’s requirements. In addition, all acronyms are now defined at their first occurrence.
